# Transition metal-free catalytic reduction of primary amides using an abnormal NHC based potassium complex: integrating nucleophilicity with Lewis acidic activation†

**DOI:** 10.1039/c9sc05953a

**Published:** 2019-12-27

**Authors:** Mrinal Bhunia, Sumeet Ranjan Sahoo, Arpan Das, Jasimuddin Ahmed, Sreejyothi P., Swadhin K. Mandal

**Affiliations:** Department of Chemical Sciences, Indian Institute of Science Education and Research Kolkata Mohanpur-741246 India swadhin.mandal@iiserkol.ac.in

## Abstract

An abnormal N-heterocyclic carbene (aNHC) based potassium complex was used as a transition metal-free catalyst for reduction of primary amides to corresponding primary amines under ambient conditions. Only 2 mol% loading of the catalyst exhibits a broad substrate scope including aromatic, aliphatic and heterocyclic primary amides with excellent functional group tolerance. This method was applicable for reduction of chiral amides and utilized for the synthesis of pharmaceutically valuable precursors on a gram scale. During mechanistic investigation, several intermediates were isolated and characterized through spectroscopic techniques and one of the catalytic intermediates was characterized through single-crystal XRD. A well-defined catalyst and isolable intermediate along with several stoichiometric experiments, *in situ* NMR experiments and the DFT study helped us to sketch the mechanistic pathway for this reduction process unravelling the dual role of the catalyst involving nucleophilic activation by aNHC along with Lewis acidic activation by K ions.

## Abstract 2

An abnormal N-heterocyclic carbene (aNHC) based potassium complex was used as a transition metal-free catalyst for reduction of primary amides to corresponding primary amines under ambient conditions.

## Graphical abstract

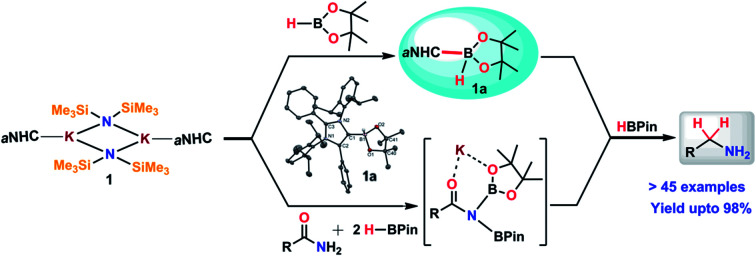

## Introduction

Amines belong to a privileged class of compounds, routinely present in many fine and bulk chemicals, drugs, agrochemicals, dyes, surfactants, detergents and organic materials.^1–6^ One of the most attractive methods for synthesis of amines is the deoxygenative reduction of amides.^7^ However, the reduction of amides is the most challenging among all carboxylic derivatives because of their higher thermodynamic stability. The reduction of primary amides to primary amines is more stringent compared to secondary and tertiary amides.^8^

**Scheme 1:**
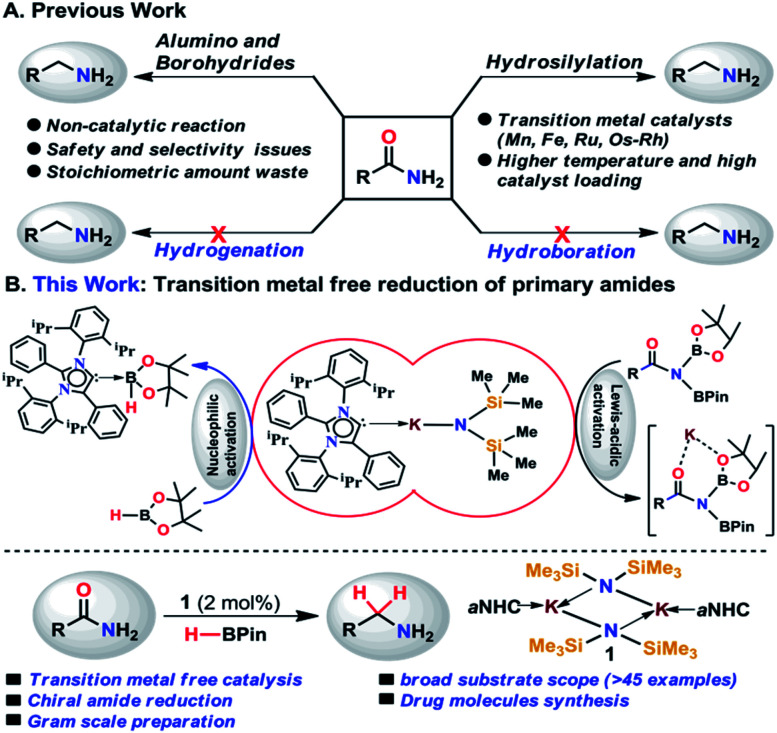
(A) Summary of the reported methods for reduction of primary amides. (B) Overview of the present work where integration of nucleophilicity and Lewis-acidic activation enables the primary amide reduction avoiding transition metals.

Among various synthetic methods to prepare primary amines such as reductive amination of carbonyls,^9–15^ borrowing hydrogenation methodologies,^16–18^ hydroamination of alkynes,^19,20^ reduction of nitriles,^21–24^ primary amides,^25^ and carboxylic acids in the presence of ammonia,^26^ reduction of primary amides is a straightforward route as amides are stable, naturally abundant, and inexpensive. Common methods for reduction of primary amides employing stoichiometric amounts of reactive metal hydride reagents such as lithium aluminum hydride or sodium borohydrides are unattractive in terms of atom economy and environmental issues. In addition, they suffer from safety and selectivity of the process generating a stoichiometric amount of inorganic waste.^7^ In contrast, atom economical catalytic hydrogenation of primary amides is highly desirable and to date no example of catalytic hydrogenation of primary amides has been reported (Scheme 1).^26^

As an alternative, catalytic hydrosilylation is an attractive approach for facile and efficient reduction of primary amides to amines.^27–30^ Earlier, Beller and coworkers utilized two different iron catalysts for efficient reduction of primary amides to primary amines in which the [NHEt_3_][Fe_3_H(CO)_11_] catalyst dehydrates primary amides to nitriles and the second catalyst Fe(OAc)_2_ was used for reduction of nitriles to the corresponding primary amines.^25^ Very recently, our group has developed a single catalyst [Mn^III^(O,N,N,O-PLY)Cl] for hydrosilylation of primary amides into amines.^31^ Only a few examples of catalytic hydrosilylation of primary amides have been reported to date,^25,31–33^ while catalytic deoxygenative hydroboration of primary amides to amines has not been reported to date.

In the present study, we have achieved the reduction of primary amides to amines through deoxygenative hydroboration exploiting an abnormal N-heterocyclic carbene (aNHC) based potassium complex^34^ (**1**) under ambient conditions in which the catalyst acts by integrating the nucleophilicity of weakly bound aNHC and Lewis acidic activation imparted by the K ions. It may be noted that to date a very few NHC–potassium complexes have been known in the literature^35–37^ and in these complexes, NHCs are found to be weakly bound (average bond length ∼ 3.00 Å) primarily by ionic interaction with K ions. Thus, we posed a question, whether a weakly bound N-heterocyclic carbene (NHC) in the form of its potassium complex is able to utilize its σ-donating (nucleophilic) properties in activating a hydride transfer reagent (such as borane) to reduce primary amides in a catalytic fashion. It is interesting to note that very recently we have utilized the superior nucleophilicity of aNHC for the reduction of thermodynamically stable CO_2_ from cylinder^38^ as well as from the atmosphere^39^ including use of CO_2_ for formylation of primary amides.^40^ These findings motivated us to check the catalytic efficacy of the aNHC based potassium complex (**1**) for selective reduction of primary amides under ambient conditions.

## Results and discussion

At first, the catalytic reduction of primary amides was investigated using 4-nitrobenzamide as a model substrate and pinacolborane (HBPin = 4,4,5,5-tetramethyl-1,3,2-dioxaborolane) as the reducing agent. Reaction of 4-nitrobenzamide (0.5 mmol) and HBPin (2.0 mmol) in the presence of catalyst **1** (5 mol%) at 40 °C in toluene afforded 85% yield of 4-nitrobenzyl amine hydrochloride salt within 24 h (see entry 3, Table S1 in the ESI†). Notably, further lowering the catalyst loading and optimizing various solvents, time and equivalents of HBPin used, the optimized condition was realized (83% yield) in the presence of 2 mol% catalyst (**1**) loading in toluene at 40 °C for 12 h when 4 equivalents of HBPin were used (entries 4–11, Table S1, ESI†). Control experiments clearly demonstrated that the deoxygenative hydroboration of 4-nitrobenzamide could not occur in the absence of either catalyst **1** (entries 8, and 12–14, Table S1, ESI†) or combination of aNHC and KN(SiMe_3_)_2_ (entries 15 and 16, Table S1, ESI†).

Having optimized the reaction conditions (entry 8, Table S1, ESI†) in hand, further the scope of the reduction was investigated using various aromatic and aliphatic primary amides to hydroborylated products which essentially upon hydrolysis resulted in the corresponding primary amines and were isolated as their hydrochloride salts (Tables 1 and 2). Under the standardized conditions, benzamide and the diverse range of benzamides bearing electron-donating (Me, OMe, OEt, and ^*t*^Bu) as well as electron-withdrawing functional groups (Cl, NO_2_, and CN) at the *para* position were reduced in good to excellent yields (61–97%) to their corresponding amine hydrochlorides (**3a–3h**, Table 1). Interestingly, in the presence of highly reducible functional groups such as nitro and cyano groups at the *para* position of benzamides, only amine hydrochloride salts were obtained without reduction of these functional groups (**3g–3h**, Table 1). Under similar conditions, a varied range of primary benzamides containing electron-donating and electron-withdrawing groups at the *meta* position (Me, OMe, Cl, CF_3_, and NO_2_) and the *ortho* position (Me, OMe, OEt, F, Cl, and Br) was reduced to the corresponding amines in moderate to excellent yields (50–98%) (**3i–3s**, Table 1). Next, we probed reduction of primary amides in the presence of sterically encumbering groups such as 2,6-dimethoxybenzamide, 2,6-difluorobenzamide, 2-methyl-3-chlorobenzamide, and 2-methyl-5-fluorobenzamide which rendered very good yields (89–93%) of their corresponding benzylamine hydrochloride (**3t–3w**, Table 1), whereas sterically less bulky 3,5-dimethoxybenzamide, 3,4-dimethylbenzamide and 3-methyl-4-bromobenzamide afforded 90%, 91% and 97% yields of the reduced products, respectively (**3x–3z**, Table 1).

**Table 1:** Catalytic reduction of aromatic primary amides to primary amines^a^

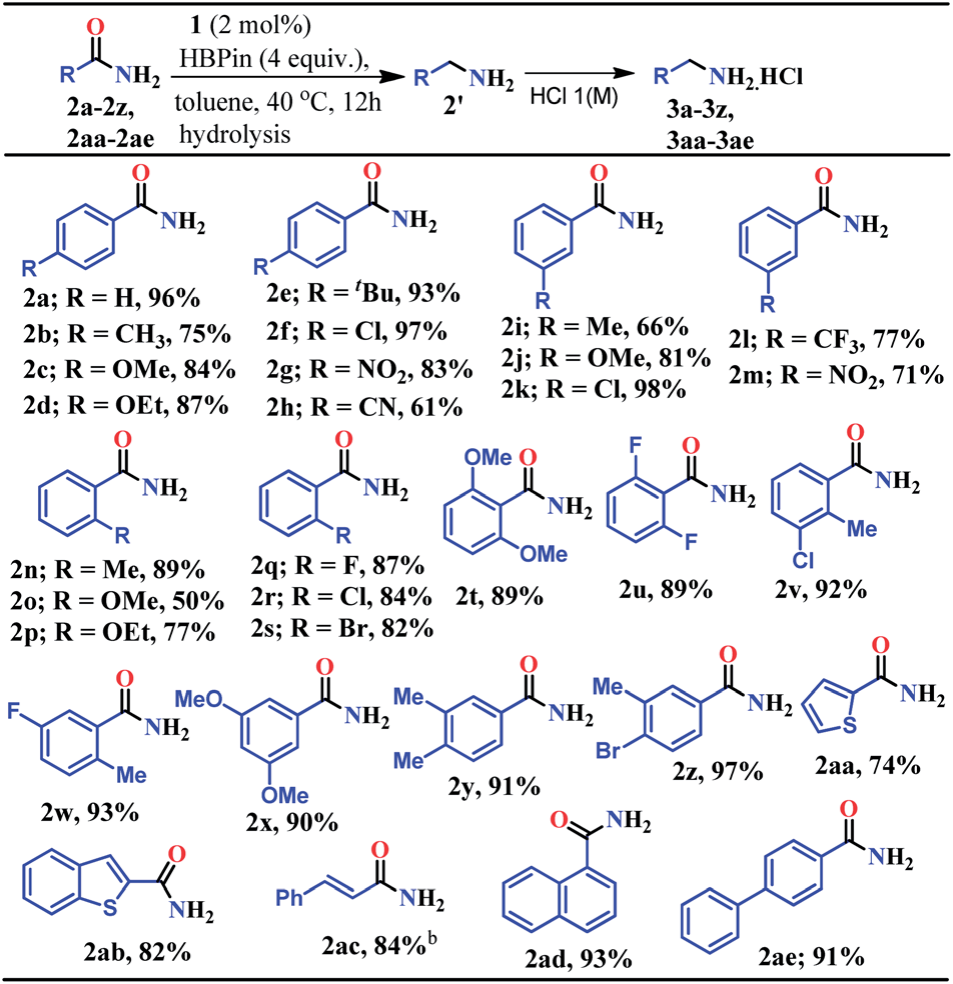

a Reaction conditions: aromatic primary amides (0.5 mmol), HBPin (4.0 mmol), and **1** (2 mol%), toluene, 40 °C, and 12 h. Isolated as amine hydrochloride salt.

b The yields were determined from ^1^H NMR spectroscopy using 1,3,5-trimethoxybenzene as an internal standard.

**Table 2:** Reduction of aliphatic primary amides to primary amines^a^

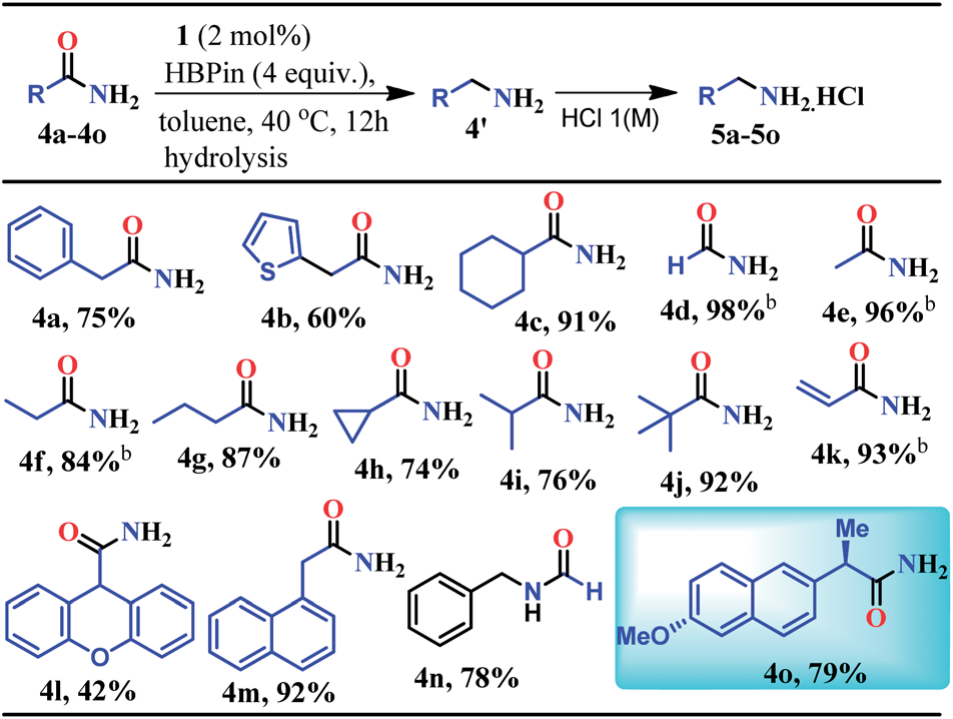

a Reaction conditions: aliphatic primary amides (0.5 mmol), HBPin (4.0 mmol), and **1** (2 mol%), toluene, 40 °C, and 12 h. Isolated as amine hydrochloride salt.

b The yields were determined from ^1^H NMR spectroscopy using 1,3,5-trimethoxybenzene as an internal standard.

Given the importance of the preparation of heterocyclic primary amines for the production of pharmaceutically and agriculturally valuable products, we attempted to reduce thiophene-2-carboxamide and benzothiophene-2-carboxamide which rendered 74% and 82% yields of the corresponding amine hydrochloride, respectively (**3aa–3ab**, Table 1). Interestingly, *trans*-cinnamamide bearing internal alkene afforded 84% yield of the corresponding amine hydrochloride (**3ac**) without reduction of the internal olefin moiety. These results unarguably established that our reduction methodology is appreciably chemoselective in nature where highly reducible groups such as –NO_2_, –CN, and alkene units remained intact (**3g**, **3h**, **3m**, and **3ac**, Table 1). Furthermore, 1-naphthamide and [1,1′-biphenyl]-4-carboxamide also afforded the reduction products in 93% and 91% yields, respectively (**3ad** and **3ae**, Table 1).

Given the fact that many drug molecules contain an aliphatic amine moiety in their structural unit,^41–43^ we employed this optimized reaction condition for the reduction of various aliphatic primary amides to their corresponding primary amines which were also isolated as hydrochloride salts (Table 2). Under the standard reaction conditions, aliphatic amides such as 2-phenylacetamide and 2-(thiophen-2-yl)acetamide furnished good yields (75% and 60%) of their corresponding reduced products (**5a–5b**, Table 2). Moreover, aliphatic primary amides such as cyclohexanecarboxamide, formamide, acetamide, propionamide, butyramide, cyclopropanecarboxamide, isobutyramide, and pivalamide when subjected to reduction with HBPin under the optimized reaction conditions, the corresponding anilinium salts were realized in excellent yield 74–98% (**5c–5j**, Table 2). Notably, selective reduction products of acrylamides and allylamines are ubiquitously present in various biologically active compounds.^44–47^ Interestingly, acrylamides are also well-tolerated by the current catalytic system and rendered 93% yield of the corresponding reduced borylated amines which can produce allyl amines upon hydrolysis and considered as valuable intermediates for a wide range of organic compounds.^48,49^ Interestingly, 2-(naphthalen-1-yl)acetamide, *N*-benzylformamide, heterocyclic primary amide, and 9*H*-xanthene-9-carboxamide afforded very good to excellent yields (42–92%) of their reduced products (**5l–5m**, Table 2). Moreover, *N*-benzylformamide under optimized reaction conditions afforded 78% yield of the corresponding amine hydrochloride (**5n**, Table 2). It may be noted that free amine of **5n** is considered as an intermediate for the preparation of biologically active compounds. Additionally, this reduction methodology was also applicable for chiral amides. Under the standard reaction conditions, (*R*)-2-(6-methoxynaphthalen-2-yl)propanamide rendered 79% yield of the amine which was isolated as the hydrochloride salt (**5o**, Table 2).

**Scheme 2:**
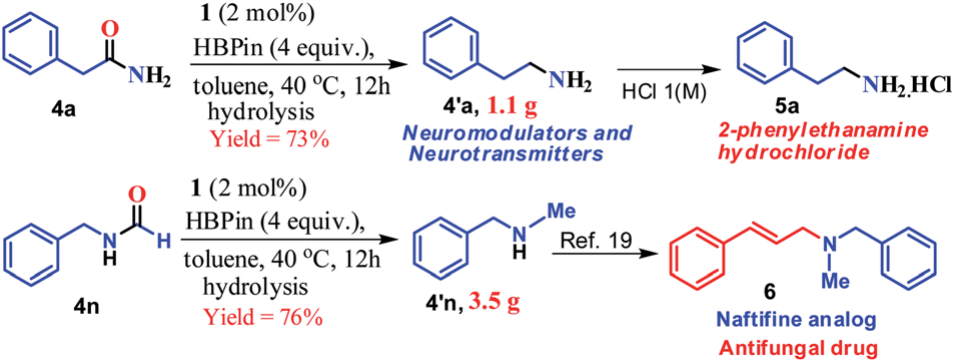
Synthetic route to phenylethylamine and the naftifine analog on a gram scale.

Furthermore, to demonstrate the practical applicability of this transition metal-free reduction protocol, 2-phenylethylamine, a monoaminergic neuromodulator and a neurotransmitter^15^ in the human central nervous system (CNS), was prepared in gram-scale (1.1 g) and isolated as 2-phenylethanamine hydrochloride salt (**5a**, Scheme 2). In addition, *N*-methyl-1-phenylmethanamine can be easily transformed into an antifungal drug naftifine analog, **6**, which was prepared on a gram-scale (76% yield, 3.5 g, Scheme 2) using this transition metal-free protocol.^50^

**Scheme 3:**
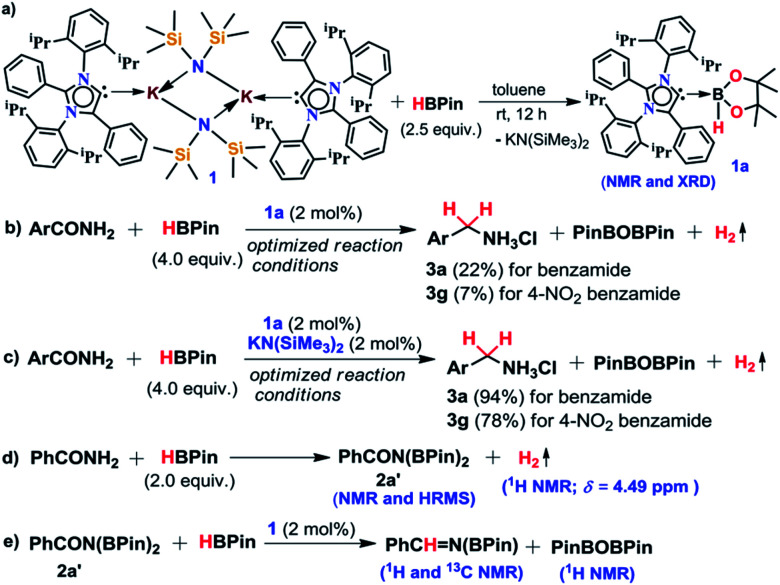
A series of control experiments (a–e) conducted to understand the mechanistic pathway.

**Fig. 1:**
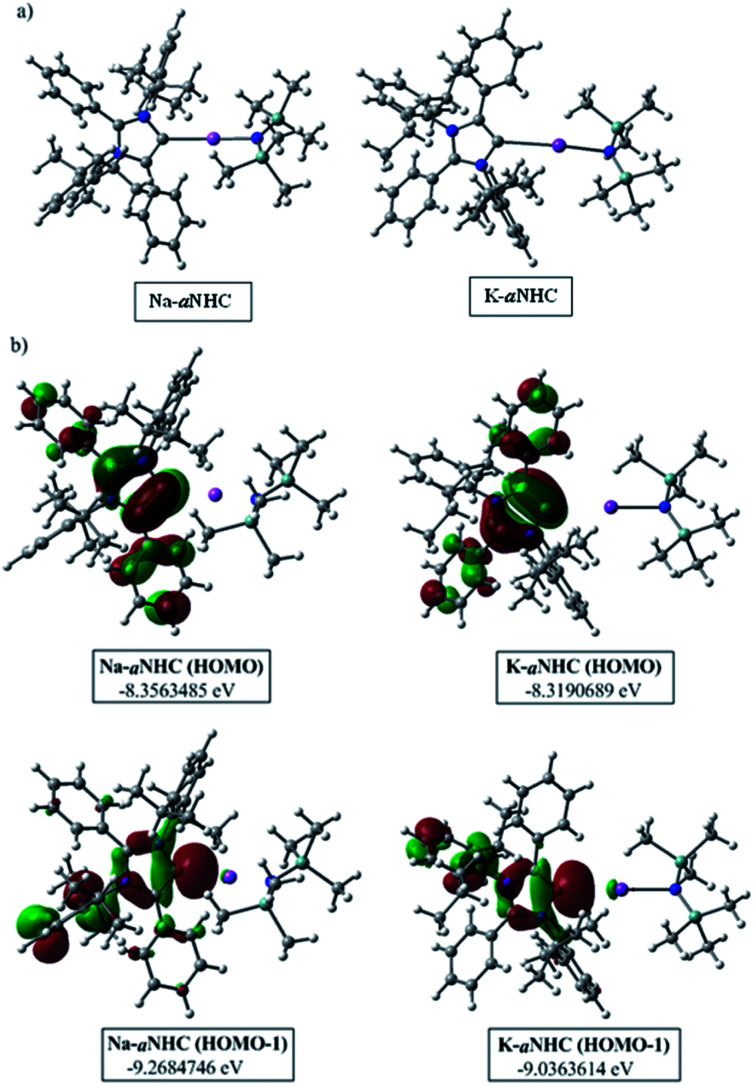
(a) Optimized structures of aNHC–NaN(SiMe_3_)_2_ and aNHC–KN(SiMe_3_)_2_ complexes. (b) HOMO and HOMO−1 of the corresponding optimized species.

**Fig. 2:**
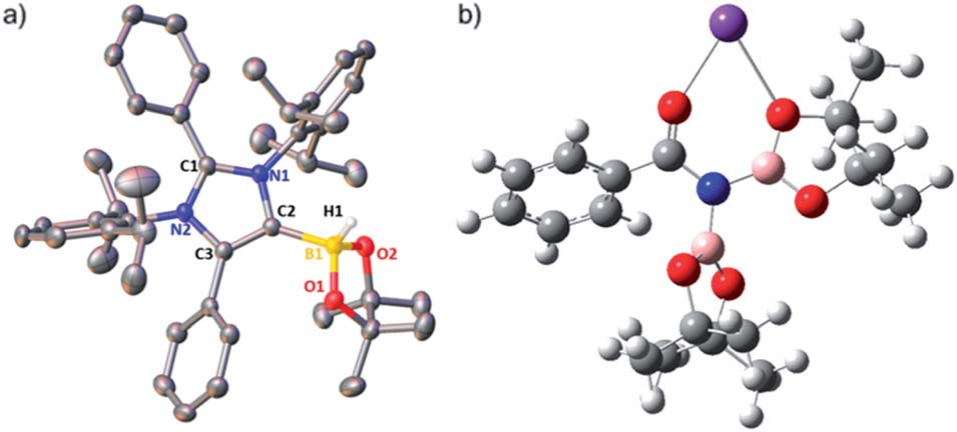
(a) View of the molecular structure of **1a**. Ellipsoids are set at the 50% probability level; hydrogen atoms except H1 of **1a** have been omitted for the sake of clarity. (b) Computationally optimized structure of **7** at the B3LYP/6-31G* level of theory, showing interaction between the K ions and carbonyl oxygen.

The phenomenal catalytic activity of the potassium aNHC complex, **1**, for the reduction of primary amides prompted us to uncover the mechanistic details on the basis of a series of stoichiometric reactions. At first, to find out the role of **1**, we performed the stoichiometric reaction of **1** with pinacolborane in toluene at room temperature which afforded colorless crystals of the aNHC–borane adduct (**1a**) at −30 °C along with formation of KN(SiMe_3_)_2_. Formation of KN(SiMe_3_)_2_ in the reaction mixture was supported by the ^1^H NMR spectrum displaying a resonance at *δ* 0.06 ppm. **1a** was isolated and further characterized using NMR spectroscopy and mass spectrometry as well as using single-crystal X-ray diffraction studies (Scheme 3a). Compound **1a** crystallizes in the triclinic space group *P*1̄ with a single molecule in the asymmetric unit where the carbene–borane bond length was determined as 1.668 (2) Å.^51^ Formation of **1a** in the reaction mixture in the presence of a borane establishes the role of **1** during catalysis. The weak ionic bond between K ion and aNHC in **1** as evident from the bond length (2.973 (2) Å)^34^ makes the free aNHC available for adduct formation with borane. Thus **1** primarily acts as a carrier of aNHC during the catalytic reaction. Furthermore, to corroborate whether **1a** is an active catalyst or not, we accomplished the catalytic reduction of benzamide and 4-nitrobenzamide using the aNHC–borane adduct in the presence of 2 mol% **1a**, when benzamide and 4-nitrobenzamide offered only 22% and 7% yields of the corresponding phenylmethanamine hydrochloride salt, respectively, under the standard reaction conditions (Scheme 3b). On the other hand, the same reaction when performed with catalytic amounts of **1a** (2 mol%) and KN(SiMe_3_)_2_ (2 mol%), an excellent yield (94% for benzamide and 78% for 4-nitrobenzamide) of the reduction product was realized (Scheme 3c). However, only KN(SiMe_3_)_2_ failed to afford any reduction product (*vide supra*). These results clearly suggest that combination of KN(SiMe_3_)_2_ and aNHC is accountable for reduction of primary amides. Next, we performed the catalytic reduction of 4-nitrobenzamide in the presence of aNHC and different MN(SiMe_3_)_2_ (M = Na, and K), and scrutinized the relative yield. When KN(SiMe_3_)_2_ was used, it resulted in 78% yield while NaN(SiMe_3_)_2_ led to only 56% yield (Table S4, ESI†). This clearly indicates the role of alkali metal ions in the catalytic outcome of this reaction. To further investigate the reactivity difference between KN(SiMe_3_)_2_ and NaN(SiMe_3_)_2_, we undertook a density functional theory (B3LYP/6-31+g(2d,p)) analysis (Fig. 1). Optimized geometries clearly exhibited both the HOMO and HOMO−1 are primarily aNHC based (Fig. 1b), and a more destabilized HOMO (−8.3190 eV) and HOMO−1 (−9.0364 eV) for aNHC–KN(SiMe_3_)_2_ infers its better nucleophilicity towards borane (HBPin) in comparison with the sodium analogue which in turn makes the B–H bond more nucleophilic upon adduct formation. Such adduct mediated nucleophilic activation of borane by aNHC has been reported in earlier studies.^38^ Furthermore, when a catalytic amount of less nucleophilic normal NHC (2.0 mol% IPr carbene) along with KN(SiMe_3_)_2_ (2.0 mol%) was used to reduce 4-nitrobenzamide, only 9% yield of the reduction product was realized (78% yield for **1**), suggesting the importance of higher nucleophilicity of aNHC in increasing the electron density on the boron center which promotes the nucleophilic addition of the B–H bond towards the carbonyl group of borylated amides (*vide supra*).^52^ Moreover, a stoichiometric reaction between benzamide and pinacolborane generates *N*-borylated amide (**2a′**), which was characterized through ^1^H, ^13^C and ^11^B NMR spectroscopic studies as well as mass spectrometry (Fig. S6–S8, ESI†). Notably, during the formation of *N*-borylated amide (**2a′**), evolution of hydrogen gas was monitored which was unambiguously supported by ^1^H NMR resonance at *δ* 4.49 ppm (Scheme 3d).^53^ Furthermore, a stoichiometric reaction was performed between *N*-borylated amide (**2a′**) and KN(SiMe_3_)_2_ to substantiate the role of KN(SiMe_3_)_2_. The sharp downfield shift of the carbonyl group (by 9.9 ppm) in the ^13^C NMR spectra (*δ* 179.1 ppm) compared to its borylated amide, **2a′** (*δ* 169.2 ppm) (Fig. S10, ESI†), clearly implies a Lewis acidic interaction between the K ion and carbonyl oxygen which was further supported by the DFT study (*vide infra*, Fig. 2b).^54^ Such a Lewis acidic interaction may enhance the electrophilicity of amide carbonyl functionality. Furthermore, on treatment of catalyst **1** with borylated amide, **2a′**, in the presence of HBPin and stopping the reaction prematurely after 2 h, partial formation of *N*-borylated imine was detected in the ^1^H NMR spectrum at *δ* 10.34 ppm and in the ^13^C NMR spectrum at *δ* 172.7 ppm in benzene-d_6_ for benzamide (Fig. S12 and S13, ESI†). A similar reaction with 4-chlorobenzamide after 2 h exhibited the ^1^H NMR resonance of the corresponding *N*-borylated imine (**9**) at *δ* 9.78 ppm (Fig. S14, ESI†) in toluene-d_8_ (Scheme 3e).^55^ Next, *N*-borylated imine, **9**, reacts with HBPin in the presence of catalyst **1** to accomplish the reduced product *N*-diborylated amine (**10**) which was also characterized through ^1^H, ^13^C and ^11^B NMR spectroscopy.^23^

**Scheme 4:**
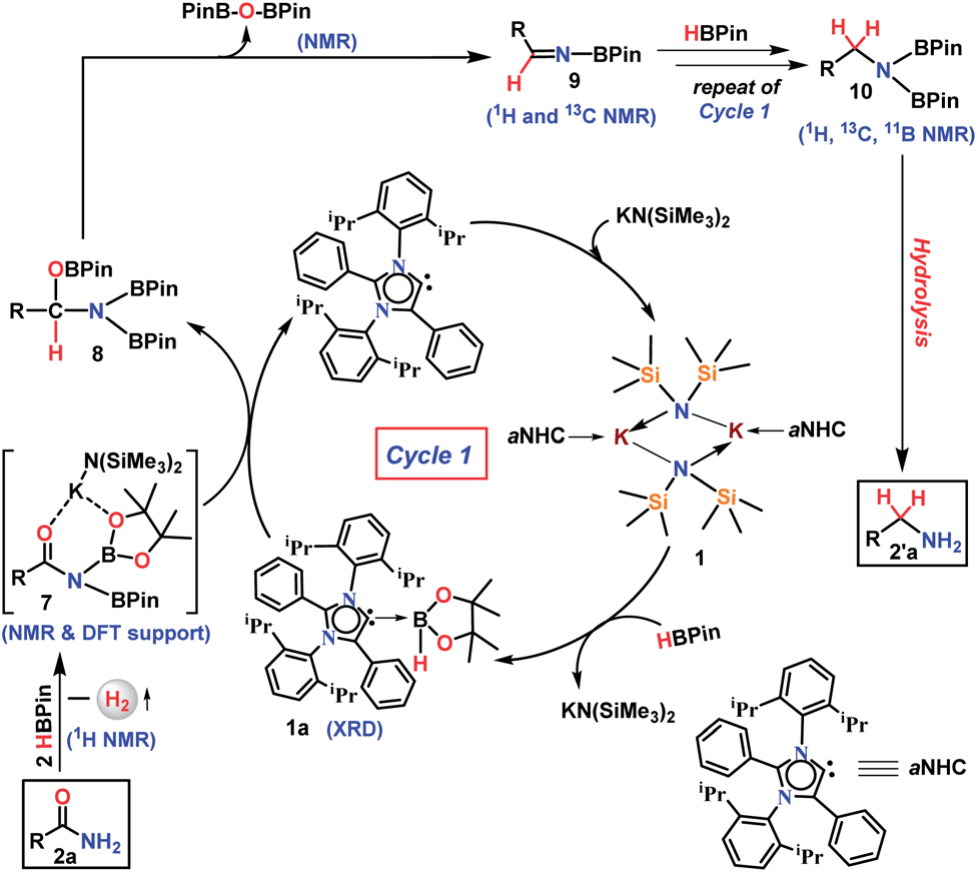
Proposed catalytic cycle based on stoichiometric reactions.

Based on these stoichiometric control experiments described above, a plausible mechanism for this transition metal-free reduction of primary amides is outlined in Scheme 4. In the first step, **1** reacts with pinacolborane to form **1a** (X-ray) where the strong σ-donating property of aNHC increases the nucleophilicity to the B–H bond of borane (Fig. 2a). The organometallic K–aNHC complex acts as the carrier of aNHC which transfers the weakly bound aNHC to borane and generates KN(SiMe_3_)_2_. Next, KN(SiMe_3_)_2_ increases the electrophilicity of borylated amide, through Lewis-acidic interactions (evinced by NMR spectroscopy and the DFT calculated structure of **7**, Fig. 2b).

On the other hand, the aNHC–borane adduct, **1a**, reacts with activated borylated amide, **7**, to form an intermediate borylated amine ester, **8**. Strikingly, deoxygenative reduction of the carbonyl groups in isocyanate and formylated amines was carried out through hydroboration using alkaline earth metals which involved a similar hydride attack on carbonyl carbon to produce methylamine derivatives.^56,57^ Also, it may be noted that the borylated amide can be formed by the dehydrogenation reaction of benzamide and pinacolborane (as evidenced by stoichiometric experiments, see Scheme 3d) which is further activated by the *in situ* generated KN(SiMe_3_)_2_ through formation of **7** (DFT supported and ^13^C NMR evidence). Next, elimination of a diboryl ether (authenticated by ^1^H and ^11^B NMR spectroscopies) leads to the formation of corresponding *N*-borylated imine, **9**, which was also characterized by ^1^H and ^13^C NMR spectroscopies. The *N*-borylated imine next can undergo further reduction *via* another catalytic cycle (likewise cycle 1) to generate deoxygenative hydroborylated amine, **10**, which was characterized through ^1^H, ^13^C and ^11^B NMR spectroscopy and subsequent hydrolysis produces the corresponding primary amine.

## Conclusions

In conclusion, we have demonstrated a transition metal-free catalytic reduction of primary amides to the corresponding primary amines using a well-defined aNHC based potassium complex through hydroboration. Notably, our reduction protocol exhibited a wide range of substrate scope including aromatic, heteroaromatic, and aliphatic primary amides. Moreover, this reduction methodology is considerably chemo-selective and applicable for chiral amides. Gram-scale production of phenylethylamine, a neuromodulator and neurotransmitter in the human CNS was also executed with this reduction strategy. Furthermore, with the help of a series of stoichiometric control experiments, *in situ* NMR experiments, X-ray crystallography and DFT study, we propose a mechanistic pathway for this challenging transformation where strong σ-donation of aNHC and labile character of organometallic K-aNHC were integrated with Lewis acidity imparted by *in situ* generated KN(SiMe_3_)_2_. Conceivably, the strong σ-donating properties of aNHC activated HBPin for nucleophilic addition of the B–H bond and the Lewis-acidic properties of KN(SiMe_3_)_2_ activated *in situ* generated borylated amide. Such a strategy of merging both nucleophilic and Lewis-acidic activation enables a transition metal free approach towards reduction of various primary amides.

## Experimental section

### General considerations

All manipulations were carried out using standard Schlenk techniques, high vacuum and also glovebox techniques below 0.1 ppm of O_2_ and H_2_O. All glassware were oven-dried at 130 °C and evacuated while hot prior to use. All solvents were distilled from Na/benzophenone prior to use. All other chemicals were purchased from Sigma Aldrich and used as received. Elemental analyses were carried out using a PerkinElmer 2400 CHN analyzer and samples were prepared by keeping under a reduced pressure (10^−2^ mbar) overnight. The FT-IR spectra were recorded by transmission measurements of thin films with a PerkinElmer FT-IR spectrometer Spectrum RXI. The NMR spectra were recorded on a JEOL ECS 400 MHz spectrometer and on a Bruker Avance III 500 MHz spectrometer. All chemical shifts were reported in ppm using tetramethylsilane as a reference. Crystallographic data for structural analysis of **1a** (aNHC–HBPin adduct) were deposited at the Cambridge Crystallographic Data Center, CCDC number 1900619.

### General method for reduction of primary amides

An oven dried 20 mL reaction tube was charged with [aNHC·KN(SiMe_3_)_2_]_2_, **1** (14.8 mg, 2 mol%), and pinacolborane (290 μL, 2.0 mmol, 4 equivalents) along with 1 mL toluene inside a N_2_ filled glovebox. Next, primary amides (0.5 mmol, 1 equivalent) were added to the reaction mixture and stirred for 12 h at 40 °C. After completion of the reaction, 1.0 mL 2.0 (M) NaOH solution was added to the reaction mixture along with 1.0 mL Et_2_O and stirred for another 1 h. Next, the reaction mixture was worked up with an Et_2_O : H_2_O mixture (1 : 1) and the corresponding reduced amines were concentrated in a vacuum. Consequently, 1.0 mL 1.0 (M) HCl was added to the concentrated amines followed by addition of 1.0 mL Et_2_O and the corresponding amine hydrochloride salt was purified by washing with Et_2_O. The isolated amine hydrochloride salts were characterized through NMR spectroscopy in DMSO-d_6_.

### Computational details

All theoretical calculations for geometry optimization and Natural Bonding Orbital (NBO) analysis of all the complexes were carried out with the help of Gaussian 16 (ref. 58) at the B3LYP level of theory by using the 6-31+g(2d,p) basis set.^59^

### X-ray crystallographic details

A single crystal of compound **1a** was mounted on a glass tip. Intensity data were collected on a SuperNova, Dual, Mo at zero, Eos diffractometer. The crystals were kept at 100 K during data collection. Atomic coordinates, isotropic and anisotropic displacement parameters of all the non-hydrogen atoms of two compounds were refined using Olex2,^60^ and the structure was solved with the Superflip^61^ structure solution program using charge flipping and refined with the ShelXL^62^ refinement package using least squares minimization. Structure graphics shown in the figures were created using Olex2 and X-Seed software (version 2.0) packages.^63^ Crystallographic data for structural analysis of **1a** were deposited at the Cambridge Crystallographic Data Center, CCDC number 1900619.

## Conflicts of interest

There is no conflict to declare.

## Supplementary Material

SC-011-C9SC05953A-s001

SC-011-C9SC05953A-s002
