# Supplementary material for: Transition metal-free catalytic reduction of primary amides using an abnormal NHC based potassium complex: integrating nucleophilicity with Lewis acidic activation
Source: Chem Sci. 2019 Dec 27;11(7):1848–54. doi: 10.1039/c9sc05953a (PMC8148392; doi:10.1039/c9sc05953a)
Supplement: SC-011-C9SC05953A-s001 [file SC-011-C9SC05953A-s001.pdf]

Electronic Supplementary Material (ESI) for Chemical Science.
This journal is © The Royal Society of Chemistry 2019

Transition Metal-Free Catalytic Reduction of Primary Amides Using an Abnormal NHC based Potassium Complex: Integrating Nucleophilicity with Lewis Acidic Activation

Mrinal Bhunia†, Sumeet Ranjan Sahoo†, Arpan Das, Jasimuddin Ahmed, Sreejyothi P. and Swadhin K. Mandal*[a]

[a]Department of Chemical Sciences, Indian Institute of Science Education and Research-Kolkata, Mohanpur-741246, India

Contents

1. **Materials and Methods……………………………………………………………….2**
2. **Procedure for optimization of 4-nitrobenzamide reduction……………………………2**
3. **General method for reduction of primary amides………………………………………4**
4. **Application of current methodology in gram scale preparation………………………24**
5. **Control experiments for mechanistic investigation…………………………………...25**
6. **NMR data of primary amine derivatives upon reduction of primary amides……….40**
7. **Computational details and Coordinates………………………………………………83**
8. **References……………………………………………………………………………..92**

1. Materials and Methods.

The pre-catalyst [*a*NHC.KN$(SiMe_3)_2$]$_2$ was prepared by following reported literature procedure.[1] All manipulations were carried out using standard Schlenk techniques using high-vacuum or inside a glovebox maintained below 0.1 ppm of O_2 and H_2O. All glassware were oven-dried at 130 °C and evacuated while hot prior to use. All solvents were distilled from Na/benzophenone prior to use. All other chemicals were purchased from Sigma Aldrich and used as received. Elemental analyses were carried out using a Perkin-Elmer 2400 CHN analyzer and samples were prepared by keeping under reduced pressure (10^{-2} mbar) for overnight. Analytical TLC was performed on a Merck 60F254 silica gel plate (0.25 mm thickness). NMR spectra were recorded on a JEOL ECS 400 MHz spectrometer and on a Bruker Avance III 500 MHz spectrometer. All chemical shifts were reported in ppm using tetramethylsilane as a reference. Crystallographic data for structural analysis of **1a** was deposited at the Cambridge Crystallographic Data Center, CCDC number 1900619. These data can be obtained free of charge from the Cambridge Crystallographic Data Center.

2. Procedure for optimization of 4-nitrobenzamide reduction.

An oven dried 20 mL reaction tube was charged with [*a*NHC.KN$(SiMe_3)_2$]$_2$, **1** (14.8 mg, 2 mol%) and pinacolborane (290 μL, 2.0 mmol, 4 equivalent) along with 1 mL solvent inside a N_2 filled glovebox. Subsequently, 4-nitrobenzamide (0.5 mmol) was added to the reaction mixture and stirred for different time interval at different temperature (°C). After completion of the reaction, 1.0 mL 2.0 (M) NaOH solutions was added to the reaction mixture drop-wise along with 1.0 mL Et_2O and stirred for another 1h. Next, the reaction mixture was worked up with Et_2O:H_2O mixture (1:1) and the corresponding reduced amines were concentrated in vacuum. Subsequently, 1.0 mL 1.0 (M) HCl was added to the concentrated amines followed by addition of 1.0 mL Et_2O and the corresponding (4-nitrophenyl)methanamine hydrochloride salt was purified by washing with Et_2O and characterized through NMR spectroscopy in DMSO-d_6. To find out the superiority of *a*NHC over NHC (Chart 1), optimization reaction was carried out with catalytic *a*NHC (2 mol%) as well as IPr (2 mol%) carbene along with KHMDS (2 mol%) (Table S1, entries 15 and 16).

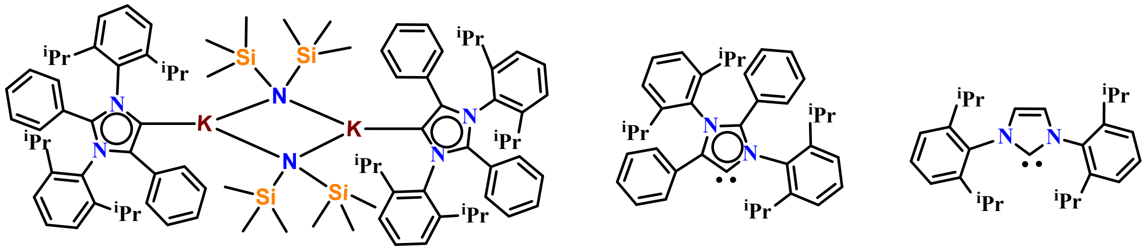

1 *a*NHC IPr

Chart S1. Molecular drawings of catalyst **1**, *a*NHC, and IPr, used for reduction of 4-nitrobenzamide.

Table S1. Optimization of the reaction conditions for the reduction of 4-nitrobenzamide, **2f**.[a]

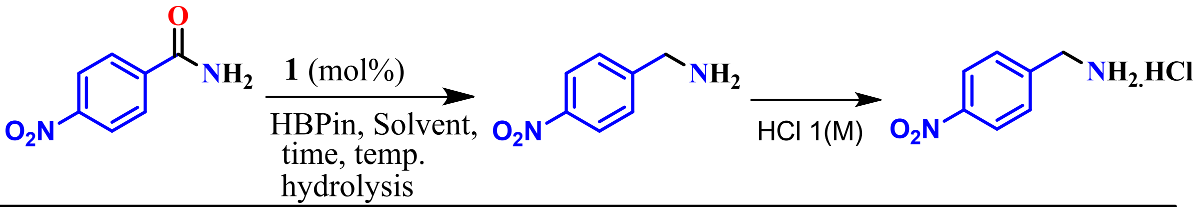

Entry	Catalyst 1 (mol%)	HBpin (equiv)	Solvent	Temp. (° C)	Time (h)	Yield (%)[b]
1	5	4	THF	rt	24	<5
2	5	4	Toluene	rt	24	12
3	5	4	Toluene	40	24	85
4	2	4	Toluene	40	24	84
5	2	4	Benzene	40	24	49
6	1	4	Toluene	40	24	47
7	2	4	Neat	40	24	34
8	**2**	**4**	**Toluene**	**40**	**12**	**83**
9	2	4	Toluene	40	8	71
10	2	3	Toluene	40	12	32
11	2	2	Toluene	40	12	<5
12	*a*NHC (4 mol%)	4	Toluene	40	12	11
13	KHMDS (4 mol%)	4	Toluene	40	12	—
14[c]	—	4	Toluene	40	12	<5
15	[*a*NHC + KHMDS] (2 mol%)	4	Toluene	40	12	78
16	[IPr + KHMDS] (2 mol%)	4	Toluene	40	12	9

[a]Reaction conditions: Catalyst **1** (2.0 mol %), HBPin (2.0 mmol, 4.0 equiv.), 4-nitrobenzamide (0.5 mmol), toluene (1.0 mL), temperature (°C), time (h). Hydrolysis was performed with 2.0 (M) NaOH solution. [b]All yields referred are isolated yields. [c]Reaction was carried out without using any catalyst.

3. General method for reduction of primary amides.

An oven dried 20 mL reaction tube was charged with [*a*NHC.KN(SiMe$_3$)$_2$]$_2$, **1** (14.8 mg, 2 mol%) and pinacolborane (290 µL, 2.0 mmol, 4 equivalent) along with 1 mL toluene inside a N_2 filled glovebox. Subsequently, primary amides (0.5 mmol) were added to the reaction mixture and stirred for 12h at 40 °C. After completion of the reaction, 1.0 mL 2.0 (M) NaOH solutions was added to the reaction mixture along with 1.0 mL Et_2O and stirred for another 1 h. Next, the reaction mixture was worked up with Et_2O:H_2O mixture (1:1) and the corresponding reduced amines were concentrated in vacuum. Consequently, 1.0 mL 1.0 (M) HCl was added to the concentrated amines followed by addition of 1.0 mL Et_2O and the corresponding amine hydrochloride salt was purified by washing with Et_2O. Isolated amine hydrochlorides were characterized through NMR spectroscopy in DMSO-d$_6$.

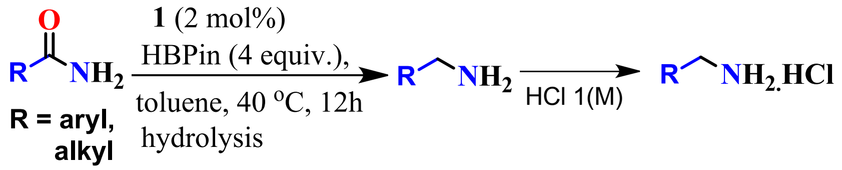

Scheme S1. Reduction of primary amides, catalyzed by **1**.

Phenylmethanamine hydrochloride (3a).[S2-S5]

The general procedure was followed for the synthesis of phenylmethanamine, **2'a**. The reaction was performed with benzamide, **2a** (60.6 mg, 0.5 mmol), HBPin (290 µL, 2.0 mmol), **1** (14.8 mg, 0.01 mmol, 2 mol%) and dry toluene (1.0 mL). The desired product phenylmethanamine, **2'a** was isolated as phenylmethanamine hydrochloride salt, **3a** (68.9 mg, 96% yield) as a colorless solid.

^{1}H NMR (400 MHz, DMSO-d$_6$): δ 8.60 (bs, 3H), 7.50 (d, J = 6.4 Hz, 2H), 7.41-7.33 (m, 3H), 3.99 (q, J = 5.6 Hz, 2H) ppm.

^{13}C{^{1}H} (100 MHz, DMSO-d$_6$): δ 134.1, 129.0, 128.6, 128.4, 42.2 ppm.

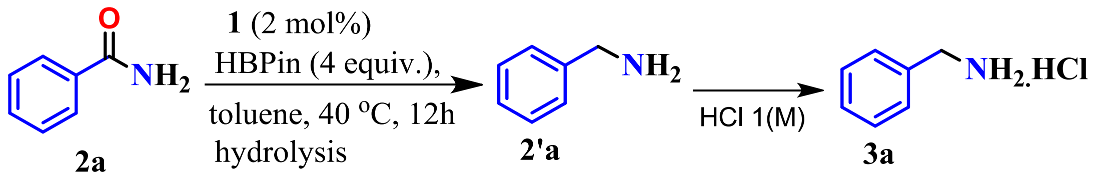

***p*-tolylmethanamine hydrochloride (3b).**[S2]

The general procedure was followed for the synthesis of *p*-tolylmethanamine, **2'b**. The reaction was performed with *p*-toluamide, **2b** (67.6 mg, 0.5 mmol), HBPin (290 µL, 2.0 mmol), **1** (14.8 mg, 0.01 mmol, 2 mol%) and dry toluene (1.0 mL). The desired product *p*-tolylmethanamine, **2'b** was isolated as *p*-tolylmethanamine hydrochloride salt, **3b** (59.1 mg, 75% yield) as a colorless solid.

^{1}H NMR (400 MHz, DMSO-d$_6$): δ 8.52 (bs, 3H), 7.38 (d, J = 7.2 Hz, 2H), 7.19 (d, J = 7.2 Hz, 2H), 3.93 (q, J = 4.4 Hz, 2H), 2.29 (s, 3H) ppm.

^{13}C{^{1}H} (100 MHz, DMSO-d$_6$): δ 137.6, 131.0, 129.0, 128.9, 41.8, 20.7 ppm.

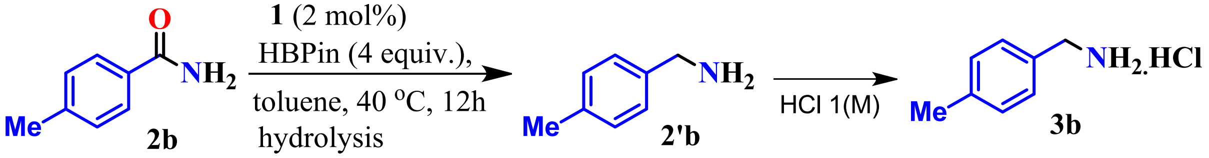

(4-methoxyphenyl)methanamine hydrochloride (3c).[S2-S3]

The general procedure was followed for the synthesis of (4-methoxyphenyl)methanamine, **2'c**. The reaction was performed with 4-methoxybenzamide, **2c** (75.6 mg, 0.5 mmol), HBPin (290 µL, 2.0 mmol), **1** (14.8 mg, 0.01 mmol, 2 mol%) and dry toluene (1.0 mL). The desired product (4-methoxyphenyl)methanamine, **2'c** was isolated as (4-methoxyphenyl)methanamine hydrochloride salt, **3c** (72.9 mg, 84% yield) as a colorless solid.

^{1}H NMR (400 MHz, DMSO-d$_6$): δ 8.45 (bs, 3H), 7.39 (d, J = 8.8 Hz, 2H), 6.90 (d, J = 8.8 Hz, 2H), 3.87 (q, J = 5.6 Hz, 2H), 3.70 (s, 3H) ppm.

^{13}C{^{1}H} (100 MHz, DMSO-d$_6$): δ 159.3, 130.6, 126.0, 113.9, 55.2, 41.6 ppm.

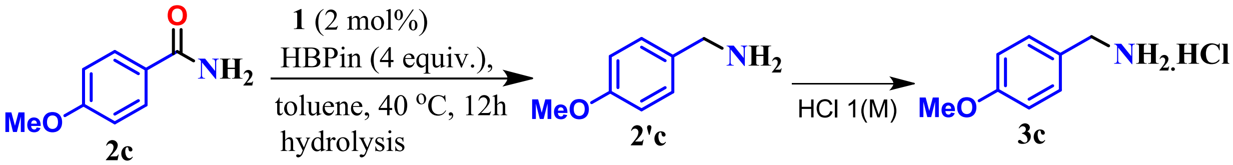

(4-ethoxyphenyl)methanamine hydrochloride (3d).[S3]

The general procedure was followed for the synthesis of (4-ethoxyphenyl)methanamine, **2'd**. The reaction was performed with 4-ethoxybenzamide, **2d** (82.6 mg, 0.5 mmol), HBPin (290 µL, 2.0 mmol), **1** (14.8

mg, 0.01 mmol, 2 mol%) and dry toluene (1.0 mL). The desired product (4-ethoxyphenyl)methanamine, **2'd** was isolated as (4-ethoxyphenyl)methanamine hydrochloride salt, **3d** (81.6 mg, 87% yield) as a colorless solid.

^{1}H NMR (400 MHz, DMSO-d$_6$): δ 8.44 (bs, 3H), 7.40 (d, J = 8.4 Hz, 2H), 6.91 (d, J = 8.4 Hz, 2H), 4.0 (q, J = 6.8 Hz, 2H), 3.90 (q, J = 4.0 Hz, 2H), 1.29 (t, J = 6.8 Hz, 3H) ppm.

^{13}C{^{1}H} (100 MHz, DMSO-d$_6$): δ 158.7, 130.7, 125.9, 114.5, 63.2, 41.8, 14.7 ppm.

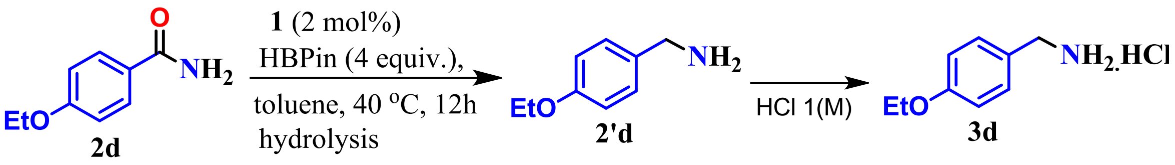

(4-(tert-butyl)phenyl)methanamine hydrochloride (3e).[S2]

The general procedure was followed for the synthesis of (4-(tert-butyl)phenyl)methanamine, **2'e**. The reaction was performed with 4-(tert-butyl)benzamide, **2e** (88.6 mg, 0.5 mmol), HBPin (290 µL, 2.0 mmol), **1** (14.8 mg, 0.01 mmol, 2 mol%) and dry toluene (1.0 mL). The desired product (4-(tert-butyl)phenyl)methanamine, **2'e** was isolated as (4-(tert-butyl)phenyl)methanamine hydrochloride salt, **3e** (92.9 mg, 93% yield) as a colorless solid.

^{1}H NMR (400 MHz, DMSO-d$_6$): δ 8.48 (bs, 3H), 7.42 (s, 4H), 3.95 (q, J = 6.0 Hz, 2H), 1.27 (s, 9H) ppm.

^{13}C{^{1}H} (100 MHz, DMSO-d$_6$): δ 150.9, 131.2, 128.8, 125.3, 41.8, 34.3, 31.1 ppm.

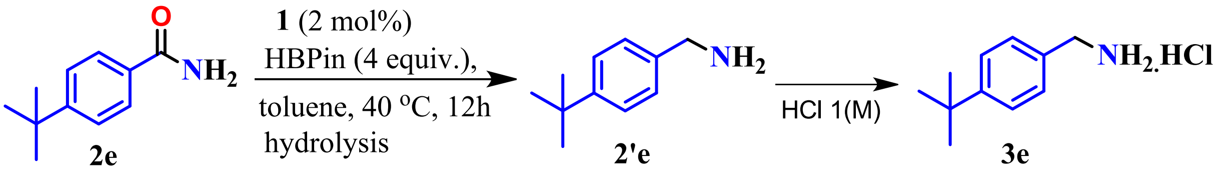

(4-chlorophenyl)methanamine hydrochloride (3f).[S2]

The general procedure was followed for the synthesis of (4-chlorophenyl)methanamine, **2'f**. The reaction was performed with 4-chlorobenzamide, **2f** (73.1 mg, 0.5 mmol), HBPin (290 µL, 2.0 mmol), **1** (14.8 mg, 0.01 mmol, 2 mol%) and dry toluene (1.0 mL). The desired product (4-chlorophenyl)methanamine, **2'f** was isolated as (4-chlorophenyl)methanamine hydrochloride salt, **3f** (51.4 mg, 97% yield) as a colorless solid.

^{1}H NMR (400 MHz, DMSO-d$_6$): δ 8.64 (bs, 3H), 7.55 (d, J = 8.4 Hz, 2H), 7.47 (d, J = 8.0 Hz, 2H), 3.99 (q, J = 5.2 Hz, 2H) ppm.

^{13}C{^{1}H} (100 MHz, DMSO-d$_6$): δ 133.2, 133.0, 131.0, 128.4, 41.3 ppm.

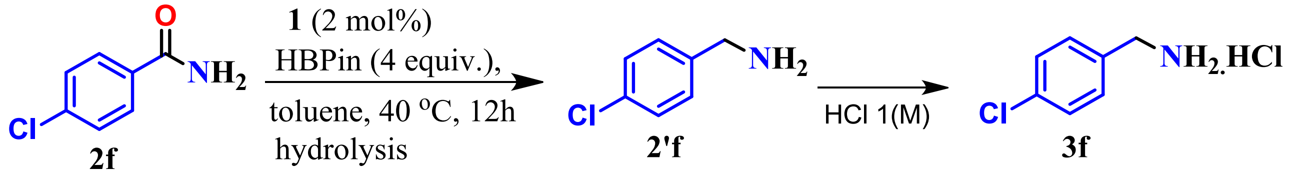

(4-nitrophenyl)methanamine hydrochloride (3g).[S3]

The general procedure was followed for the synthesis of (4-nitrophenyl)methanamine, **2'g**. The reaction was performed with 4-nitrobenzamide, **2g** (83.1 mg, 0.5 mmol), HBPin (290 µL, 2.0 mmol), **1** (14.8 mg, 0.01 mmol, 2 mol%) and dry toluene (1.0 mL). The desired product (4-nitrophenyl)methanamine, **2'g** was isolated as (4-nitrophenyl)methanamine hydrochloride salt, **3g** (78.3 mg, 83% yield) as a brown solid.

^{1}H NMR (500 MHz, DMSO-d$_6$): δ 8.87 (bs, 3H), 8.24 (d, J = 9.0 Hz, 2H), 7.81 (d, J = 8.5 Hz, 2H), 4.17 (q, J = 4.4 Hz, 2H) ppm.

^{13}C{^{1}H} (100 MHz, DMSO-d$_6$): δ 147.4, 141.7, 130.3, 123.5, 41.4 ppm.

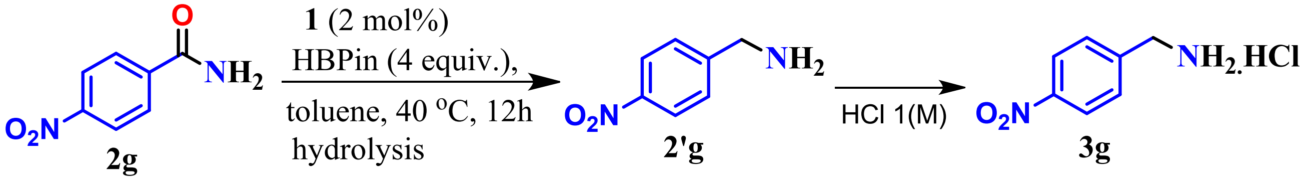

4-(aminomethyl)benzonitrile hydrochloride (3h).[S6]

The general procedure was followed for the synthesis of 4-(aminomethyl)benzonitrile, **2'h**. The reaction was performed with 4-cyanobenzamide, **2h** (73.1 mg, 0.5 mmol), HBPin (290 µL, 2.0 mmol), **1** (14.8 mg, 0.01 mmol, 2 mol%) and dry toluene (1.0 mL). The desired product 4-(aminomethyl)benzonitrile, **2'h** was isolated as 4-(aminomethyl)benzonitrile hydrochloride salt, **3h** (51.4 mg, 61% yield) as a white solid.

^{1}H NMR (400 MHz, DMSO-d$_6$): δ 8.77 (bs, 3H), 7.88 (d, J = 8.4 Hz, 2H), 7.73 (d, J = 7.6 Hz, 2H),4.11 (q, J = 5.2 Hz, 2H), 2.31 (s, 3H) ppm.

^{13}C{^{1}H} (100 MHz, DMSO-d$_6$): δ 139.7, 132.4, 129.9, 118.6, 111.1, 41.6 ppm.

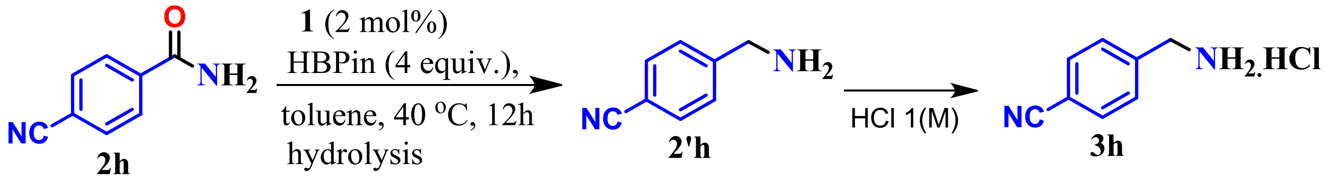

_m_-tolylmethanamine hydrochloride (3i).[S2]

The general procedure was followed for the synthesis of *m*-tolylmethanamine, **2'i**. The reaction was performed with 3-methylbenzamide, **2i** (67.6 mg, 0.5 mmol), HBPin (290 μL, 2.0 mmol), **1** (14.8 mg, 0.01 mmol, 2 mol%) and dry toluene (1.0 mL). The desired product m-tolylmethanamine, **2'i** was isolated as *m*-tolylmethanamine hydrochloride salt, **3i** (52.0 mg, 66% yield) as a colorless solid.

^{1}H NMR (400 MHz, DMSO-d$_6$): δ 8.59 (bs, 3H), 7.32-7.26 (m, 3H), 7.17 (d, J = 6.8 Hz, 1H), 3.94 (q, J = 5.2 Hz, 2H), 2.30 (s, 3H) ppm.

^{13}C{^{1}H} (100 MHz, DMSO-d$_6$): δ 137.7, 134.0, 129.5, 128.9, 128.5, 126.0, 42.1, 21.0 ppm.

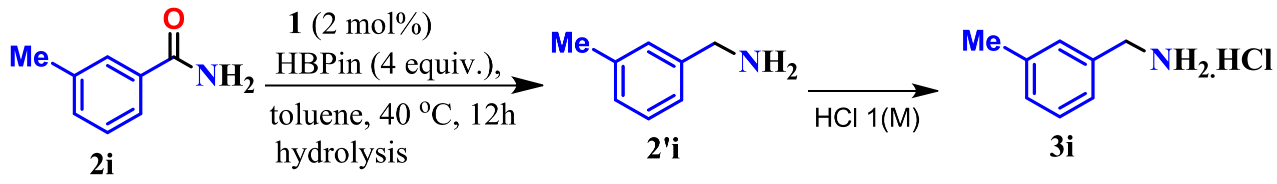

(3-methoxyphenyl)methanamine hydrochloride (3j).[S7]

The general procedure was followed for the synthesis of (3-methoxyphenyl)methanamine, **2'j**. The reaction was performed with 3-methoxybenzamide, **2j** (75.6 mg, 0.5 mmol), HBPin (290 μL, 2.0 mmol), **1** (14.8 mg, 0.01 mmol, 2 mol%) and dry toluene (1.0 mL). The desired product (3-methoxyphenyl)methanamine, **2'j** was isolated as (3-methoxyphenyl)methanamine hydrochloride salt, **3j** (70.3 mg, 81% yield) as a colorless solid.

^{1}H NMR (400 MHz, DMSO-d$_6$): δ 8.67 (bs, 3H), 7.28 (t, J = 6.4 Hz, 1H), 7.18 (s, 1H), 7.05 (d, J = 6.0 Hz, 1H), 6.91-6.89 (m, J = 4.8 Hz, 1H), 3.95 (q, J = 4.4 Hz, 2H), 3.75 (s, 3H) ppm.

^{13}C{^{1}H} (125 MHz, DMSO-d$_6$): δ 159.4, 135.6, 129.7, 121.0, 114.6, 114.0, 55.3, 42.1 ppm.

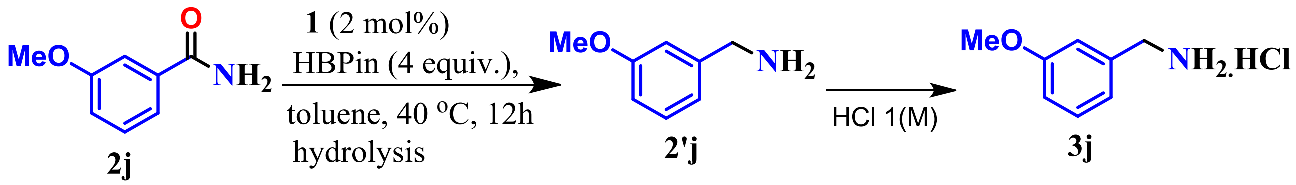

(3-chlorophenyl)methanamine hydrochloride (3k). [S2]

The general procedure was followed for the synthesis of (3-chlorophenyl)methanamine, **2'k**. The reaction was performed with 3-chlorobenzamide, **2k** (77.8 mg, 0.5 mmol), HBPin (290 µL, 2.0 mmol), **1** (14.8 mg, 0.01 mmol, 2 mol%) and dry toluene (1.0 mL). The desired product (3-chlorophenyl)methanamine, **2'k** was isolated as (3-chlorophenyl)methanamine hydrochloride salt, **3k** (87.2 mg, 98% yield) as a colorless solid.

^{1}H NMR (400 MHz, DMSO-d$_6$): δ 8.68 (bs, 3H), 7.65 (s, 1H), 7.50-7.48 (m, 1H), 7.43-7.42 (m, 2H), 4.02 (q, J = 5.6 Hz, 2H) ppm.

^{13}C{^{1}H} (100 MHz, DMSO-d$_6$): δ 136.6, 133.0, 130.4, 129.0, 128.3, 127.9, 41.5 ppm.

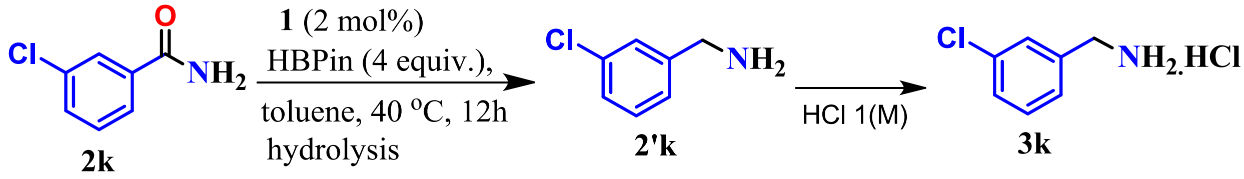

(3-(trifluoromethyl)phenyl)methanamine hydrochloride (3l).[S5]

The general procedure was followed for the synthesis of (3-(trifluoromethyl)phenyl)methanamine, **2'l**. The reaction was performed with 3-(trifluoromethyl)benzamide, **2l** (94.6 mg, 0.5 mmol), HBPin (290 µL, 2.0 mmol), **1** (14.8 mg, 0.01 mmol, 2 mol%) and dry toluene (1.0 mL). The desired product (3-(trifluoromethyl)phenyl)methanamine, **2'l** was isolated as (3-(trifluoromethyl)phenyl)methanamine hydrochloride salt, **3l** (81.5 mg, 77% yield) as a colorless solid.

^{1}H NMR (400 MHz, DMSO-d$_6$): δ 8.70 (bs, 3H), 7.95 (s, 1H), 7.84 (d, J = 7.6 Hz, 1H), 7.73 (d, J = 8.0 Hz, 1H), 7.64 (t, J = 8.0 Hz, 1H), 4.12 (s, 2H) ppm.

^{13}C{^{1}H} (100 MHz, DMSO-d$_6$): δ 135.6, 133.4, 129.6, 129.3 ($J_{C\text{-}F}$ = 31.5 Hz), 125.9 ($J_{C\text{-}F}$ = 3.7 Hz), 125.1 ($J_{C\text{-}F}$ = 3.8 Hz),124.1 ($J_{C\text{-}F}$ = 271.0 Hz), 41.5 ppm.

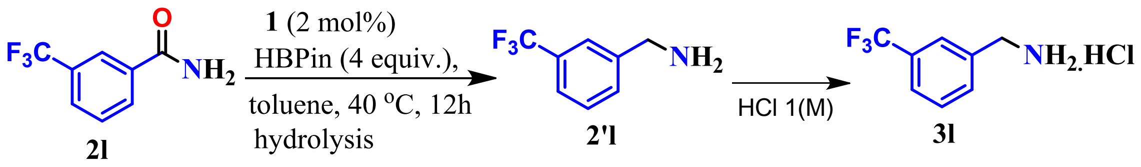

(3-nitrophenyl)methanamine hydrochloride (3m).[S8]

The general procedure was followed for the synthesis of (3-nitrophenyl)methanamine, **2'm**. The reaction was performed with 3-nitrobenzamide, **2m** (83.1 mg, 0.5 mmol), HBPin (290 µL, 2.0 mmol), **1** (14.8 mg, 0.01 mmol, 2 mol%) and dry toluene (1.0 mL). The desired product (3-nitrophenyl)methanamine, **2'm** was isolated as (3-nitrophenyl)methanamine hydrochloride salt, **3m** (66.9 mg, 71% yield) as a pale brown color solid.

^{1}H NMR (500 MHz, DMSO-d$_6$): δ 8.74 (bs, 3H), 8.46 (s, 1H), 8.22 (d, J = 8.5 Hz, 1H), 8.0 (d, J = 7.5 Hz, 1H), 7.71 (t, J = 8.5 Hz, 1H), 4.18 (q, J = 6.0 Hz, 2H) ppm.

^{13}C{^{1}H} (100 MHz, DMSO-d$_6$): δ 147.7, 136.3, 136.1, 130.2, 124.1, 123.4, 41.4 ppm.

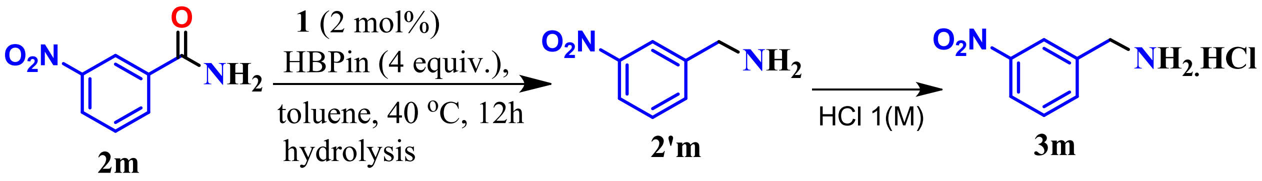

***o*-tolylmethanamine hydrochloride (3n).**[S2]

The general procedure was followed for the synthesis of *o*-tolylmethanamine, **2'n**. The reaction was performed with 2-methylbenzamide, **2n** (67.6 mg, 0.5 mmol), HBPin (290 µL, 2.0 mmol), **1** (14.8 mg, 0.01 mmol, 2 mol%) and dry toluene (1.0 mL). The desired product *o*-tolylmethanamine salt, **2'n** was isolated as *o*-tolylmethanamine hydrochloride, **3n** (70.2 mg, 89% yield) as a colorless solid.

^{1}H NMR (500 MHz, DMSO-d$_6$): δ 8.64 (bs, 3H), 7.44 (d, J = 7.0 Hz, 1H), 7.27-7.21 (m, 3H), 3.97 (q, J = 5.5 Hz, 2H), 2.34 (s, 3H) ppm.

^{13}C{^{1}H} (100 MHz, DMSO-d$_6$): δ 136.7, 132.4, 130.3, 129.3, 128.4, 126.0, 39.4, 18.9 ppm.

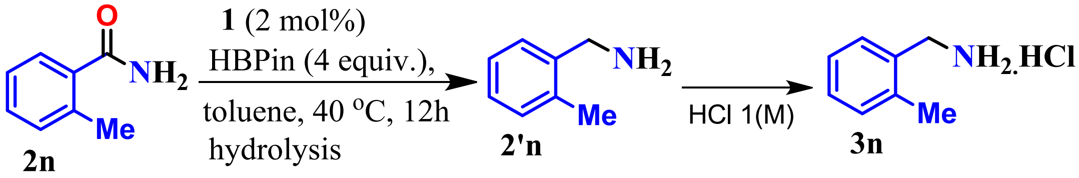

(2-methoxyphenyl)methanamine hydrochloride (3o).[S7]

The general procedure was followed for the synthesis of (2-methoxyphenyl)methanamine, **2'o**. The reaction was performed with 2-methoxybenzamide, **2o** (75.6 mg, 0.5 mmol), HBPin (290 µL, 2.0 mmol), **1** (14.8 mg, 0.01 mmol, 2 mol%) and dry toluene (1.0 mL). The desired product (2-methoxyphenyl)methanamine, **2'o** was isolated as (2-methoxyphenyl)methanamine hydrochloride salt, **3o** (43.4 mg, 50% yield) as a colorless solid.

^{1}H NMR (400 MHz, DMSO-d$_6$): δ 8.39 (bs, 3H), 7.41-7.35 (m, 2H), 7.05 (d, J = 8.4 Hz, 1H), 6.96 (t, J = 7.6 Hz, 1H), 3.94 (q, J = 5.6 Hz, 2H), 3.83 (s, 3H) ppm.

^{13}C{^{1}H} (100 MHz, DMSO-d$_6$): δ 157.2, 130.3, 130.2, 121.7, 120.3, 110.9, 55.5, 37.5 ppm.

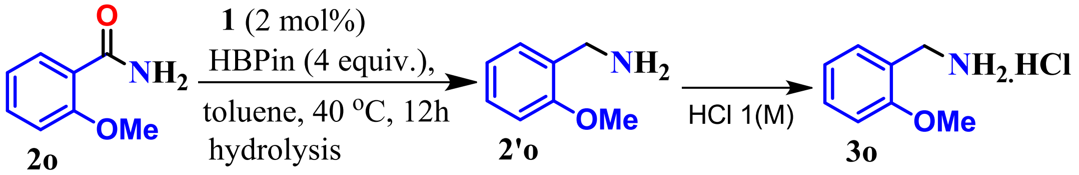

(2-ethoxyphenyl)methanamine hydrochloride (3p).

The general procedure was followed for the synthesis of (2-ethoxyphenyl)methanamine, **2'p**. The reaction was performed with 2-ethoxybenzamide, **2p** (82.6 mg, 0.5 mmol), HBPin (290 µL, 2.0 mmol), **1** (14.8 mg, 0.01 mmol, 2 mol%) and dry toluene (1.0 mL). The desired product (2-ethoxyphenyl)methanamine, **2'p** was isolated as (2-ethoxyphenyl)methanamine hydrochloride salt, **3p** (72.3 mg, 77% yield) as a colorless solid.

^{1}H NMR (400 MHz, DMSO-d$_6$): δ 8.09 (bs, 3H), 7.37-7.33 (m, 2H), 7.04 (d, J = 8.0 Hz, 1H), 6.95 (t, J = 7.6 Hz, 1H), 4.07 (q, J = 7.2 Hz, 2H), 3.95 (s, 2H), 1.36 (t, J = 7.2 Hz, 3H) ppm.

^{13}C{^{1}H} (100 MHz, DMSO-d$_6$): δ 156.7, 130.5, 130.4, 121.8, 120.4, 111.9, 63.8, 37.9, 14.7 ppm.

(HRMS): m/z (%) calcd for [C9H14ClNO]$^+$: 187.0764; found: 187.0762.

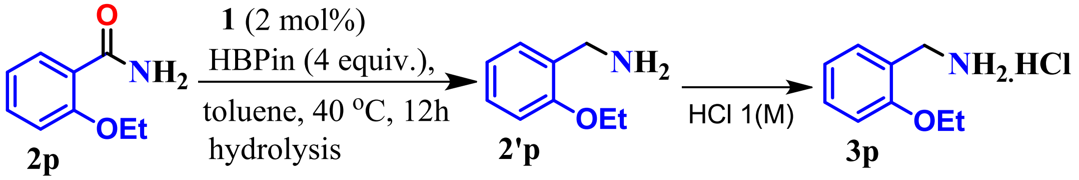

(2-fluorophenyl)methanamine hydrochloride (3q).[S9]

The general procedure was followed for the synthesis of (2-fluorophenyl)methanamine, **2'q**. The reaction was performed with 2-fluorobenzamide, **2q** (69.6 mg, 0.5 mmol), HBPin (290 µL, 2.0 mmol), **1** (14.8 mg, 0.01 mmol, 2 mol%) and dry toluene (1.0 mL). The desired product (2-fluorophenyl)methanamine, **2'q** was isolated as (2-fluorophenyl)methanamine hydrochloride salt, **3q** (70.3 mg, 87% yield) as a colorless solid.

^{1}H NMR (400 MHz, DMSO-d$_6$): δ 8.76 (bs, 3H), 7.66 (t, J = 7.6 Hz, 1H), 7.43 (q, J = 6.4 Hz, 1H),7.24 (t, J = 6.4 Hz, 2H), 4.03 (q, J = 4.8 Hz, 2H) ppm.

^{13}C{^{1}H} (100 MHz, DMSO-d$_6$): δ 160.3 (d, J = 245.1 Hz), 131.4, 130.8 (d, J = 8.2 Hz), 124.6, 121.1 (d, J = 14.6 Hz), 115.5 (d, J = 21.1 Hz), 35.4 ppm.

^{19}F (470 MHz, DMSO-d$_6$): δ -117.2 ppm.

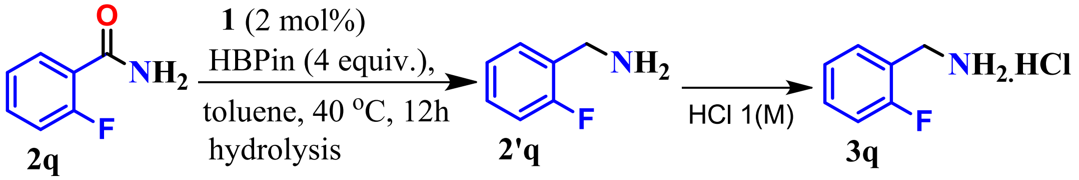

(2-chlorophenyl)methanamine hydrochloride (3r).[S2]

The general procedure was followed for the synthesis of (2-chlorophenyl)methanamine, **2'r**. The reaction was performed with 2-chlorobenzamide, **2r** (77.8 mg, 0.5 mmol), HBPin (290 µL, 2.0 mmol), **1** (14.8 mg, 0.01 mmol, 2 mol%) and dry toluene (1.0 mL). The desired product (2-chlorophenyl)methanamine, **2'r** was isolated as (2-chlorophenyl)methanamine hydrochloride salt, **3r** (74.8 mg, 84% yield) as a colorless solid.

^{1}H NMR (400 MHz, DMSO-d$_6$): δ 8.82 (bs, 3H), 7.71-7.67 (m, 1H), 7.54-7.49 (m, 1H), 7.43-7.38 (m, 2H), 4.10 (q, J = 5.2 Hz, 2H) ppm.

^{13}C{^{1}H} (100 MHz, DMSO-d$_6$): δ 132.8, 131.7, 130.7, 130.3, 129.4, 127.4, 39.3 ppm.

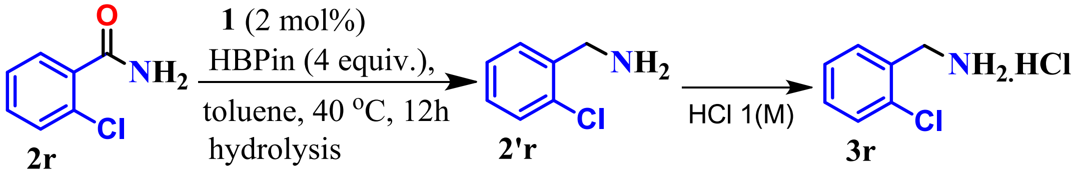

(2-bromophenyl)methanamine hydrochloride (3s).[S5]

The general procedure was followed for the synthesis of (2-bromophenyl)methanamine, **2's**. The reaction was performed with 2-bromobenzamide, **2s** (100.0 mg, 0.5 mmol), HBPin (290 μL, 2.0 mmol), **1** (14.8 mg, 0.01 mmol, 2 mol%) and dry toluene (1.0 mL). The desired product (2-bromophenyl)methanamine, **2's** was isolated as (2-bromophenyl)methanamine hydrochloride salt, **3s** (91.2 mg, 82% yield) as a colorless solid.

^{1}H NMR (400 MHz, DMSO-d$_6$): δ 8.90 (bs, 3H), 7.70-7.65 (m, J = 7.6 Hz, 2H), 7.44 (t, J = 7.2 Hz, 1H), 7.31 (t, J = 7.6 Hz, 1H), 4.08 (q, J = 6.8 Hz, 2H) ppm.

^{13}C{^{1}H} (100 MHz, DMSO-d$_6$): δ 133.8, 133.2, 131.0, 130.9, 128.5, 123.7, 42.4 ppm.

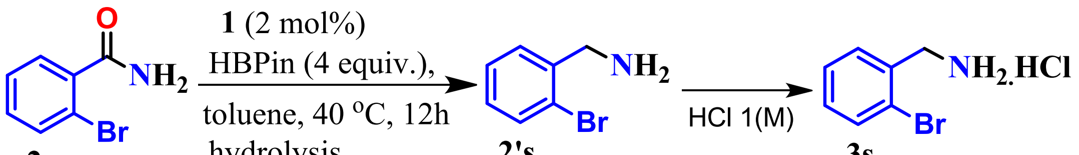

2s **2's** **3s**

(2,6-dimethoxyphenyl)methanamine hydrochloride (3t).[S2]

The general procedure was followed for the synthesis of (2,6-dimethoxyphenyl)methanamine, **2'x**. The reaction was performed with 2,6-dimethoxybenzamide, **2x** (90.6 mg, 0.5 mmol), HBPin (290 μL, 2.0 mmol), **1** (14.8 mg, 0.01 mmol, 2 mol%) and dry toluene (1.0 mL). The desired product (2,6-dimethoxyphenyl)methanamine, **2'x** was isolated as (2,6-dimethoxyphenyl)methanamine hydrochloride salt, **3x** (90.6 mg, 89% yield) as a colorless solid.

^{1}H NMR (400 MHz, DMSO-d$_6$): δ 8.56 (bs, 3H), 6.73 (d, J = 2.0 Hz, 2H), 6.47 (t, J = 2.0 Hz, 1H), 3.92 (q, J = 5.6 Hz, 2H), 3.74 (s, 6H) ppm.

^{13}C{^{1}H} (100 MHz, DMSO-d$_6$): δ 160.5, 136.3, 106.9, 99.9, 55.4, 42.2 ppm.

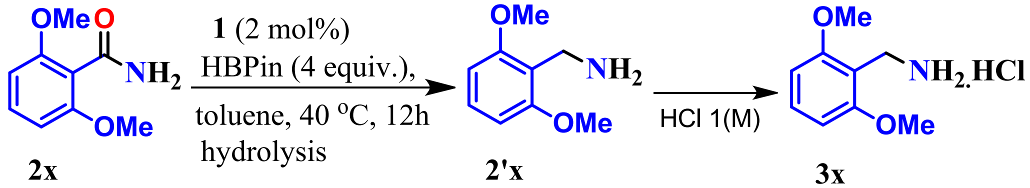

2x **2'x** **3x**

(2,6-difluorophenyl)methanamine hydrochloride (3t).[S10]

The general procedure was followed for the synthesis of (2,6-difluorophenyl)methanamine, **2't**. The reaction was performed with 2,6-difluorobenzamide, **2t** (78.6 mg, 0.5 mmol), HBPin (290 μL, 2.0 mmol), **1** (14.8 mg, 0.01 mmol, 2 mol%) and dry toluene (1.0 mL). The desired product (2,6-

difluorophenyl)methanamine, **2't** was isolated as (2,6-difluorophenyl)methanamine hydrochloride salt, **3t** (79.9 mg, 89% yield) as a colorless solid.

^{1}H NMR (400 MHz, DMSO-d$_6$): δ 8.56 (bs, 3H), 7.58-7.50 (m, 1H), 7.20 (t, J = 8.0 Hz, 2H), 4.04 (s, 2H) ppm.

^{13}C{^{1}H} (100 MHz, DMSO-d$_6$): δ 162.2 (d, $J_{C\text{-}F}$ = 7.5 Hz), 159.7 (d, $J_{C\text{-}F}$ = 7.3 Hz), 131.9 (t, $J_{C\text{-}F}$ = 10.4 Hz), 111.9 (d, $J_{C\text{-}F}$ = 5.5 Hz), 111.7 (d, $J_{C\text{-}F}$ = 5.4 Hz), 109.9 (t, $J_{C\text{-}F}$ = 19.2 Hz), 29.9 (t, $J_{C\text{-}F}$ = 3.7 Hz) ppm.

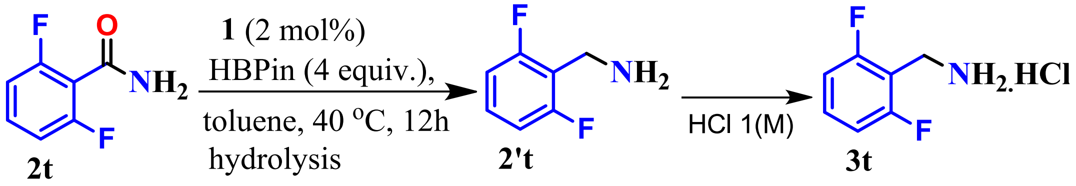

(3-chloro-2-methylphenyl)methanamine hydrochloride (3u).[S2]

The general procedure was followed for the synthesis of (3-chloro-2-methylphenyl)methanamine, **2'u**. The reaction was performed with 3-chloro-2-methylbenzamide, **2u** (84.8 mg, 0.5 mmol), HBPin (290 µL, 2.0 mmol), **1** (14.8 mg, 0.01 mmol, 2 mol%) and dry toluene (1.0 mL). The desired product (3-chloro-2-methylphenyl)methanamine, **2'u** was isolated as (3-chloro-2-methylphenyl)methanamine hydrochloride salt, **3u** (88.4 mg, 92% yield) as a colorless solid.

^{1}H NMR (400 MHz, DMSO-d$_6$): δ 8.68 (bs, 3H), 7.44 (d, J = 7.6 Hz, 2H), 7.26 (t, J = 7.6 Hz, 1H), 4.05 (q, J = 4.8 Hz, 2H), 2.38 (s, 3H) ppm.

^{13}C{^{1}H} (100 MHz, DMSO-d$_6$): δ 134.8, 134.6, 133.9, 129.2, 128.5, 127.2, 40.0, 15.8 ppm.

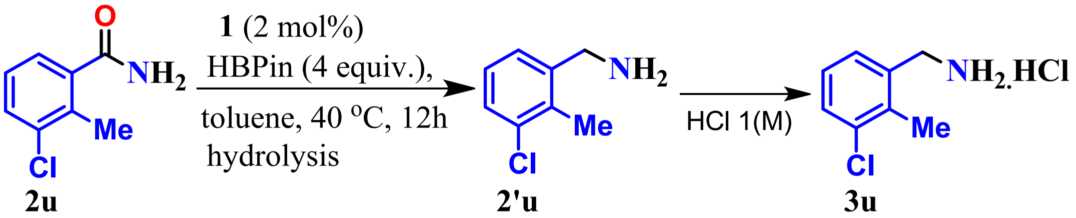

(5-fluoro-2-methylphenyl)methanamine hydrochloride (3v).[S2]

The general procedure was followed for the synthesis of (5-fluoro-2-methylphenyl)methanamine, **2'v**. The reaction was performed with 5-fluoro-2-methylbenzamide, **2v** (76.6 mg, 0.5 mmol), HBPin (290 µL, 2.0 mmol), **1** (14.8 mg, 0.01 mmol, 2 mol%) and dry toluene (1.0 mL). The desired product (5-fluoro-2-

methylphenyl)methanamine, **2'v** was isolated as (5-fluoro-2-methylphenyl)methanamine hydrochloride salt, **3v** (81.7 mg, 93% yield) as a colorless solid.

^{1}H NMR (500 MHz, DMSO-d$_6$): δ 8.67 (bs, 3H), 7.36-7.32 (m, 1H), 7.28-7.24 (m, 1H), 7.12-7.07 (m, 1H), 3.99 (q, J = 6.5 Hz, 2H), 2.30 (s, 3H) ppm.

^{13}C{^{1}H} (125 MHz, DMSO-d$_6$): δ 160.6 (d, J_{C-F} = 239.4 Hz), 134.6 (d, J_{C-F} = 7.5 Hz), 132.8 (d, J_{C-F} = 2.8 Hz), 132.2 (d, J_{C-F} = 7.7 Hz), 115.9 (d, J_{C-F} = 22.3 Hz), 115.1 (d, J_{C-F} = 20.4 Hz), 18.3 ppm.

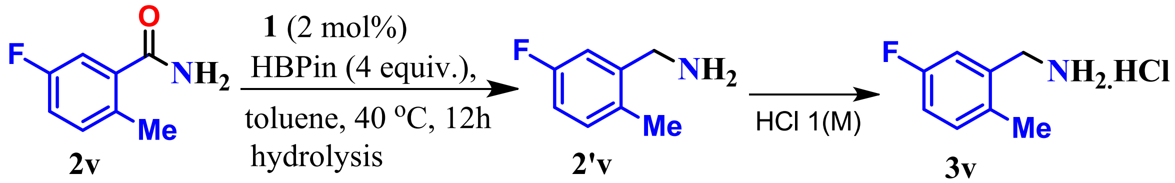

2v **2'v** **3v**

(3,5-dimethoxyphenyl)methanamine hydrochloride (3w).[S11]

The general procedure was followed for the synthesis of (3,5-dimethoxyphenyl)methanamine, **2'w**. The reaction was performed with 3,5-dimethoxybenzamide, **2w** (90.6 mg, 0.5 mmol), HBPin (290 µL, 2.0 mmol), **1** (14.8 mg, 0.01 mmol, 2 mol%) and dry toluene (1.0 mL). The desired product (3,5-dimethoxyphenyl)methanamine, **2'w** was isolated as (3,5-dimethoxyphenyl)methanamine hydrochloride salt, **3w** (91.6 mg, 90% yield) as a colorless solid.

^{1}H NMR (400 MHz, DMSO-d$_6$): δ 8.58 (bs, 3H), 6.74 (s, 2H), 6.47 (d, J = 2.0 Hz, 1H), 3.92 (s, 2H), 3.75 (s, 3H), 3.74 (s, 3H) ppm.

^{13}C{^{1}H} (100 MHz, DMSO-d$_6$): δ 160.5, 136.2, 106.9, 100.0, 55.4, 55.3, 42.2 ppm.

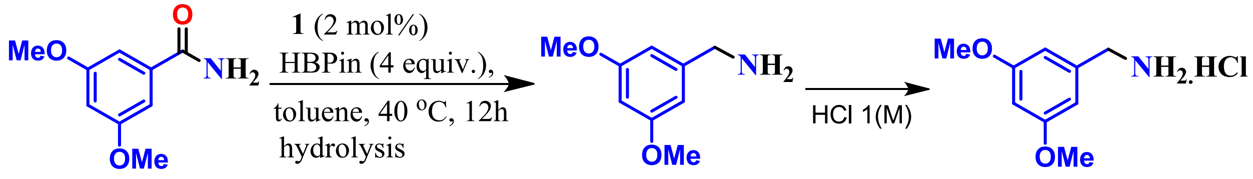

2w **2'w** **3w**

(3,4-dimethylphenyl)methanamine hydrochloride (3y).[S2]

The general procedure was followed for the synthesis of (3,4-dimethylphenyl)methanamine, **2'y**. The reaction was performed with 3,4-dimethylbenzamide, **2y** (74.6 mg, 0.5 mmol), HBPin (290 µL, 2.0 mmol), **1** (14.8 mg, 0.01 mmol, 2 mol%) and dry toluene (1.0 mL). The desired product (3,4-

dimethylphenyl)methanamine, **2'y** was isolated as (3,4-dimethylphenyl)methanamine hydrochloride salt, **3y** (78.1 mg, 91% yield) as a colorless solid.

^{1}H NMR (400 MHz, DMSO-d$_6$): δ 8.48 (bs, 3H), 7.26 (s, 1H), 7.22-7.19 (m, 1H), 7.15 (d, J = 7.6 Hz, 1H), 3.90 (q, J = 6.0 Hz, 2H), 2.21 (s, 6H) ppm.

^{13}C{^{1}H} (100 MHz, DMSO-d$_6$): δ 136.6, 136.5, 131.4, 130.2, 129.8, 126.5, 42.1, 19.5, 19.2 ppm.

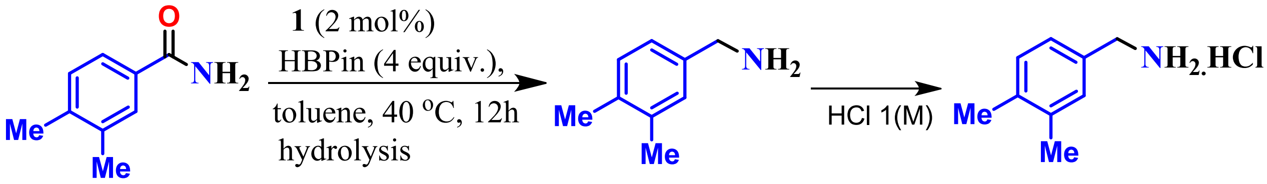

2y **2'y** **3y**

(4-bromo-3-methylphenyl)methanamine hydrochloride (3z).[S2]

The general procedure was followed for the synthesis of (4-bromo-3-methylphenyl)methanamine, **2'z**. The reaction was performed with 4-bromo-3-methylbenzamide, **2z** (107.0 mg, 0.5 mmol), HBPin (290 µL, 2.0 mmol), **1** (14.8 mg, 0.01 mmol, 2 mol%) and dry toluene (1.0 mL). The desired product (4-bromo-3-methylphenyl)methanamine, **2'z** was isolated as (4-bromo-3-methylphenyl)methanamine hydrochloride salt, **3z** (114.7 mg, 97% yield) as a colorless solid.

^{1}H NMR (400 MHz, DMSO-d$_6$): δ 8.73 (bs, 3H), 7.58 (d, J = 7.6 Hz, 1H),7.52 (s, 1H), 7.31-7.28 (m, 1H), 3.93 (q, J = 4.0 Hz, 2H), 2.32 (s, 3H) ppm.

^{13}C{^{1}H} (100 MHz, DMSO-d$_6$): δ 137.7, 134.3, 132.6, 132.3, 129.0, 124.5, 41.9, 22.9 ppm.

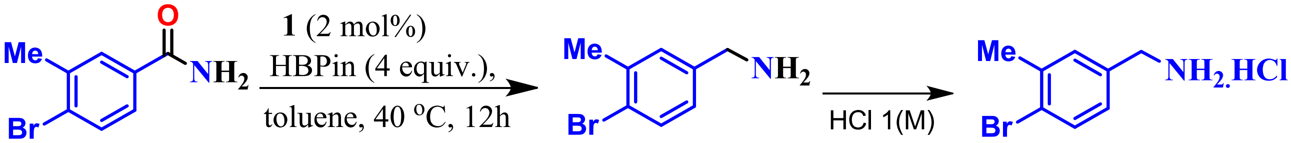

2z **2'z** **3z**

Thiophen-2-ylmethanamine hydrochloride (3aa).[S2]

The general procedure was followed for the synthesis of thiophen-2-ylmethanamine, **2'aa**. The reaction was performed with thiophene-2-carboxamide, **2aa** (63.6 mg, 0.5 mmol), HBPin (290 µL, 2.0 mmol), **1** (14.8 mg, 0.01 mmol, 2 mol%) and dry toluene (1.0 mL). The desired product thiophen-2-ylmethanamine, **2'aa** was isolated as thiophen-2-ylmethanamine hydrochloride salt **3aa** (55.4 mg, 74% yield) as a white solid.

^{1}H NMR (400 MHz, DMSO-d$_6$): δ 8.48 (bs, 3H), 7.56 (dd, J = 4.0, 1.6 Hz, 1H), 7.27 (d, J = 3.6 Hz, 1H), 7.06-7.04 (m, 1H), 4.20 (s, 2H) ppm.

^{13}C{^{1}H} (100 MHz, DMSO-d$_6$): δ 135.7, 129.7, 127.9, 127.8, 37.2 ppm.

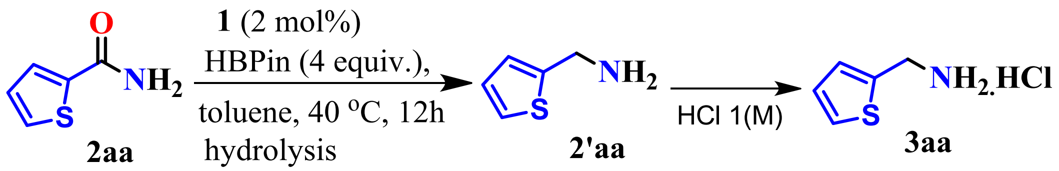

Benzothiophene-2-ylmethanamine hydrochloride (3ab).[S12]

The general procedure was followed for the synthesis of benzothiophene-2-ylmethanamine, **2'ab**. The reaction was performed with benzothiophene carboxamide, **2ab** (88.6 mg, 0.5 mmol), HBPin (290 µL, 2.0 mmol), **1** (14.8 mg, 0.01 mmol, 2 mol%) and dry toluene (1.0 mL). The desired product benzothiophene-2-ylmethanamine, **2'ab** was isolated as benzothiophene-2-ylmethanamine hydrochloride salt, **3ab** (81.9 mg, 82% yield) as a light yellow solid.

^{1}H NMR (400 MHz, DMSO-d$_6$): δ 8.83 (bs, 3H), 8.00-7.96 (m, 1H), 7.87-7.83 (m, 1H), 7.58 (s, 1H), 7.41-7.35 (m, 2H), 4.32 (s, 2H) ppm.

^{13}C{^{1}H} (100 MHz, DMSO-d$_6$): δ 139.6, 138.9, 136.7, 125.3, 124.8, 124.7, 123.8, 122.5, 37.5 ppm.

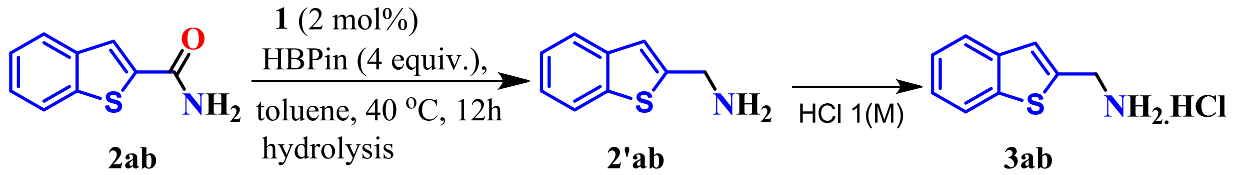

Naphthalen-1-ylmethanamine hydrochloride (3ad).[S2]

The general procedure was followed for the synthesis of naphthalen-1-ylmethanamine, **2'ad**. The reaction was performed with 1-naphthamide, **2ad** (79.6 mg, 0.5 mmol), HBPin (290 µL, 2.0 mmol), **1** (14.8 mg, 0.01 mmol, 2 mol%) and dry toluene (1.0 mL). The desired product naphthalen-1-ylmethanamine, **2'ad** was isolated as naphthalen-1-ylmethanamine hydrochloride salt, **3ad** (114.7 mg, 93% yield) as an off-white solid.

^{1}H NMR (400 MHz, DMSO-d$_6$): δ 8.69 (bs, 3H), 8.15 (d, J = 8.4 Hz, 1H), 8.01-7.96 (m,2H), 7.68-7.65 (m,1H), 7.64-7.53 (m,3H), 4.51 (q, J = 5.6 Hz, 2H) ppm.

$^{13}C\{^{1}H\}$ (100 MHz, DMSO-d_6): δ 133.2, 130.7, 129.9, 129.0, 128.7, 127.3, 126.8, 126.2, 125.4, 123.5, 39.1 ppm.

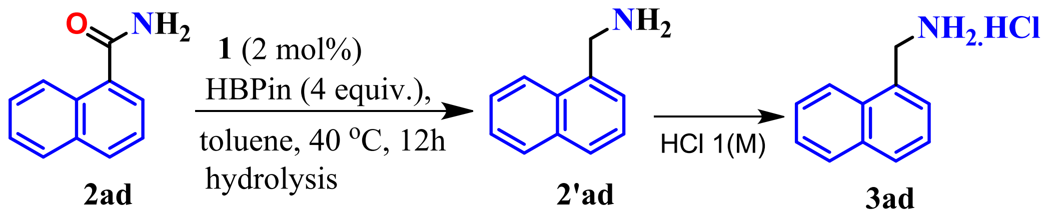

[1,1'-biphenyl]-4-ylmethanamine hydrochloride (3ae).[S3]

The general procedure was followed for the synthesis of [1,1'-biphenyl]-4-ylmethanamine, **2'ae**. The reaction was performed with [1,1'-biphenyl]-4-carboxamide, **2ae** (98.6 mg, 0.5 mmol), HBPin (290 µL, 2.0 mmol), **1** (14.8 mg, 0.01 mmol, 2 mol%) and dry toluene (1.0 mL). The desired product [1,1'-biphenyl]-4-ylmethanamine, **2'ae** was isolated as [1,1'-biphenyl]-4-ylmethanamine hydrochloride salt, **3ae** (99.9 mg, 91% yield) as a colorless solid.

^{1}H NMR (400 MHz, DMSO-d_6): δ 8.48 (bs, 3H), 7.69-7.65 (m, 4H), 7.58 (d, J = 8.4 Hz, 2H), 7.46 (t, J = 8.0 Hz, 2H), 7.36 (t, J = 7.6 Hz, 1H), 4.05 (s, 2H) ppm.

$^{13}C\{^{1}H\}$ (100 MHz, DMSO-d_6): δ 140.5, 139.7, 133.3, 129.9, 129.3, 127.9, 127.0, 126.9, 42.1 ppm.

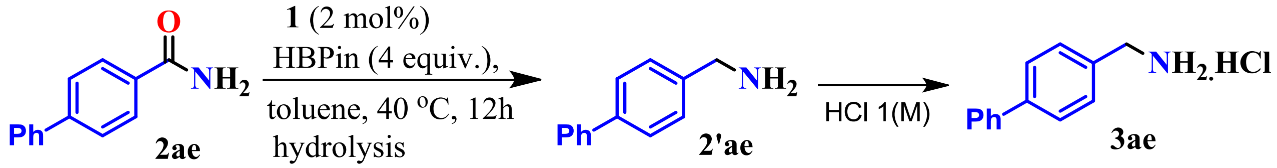

2-phenylethanamine hydrochloride (5a).[S3]

The general procedure was followed for the synthesis of 2-phenylethanamine, **4'a**. The reaction was performed with 2-phenylacetamide, **4a** (49.0 mg, 0.5 mmol), HBPin (290 µL, 2.0 mmol), **1** (14.8 mg, 0.01 mmol, 2 mol%) and dry toluene (1.0 mL). The desired product 2-phenylethanamine, **4'a** was isolated as 2-phenylethanamine hydrochloride salt, **5a** (67.6 mg, 75% yield) as a colorless solid.

^{1}H NMR (400 MHz, DMSO-d_6): δ 8.13 (bs, 3H), 7.33-7.29 (m, 2H), 7.25-7.21 (m, 3H), 2.99 (bs, 2H), 2.90-2.87 (m, 2H) ppm.

$^{13}C\{^{1}H\}$ (100 MHz, DMSO-d_6): δ 137.6, 128.9, 128.9, 126.9, 40.1, 33.1 ppm.

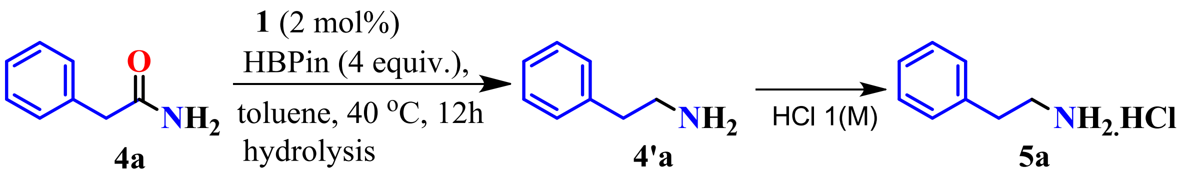

2-(thiophen-2-yl)ethanamine hydrochloride (5b).

The general procedure was followed for the synthesis of 2-(thiophen-2-yl)ethanamine, **4'b**. The reaction was performed with 2-(thiophen-2-yl)acetamide, **4b** (49.0 mg, 0.5 mmol), HBPin (290 μL, 2.0 mmol), **1** (14.8 mg, 0.01 mmol, 2 mol%) and dry toluene (1.0 mL). The desired product 2-(thiophen-2-yl)ethanamine, **4'b** was isolated as 2-(thiophen-2-yl)ethanamine hydrochloride salt, **5b** (49.1 mg, 60% yield) as a colorless solid.

^{1}H NMR (400 MHz, DMSO-d$_6$): δ 8.29 (bs, 3H), 7.39 (dd, J = 3.6, 1.6 Hz, 1H), 6.99-6.97 (m, 2H), 3.16-3.12 (m, 2H), 3.02 (bs, 2H) ppm.

^{13}C{^{1}H} (100 MHz, DMSO-d$_6$): δ 139.2, 127.3, 125.9, 124.7, 40.0, 27.1 ppm.

(HRMS): m/z (%) calcd for [$C_6H_{10}ClNS$]$^+$: 163.0222; found: 163.0219.

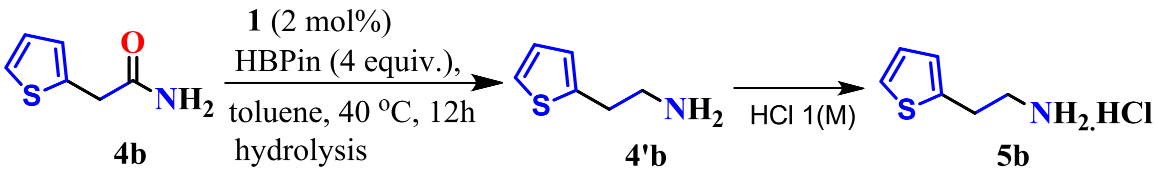

Cyclohexylmethanamine hydrochloride (5c).[S2-S3]

The general procedure was followed for the synthesis of cyclohexylmethanamine, **4'c**. The reaction was performed with cyclohexanecarboxamide, **4c** (56.6 mg, 0.5 mmol), HBPin (290 μL, 2.0 mmol), **1** (14.8 mg, 0.01 mmol, 2 mol%) and dry toluene (1.0 mL). The desired product cyclohexylmethanamine, **4'c** was isolated as cyclohexylmethanamine hydrochloride salt, **5c** (68.1 mg, 91% yield) as a colorless solid.

^{1}H NMR (400 MHz, DMSO-d$_6$): δ 8.16 (bs, 3H), 2.58 (t, J = 6.4 Hz, 2H), 1.75-1.52 (m, 6H), 1.22-1.04 (m, 3H), 0.93-0.84 (m, 2H) ppm.

^{13}C{^{1}H} (100 MHz, DMSO-d$_6$): δ 44.3, 35.3, 29.8, 25.7, 25.1 ppm.

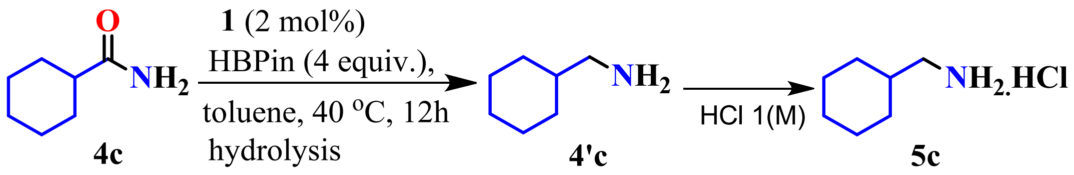

Butan-1-amine hydrochloride (5g).[S2]

The general procedure was followed for the synthesis of butan-1-amine, **4'g**. The reaction was performed with butyramide, **4g** (43.6 mg, 0.5 mmol), HBPin (290 µL, 2.0 mmol), **1** (14.8 mg, 0.01 mmol, 2 mol%) and dry toluene (1.0 mL). The desired product butan-1-amine, **4'g** was isolated as butan-1-amine hydrochloride salt, **5g** (47.7 mg, 87% yield) as a colorless solid.

^{1}H NMR (400 MHz, DMSO-d$_6$): δ 8.15 (bs, 3H), 2.71 (s, 2H), 1.53 (quint, J = 8.0 Hz, 2H), 1.35-1.26 (m, 2H), 0.85 (t, J = 7.6 Hz, 3H) ppm.

^{13}C{^{1}H} (100 MHz, DMSO-D6): δ 38.5, 29.0, 19.3, 13.6 ppm.

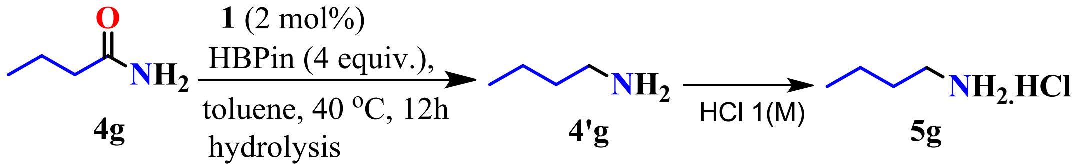

Cyclopropylmethanamine hydrochloride (5h).[S2]

The general procedure was followed for the synthesis of cyclopropylmethanamine, **4'h**. The reaction was performed with cyclopropanecarboxamide, **4h** (42.5 mg, 0.5 mmol), HBPin (290 µL, 2.0 mmol), **1** (14.8 mg, 0.01 mmol, 2 mol%) and dry toluene (1.0 mL). The desired product cyclopropylmethanamine, **4'h** was isolated as cyclopropylmethanamine hydrochloride salt, **5h** (39.8 mg, 74% yield) as a colorless solid.

^{1}H NMR (400 MHz, DMSO-d$_6$): δ 8.21 (bs, 3H), 2.65-2.59 (m, 2H), 1.08-0.98 (m, 1H), 0.51-0.47 (m, 2H), 0.33-0.29 (m, 2H) ppm.

^{13}C{^{1}H} (100 MHz, DMSO-d$_6$): δ 43.4, 8.5, 3.8 ppm.

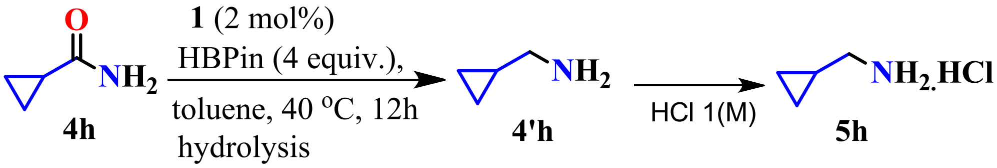

2-methylpropan-1-amine hydrochloride (5i).[S2-S3]

The general procedure was followed for the synthesis of 2-methylpropan-1-amine, **4'i**. The reaction was performed with isobutyramide, **4i** (43.6 mg, 0.5 mmol), HBPin (290 µL, 2.0 mmol), **1** (14.8 mg, 0.01 mmol, 2 mol%) and dry toluene (1.0 mL). The desired product 2-methylpropan-1-amine, **4'i** was isolated as 2-methylpropan-1-amine hydrochloride salt, **5i** (41.6 mg, 76% yield) as a colorless solid.

^{1}H NMR (400 MHz, DMSO-d$_6$): δ 8.09 (bs, 3H), 2.62-2.56 (m, 2H), 1.87 (sept, J = 6.8 Hz, 1H), 0.91 (d, J = 6.8 Hz, 6H) ppm.

^{13}C{^{1}H} (100 MHz, DMSO-D6): δ 45.7, 30.7, 26.4, 19.8 ppm.

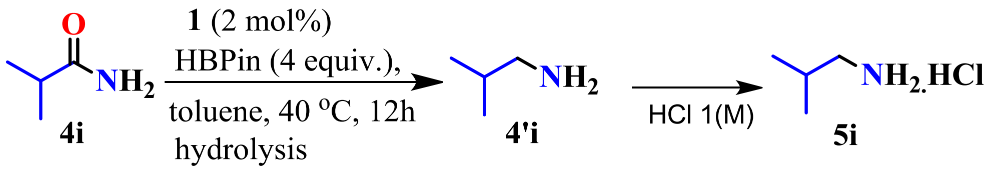

2,2-dimethylpropan-1-amine hydrochloride (5j).[S2-S3]

The general procedure was followed for the synthesis of 2,2-dimethylpropan-1-amine, **4'j**. The reaction was performed with pivalamide, **4j** (50.6 mg, 0.5 mmol), HBPin (290 µL, 2.0 mmol), **1** (14.8 mg, 0.01 mmol, 2 mol%) and dry toluene (1.0 mL). The desired product 2,2-dimethylpropan-1-amine, **4'j** was isolated as 2,2-dimethylpropan-1-amine hydrochloride salt, **5j** (56.9 mg, 92% yield) as a colorless solid.

^{1}H NMR (400 MHz, DMSO-d$_6$): δ 8.11 (bs, 3H), 2.58 (q, J = 6.0 Hz, 2H), 0.94 (s, 9H) ppm.

^{13}C{^{1}H} (100 MHz, DMSO-d$_6$): δ 49.7, 30.2, 26.9 ppm.

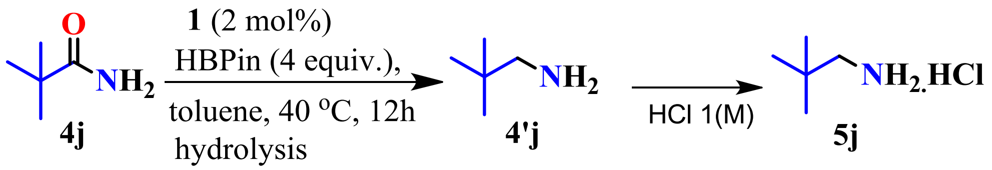

(9H-xanthen-9-yl)methanamine hydrochloride (5l).[S13]

The general procedure was followed for the synthesis of (9H-xanthen-9-yl)methanamine, **4'l**. The reaction was performed with 9H-xanthene-9-carboxamide, **4l** (112.6 mg, 0.5 mmol), HBPin (290 µL, 2.0 mmol), **1** (14.8 mg, 0.01 mmol, 2 mol%) and dry toluene (1.0 mL). The desired product (9H-xanthen-9-

yl)methanamine, **4'l** was isolated as (9H-xanthen-9-yl)methanamine hydrochloride salt, **5l** (113.9 mg, 92% yield) as a pale yellow color solid.

^{1}H NMR (400 MHz, DMSO-d$_6$): δ 8.09 (bs, 3H), 7.49 (dd, J = 8.0, 1.2 Hz, 2H), 7.35 (dt, J = 7.6, 1.2 Hz, 2H), 7.19-7.16 (m, 4H), 4.46 (t, J = 7.2 Hz, 1H), 2.97 (d, J = 6.8, 2H) ppm.

^{13}C{^{1}H} (100 MHz, DMSO-d$_6$): δ 133.6, 133.5, 131.3, 128.7,127.4, 126.9, 126.4, 125.8, 125.7, 123.6, 39.6, 30.2 ppm.

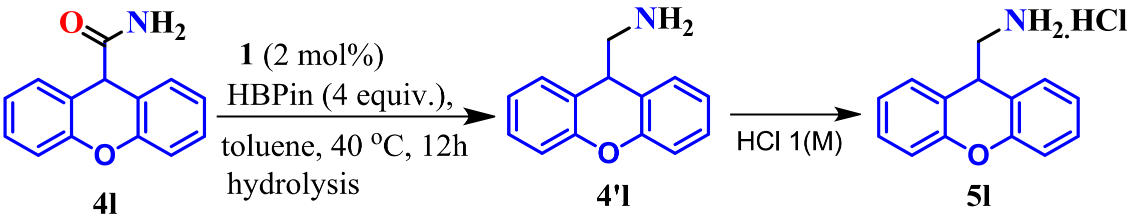

2-(naphthalen-1-yl)ethanamine hydrochloride (5m).[S10]

The general procedure was followed for the synthesis of 2-(naphthalen-1-yl)ethanamine, **4'm**. The reaction was performed with 2-(naphthalen-1-yl)acetamide, **4m** (92.6 mg, 0.5 mmol), HBPin (290 µL, 2.0 mmol), **1** (14.8 mg, 0.01 mmol, 2 mol%) and dry toluene (1.0 mL). The desired product 2-(naphthalen-1-yl)ethanamine, **4'm** was isolated as corresponding (9H-xanthen-9-yl)methanamine hydrochloride salt, **5m** (95.5 mg, 92% yield) as a colorless solid.

^{1}H NMR (400 MHz, DMSO-d$_6$): δ 8.46 (bs, 3H), 8.21 (d, J = 8.0Hz, 1H), 7.93 (dd, J = 8.4, 1.2 Hz, 1H), 7.83 (dd, J = 4.8, 2.4 Hz, 1H),7.59-7.55 (m, 2H), 7.46-7.43 (m, 2H), 3.46-3.42 (m, 2H), 3.12-3.04 (m, 2H) ppm.

^{13}C{^{1}H} NMR (100 MHz, DMSO-d$_6$): δ 151.9, 129.5, 129.1, 124.1, 121.4, 116.7, 45.8, 36.4 ppm.

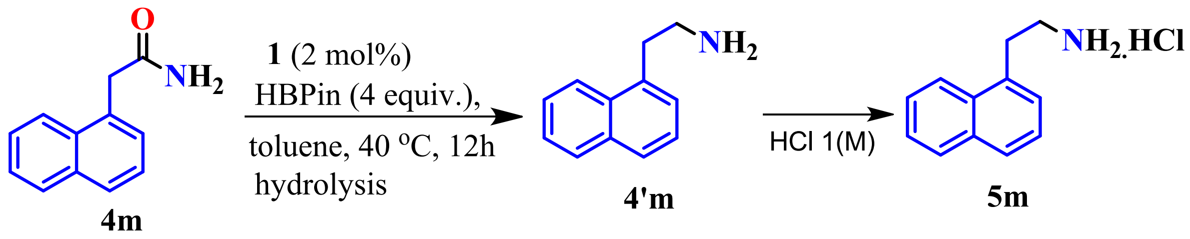

N-methyl-1-phenylmethanamine hydrochloride (5n).[S14]

The general procedure was followed for the synthesis of N-methyl-1-phenylmethanamine, **4'n**. The reaction was performed with N-benzylformamide, **4n** (67.6 mg, 0.5 mmol), HBPin (290 µL, 2.0 mmol), **1**

(14.8 mg, 0.01 mmol, 2 mol%) and dry toluene (1.0 mL). The desired product N-methyl-1-phenylmethanamine, **4'n** was isolated as N-methyl-1-phenylmethanamine hydrochloride salt, **5n** (61.5 mg, 78% yield) as white solid.

^{1}H NMR (400 MHz, DMSO-d$_6$): δ 9.67 (bs, 2H), 7.62-7.59 (m, 2H), 7.39-7.37 (m, 3H), 4.60 (s, 2H), 4.12 (t, J = 6.0Hz, 3H) ppm.

^{13}C{^{1}H} NMR (100 MHz, DMSO-d$_6$): δ 132.1, 130.2, 128.9, 128.7, 51.2, 32.0 ppm.

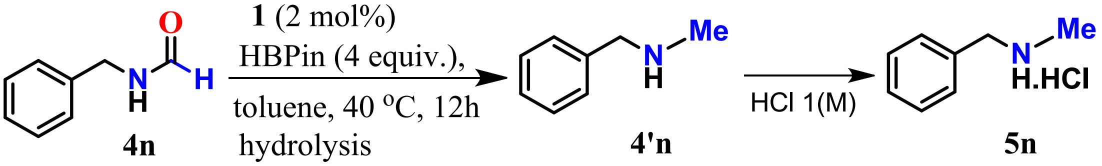

(R)-2-(6-methoxynaphthalen-2-yl)propan-1-amine hydrochloride (5o).

The general procedure was followed for the synthesis of (R)-2-(6-methoxynaphthalen-2-yl)propan-1-amine, **4'o**. The reaction was performed with (R)-2-(6-methoxynaphthalen-2-yl)propanamide, **4o** (114.6 mg, 0.5 mmol), HBPin (290 µL, 2.0 mmol), **1** (14.8 mg, 0.01 mmol, 2 mol%) and dry toluene (1.0 mL). The desired product (R)-2-(6-methoxynaphthalen-2-yl)propan-1-amine, **4'o** was isolated as (R)-2-(6-methoxynaphthalen-2-yl)propan-1-amine hydrochloride salt, **5o** (99.4 mg, 79% yield) as a colorless solid.

^{1}H NMR (400 MHz, DMSO-d$_6$): δ 8.17 (bs, 3H), 7.79 (dd, J = 6.4, 2.8 Hz, 2H), 7.71 (s, 1H), 7.42 (dd, J = 8.4, 1.6 Hz, 1H), 7.30 (d, J = 2.4 Hz, 1H), 7.15 (dd, J = 8.8, 2.8 Hz, 1H), 3.85 (s, 1H), 3.27-3.18 (m, 1H), 3.06-3.04 (m, 2H), 1.33 (d, J = 6.8 Hz, 3H) ppm.

^{13}C{^{1}H} (100 MHz, DMSO-d$_6$): δ 157.1, 137.9, 133.4, 129.1, 128.6, 127.2, 126.1, 125.5, 118.7, 105.8, 55.2, 44.9, 37.3, 19.4 ppm.

HRMS: m/z calc. for $C_{14}H_{19}ClNO$ [M+H]$^+$; 252.1155, found 252.1151.

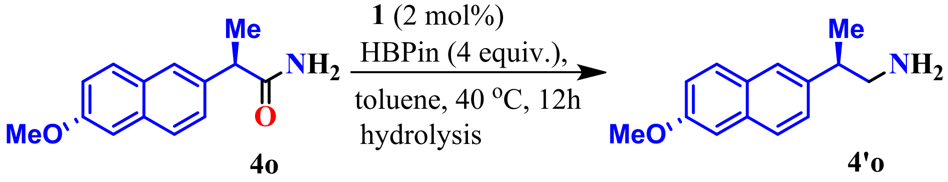

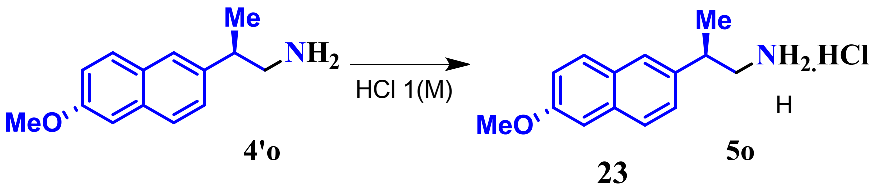

4. Application of current methodology in gram scale preparation.

2-phenylethanamine (4'a).

An oven dried 100 mL Schlenk flask was charged with [*a*NHC.KN(SiMe$_3$)$_2$]$_2$, **1** (385.0 mg, 0.26 mmol, 2 mol%) and HBPin (7.54 mL, 52.0 mmol, 4 equivalent) along with 20.0 mL toluene inside a N_2 filled glovebox. Then 2-phenylacetamide, **4a** (1.76 G, 13.0 mmol) was added to the reaction mixture and stirred for 12h at 40 °C. After completion of the reaction, 10.0 mL 2.0 (M) NaOH solution was added to the reaction mixture along with 20.0 mL Et_2O and stirred for another 1h. Next, the reaction mixture was worked up with Et_2O:H_2O mixture (1:1) and the reduced 2-phenylethanamine, **4'a** (1.15 mL) was concentrated in vacuum. Consequently, 10.0 mL 1.0 (M) HCl was added to the concentrated amines followed by addition of 20.0 mL Et_2O and the corresponding 2-phenylethanamine hydrochloride salt, **5a** (1.50 G) was purified by washing with Et_2O. Isolated **5a** was characterized through NMR spectroscopy in DMSO-d$_6$.

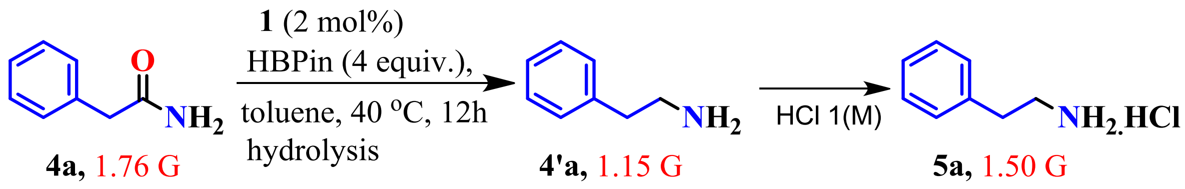

Scheme S2. Gram scale preparation of 2-phenylethanamine **(4'a).**

N-methyl-1-phenylmethanamine (4'n).[S5]

An oven dried 100 mL Schlenk flask was charged with [*a*NHC.KN(SiMe$_3$)$_2$]$_2$, **1** (1.13 G, 0.76 mmol, 2 mol%) and HBPin (22.1 mL, 152.0 mmol, 4 equivalent) along with 35.0 mL toluene inside a N_2 filled glovebox. Subsequently, N-benzylformamide, **4n** (5.14 G, 38.0 mmol) was added to the reaction mixture and stirred for 12h at 40 °C. After completion of the reaction, 35.0 mL 2.0 (M) NaOH solution was added to the reaction mixture along with 35.0 mL Et_2O and stirred for another 1h. Next, the reaction mixture was worked up with Et_2O:H_2O mixture (1:1) and the reduced N-methyl-1-phenylmethanamine, **4'n** (3.5 G) was concentrated in vacuum and characterized through NMR spectroscopy in $CDCl_3$.

^{1}H NMR (400 MHz, $CDCl_3$): δ 7.35-7.30 (m, 4H), 7.27-7.22 (m, 1H), 3.74 (s, 2H), 2.45 (s, 3H) ppm.

^{13}C{^{1}H} NMR (100 MHz, $CDCl_3$): δ 140.1, 128.3, 128.0, 126.8, 56.0, 35.9 ppm.

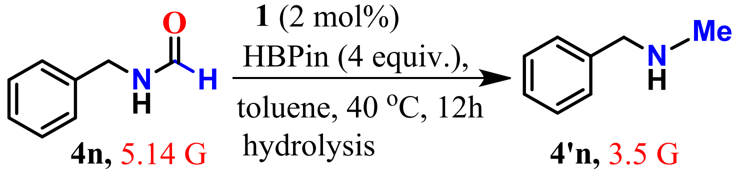

Scheme S3. Gram scale preparation of N-methyl-1-phenylmethanamine **(4'n).**

5. Control experiments for mechanistic investigation.

To proof the mechanistic course for the reduction of benzamides, we performed several stoichiometric reactions.

5a. Investigation into the radical or non-radical nature of 1 catalyzed benzamide reduction.

To evaluate whether the reduction of benzamide proceeds through a radical pathway or not, we performed the reaction in presence of a radical scavenger (TEMPO). An oven dried 20 mL reaction tube was charged with [*a*NHC.KN(SiMe$_3$)$_2$]$_2$, **1** (14.8 mg, 2 mol%) and pinacolborane (290 µL, 2.0 mmol, 4 equivalent), and TEMPO (0.5 mmol) along with 1 mL solvent inside the N_2 filled glovebox. Subsequently benzamide (60.6 mg, 0.5 mmol) was added to the reaction mixture and stirred for 12h at 40 ^{o}C. After completion of the reaction, 1.0 mL 2.0 (M) NaOH solution was added to the reaction mixture along with 1.0 mL Et_2O and stirred for another 1h. Next, the reaction mixture was worked up with Et_2O:H_2O mixture (1:1) and the corresponding benzylamine was concentrated in vacuum. Subsequently, 1.0 mL 1.0 (M) HCl was added to the concentrated amines followed by addition of 1.0 mL Et_2O and the corresponding benzylamine hydrochloride salt was purified by washing with Et_2O and characterized through ^{1}H NMR spectroscopy in DMSO-d$_6$. The quantitative yield of the benzylamine hydrochloride clearly suggests that this reduction reaction proceeds through a non-radical pathway.

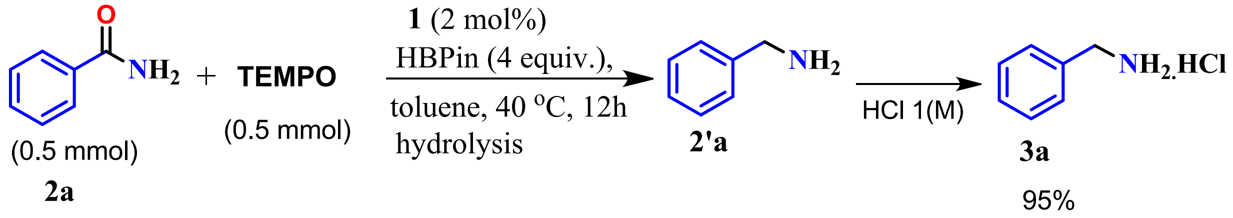

Scheme S4. Reduction of benzamide in presence of a radical scavenger, TEMPO.

5b. Detection of molecular hydrogen in non-catalytic and catalytic hydroboration of benzamide.

An oven dried screw-cap NMR tube was charged with benzamide, **2a** (0.1 mmol), pinacolborane (29 µL, 0.2 mmol, 2 equivalent) and benzene-d$_6$ (600 µL) in non-catalytic reaction and immediate evolution of molecular hydrogen was observed which was characterized through ^{1}H NMR spectroscopy.

Similarly, [*a*NHC.KN(SiMe$_3$)$_2$]$_2$, **1** (2 mol%), pinacolborane (58 µL, 0.4 mmol, 4 equivalent) and benzamide (0.1 mmol) were loaded along with benzene-d$_6$ (600 µL) in catalytic reaction and kept in pre-

heated (40 °C) oil bath for 1h. An evolution of molecular hydrogen was observed which was characterized again through ^{1}H NMR spectroscopy in C_6D_6.

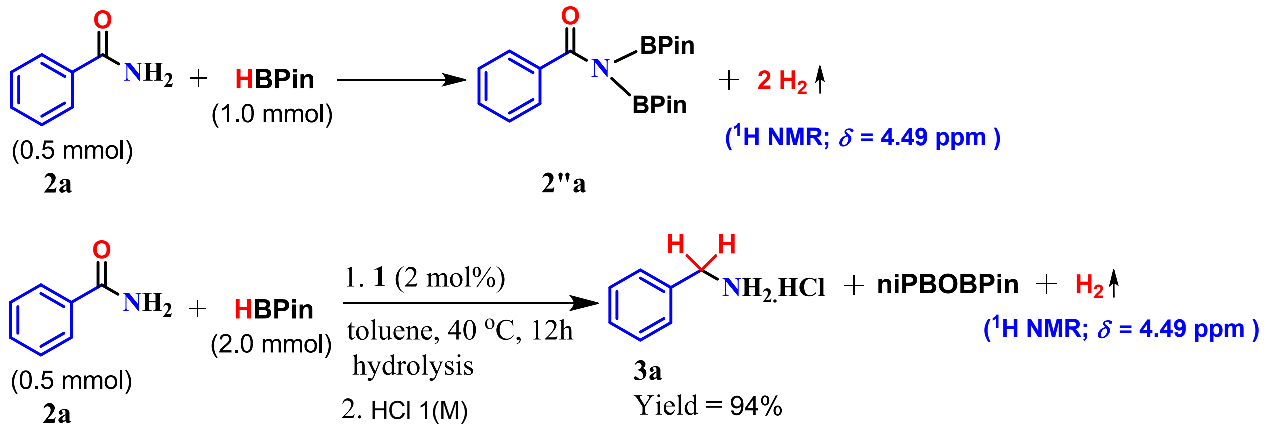

Scheme S5. Detection of molecular hydrogen in non-catalytic and catalytic hydroboration of benzamide.

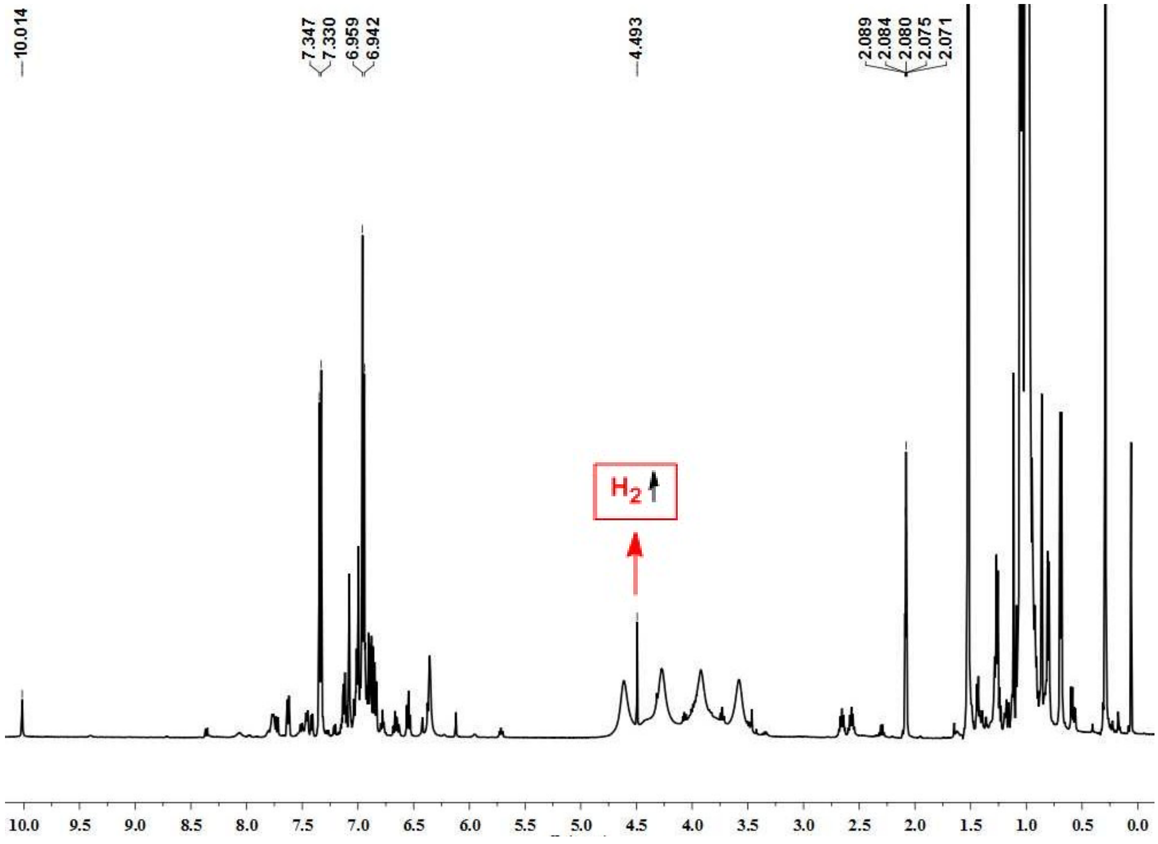

Figure S1. ^{1}H NMR spectrum of molecular hydrogen recorded in toluene-d_8 while performing non-catalytic hydroboration of benzamide.

5c. Preparation and characterization of *a*NHC-HBPin adduct.

An oven dried 5 mL borosil vial was charged with [*a*NHC.KN($SiMe_3$)$_2$]$_2$, **1** (0.2 mmol), pinacolborane (64 µL, 0.44 mmol, 2.2 equivalent) and toluene (700 µL) in a nitrogen filled glovebox. The green color of the reaction mixture was changed to colorless within few minutes and the reaction mixture was stirred for 12 h at room temperature. Subsequently, the reaction mixture was kept for crystallization at -35 ^{o}C. Colorless crystals were grown from toluene at -35 ^{o}C within 5 days. Compound **1a** was characterized through SCXRD, as well as ^{1}H, ^{13}C, and ^{11}B NMR spectroscopies. After isolation of crystals, ^{1}H NMR spectrum of remaining solution of the reaction mixture was subjected to 1H NMR spectroscopy after evaporation of solvents and re-dissolving in toluene-d_8 when a singlet at δ 0.06 ppm clearly suggests the presence of KN($SiMe_3$)$_2$.

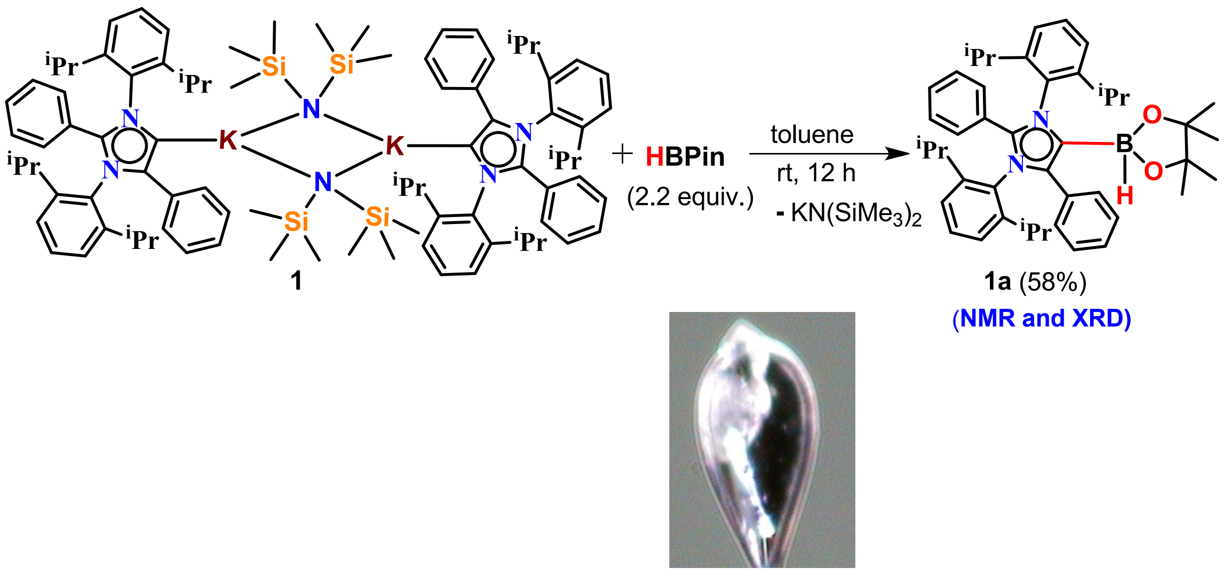

Scheme S6. Synthesis of *a*NHC-HBPin adduct and image of crystal (**1a**).

^{1}H NMR (400 MHz, C_6D_6): δ 7.91 (d, J = 7.6 Hz, 2H), 7.25-7.21 (m, 2H), 7.15-7.11 (m, 3H), 7.04 (t, J = 7.6 Hz, 2H), 6.96 (dd, J = 8.4, 1.6 Hz, 2H), 6.84 (d, J = 7.6 Hz, 2H), 6.62-6.54 (m, 3H), 3.29 (sept, J = 6.8 Hz, 2H), 2.84 (sept, J = 6.8 Hz, 2H), 2.16 (s, 1H), 1.75 (d, J = 6.8 Hz, 6H), 1.50 (s, 6H), 1.12 (s, 6H), 0.95 (d, J = 7.2 Hz, 6H), 0.81 (d, J = 6.8 Hz, 6H), 0.76 (d, J = 6.8 Hz, 6H) ppm.

^{13}C{^{1}H} NMR (100 MHz, C_6D_6): δ 145.7, 145.6, 141.8, 137.5, 134.7, 133.9, 131.7, 131.1, 130.6, 130.1, 130.0, 129.3, 129.2, 128.6, 126.8, 125.7, 125.3, 124.6, 124.2, 78.0, 29.3. 28.7, 26.3, 25.9, 24.9, 24.1, 23.7, 23.5 ppm.

^{11}B{^{1}H} NMR (128 MHz, C_6D_6): δ 1.47 ppm.

Elemental analysis: Anal. Calcd for $C_{45}H_{57}BN_2O_2$: C, 80.82; H, 8.59; N, 4.19. Found: C, 80.84; H, 8.56; N, 4.16.

HRMS: m/z calc. for $C_{45}H_{58}N_2O_2B$ $[M+H]^+$ 669.4585, found 669.4581.

X-ray crystallographic details.

Single crystals of compound **1a** were mounted on a glass pip. Intensity data were collected on a SuperNova, Dual, Mo at zero, Eos diffractometer. The crystals were kept at 100K during data collection. Atomic coordinates, isotropic and anisotropic displacement parameters of all the non-hydrogen atoms of two compounds were refined using Olex2,[S15] and the structure was solved with the Superflip[S16] structure solution program using Charge Flipping and refined with the ShelXL[S17] refinement package using Least Squares minimization. Structure graphics shown in the figures were created using the Olex2 and X-Seed software package version 2.0.[S18]

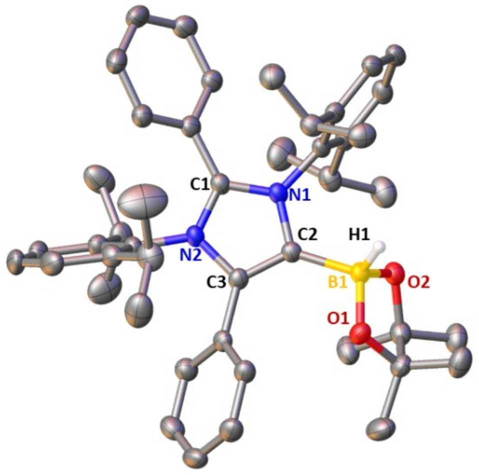

Figure S2. View of the molecular structure of **1a**. Ellipsoids are set at 50% probability level; hydrogen atoms of **1a** have been omitted for the sake of clarity.

Table S2. Crystallographic and structure refinement data for compound **1a**.

Complex	**1a**
CCDC No	1900619
Formula	$C_{45} H_{57} B N_2 O_2$
Fw	668.73
Crystal System	Triclinic
Space group	P-1
a [Å]	9.6381(5)
b [Å]	11.4850(6)
c [Å]	19.0369(9)
α [^{0}]	85.238(4)
β [^{0}]	75.946(4)
γ [^{0}]	75.838(5)
V [Å ^{3}]	1981.44(18)
Z	2
λ [Å]	1.54184
ρ_{calcd} [gcm^{-3}]	1.121
F[000]	724.0
μ [mm^{-1}]	0.513
θ [^{0}]	4.786- 131.826
index ranges	−10≤ h ≤ 11<br>−13≤ k ≤13<br>−22≤ l ≤22
T [K]	100
R1	0.0484

*wR*2	0.1208
R_{merge}	0.0626
Parameters	463
GOF	1.019
reflns total	16871
unique reflns	6844
Obsdreflns	5448

Table S3. Selected bond distances (Å) and angles (°) observed in **1a**

Bond	Distance	Bond	Angles
N(1)-C(1)	1.351(2)	C(1)-N(1)-C(2)	111.83(13)
N(2)-C(1)	1.346(2)	C(1)-N(2)-C(3)	109.46(13)
N(1)-C(2)	1.399(2)	N(2)-C(1)-N(1)	106.18(14)
N(2)-C(3)	1.406(2)	N(1)-C(2)-B(1)	120.65(13)
C(2)-C(3)	1.371(2)	C(3)-C(2)-N(1)	104.55(13)
C(2)-B(1)	1.668(2)	C(3)-C(2)-B(1)	134.54(14)
B(1)-O(1)	1.469(2)	O(1)-B(1)-C(2)	108.73(13)
B(1)-O(2)	1.468(2)	O(2)-B(1)-O(1)	106.02(13)
		O(2)-B(1)-C(2)	113.02(13)

NMR characterization of *a*NHC-HBPin adduct (1a).

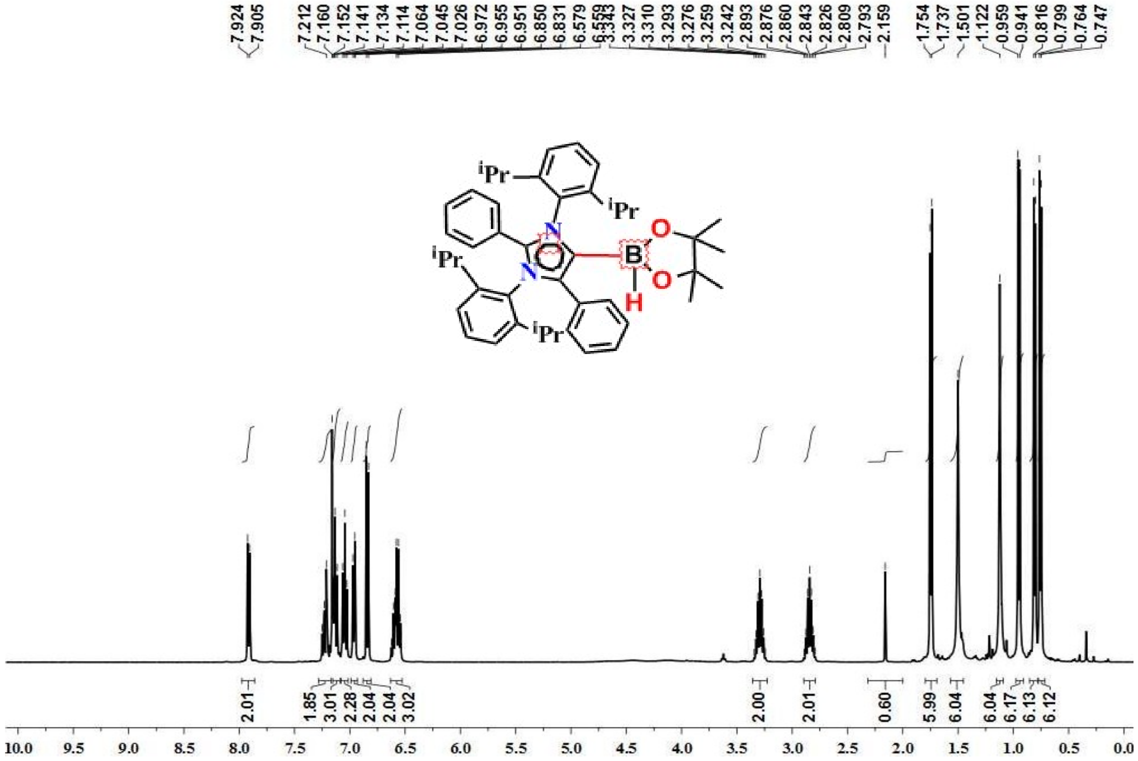

Figure S3. ^{1}H NMR spectrum of *a*NHC-HBPin adduct (**1a**) recorded in C_6D_6.

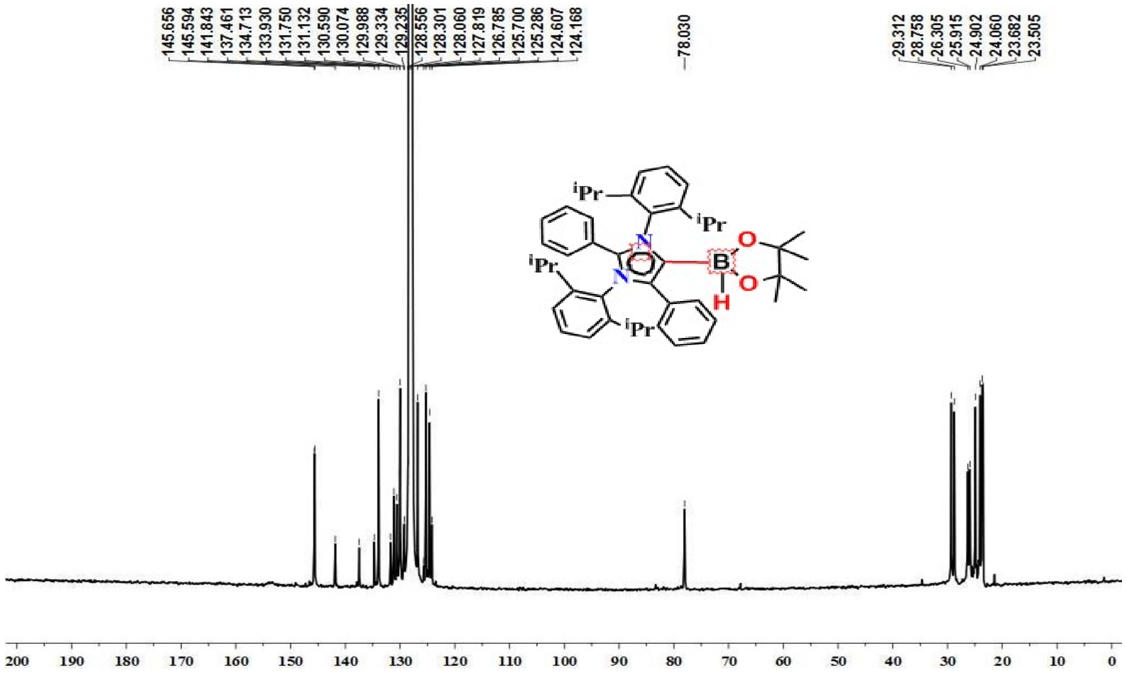

Figure S4. $^{13}C\{^{1}H\}$ NMR spectrum of *a*NHC-HBPin adduct (**1a**) recorded in C_6D_6.

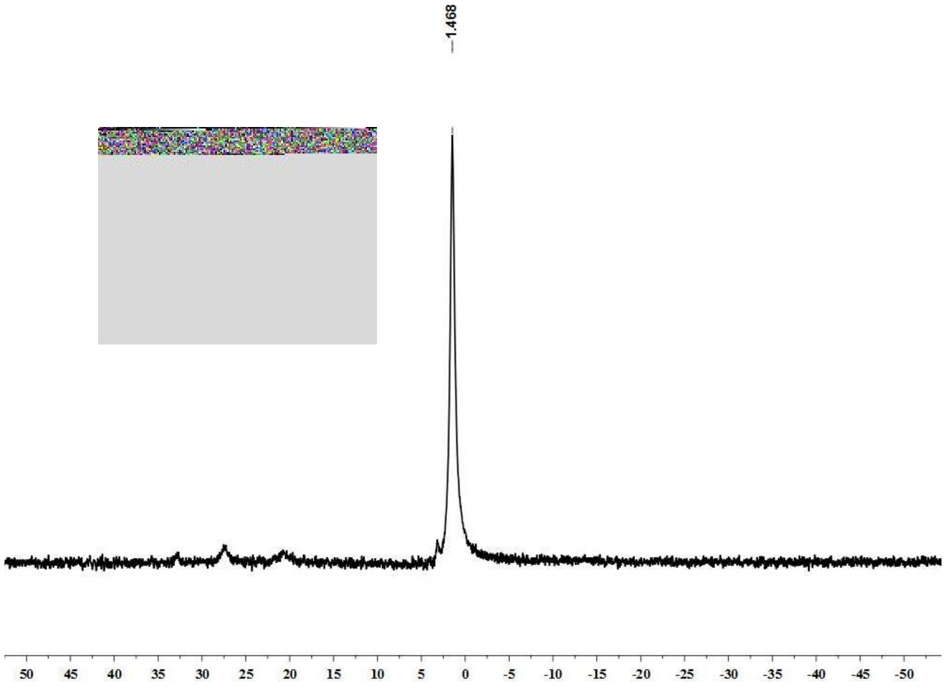

Figure S5. $^{11}B\{^1H\}$ NMR spectrum of *a*NHC-HBPin adduct (**1a**) recorded in C_6D_6.

5d. Activation of amide through interaction of borylated-amide (2a') and $KN(SiMe_3)_2$.

Table S4. Reduction of 4-nitrobenzamide in presence of catalytic *a*NHC and different $MN(SiMe_3)_2$ (M = K, and Na).[a]

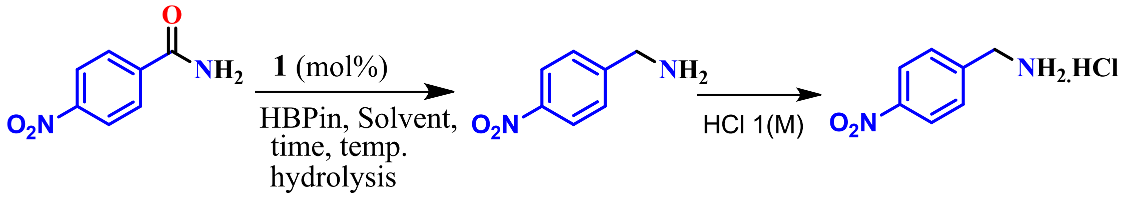

Entry	Catalyst 1 (mol%)	HBpin (equiv)	Solvent	Temp. (° C)	Time (h)	Yield (%)[b]
1	**1** (2 mol%)	**4**	**Toluene**	**40**	**12**	**83**
2	[*a*NHC + KHMDS] (2 mol%)	4	Toluene	40	12	78
3	[*a*NHC + NaHMDS] (2 mol%)	4	Toluene	40	12	56

[a]Reaction conditions: Catalyst (2.0 mol %), HBPin (2.0 mmol, 4.0 equiv.), 4-nitro benzamide (0.5 mmol), toluene (1.0 mL), 40 °C, 12 h. Hydrolysis was performed with 2.0 (M) NaOH solution. [b]All yields are isolated yields.

Preparation and characterization of borylated-amide (2a').

An oven dried screw cap NMR tube was charged with benzamide (24.2 mg, 0.2 mmol), HBPin (58 µL, 0.4 mmol, 2.0 equivalent) and THF-d_8 (600 µL) in a nitrogen filled glovebox. Subsequently, the reaction mixture was kept at room temperature for 14 h and during the reaction, evolution of hydrogen gas was monitored through ^{1}H NMR spectroscopy. After completion of the reaction, borylated-amide (**2a'**) was characterized through ^{1}H, ^{13}C, and ^{11}B NMR spectroscopies.

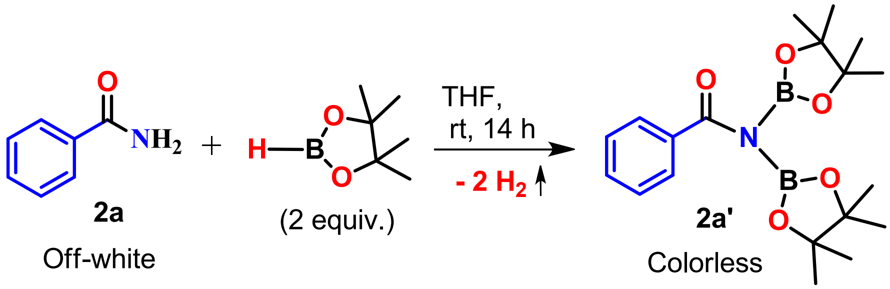

Scheme S7. Preparation of borylated-amide (**2a'**).

^{1}H NMR (400 MHz, THF-d_8): δ 7.93-7.88 (m, 2H), 7.48-7.35 (m, 3H), 1.22-1.19 (m, 24H) ppm.

^{13}C{^{1}H} NMR (100 MHz, C_6D_6): δ 169.2, 136.3, 131.9, 128.6, 128.6, 83.6, 83.4, 82.2, 79.6 ppm.

^{11}B{^{1}H} NMR (128 MHz, C_6D_6): δ 22.5, 19.5 ppm.

HRMS: m/z calc. for $C_{19}H_{34}B_2N_2O_5$ $[M+H+NH_4]^+$ 392.2648, found 392.2577.

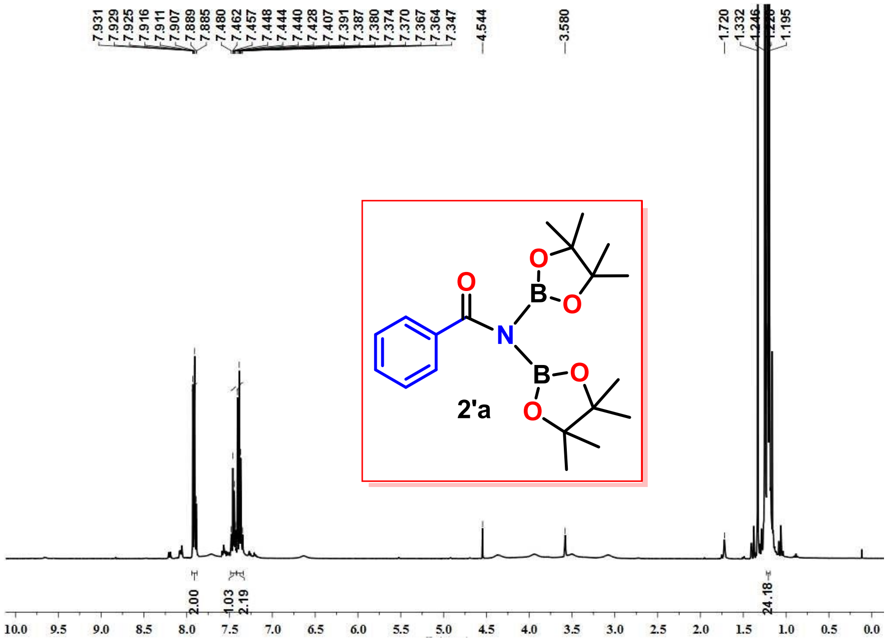

Figure S6. ^{1}H NMR spectrum of borylated-amide (**2a'**) recorded in THF-d_8.

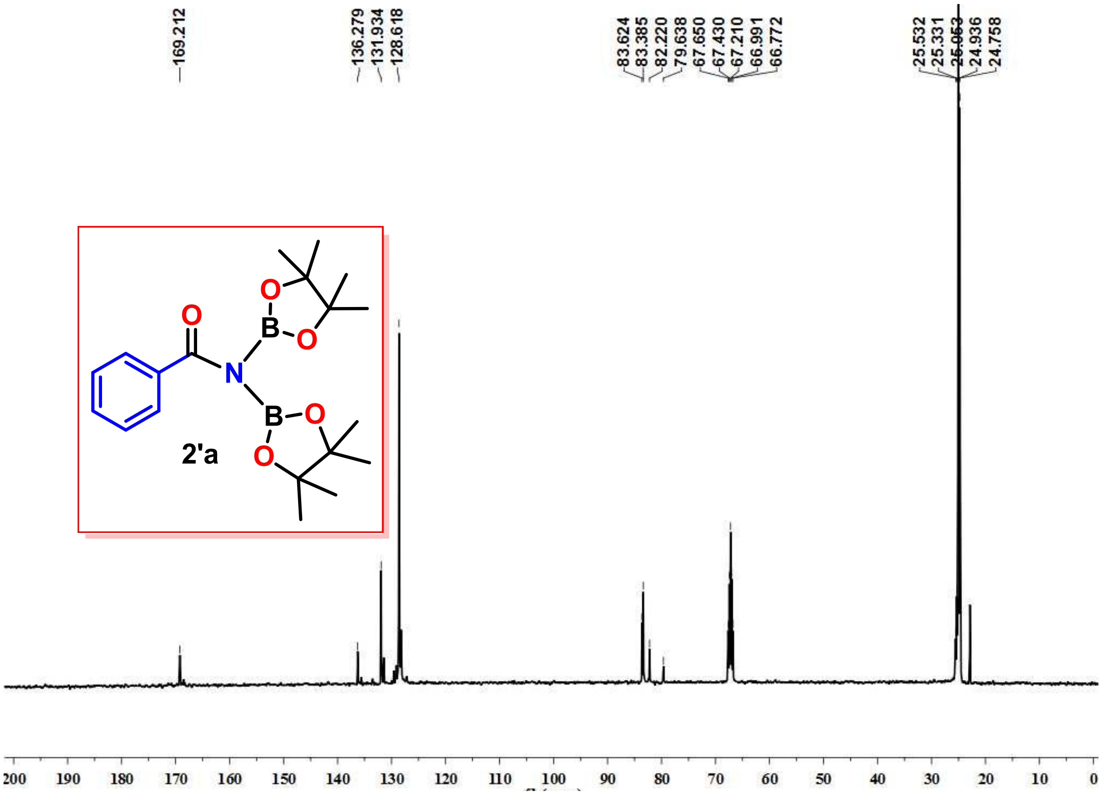

Figure S7. $^{13}C\{^{1}H\}$ NMR spectrum of borylated-amide (**2a'**) recorded in THF-d$_8$.

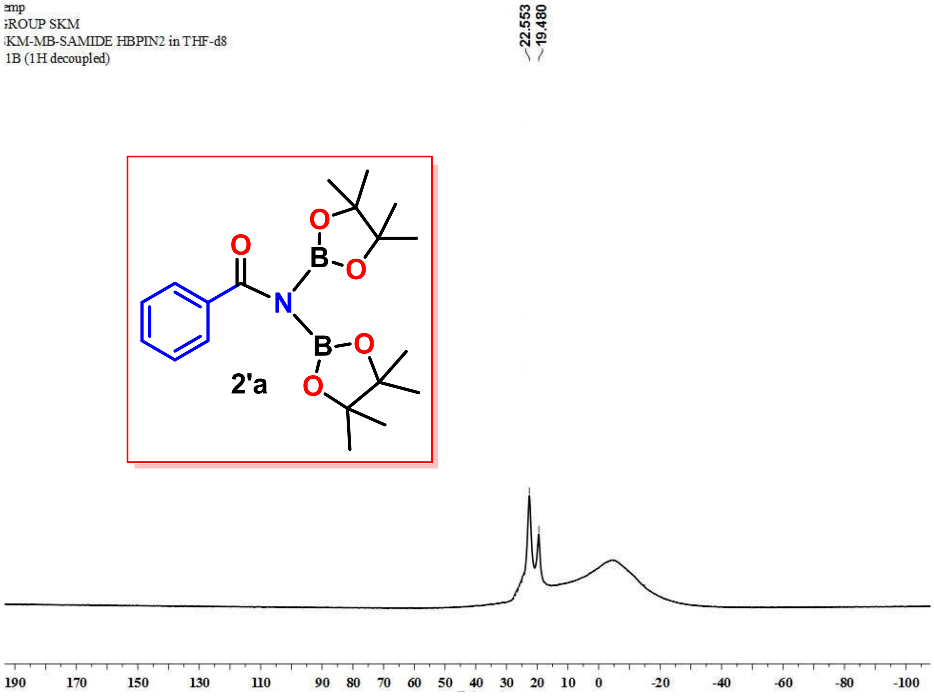

Figure S8. $^{11}B\{^{1}H\}$ NMR spectrum of borylated-amide (**2a'**) recorded in THF-d$_8$.

In situ NMR study to characterize the interaction between borylated-amide (2a') and $KN(SiMe_3)_2$.

An oven dried screw cap NMR tube was charged with borylated-amide, **2a'** (0.2 mmol), $KN(SiMe_3)_2$ (39.9 mg, 0.2 mmol) and THF-d_8 (600 μL) in a nitrogen filled glovebox. Subsequently, the reaction mixture was repeatedly shaken at room temperature. Next, the interaction between borylated-amide, **2a'** and $KN(SiMe_3)_2$ was characterized through ^{1}H, ^{13}C, and ^{11}B NMR spectroscopies. In ^{13}C NMR spectrum, ~ δ 9.9 ppm downfield shift of carbonyl carbon was observed as compared to that of **2a'**, and relatively low downfield shift was noticed in ^{11}B NMR (~ δ 1.3 ppm) spectroscopy. These observations clearly suggest the interaction between the K ion and carbonyl oxygen and along with this observation as well as taking into account the DFT calculations, formation of 7 was proposed.

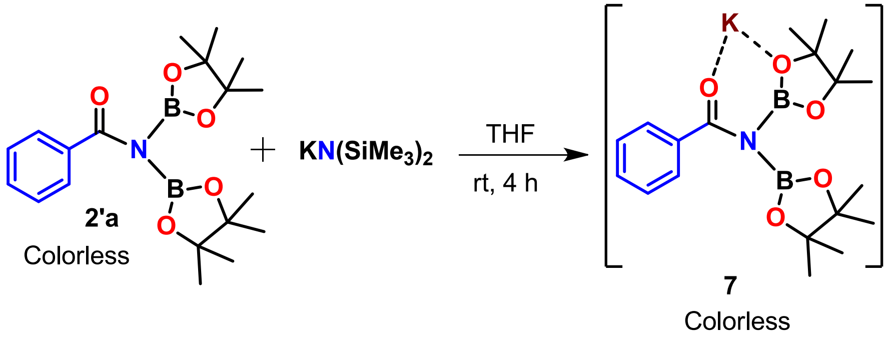

Scheme S8. Interaction between borylated-amide (**2a'**) and $KN(SiMe_3)_2$ in THF-d_8.

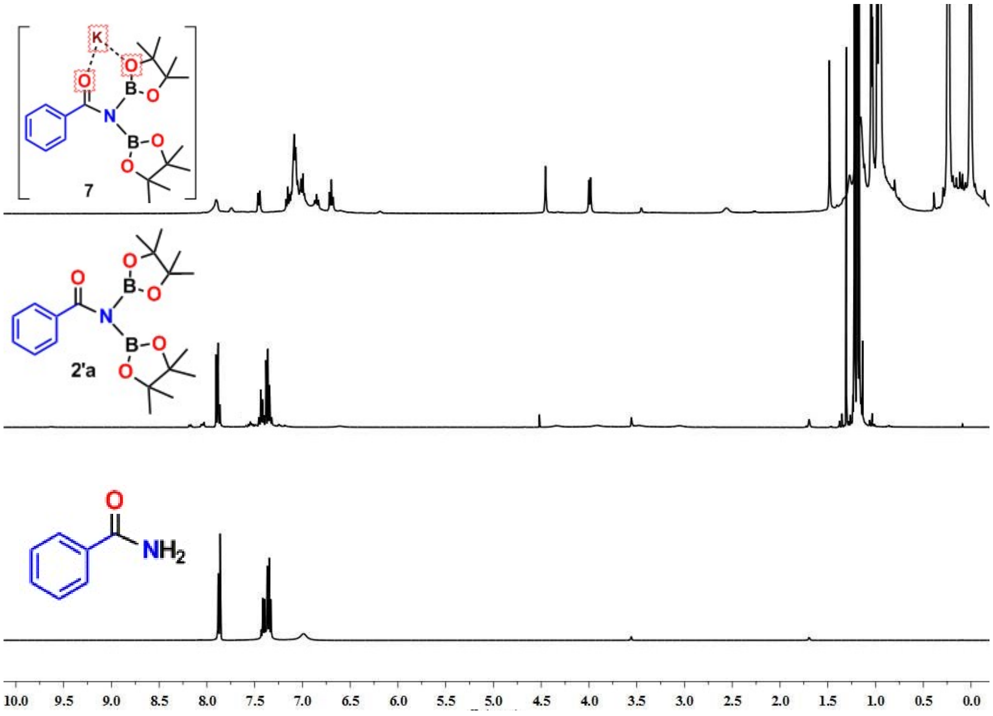

Figure S9. Stack plots for ^{1}H NMR spectra of benzamide, borylated amide (**2a'**), and reaction mixture in THF-d_8.

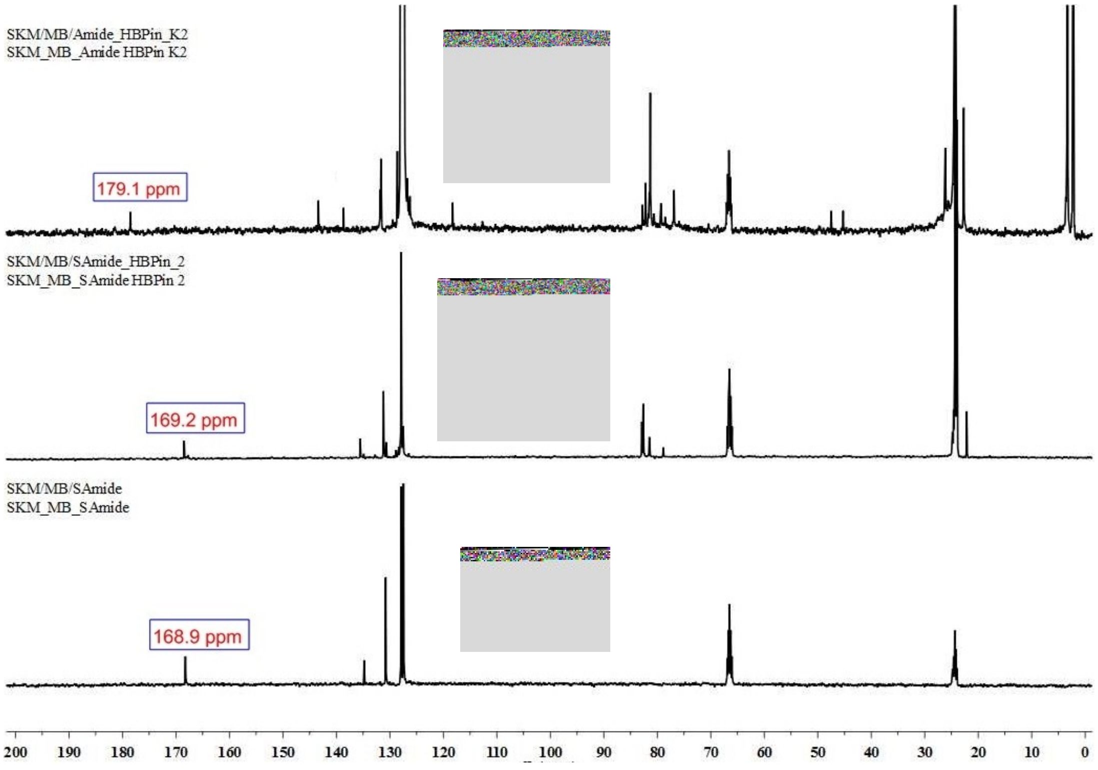

Figure S10. Stack plots of $^{13}C\{^{1}H\}$ NMR spectra of benzamide, borylated amide (**2a'**), and reaction mixture in THF-d_8.

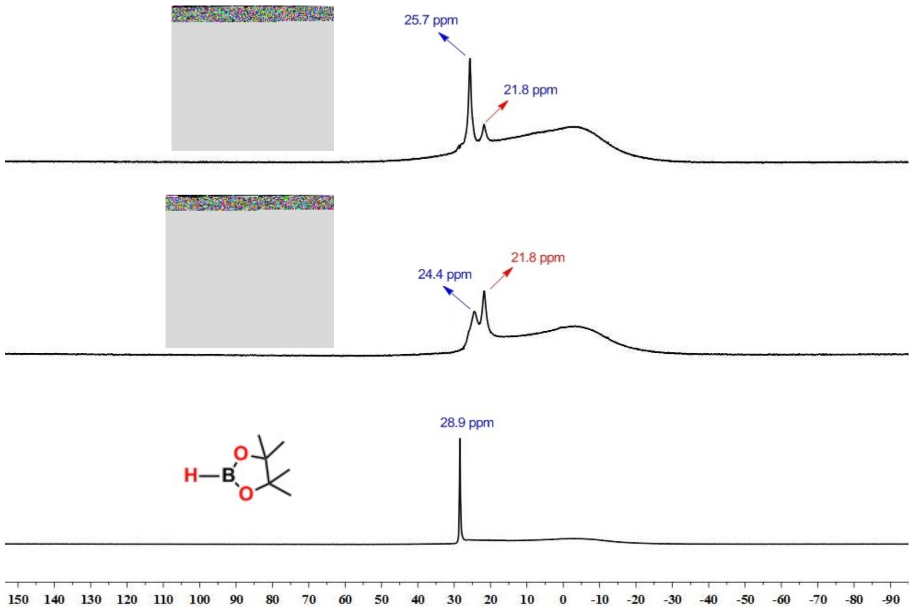

Figure S11. Stack plots for $^{11}B\{^{1}H\}$ NMR spectra of benzamide, borylated amide (**2a'**), and reaction mixture in C_6D_6.

5e. Characterization of *in situ* generated intermediate imine.

A screw cap NMR tube was charged with benzamide (12.1 mg, 0.1 mmol), or 4-chloro benzamide (15.6 mg, 0.1 mmol), HBPin (58 µL, 0.4 mmol), **1** (2.9 mg, 0.002 mmol, 2 mol%) and toluene-d_8 (600 µL) in a nitrogen filled glovebox and the reaction mixture was kept at 40 ^oC. Next, ^1H NMR spectroscopy of the reaction mixture was recorded after 2 h, when a resonance at δ 10.34 ppm for benzamide and δ 9.78 ppm for 4-chloro benzamide was observed in ^1H NMR spectroscopy. Also a resonance at δ 172.7 ppm for benzamide appeared in ^{13}C NMR spectrum, which clearly indicates the formation of an imine intermediate (Scheme S9).

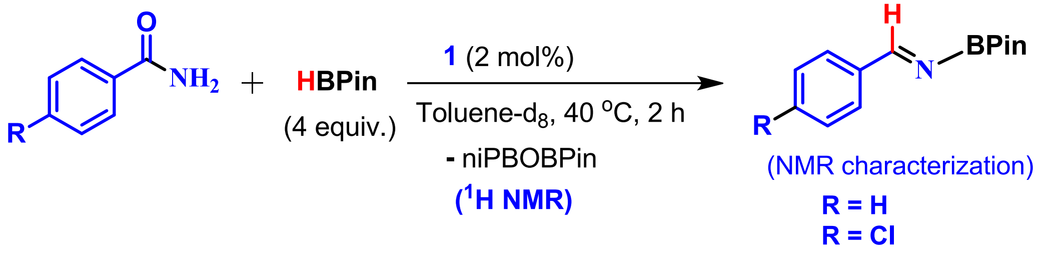

Scheme S9. Synthetic scheme for the formation of intermediate imine.

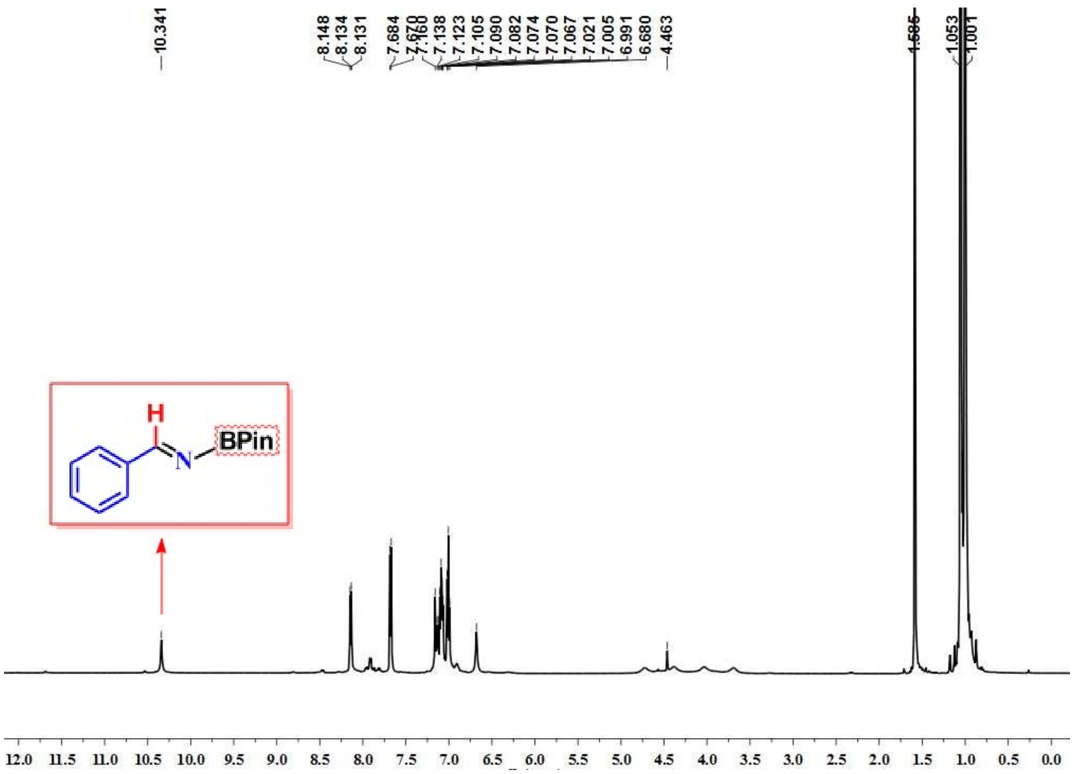

Figure S12. ^1H NMR spectrum of *in situ* generated N-borylated imine (**9a**) recorded in C_6D_6.

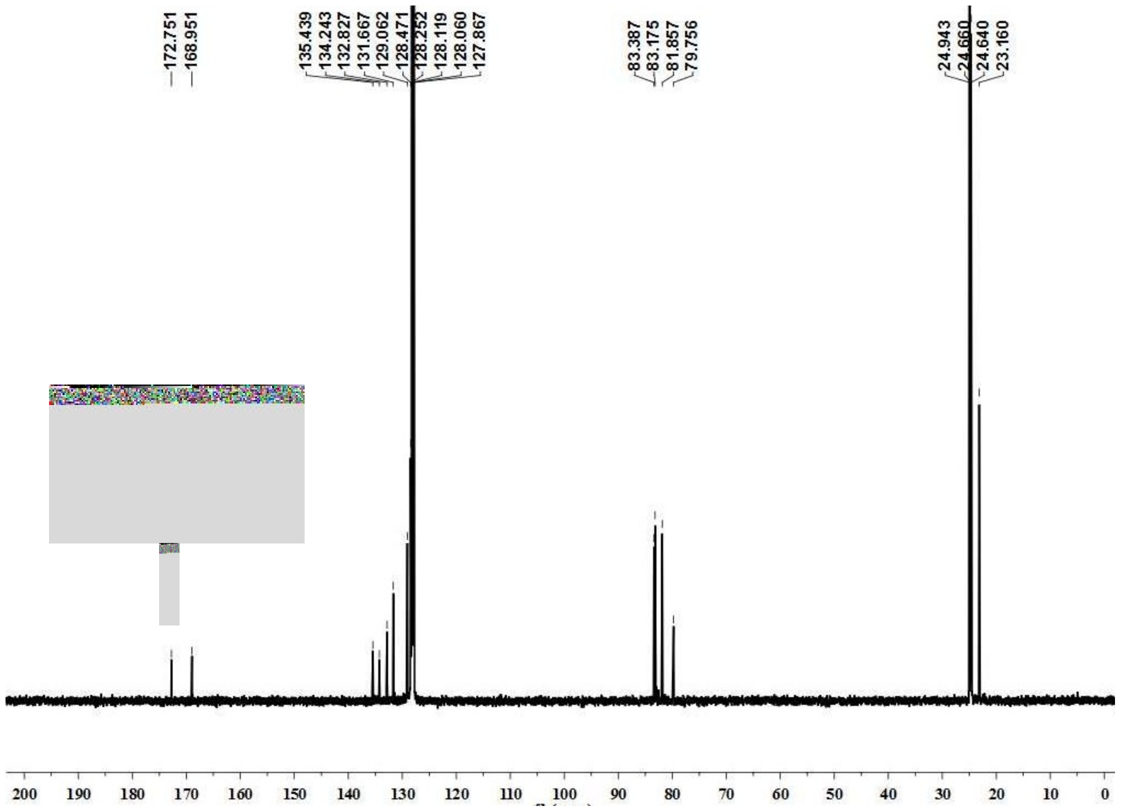

Figure S13. $^{13}C\{^{1}H\}$ NMR spectrum of *in situ* generated N-borylated imine (**9a**) recorded in C_6D_6.

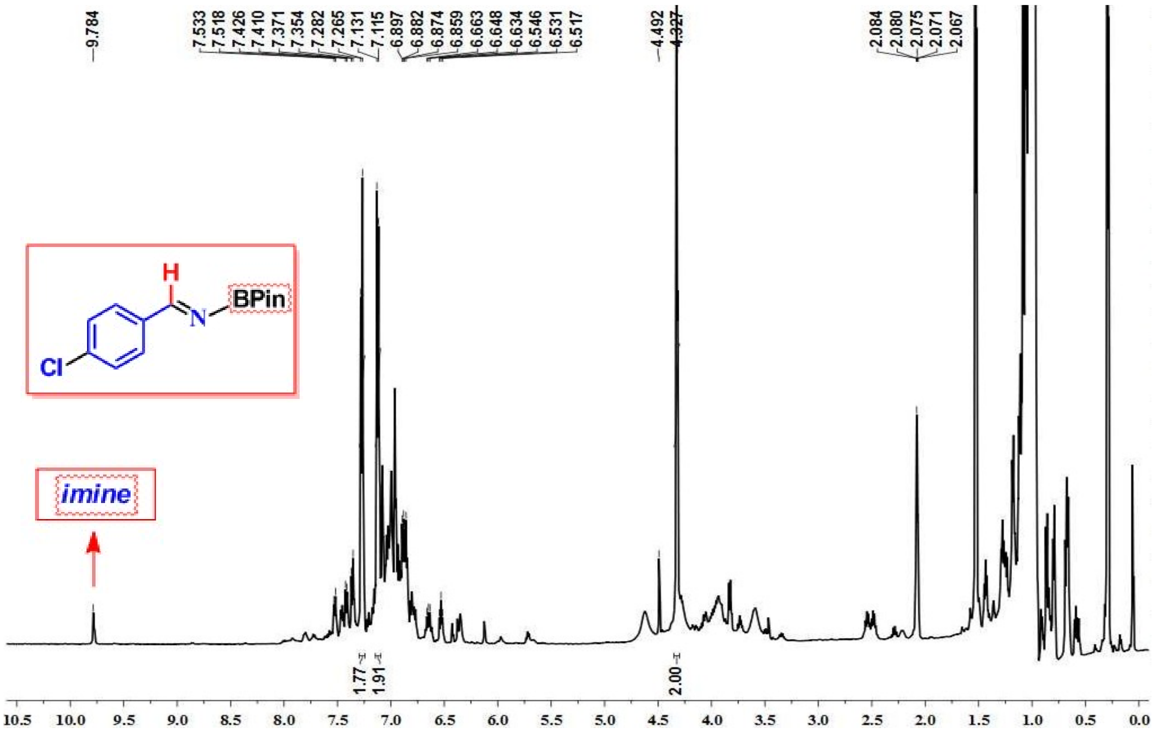

Figure S14. ^{1}H NMR spectrum of *in situ* generated N-borylated imine (**9a**) from 4-chloro benzamide recorded in toluene-d_8.

5f. Preparation of N,N-diborylated amine upon hydroborylation of benzamide.

An oven dried 20 mL reaction tube was charged with [*a*NHC.KN(SiMe$_3$)$_2$]$_2$, **1** (14.8 mg, 2 mol%) and pinacolborane (290 μL, 2.0 mmol, 4 equivalent) along with 1 mL toluene-d$_8$ inside a N_2 filled glovebox. Subsequently benzamide (0.5 mmol) was added to the reaction mixture and stirred for 12h at 40 °C. After completion of the reaction NMR was recorded in toluene-d$_8$.[S19]

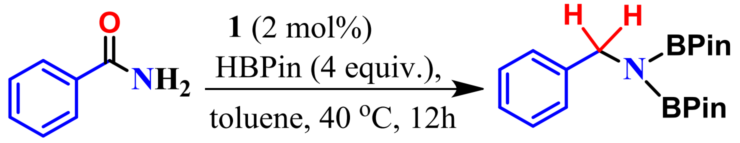

2a **10**

Scheme S10. Synthetic scheme for the formation of N,N-diborylated amine from benzamide.

6. NMR data of primary amine derivatives upon reduction of primary amides.

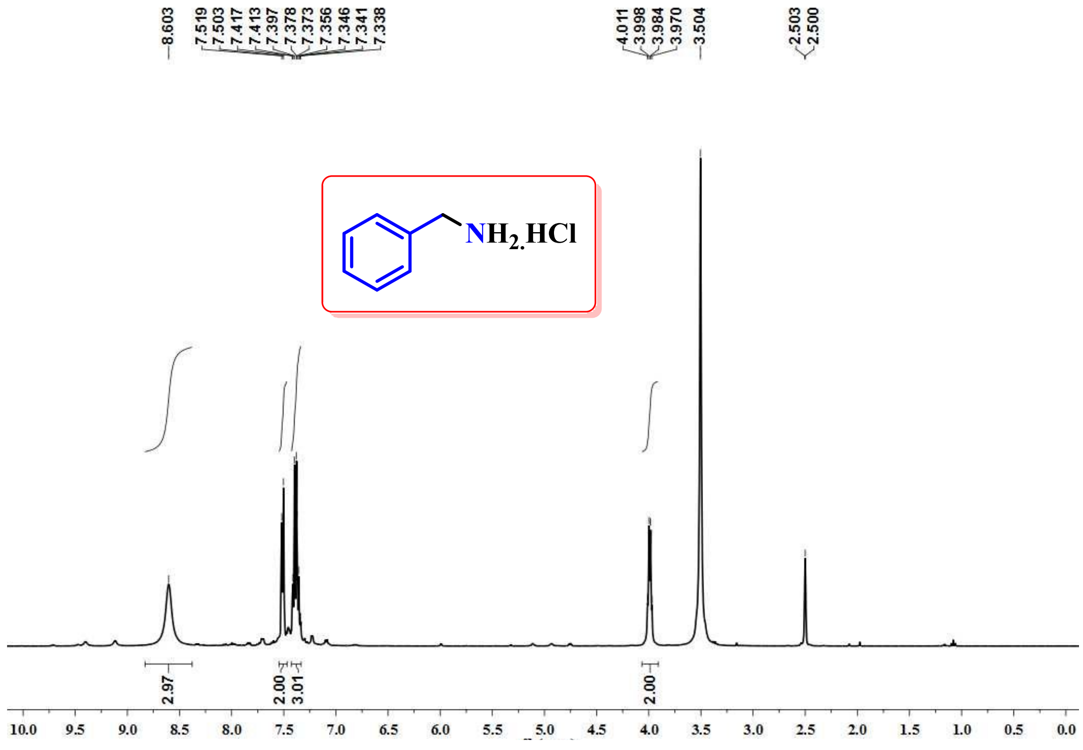

Figure S15. ^{1}H NMR spectrum of phenylmethanamine hydrochloride (**3a**) recorded in DMSO-d_6.

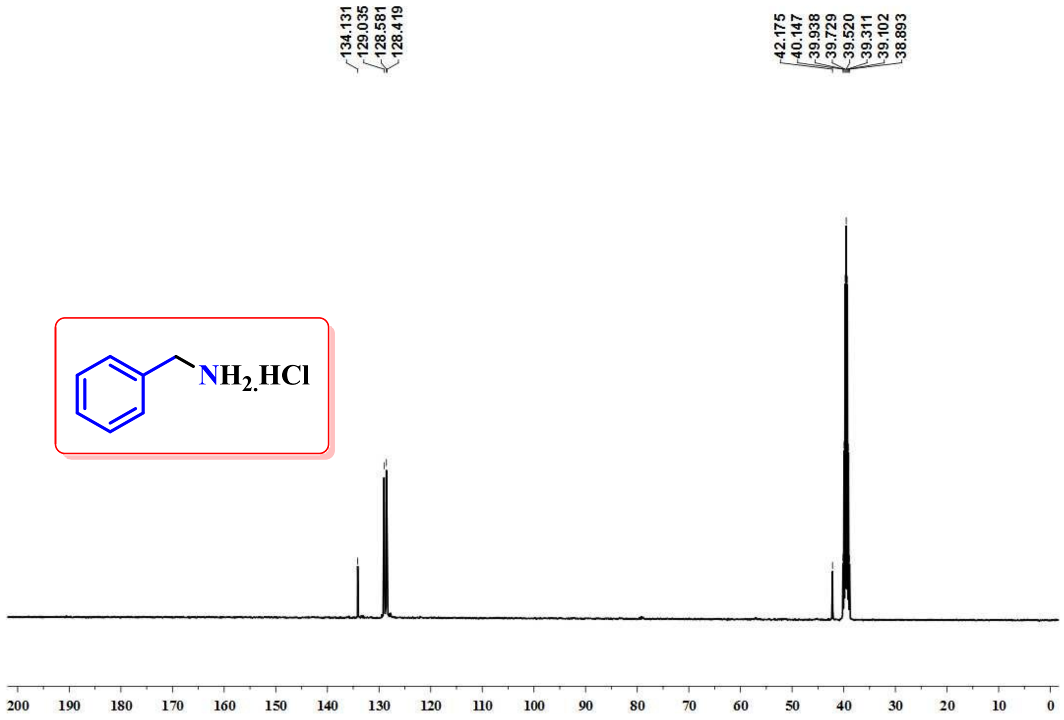

Figure S16. $^{13}C\{^{1}H\}$ NMR spectrum of phenylmethanamine hydrochloride (**3a**) recorded in DMSO-d_6.

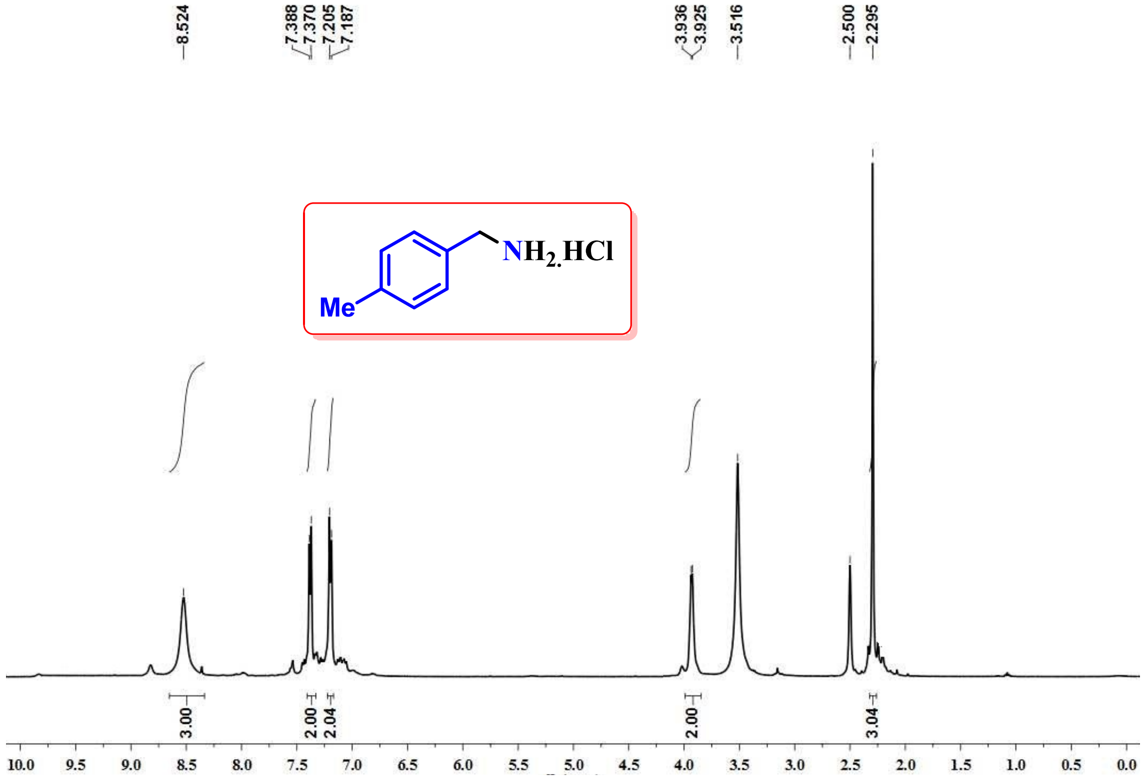

Figure S17. ^{1}H NMR spectrum of *p*-tolylmethanamine hydrochloride (**3b**) recorded in DMSO-d_6.

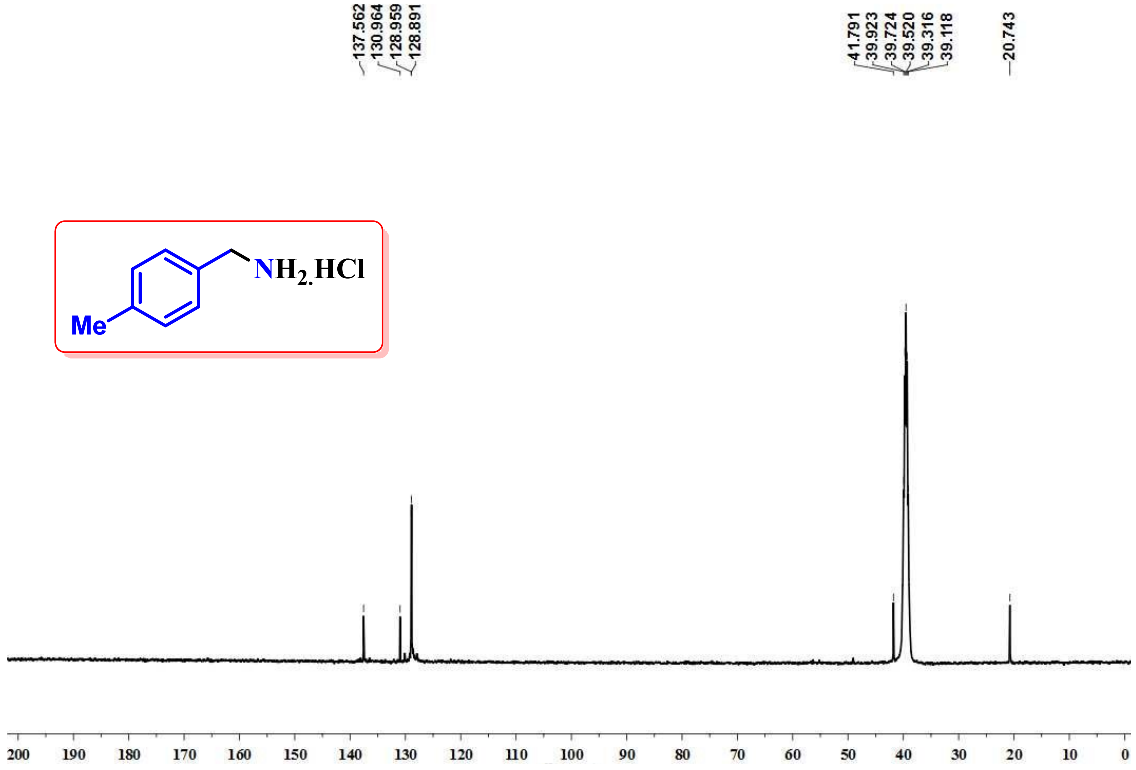

Figure S18. $^{13}C\{^1H\}$ NMR spectrum of *p*-tolylmethanamine hydrochloride (**3b**) recorded in DMSO-d_6.

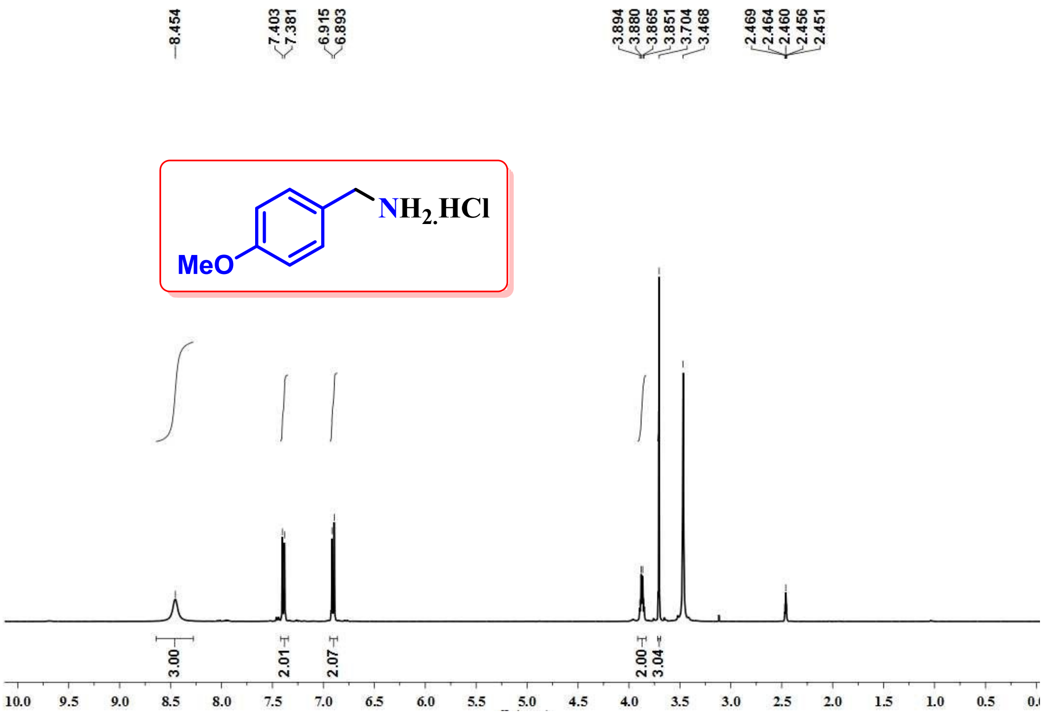

Figure S19. ^{1}H NMR spectrum of (4-methoxyphenyl)methanamine hydrochloride (**3c**) recorded in DMSO-d_6.

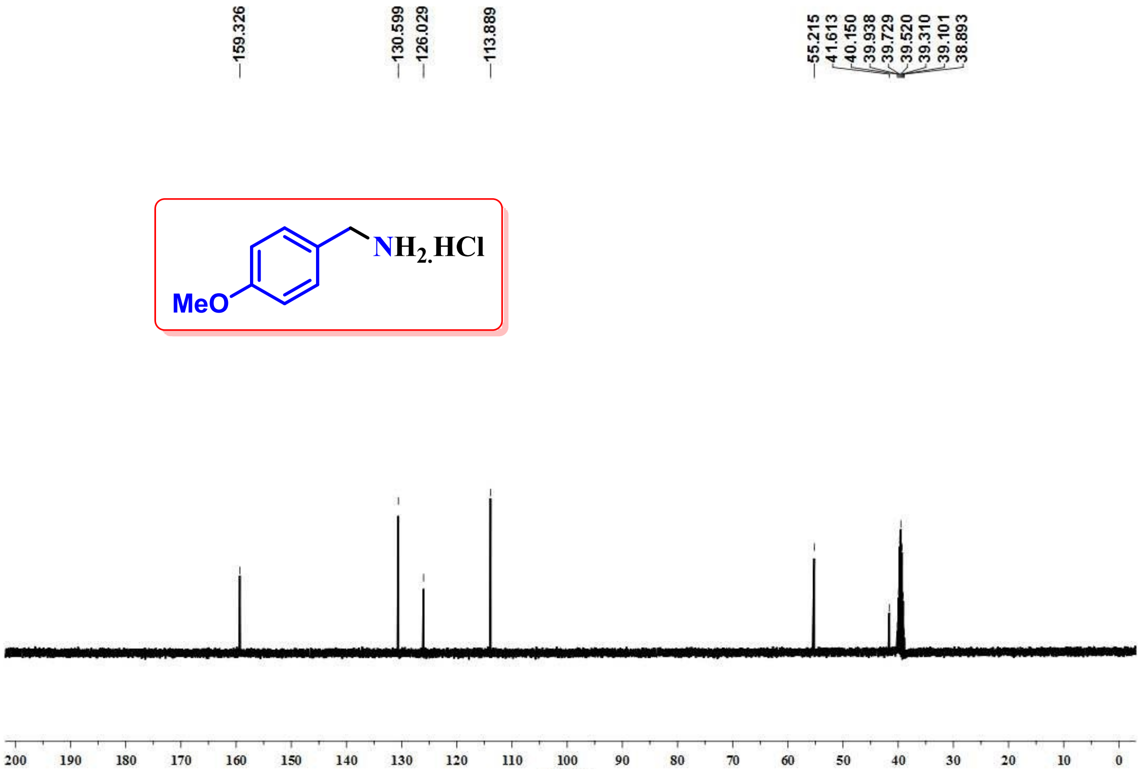

Figure S20. $^{13}C\{^{1}H\}$ NMR spectrum of (4-methoxyphenyl)methanamine hydrochloride (**3c**) recorded in DMSO-d_6.

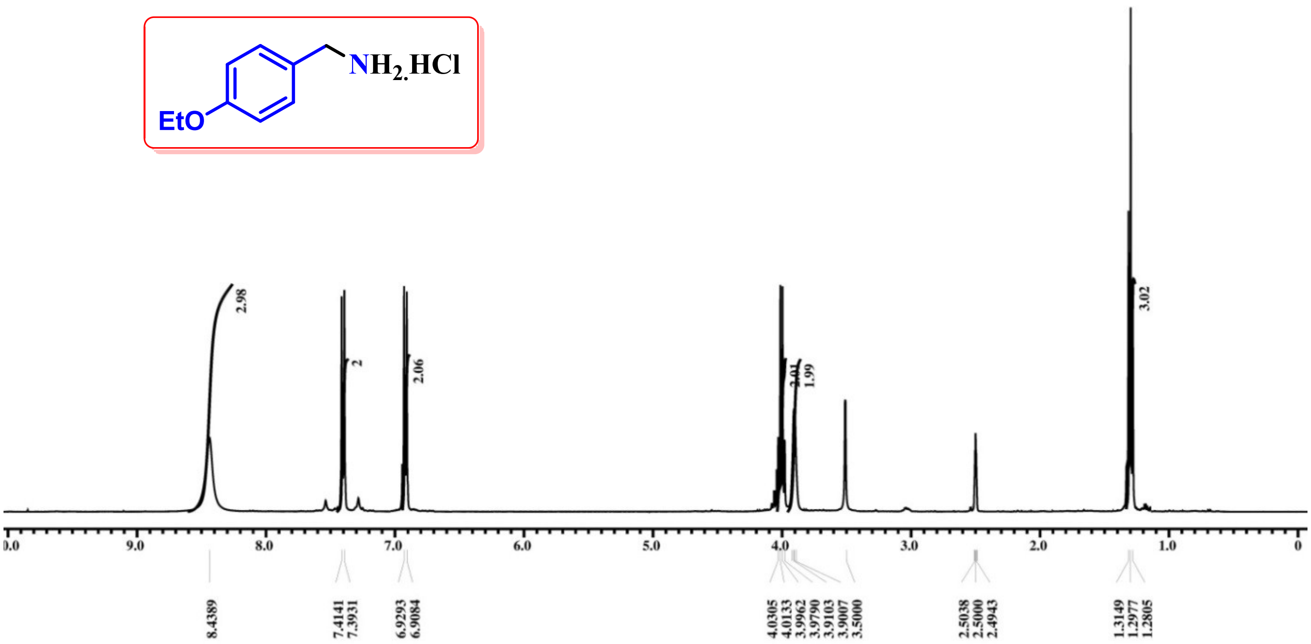

Figure S21. ^{1}H NMR spectrum of (4-ethoxyphenyl)methanamine hydrochloride (**3d**) recorded in DMSO-d_6.

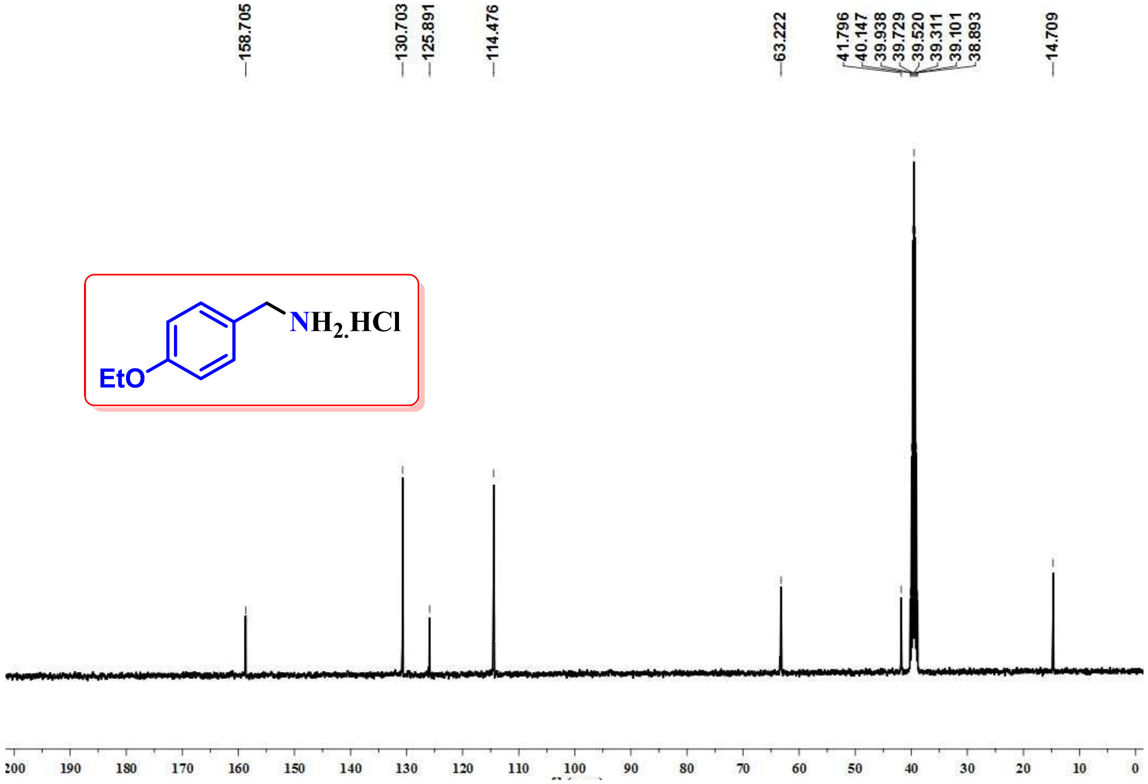

Figure S22. ^{13}C{^{1}H} NMR spectrum of (4-ethoxyphenyl)methanamine hydrochloride (**3d**) recorded in DMSO-d_6.

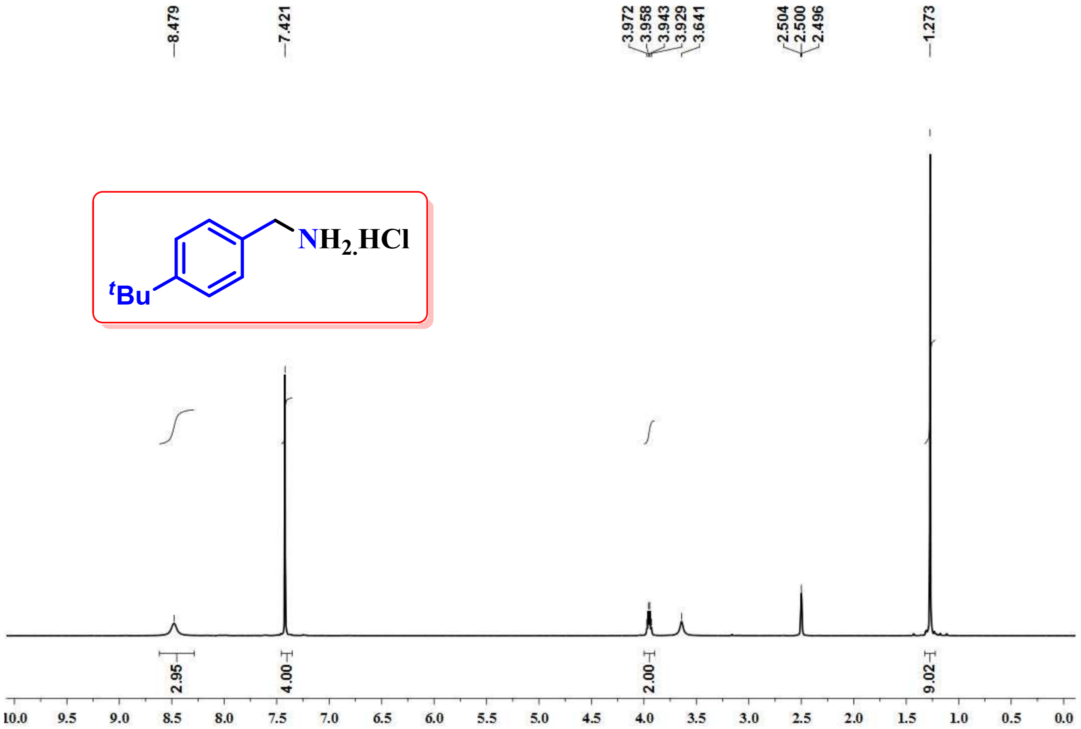

Figure S23. ^{1}H NMR spectrum of (4-(tert-butyl)phenyl)methanamine hydrochloride (**3e**) recorded in DMSO-d_6.

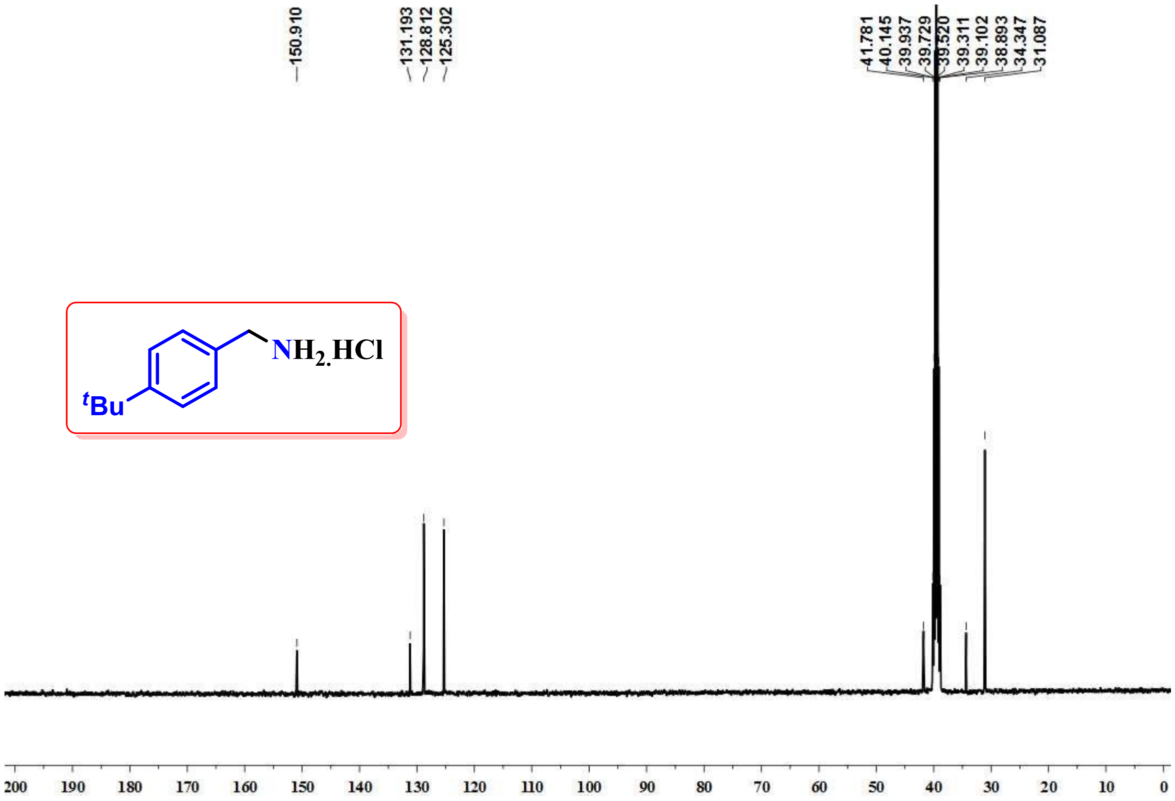

Figure S24. ^{13}C{^{1}H} NMR spectrum of (4-(tert-butyl)phenyl)methanamine hydrochloride (**3e**) recorded in DMSO-d_6.

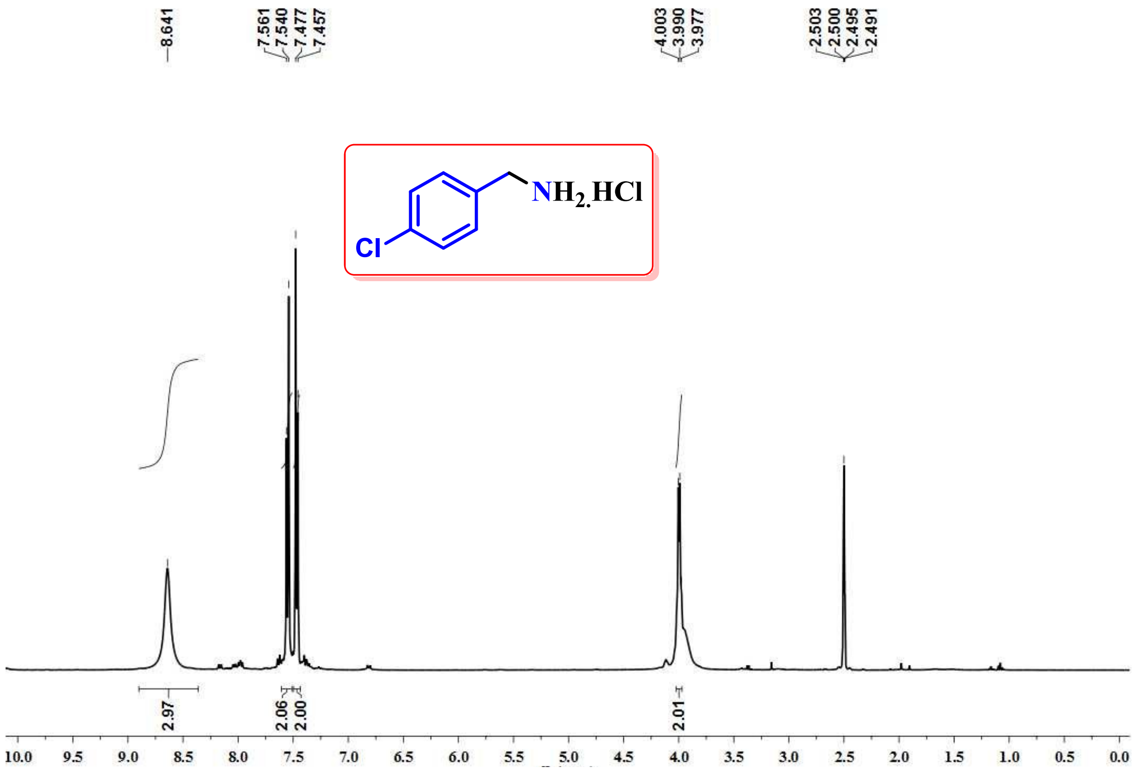

Figure S25. ^{1}H NMR spectrum of (4-chlorophenyl)methanamine hydrochloride (**3f**) recorded in DMSO-d_6.

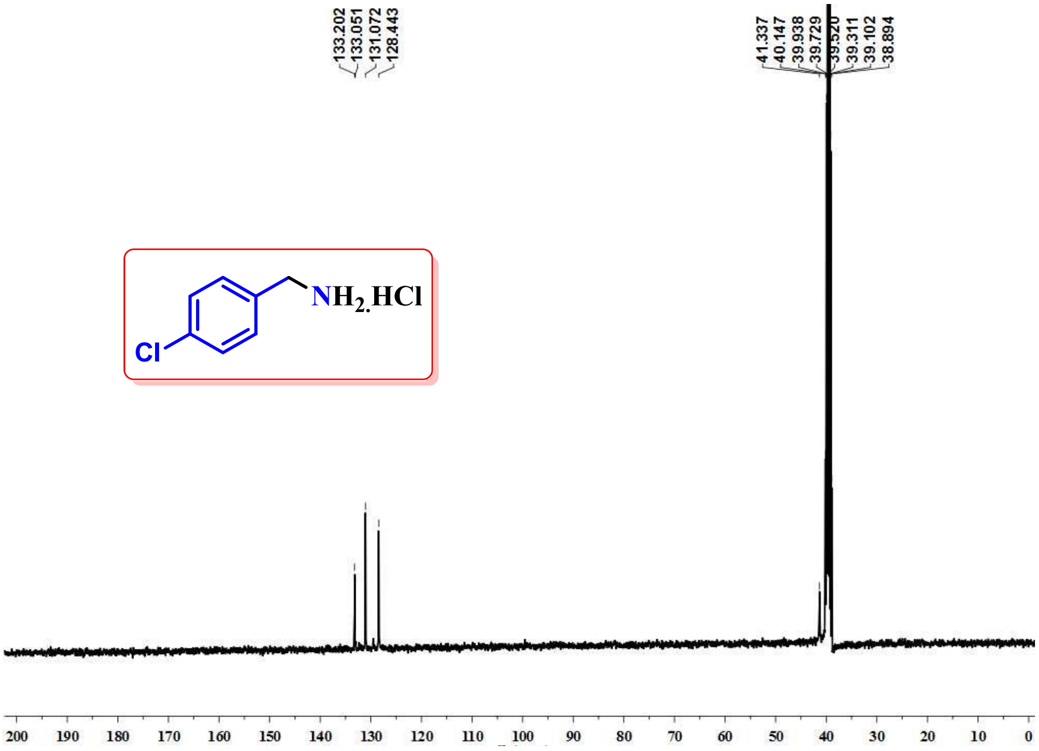

Figure S26. $^{13}C\{^{1}H\}$ NMR spectrum of (4-chlorophenyl)methanamine hydrochloride (**3f**) recorded in DMSO-d_6.

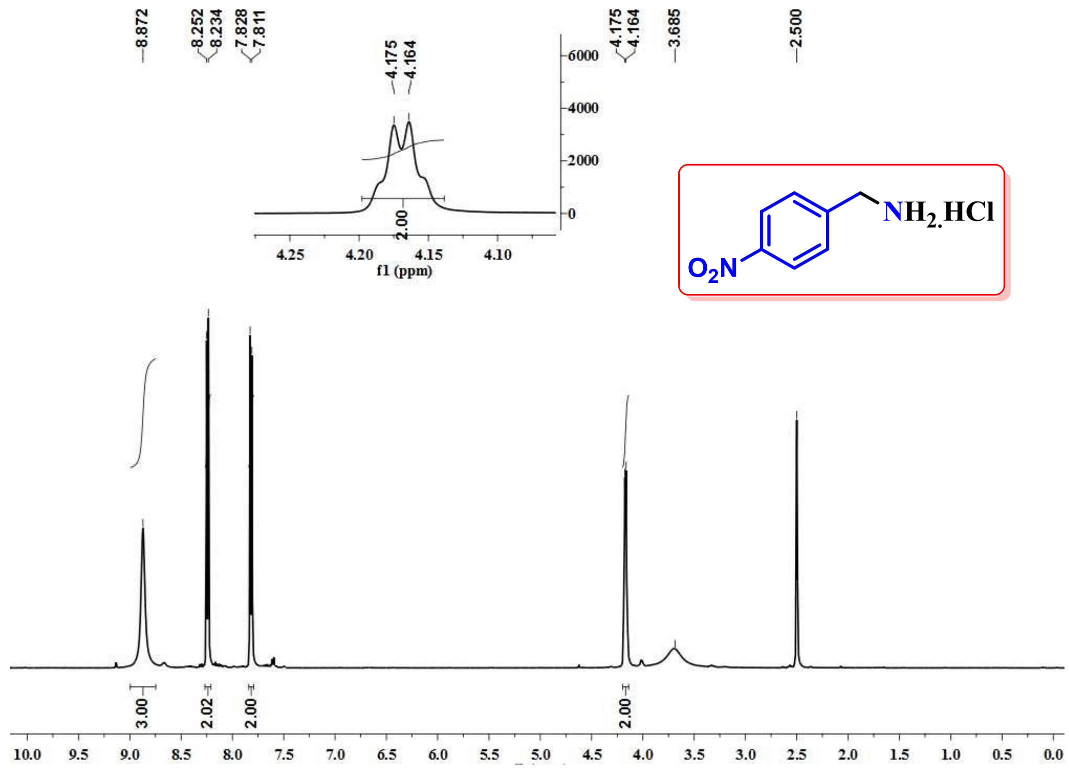

Figure S27. ^{1}H NMR spectrum of (4-nitrophenyl)methanamine hydrochloride (**3g**) in DMSO-d_6.

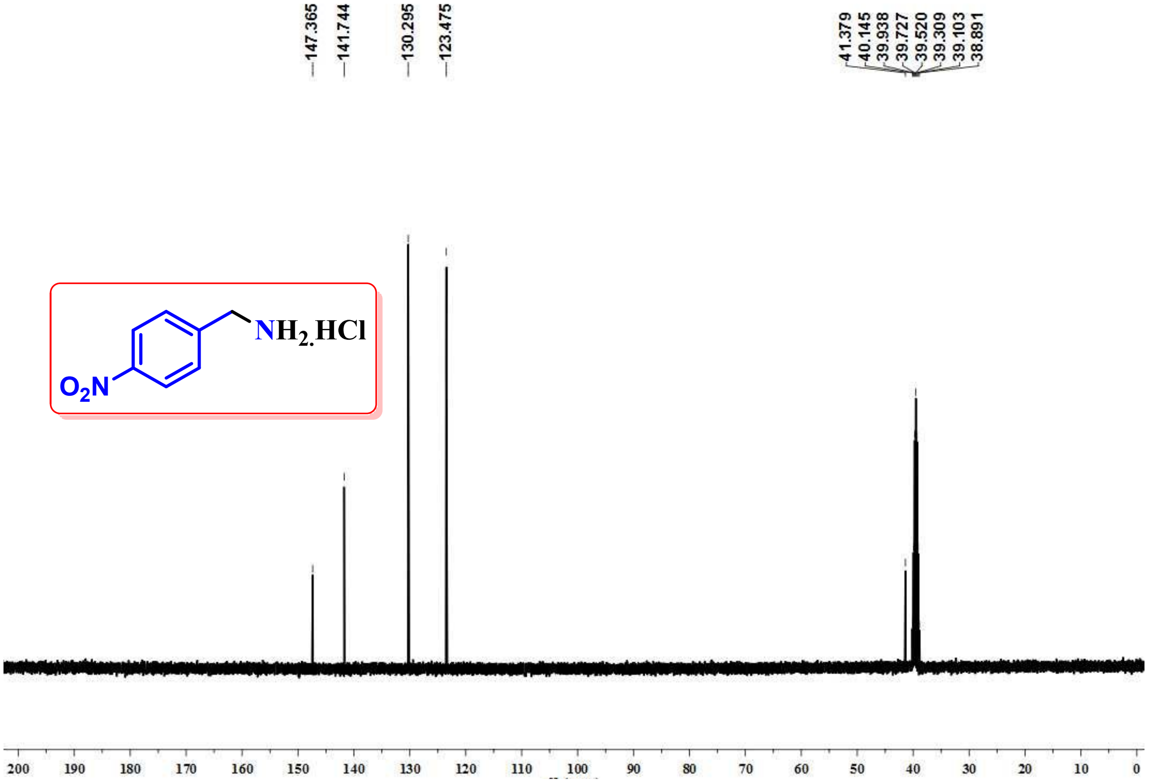

Figure S28. $^{13}C\{^{1}H\}$ NMR spectrum of (4-nitrophenyl)methanamine hydrochloride (**3g**) in DMSO-d_6.

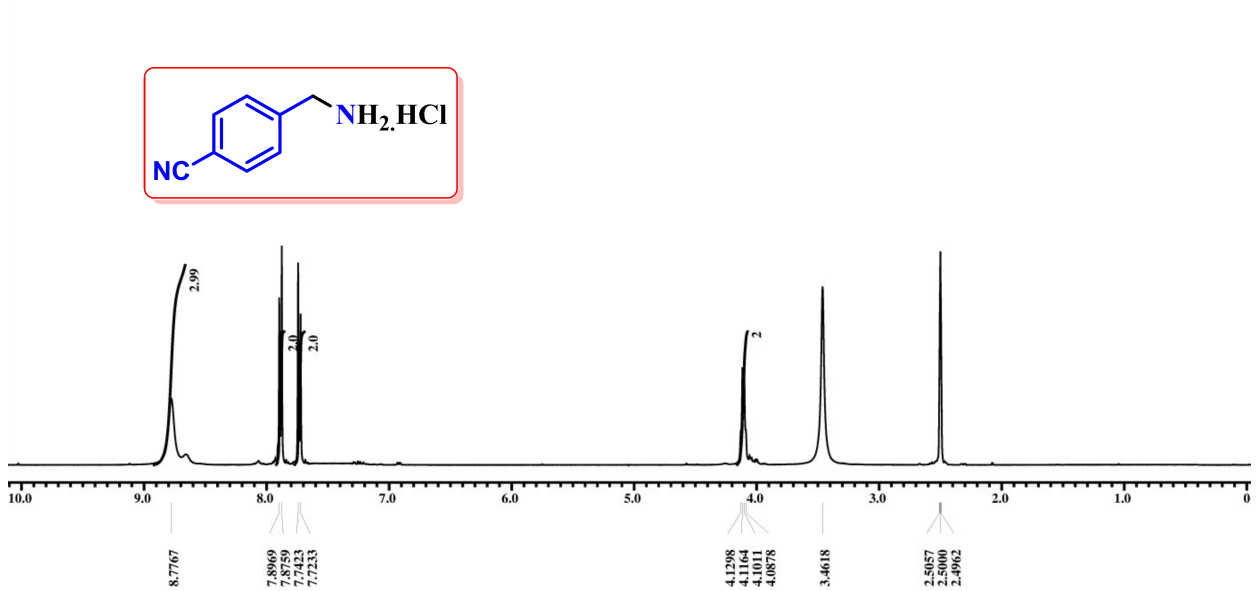

Figure S29. ^{1}H NMR spectrum of 4-(aminomethyl)benzonitrile hydrochloride (**3h**) in DMSO-d_6.

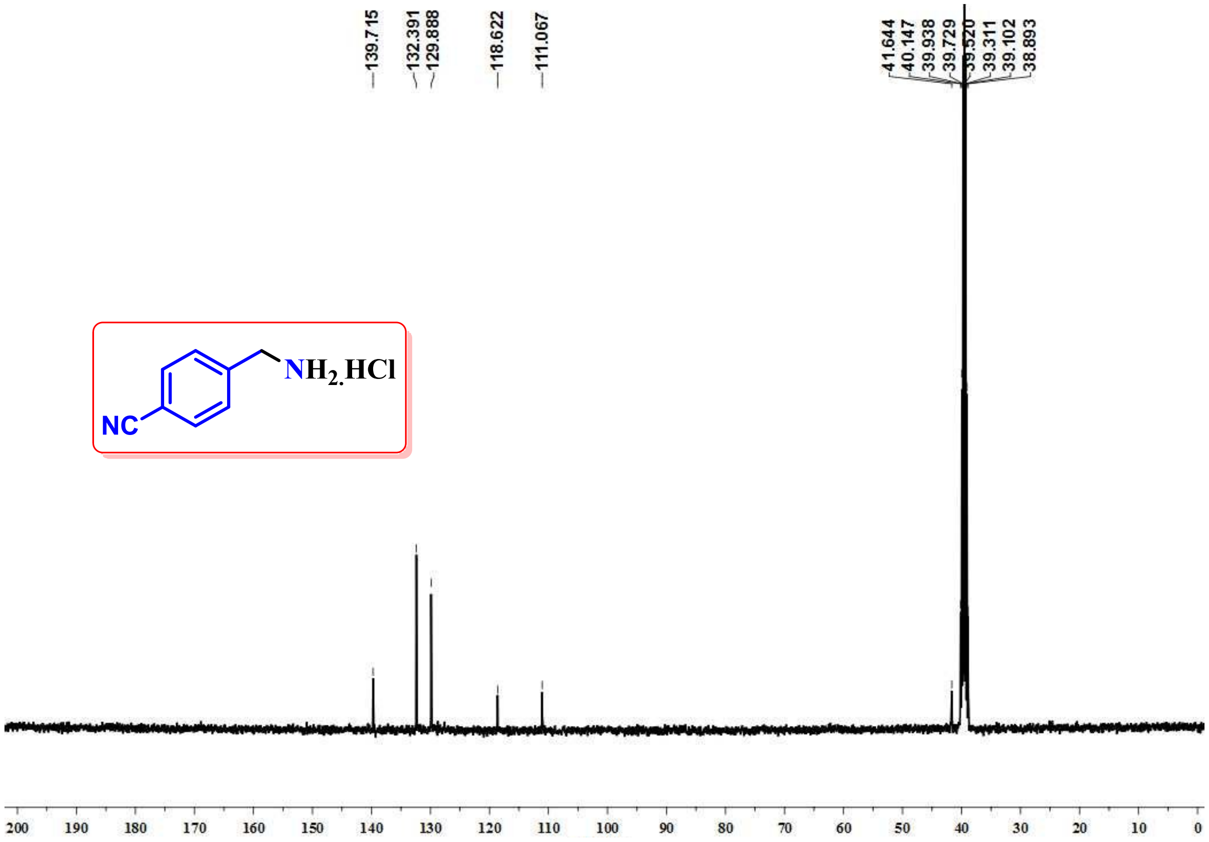

Figure S30. ^{13}C{^{1}H} NMR spectrum of 4-(aminomethyl)benzonitrile hydrochloride (**3h**) in DMSO-d_6.

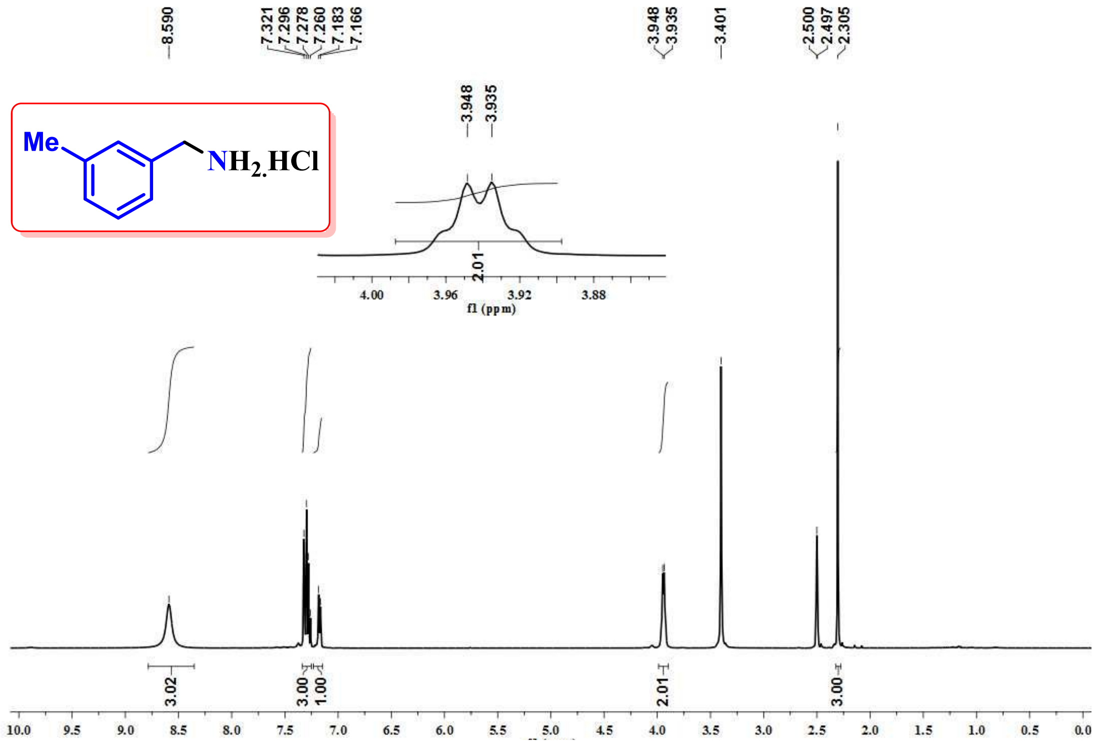

Figure S31. ^{1}H NMR spectrum of *m*-tolylmethanamine hydrochloride (**3i**) in DMSO-d$_6$.

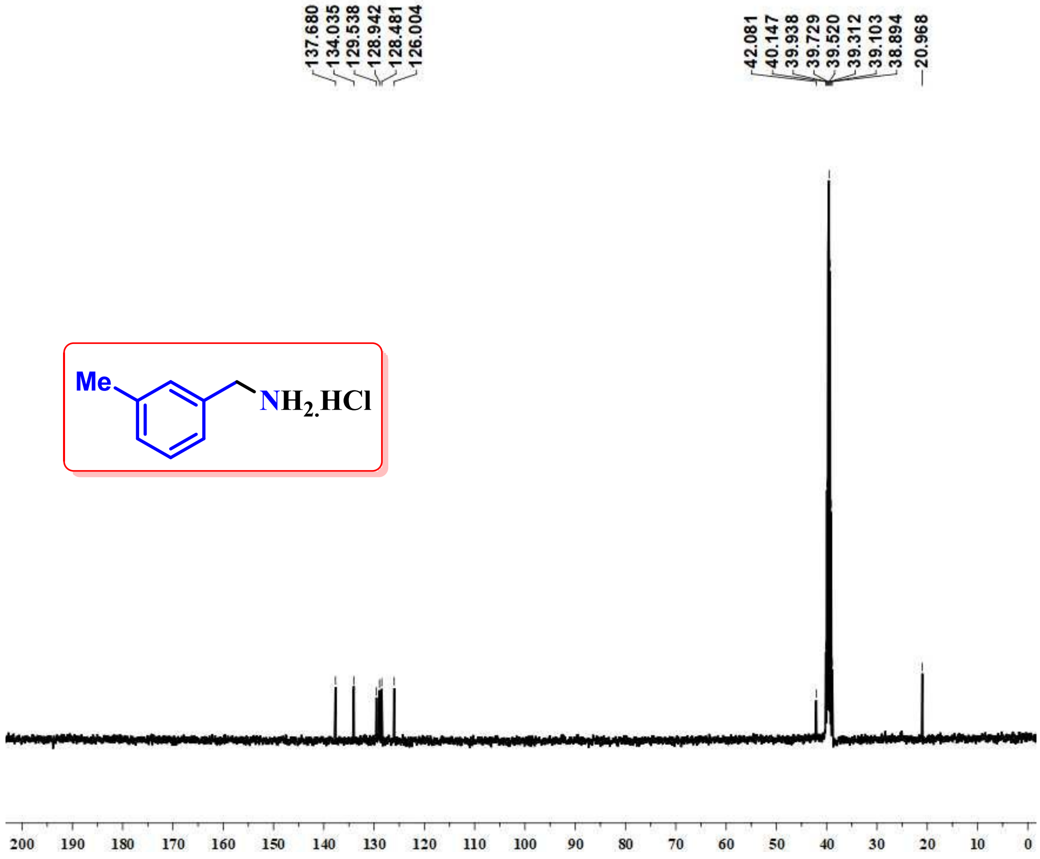

Figure S32. ^{13}C{^{1}H} NMR spectrum of *m*-tolylmethanamine hydrochloride (**3i**) in DMSO-d$_6$.

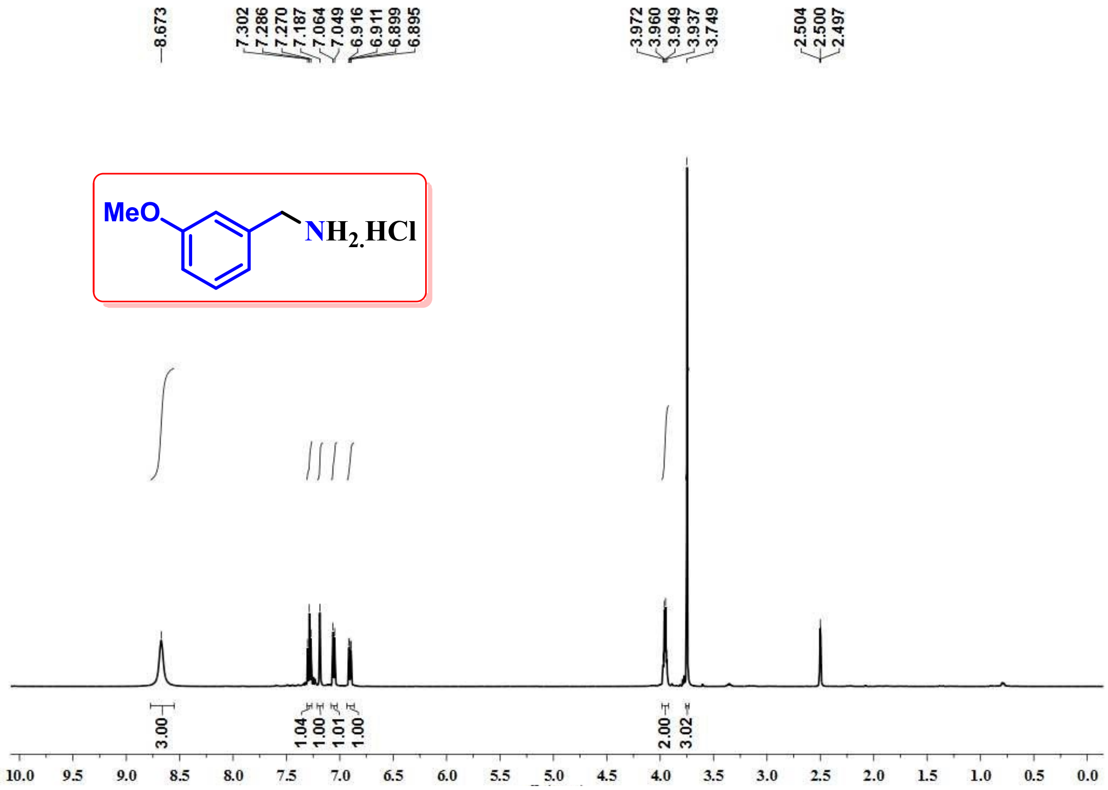

Figure S33. ^{1}H NMR spectrum of (3-methoxyphenyl)methanamine hydrochloride (**3j)** in DMSO-d_6.

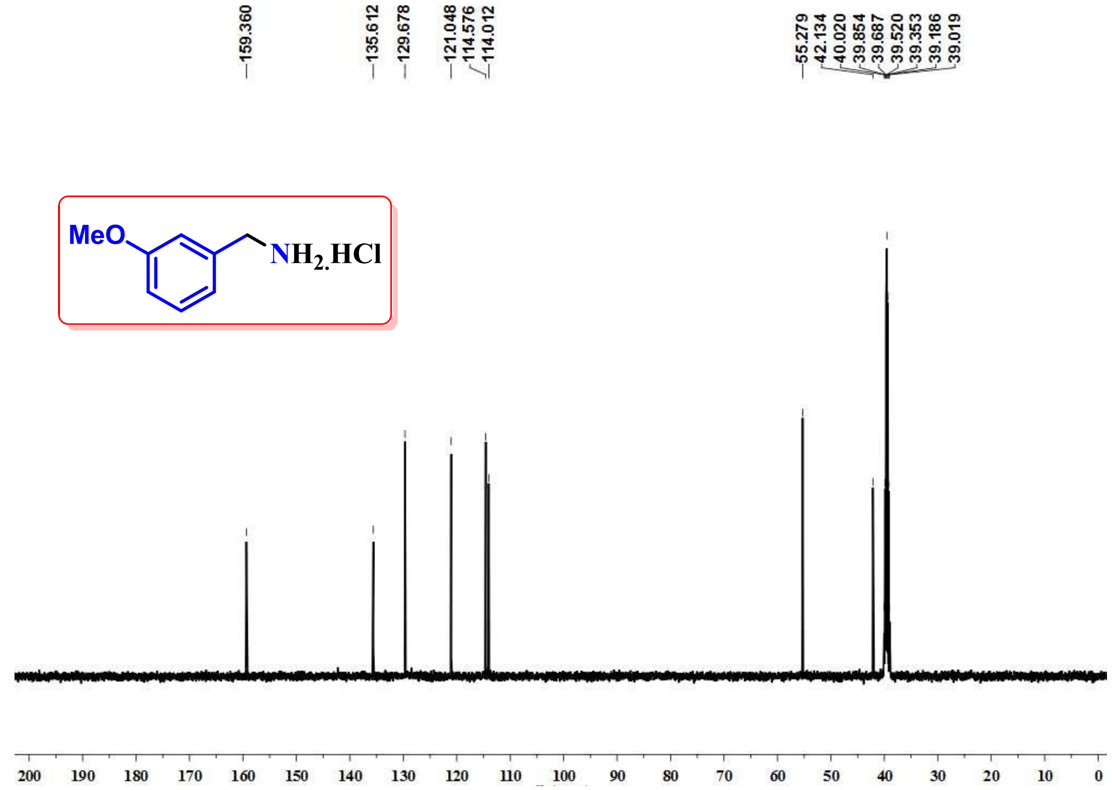

Figure S34. ^{13}C{^{1}H} NMR spectrum of (3-methoxyphenyl)methanamine hydrochloride (**3j)** in DMSO-d_6.

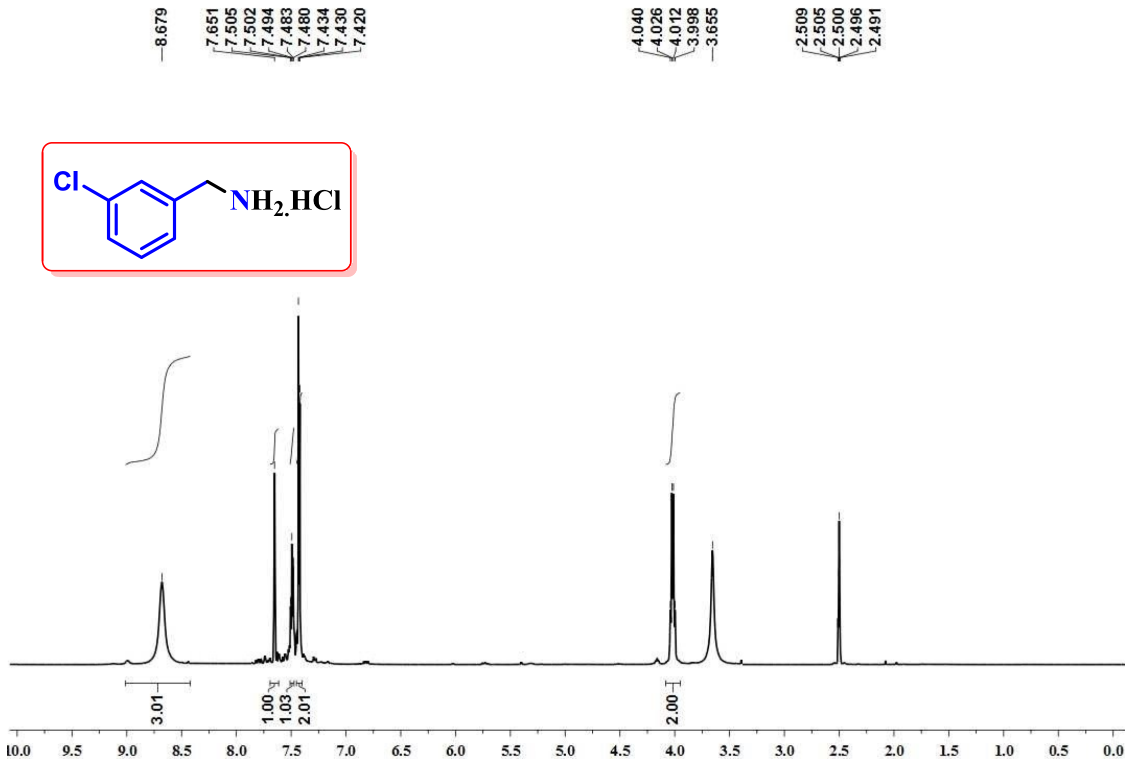

Figure S35. ^{1}H NMR spectrum of (3-chlorophenyl)methanamine hydrochloride (**3k**) in DMSO-d_6.

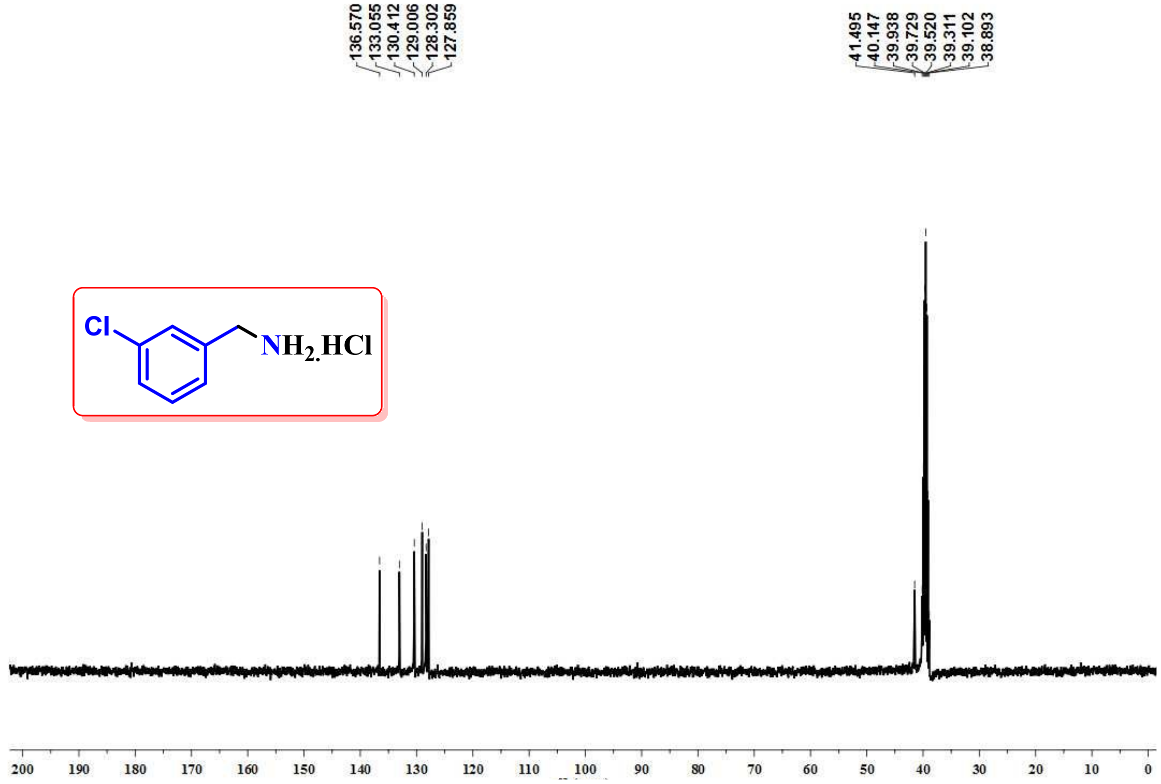

Figure S36. $^{13}C\{^{1}H\}$ NMR spectrum of (3-chlorophenyl)methanamine hydrochloride (**3k**) in DMSO-d_6.

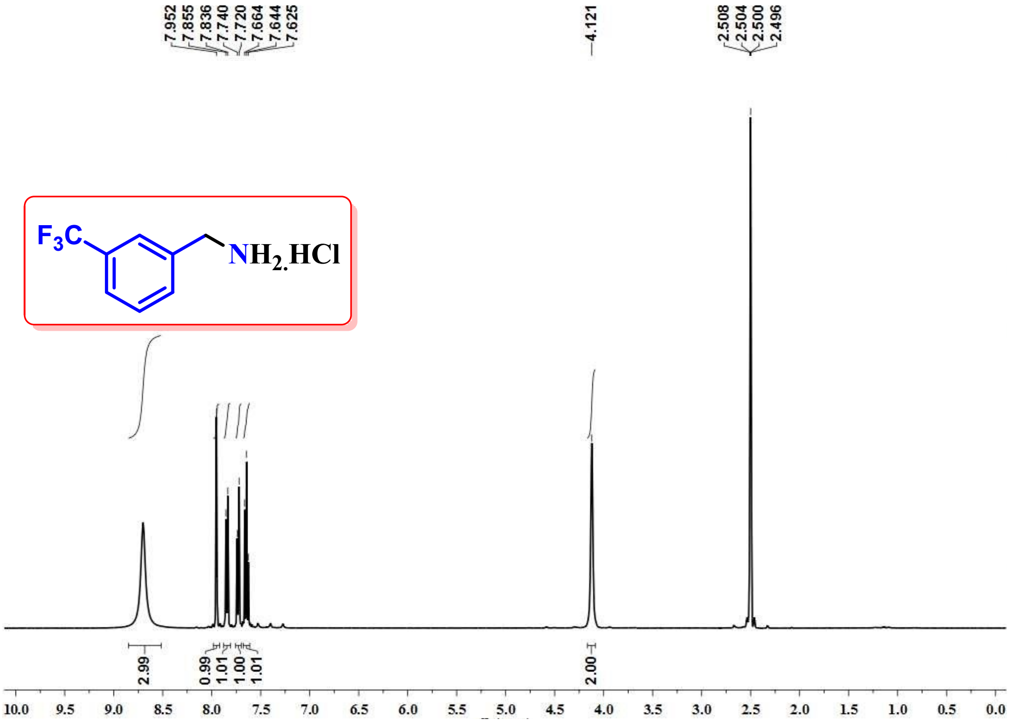

Figure S37. ^{1}H NMR spectrum of (3-(trifluoromethyl)phenyl)methanamine hydrochloride (**3l)** in DMSO-d_6.

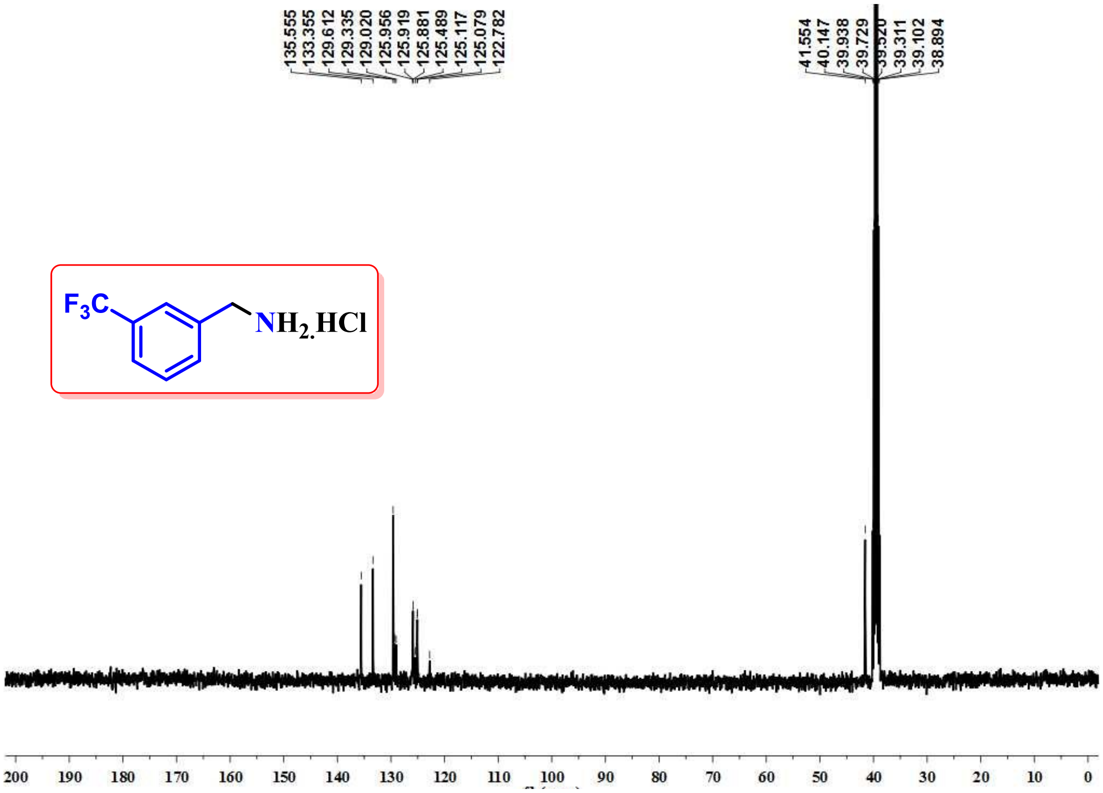

Figure S38. ^{13}C{^{1}H} NMR spectrum of (3-(trifluoromethyl)phenyl)methanamine hydrochloride (**3l)** in DMSO-d_6.

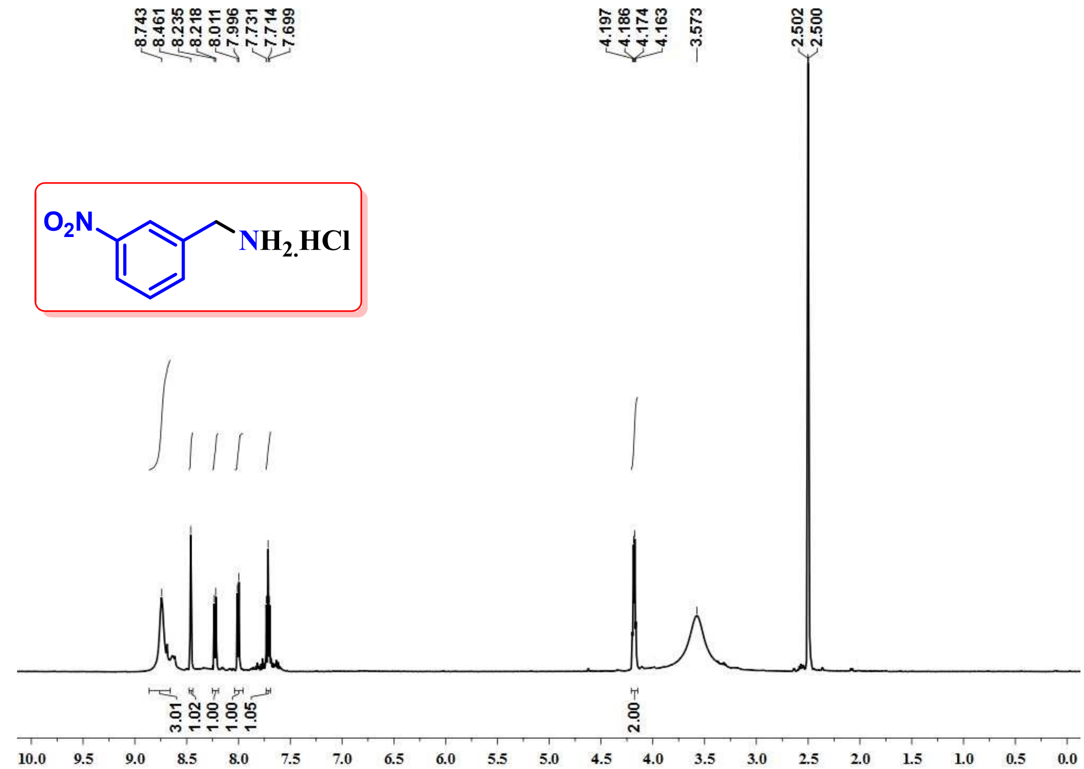

Figure S39. ^{1}H NMR spectrum of (3-nitrophenyl)methanamine hydrochloride (**3m)** in DMSO-d$_6$.

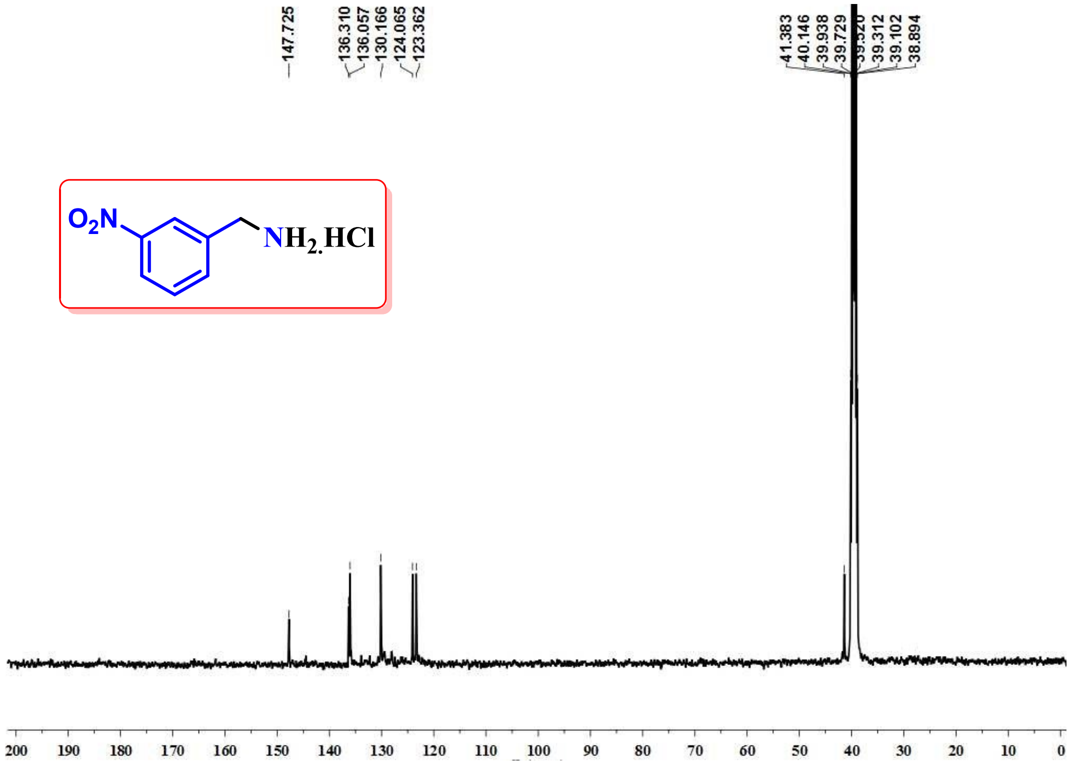

Figure S40. ^{13}C{^{1}H} NMR spectrum of (3-nitrophenyl)methanamine hydrochloride (**3m)** in DMSO-d$_6$.

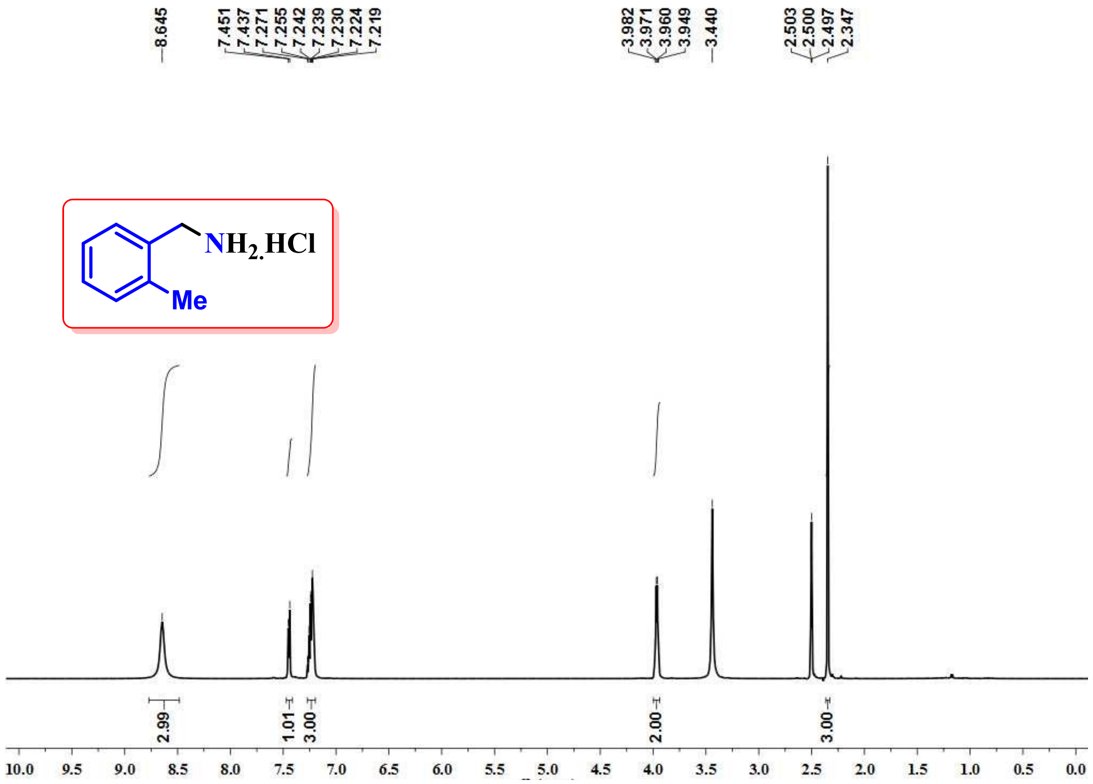

Figure S41. ^{1}H NMR spectrum of *o*-tolylmethanamine hydrochloride (**3n)** in DMSO-d_6.

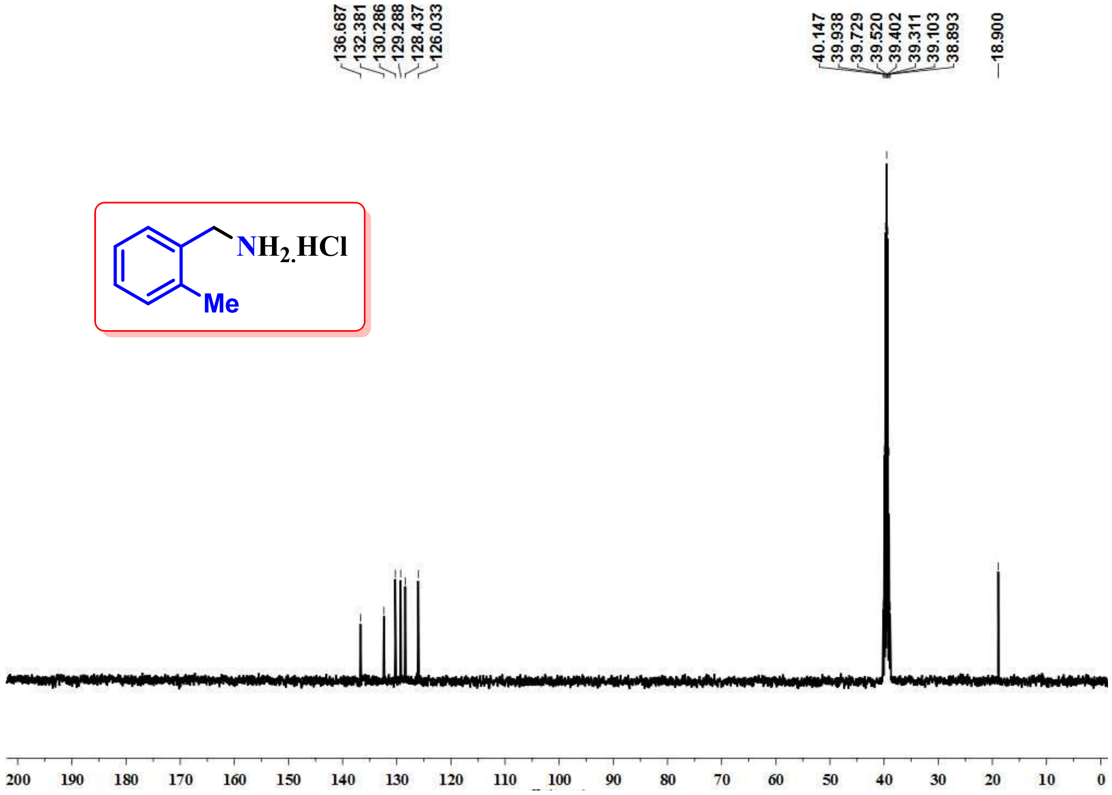

Figure S42. $^{13}C\{^{1}H\}$ NMR spectrum of *o*-tolylmethanamine hydrochloride (**3n)** in DMSO-d_6.

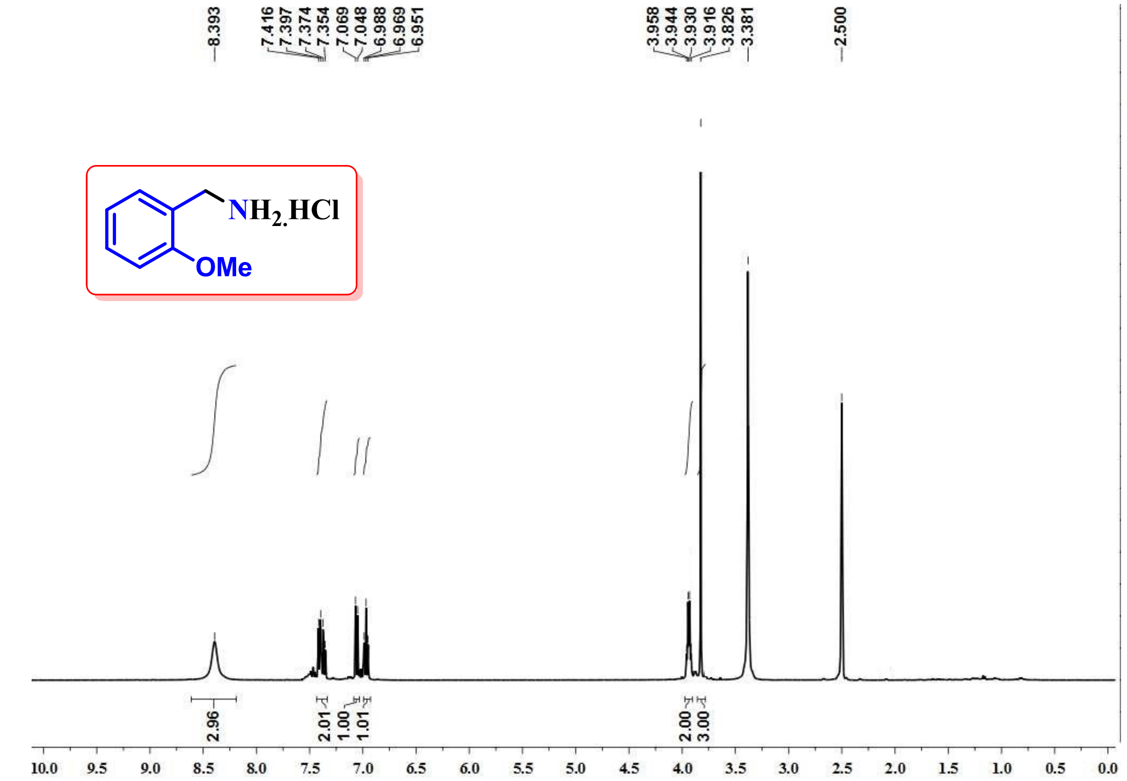

Figure S43. ^{1}H NMR spectrum of (2-methoxyphenyl)methanamine hydrochloride (**3o)** in DMSO-d_6.

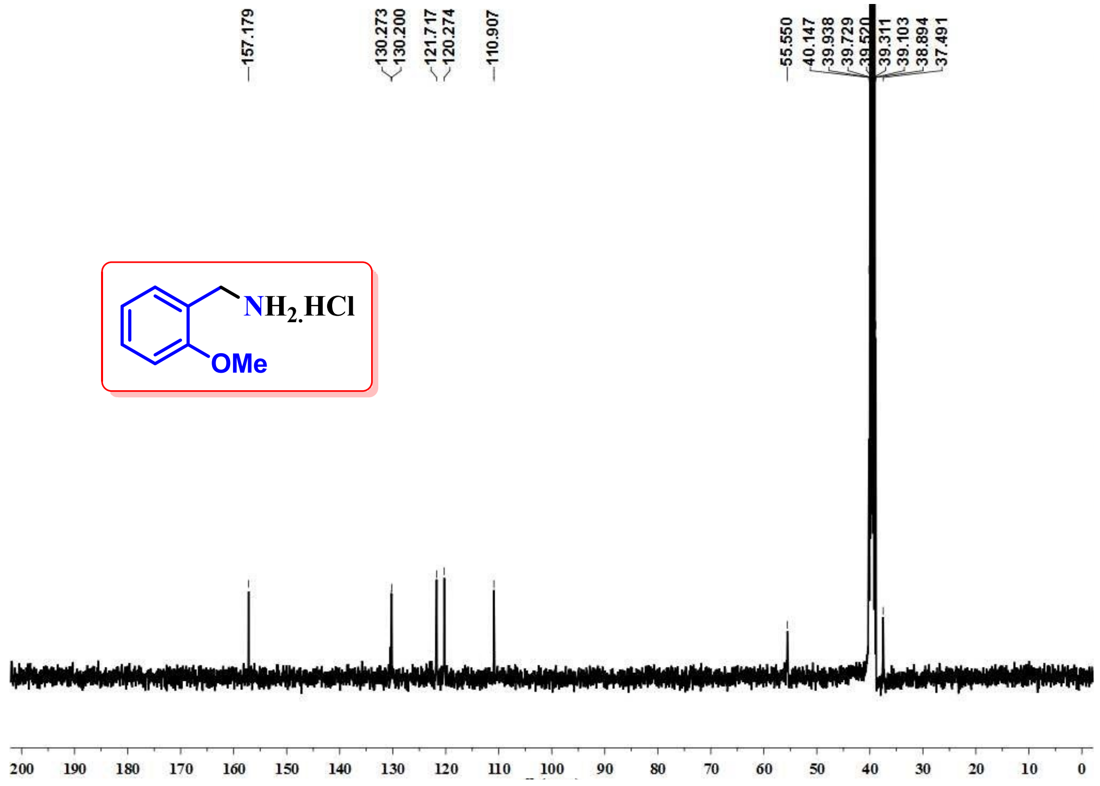

Figure S44. ^{13}C{^{1}H} NMR spectrum of (2-methoxyphenyl)methanamine hydrochloride (**3o)** in DMSO-d_6.

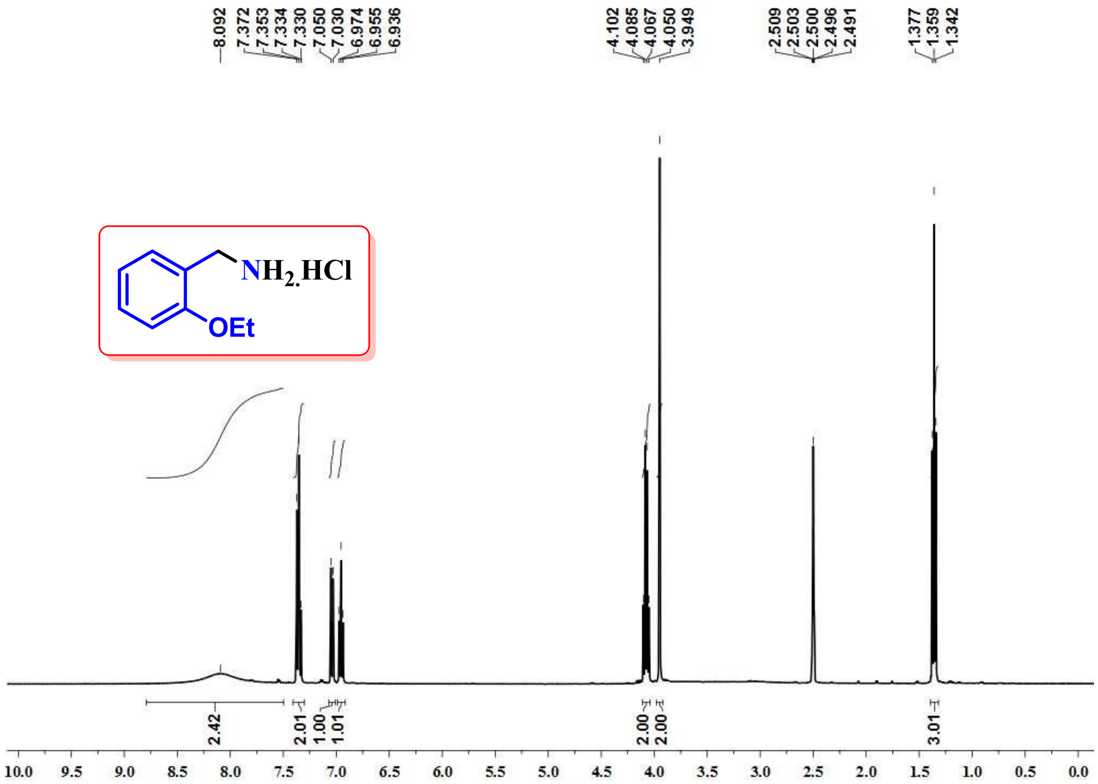

Figure S45. ^{1}H NMR spectrum of (2-ethoxyphenyl)methanamine hydrochloride (**3p)** in DMSO-d_6.

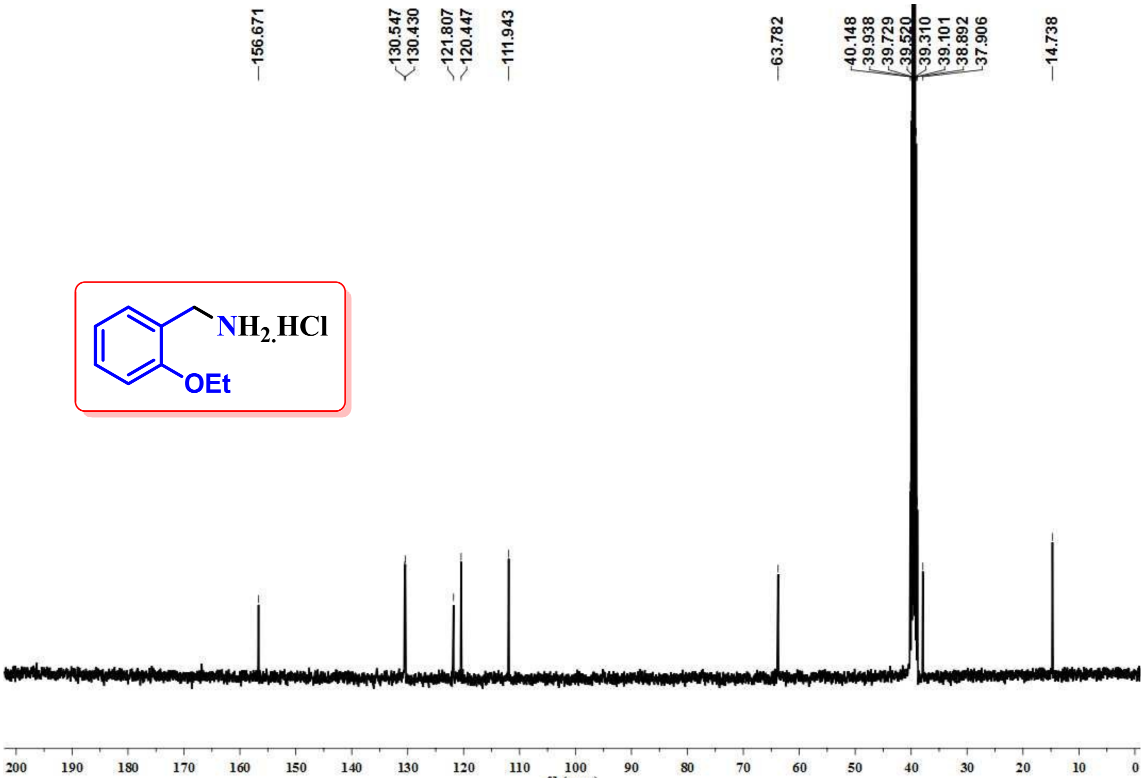

Figure S46. $^{13}C\{^{1}H\}$ NMR spectrum of (2-ethoxyphenyl)methanamine hydrochloride (**3p)** in DMSO-d_6.

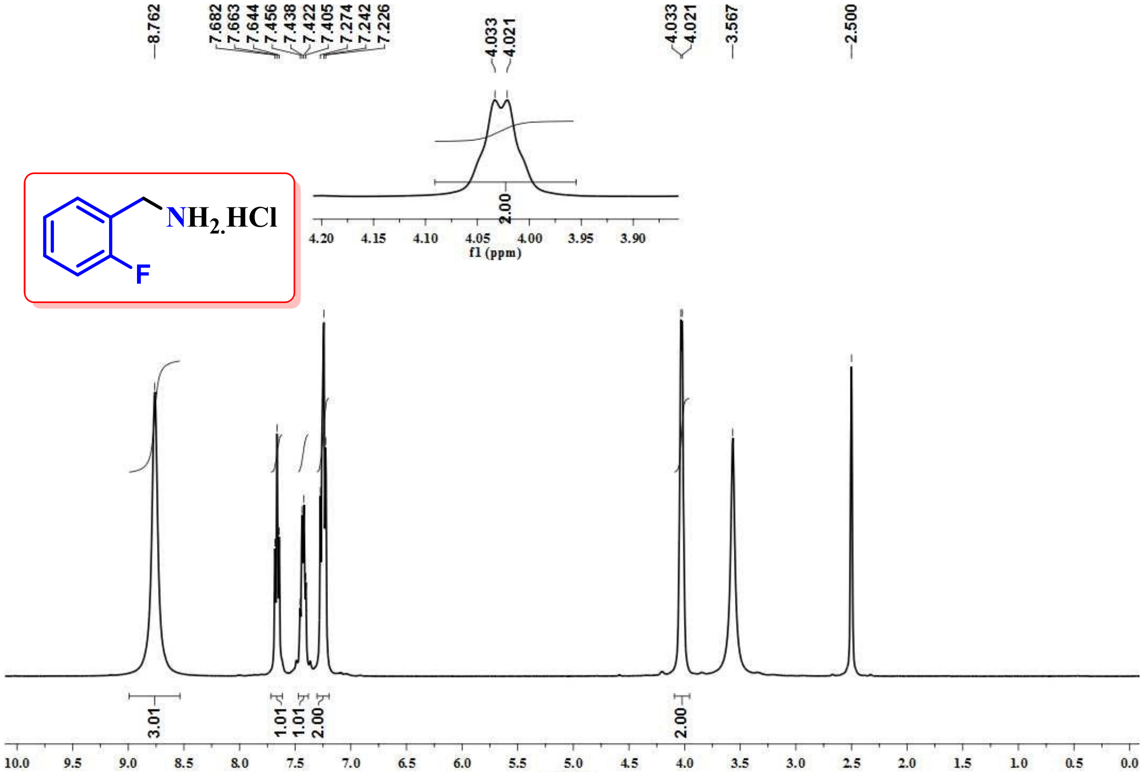

Figure S47. ^{1}H NMR spectrum of (2-fluorophenyl)methanamine hydrochloride (**3q)** in DMSO-d$_6$.

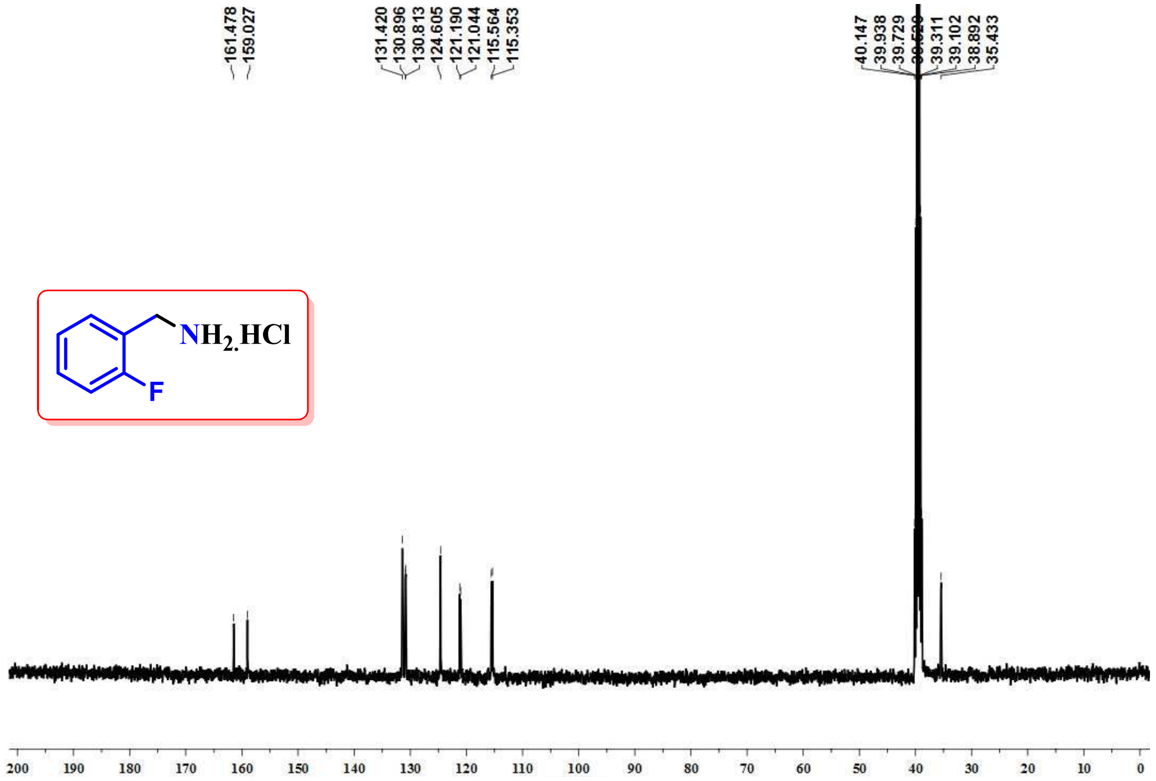

Figure S48. $^{13}C\{^{1}H\}$ NMR spectrum of (2-fluorophenyl)methanamine hydrochloride (**3q)** in DMSO-d$_6$.

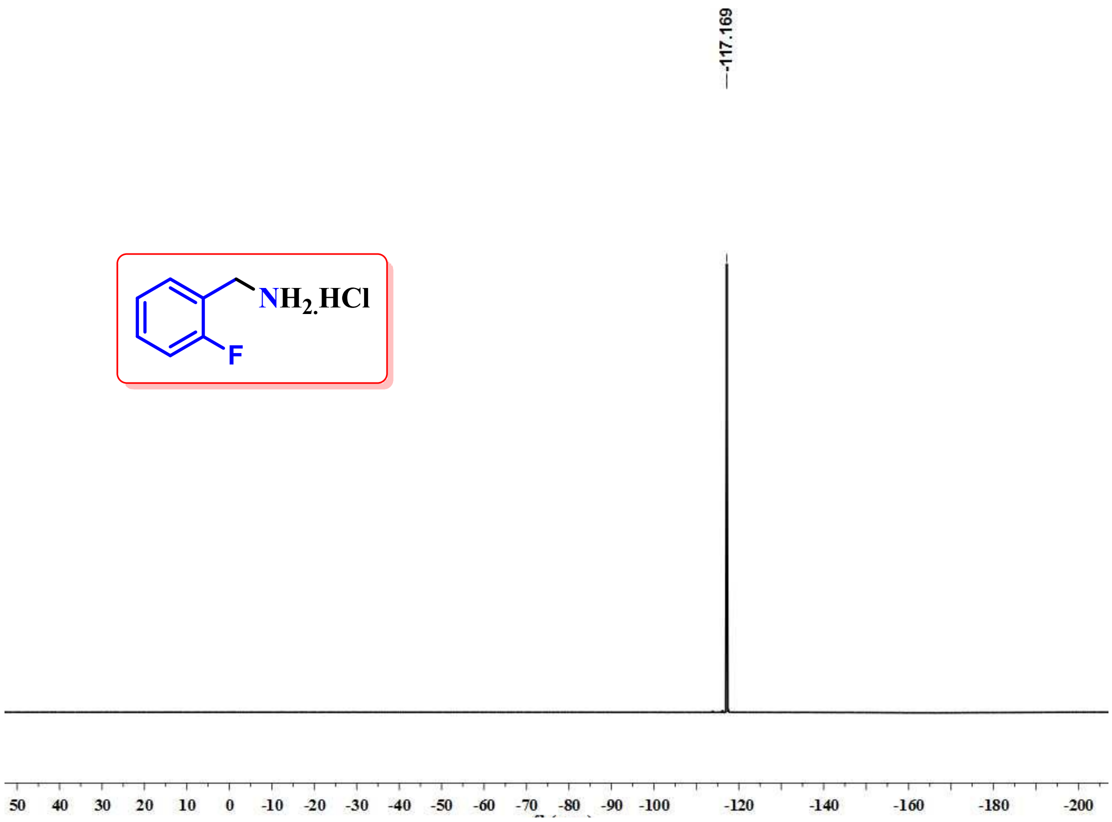

Figure S49. ^{19}F NMR spectrum of (2-fluorophenyl)methanamine hydrochloride (**3q)** in DMSO-d_6.

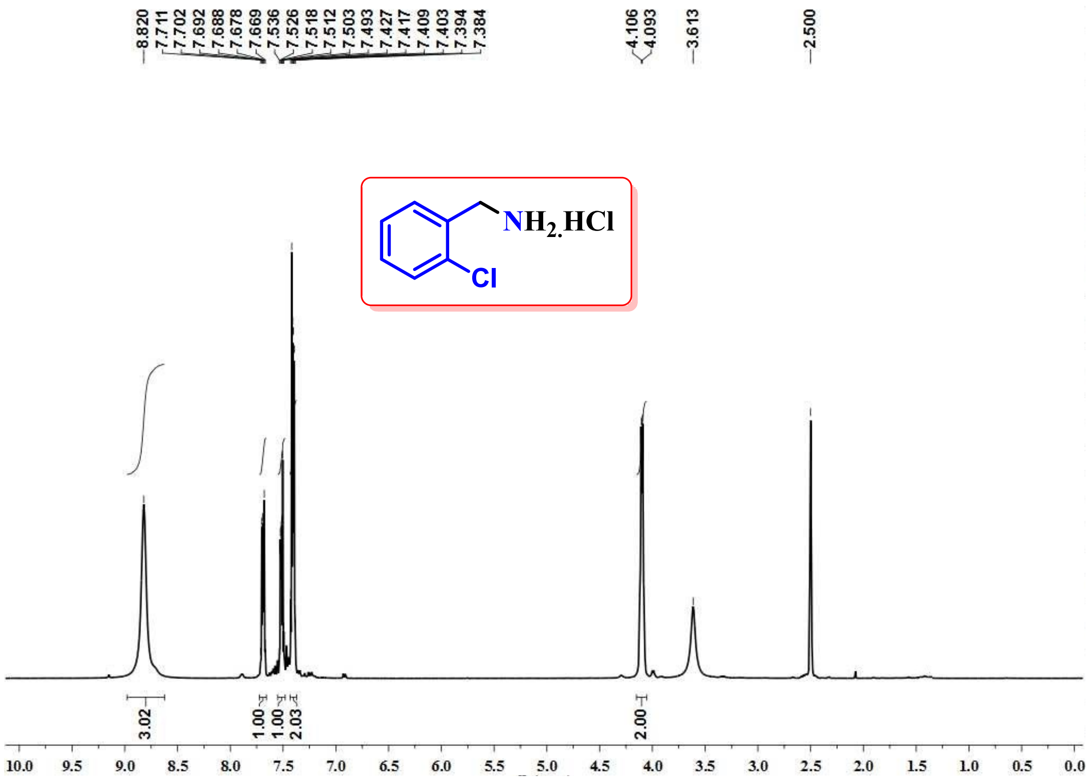

Figure S50. ^{1}H NMR spectrum of (2-chlorophenyl)methanamine hydrochloride (**3r)** in DMSO-d_6.

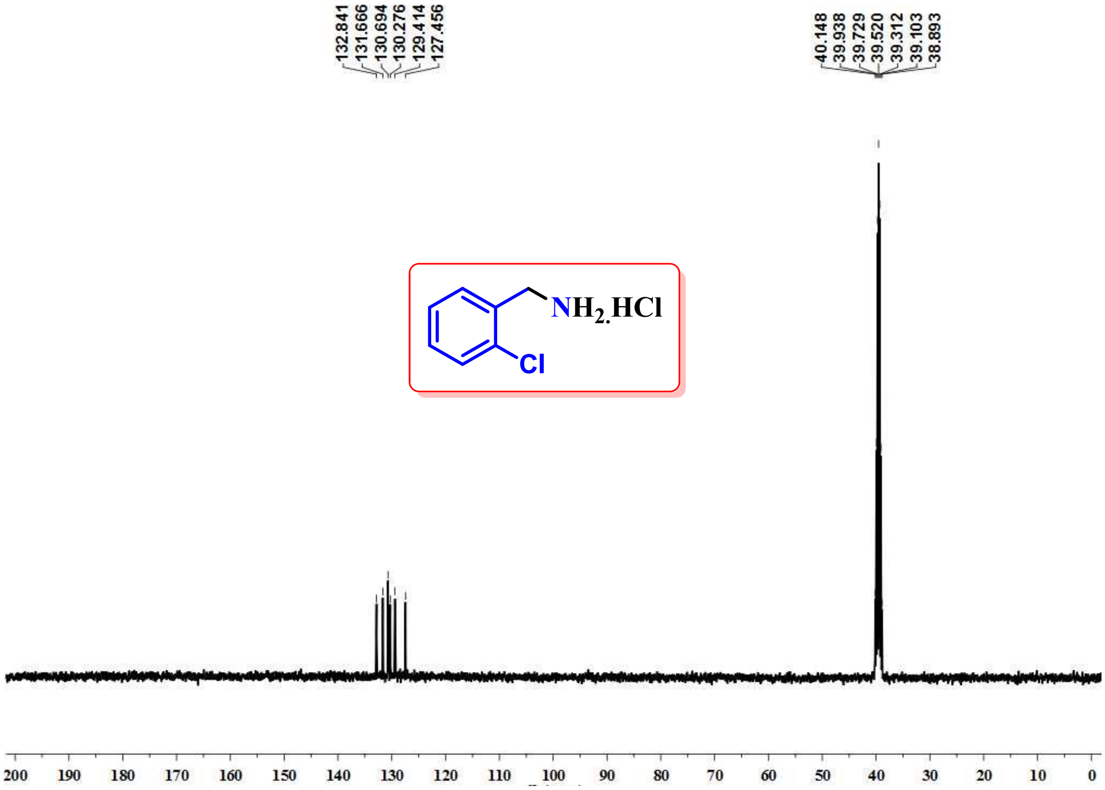

Figure S51. ^{13}C{^{1}H} NMR spectrum of (2-chlorophenyl)methanamine hydrochloride (**3r)** in DMSO-d_6.

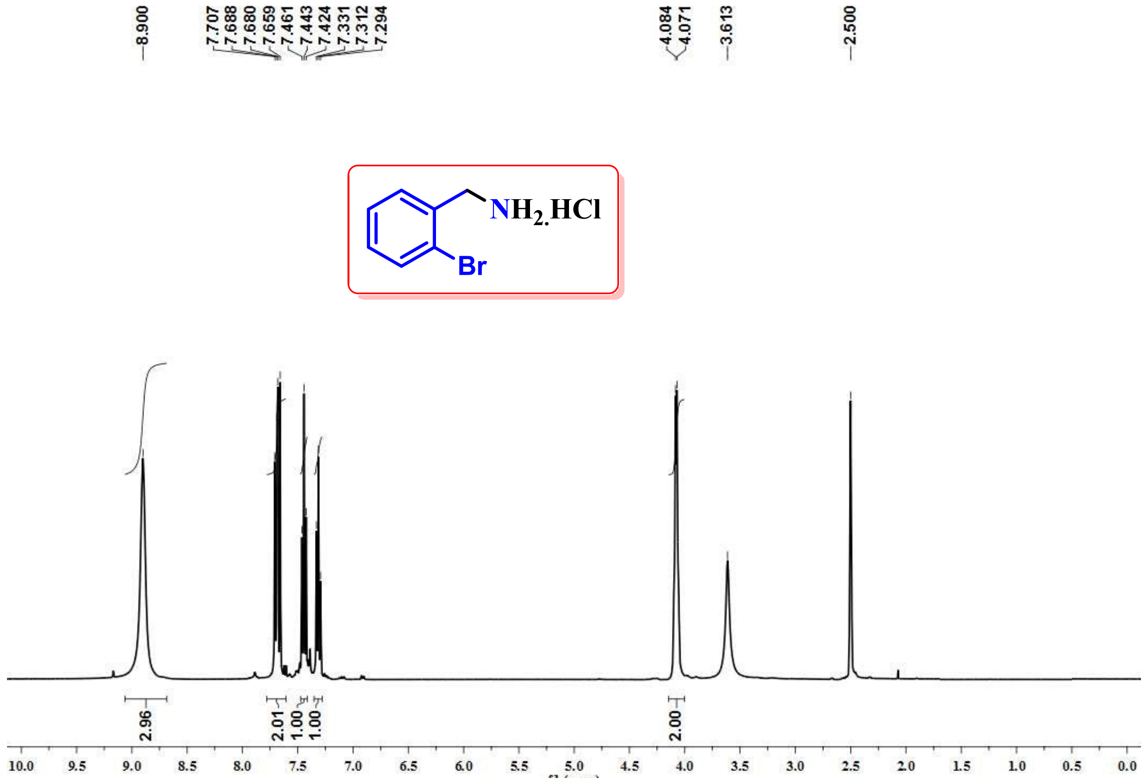

Figure S52. ^{1}H NMR spectrum of (2-bromophenyl)methanamine hydrochloride (**3s**) in DMSO-d$_6$.

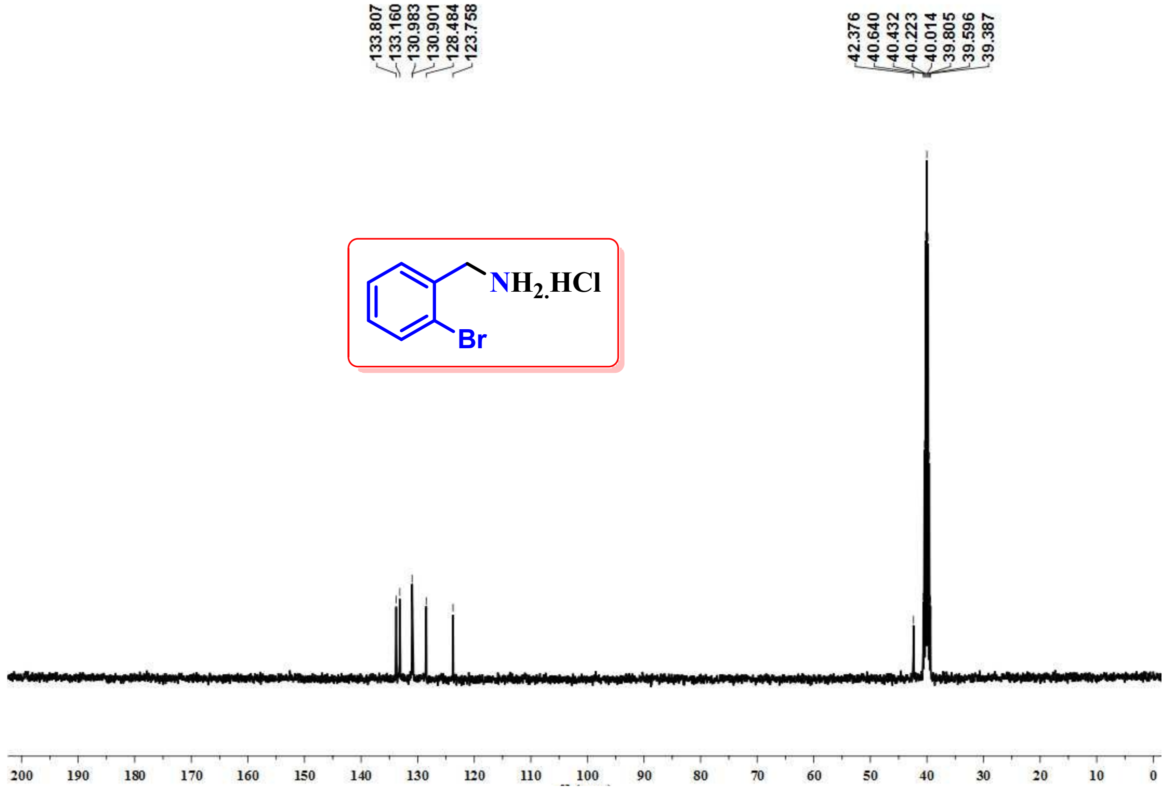

Figure S53. ^{13}C{^{1}H} NMR spectrum of (2-bromophenyl)methanamine hydrochloride (**3s**) in DMSO-d$_6$.

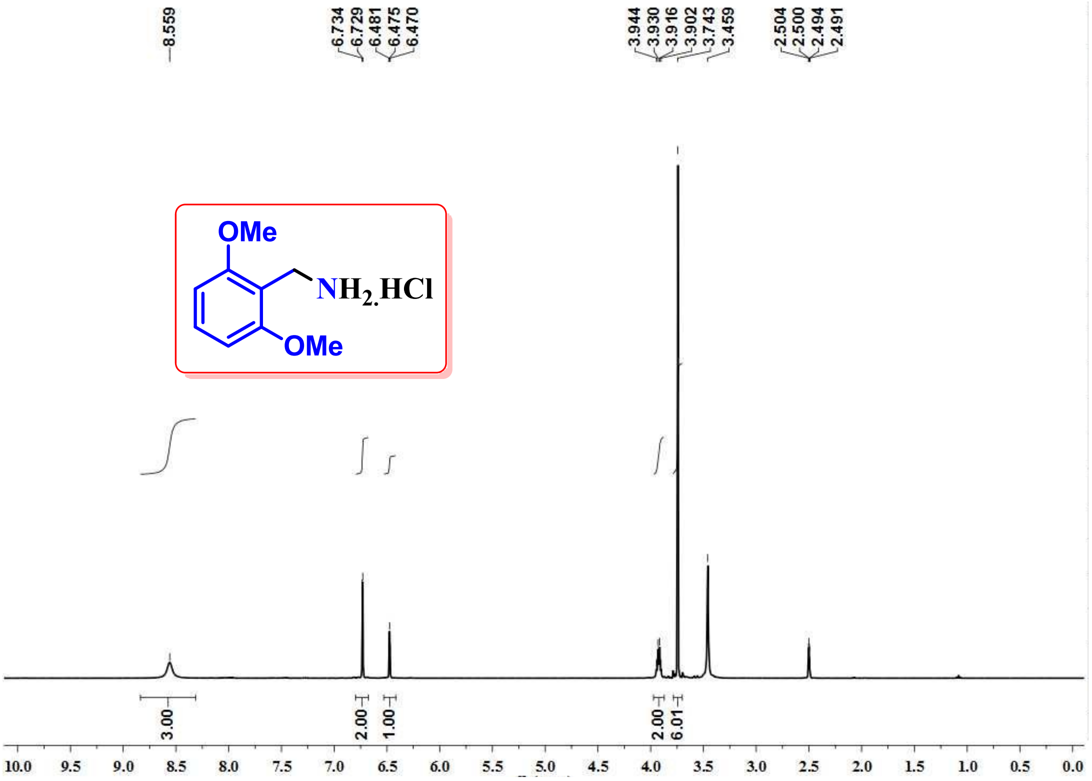

Figure S54. ^{1}H NMR spectrum of (2,6-dimethoxyphenyl)methanamine hydrochloride (**3t)** in DMSO-d_6.

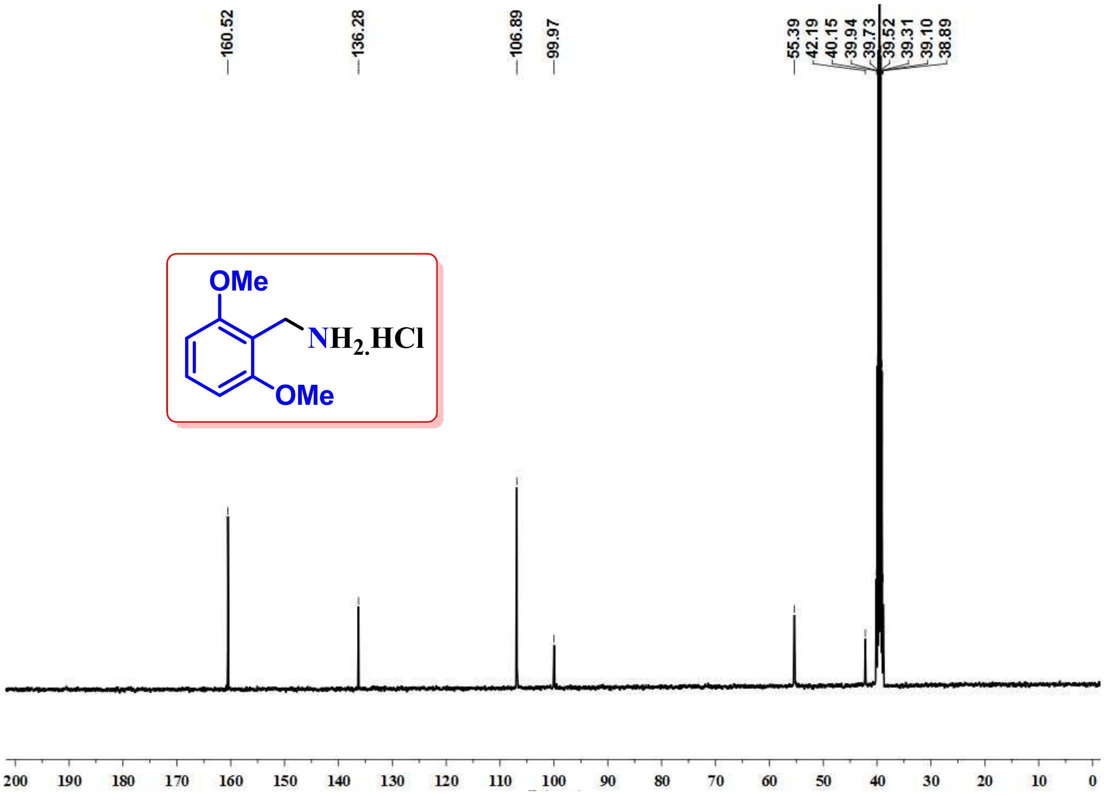

Figure S55. $^{13}C\{^{1}H\}$ NMR spectrum of (2,6-dimethoxyphenyl)methanamine hydrochloride (**3t)** in DMSO-d_6.

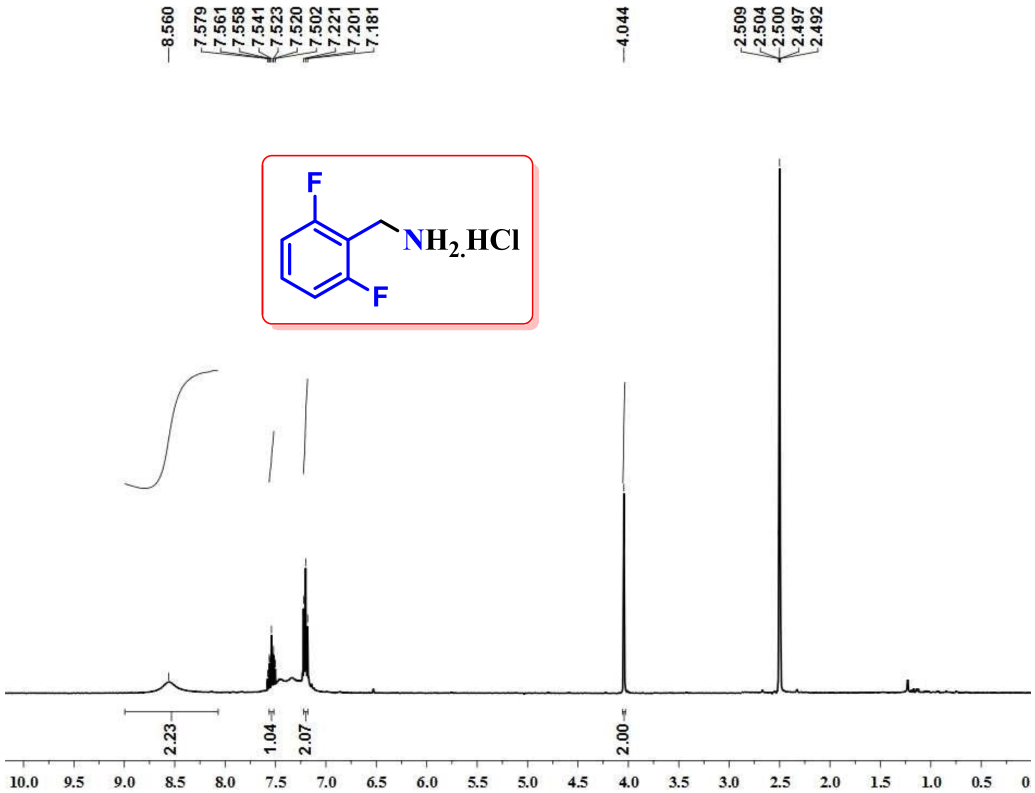

Figure S56. ^{1}H NMR spectrum of (2,6-difluorophenyl)methanamine hydrochloride (**3u)** in DMSO-d_6.

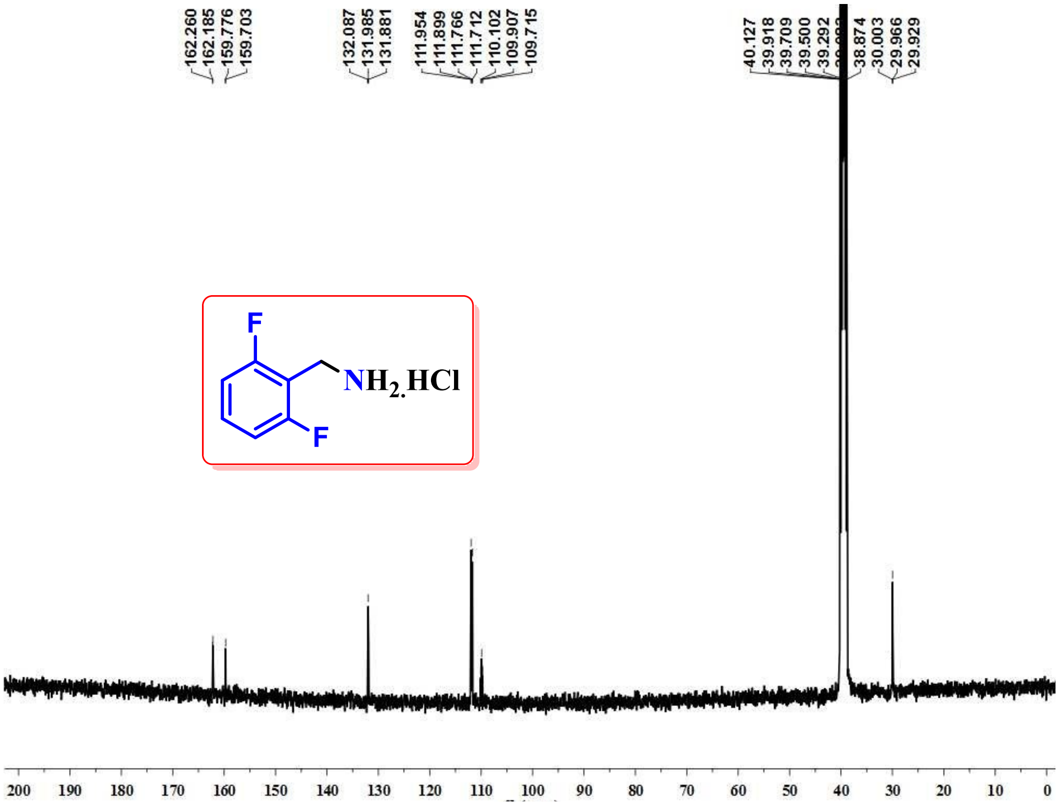

Figure S57. ^{13}C{^{1}H} NMR spectrum of (2,6-difluorophenyl)methanamine hydrochloride (**3u)** in DMSO-d_6.

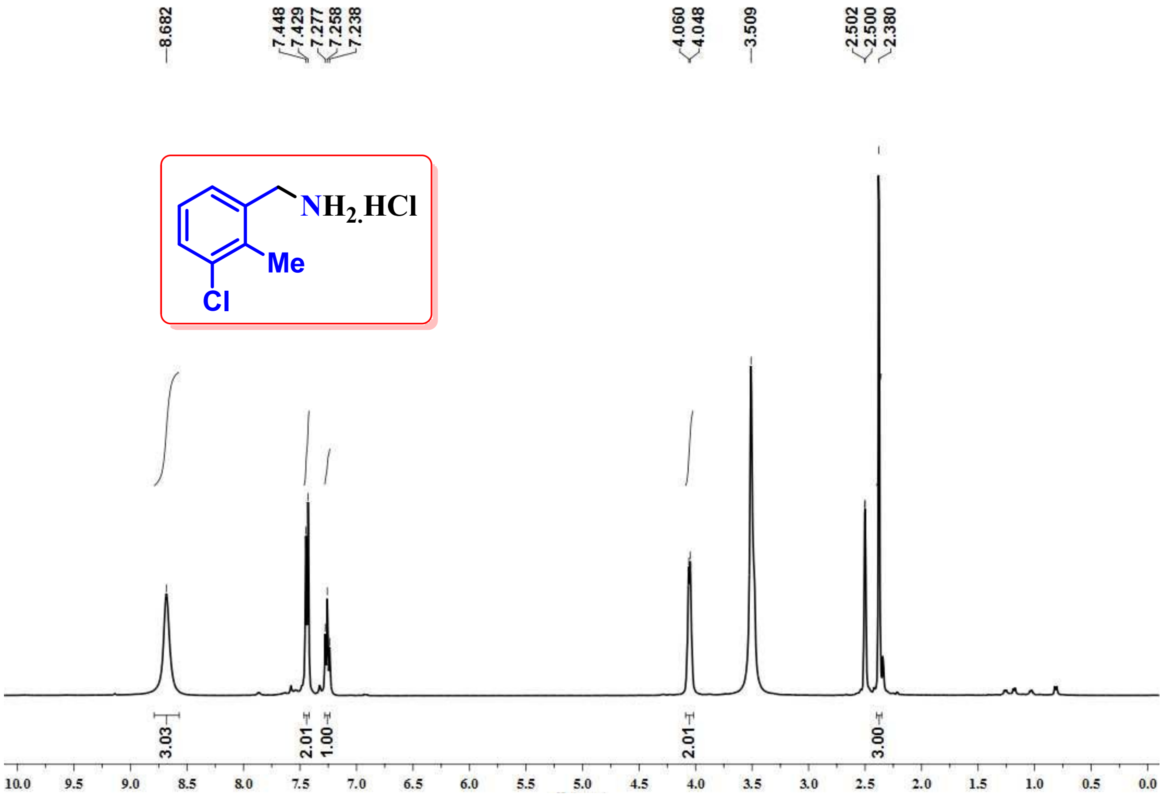

Figure S58. ^{1}H NMR spectrum of (3-chloro-2-methylphenyl)methanamine hydrochloride (**3v**) in DMSO-d_6.

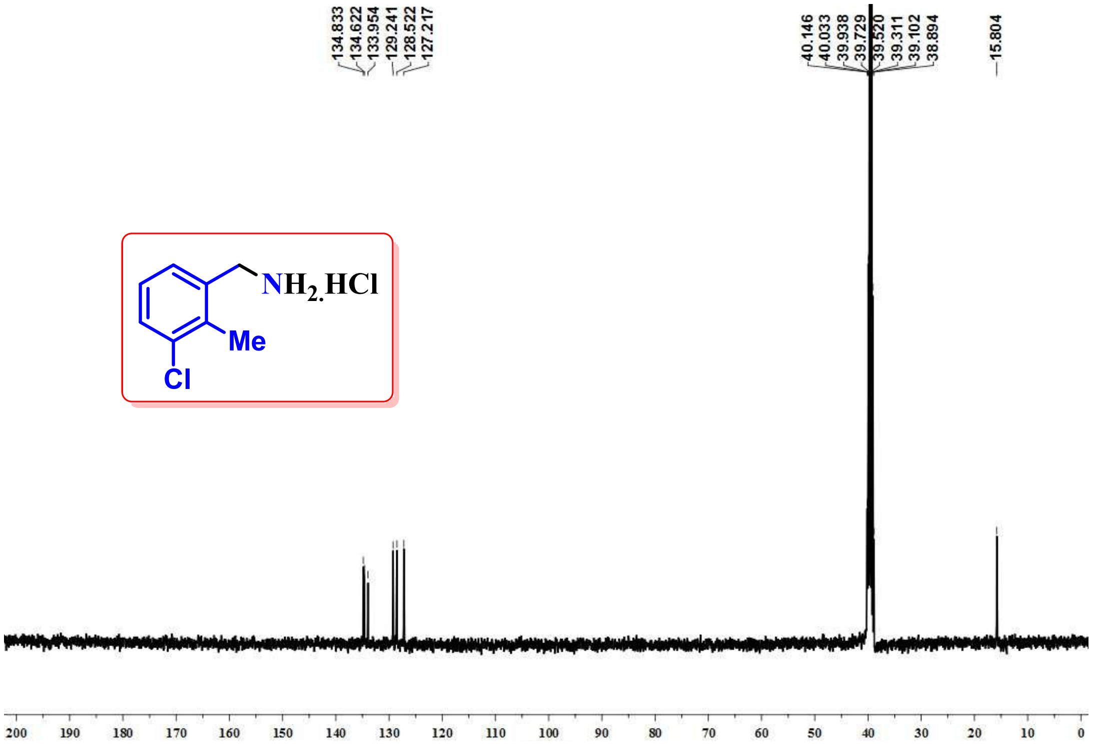

Figure S59. $^{13}C\{^{1}H\}$ NMR spectrum of (3-chloro-2-methylphenyl)methanamine hydrochloride (**3v**) in DMSO-d_6.

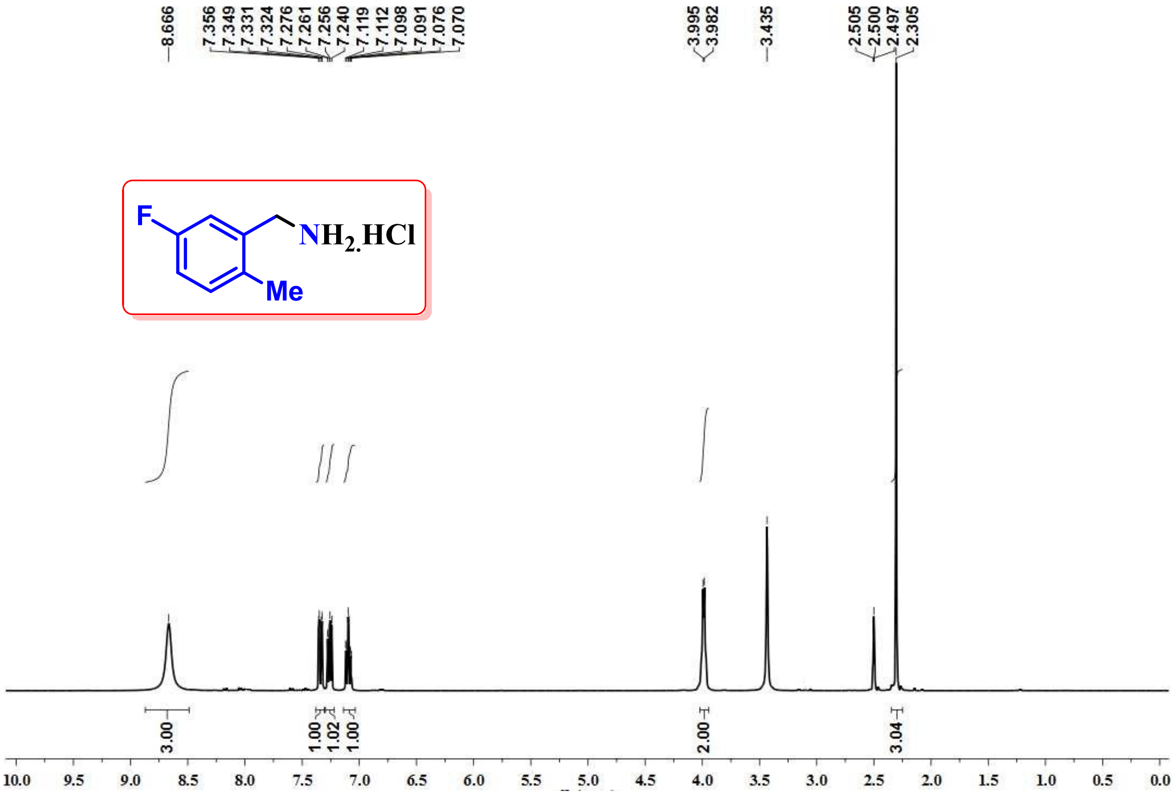

Figure S60. ^{1}H NMR spectrum of (5-fluoro-2-methylphenyl)methanamine hydrochloride (**3w)** in DMSO-d_6.

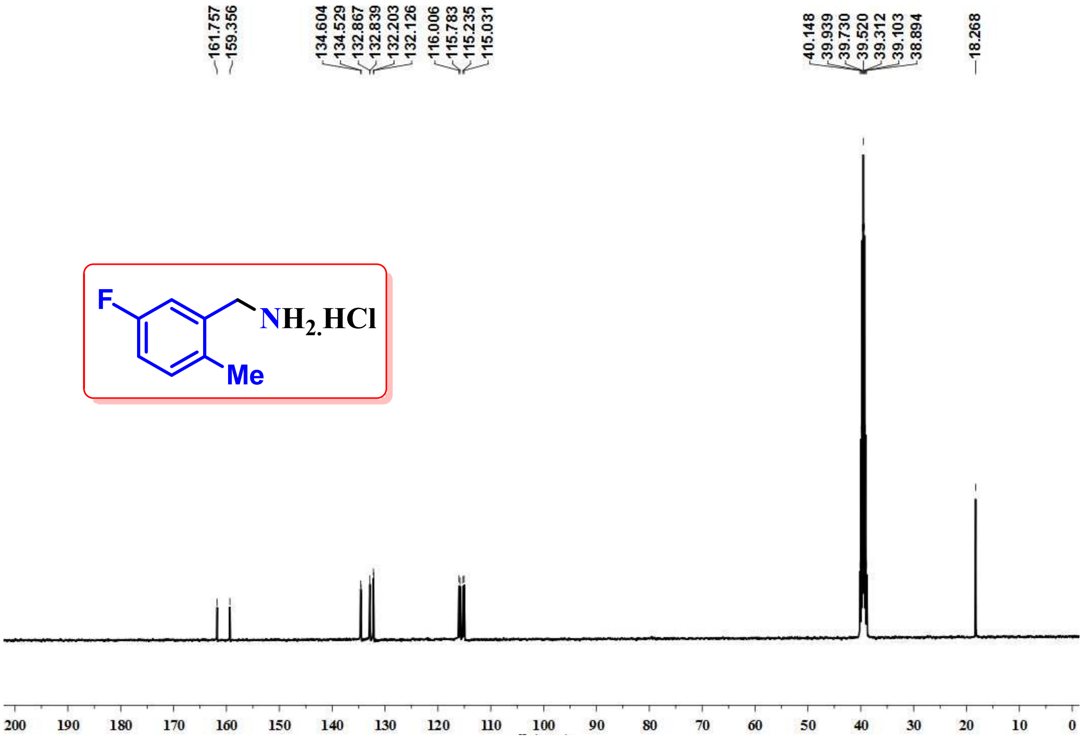

Figure S61. ^{13}C{^{1}H} NMR spectrum of (5-fluoro-2-methylphenyl)methanamine hydrochloride (**3w)** in DMSO-d_6.

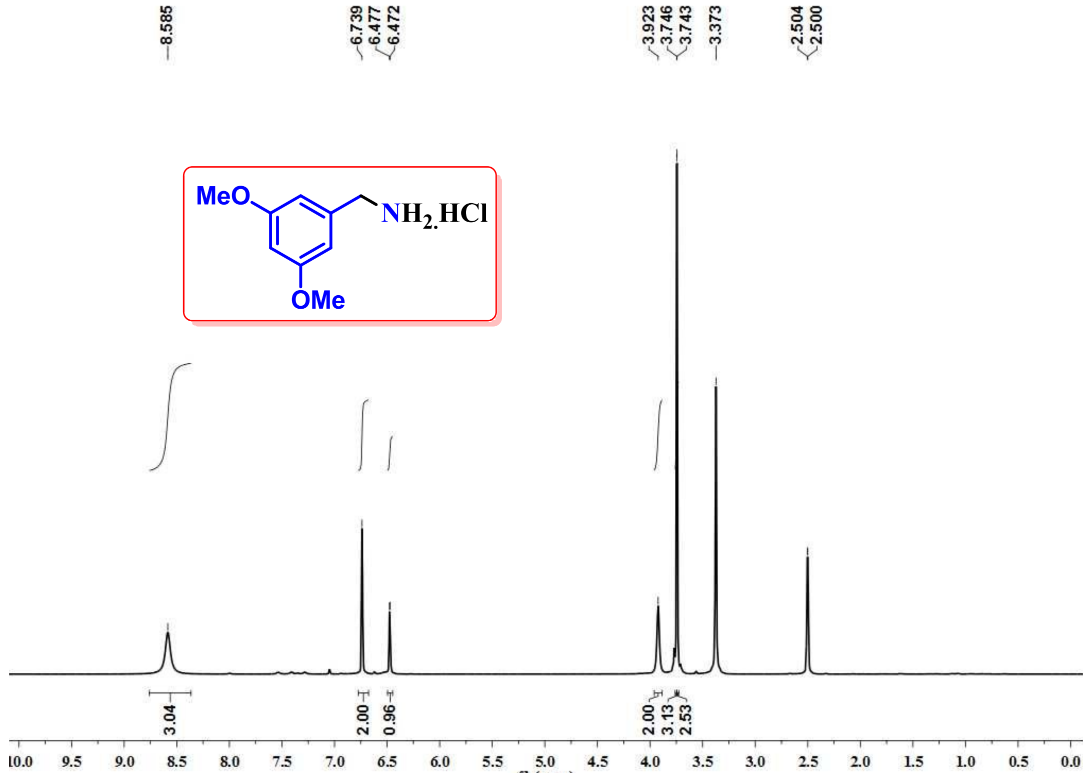

Figure S62. ^{1}H NMR spectrum of (3,5-dimethoxyphenyl)methanamine hydrochloride (**3x)** in DMSO-d_6.

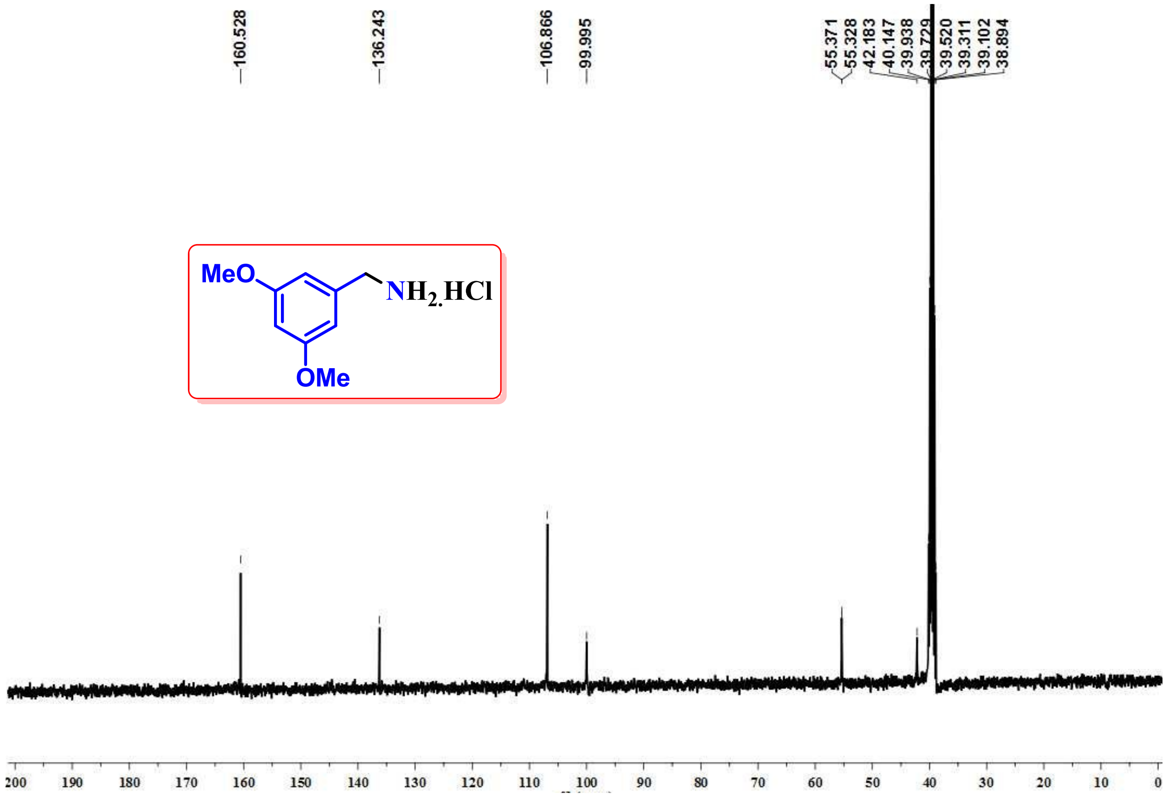

Figure S63. ^{13}C{^{1}H} NMR spectrum of (3,5-dimethoxyphenyl)methanamine hydrochloride (**3x)** in DMSO-d_6.

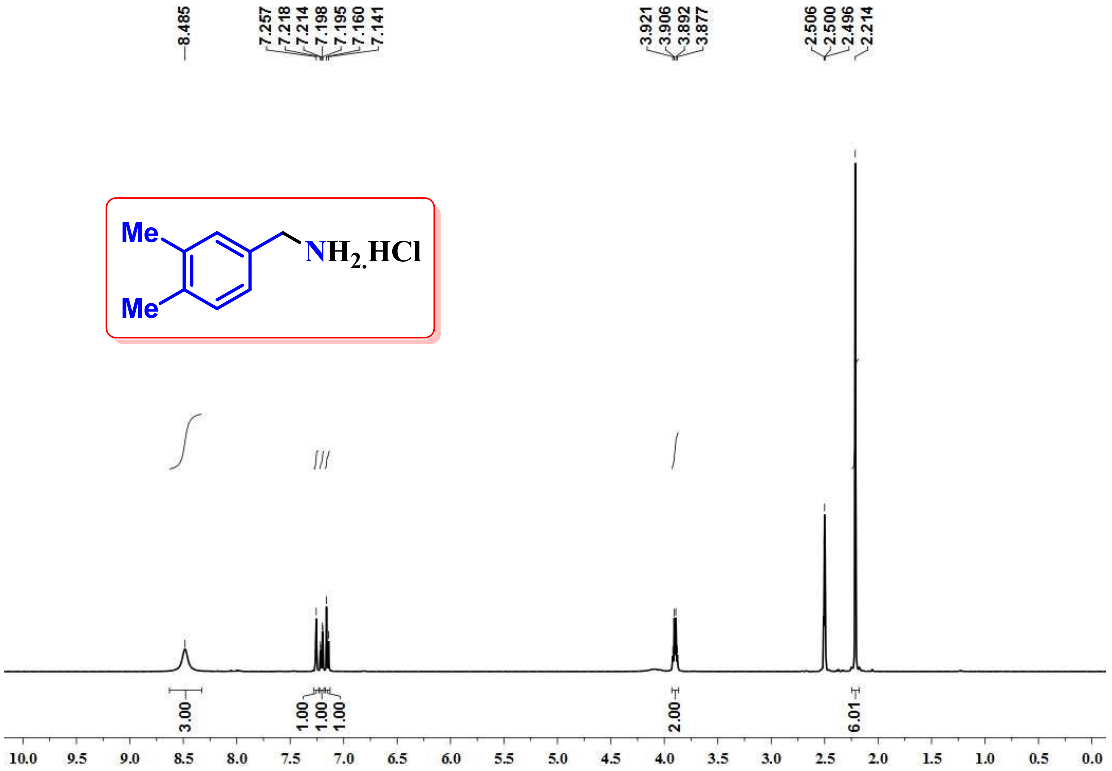

Figure S64. ^{1}H NMR spectrum of (3,4-dimethylphenyl)methanamine hydrochloride (**3y)** in DMSO-d_6.

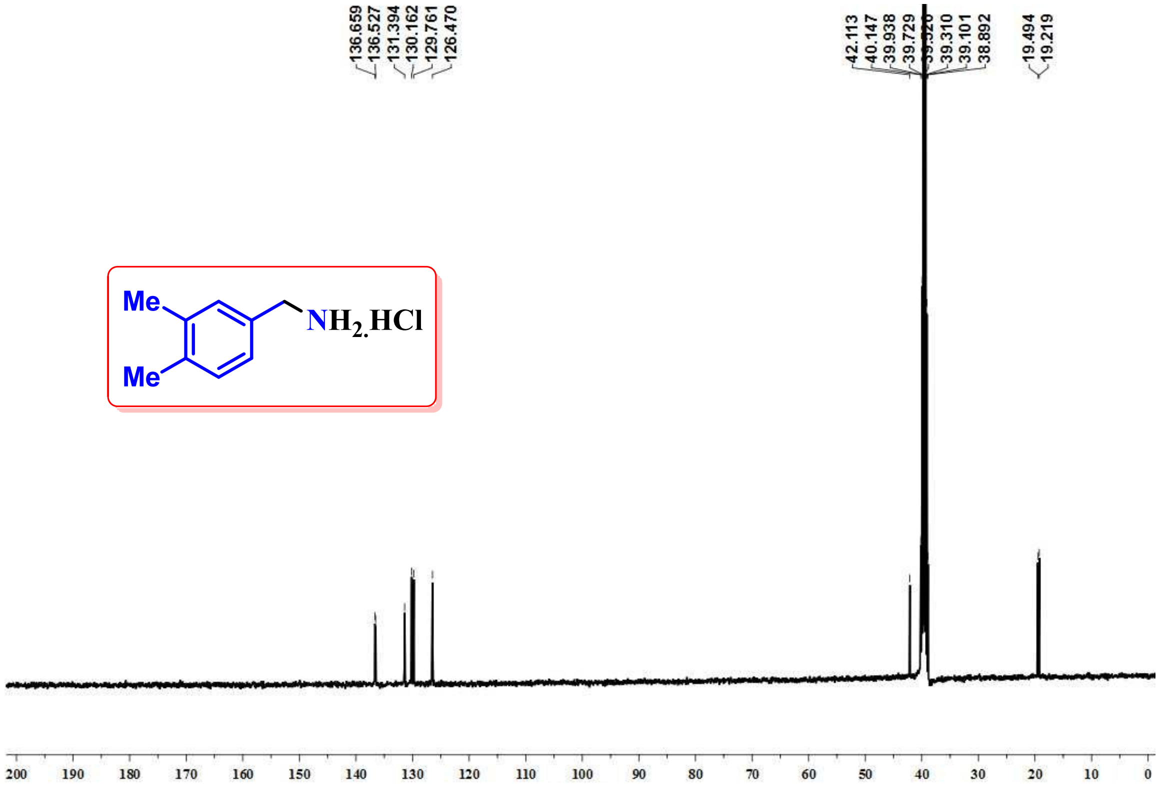

Figure S65. $^{13}C\{^{1}H\}$ NMR spectrum of (3,4-dimethylphenyl)methanamine hydrochloride (**3y)** in DMSO-d_6.

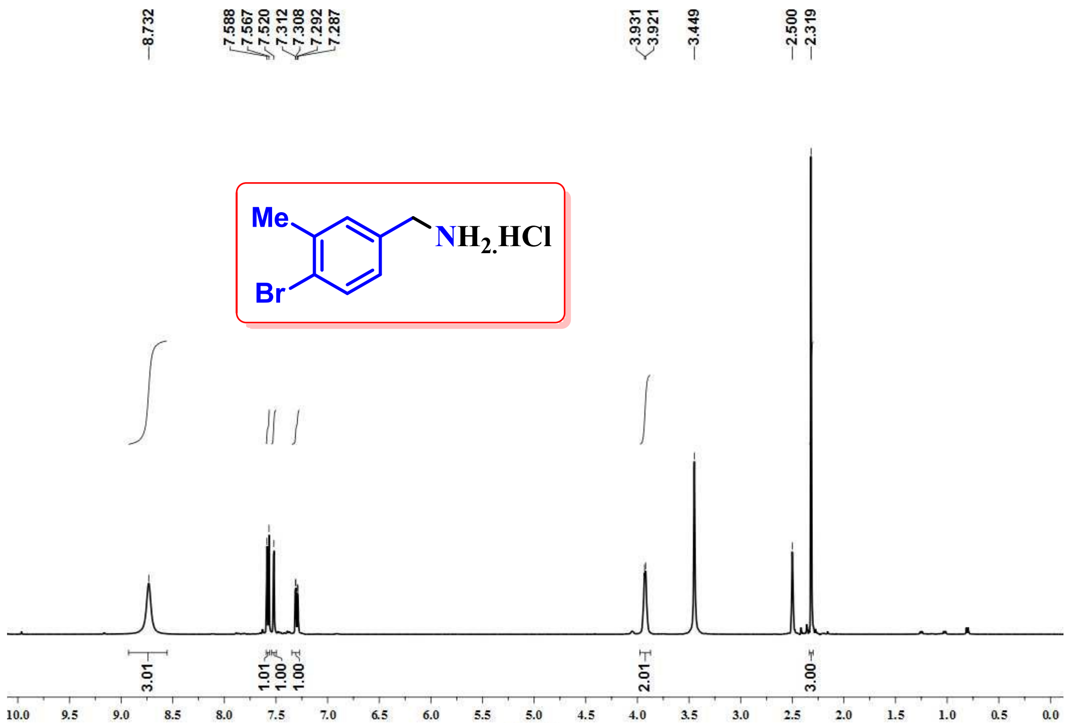

Figure S66. ^{1}H NMR spectrum of (4-bromo-3-methylphenyl)methanamine hydrochloride (**3z)** in DMSO-d_6.

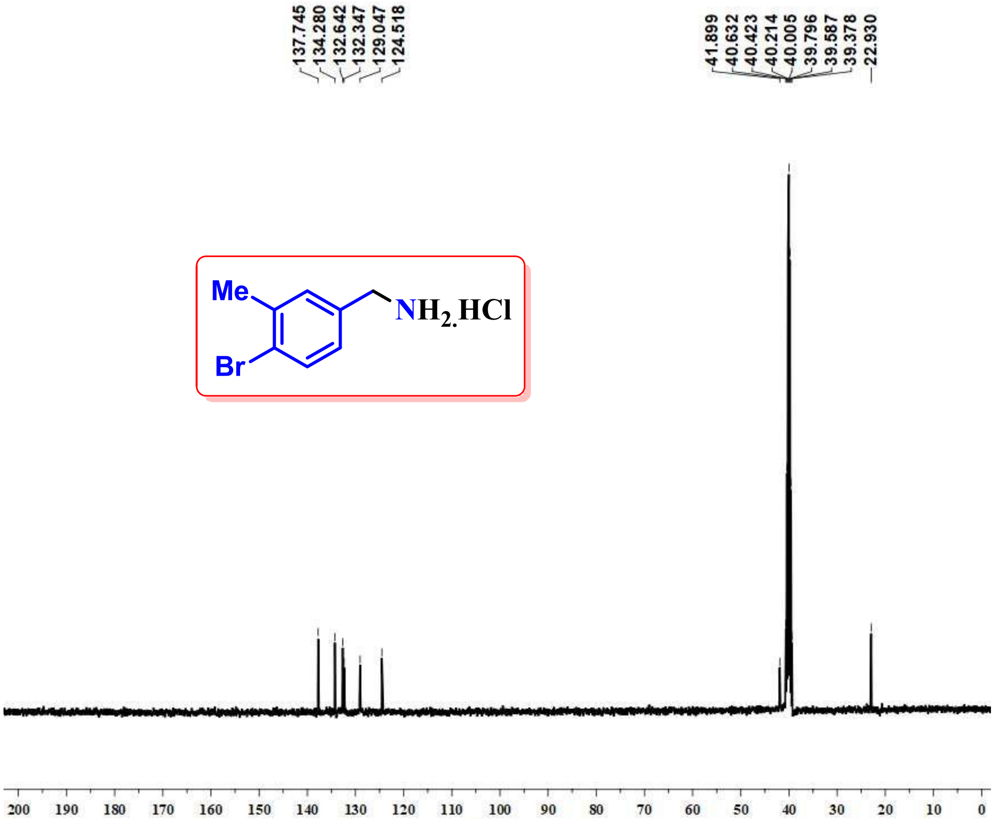

Figure S67. $^{13}C\{^1H\}$ NMR spectrum of (4-bromo-3-methylphenyl)methanamine hydrochloride (**3z)** in DMSO-d_6.

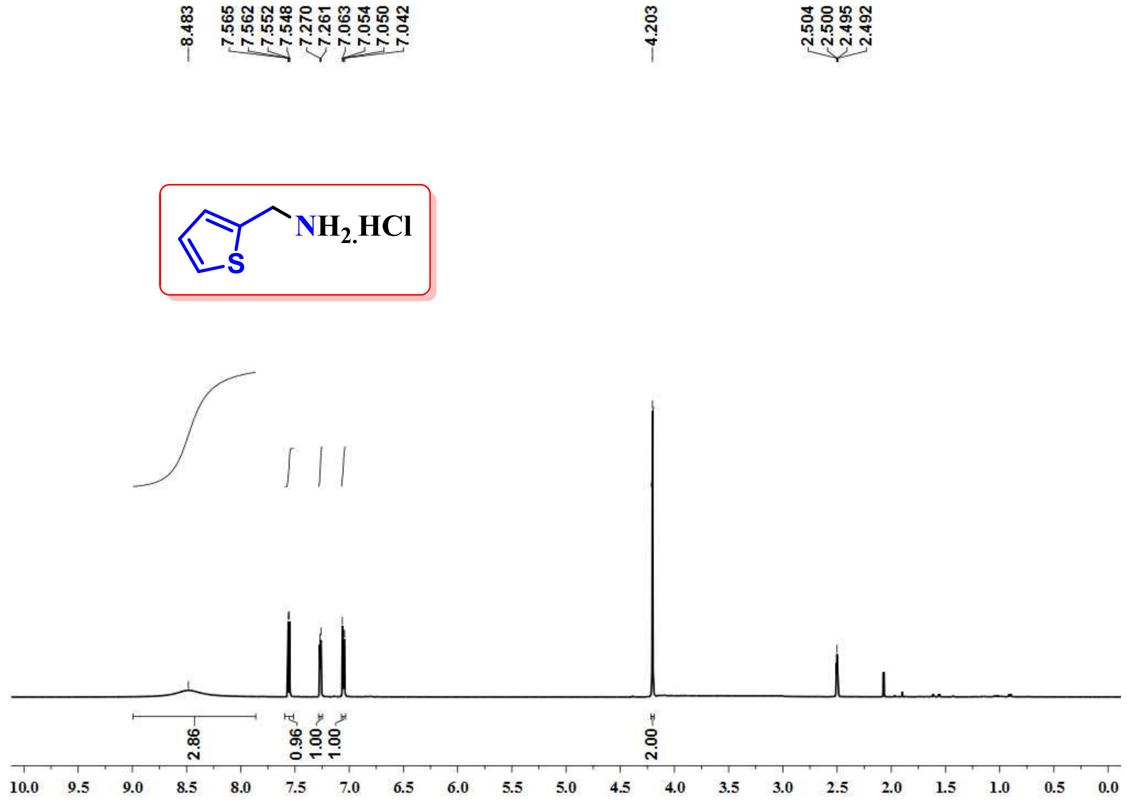

Figure S68. ^{1}H NMR spectrum of thiophen-2-ylmethanamine hydrochloride (**3aa)** in DMSO-d_6.

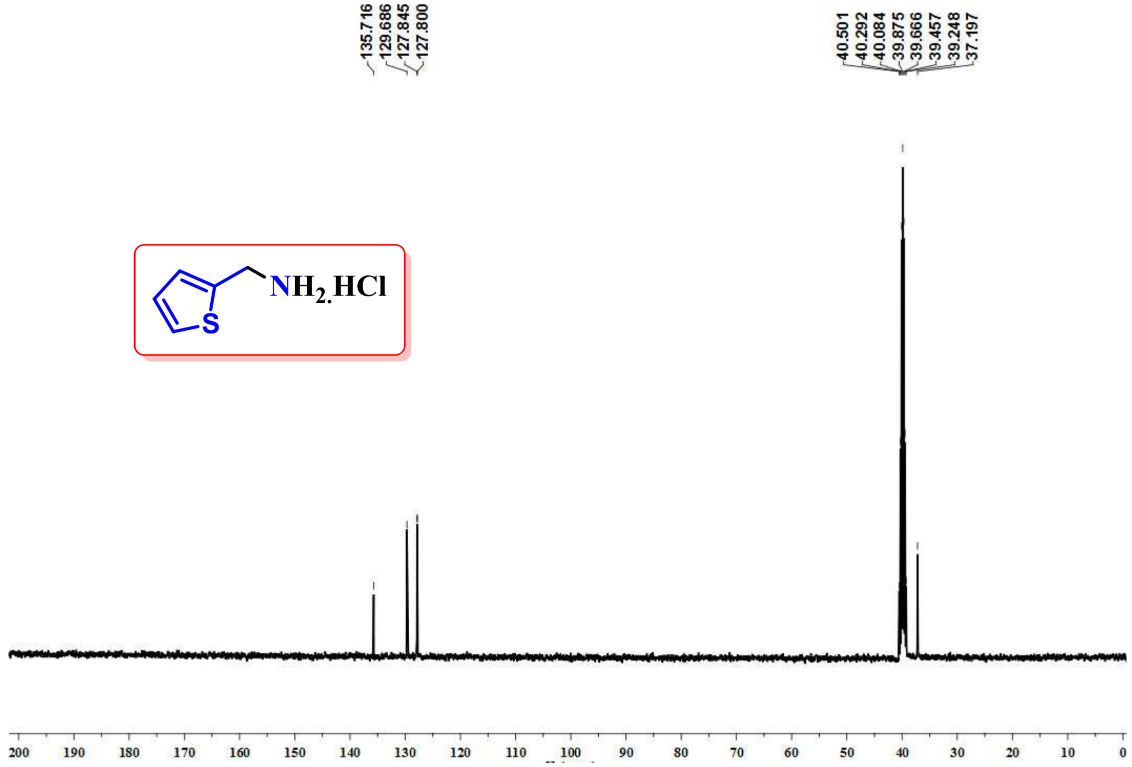

Figure S69. $^{13}C\{^{1}H\}$ NMR spectrum of thiophen-2-ylmethanamine hydrochloride (**3aa)** in DMSO-d_6.

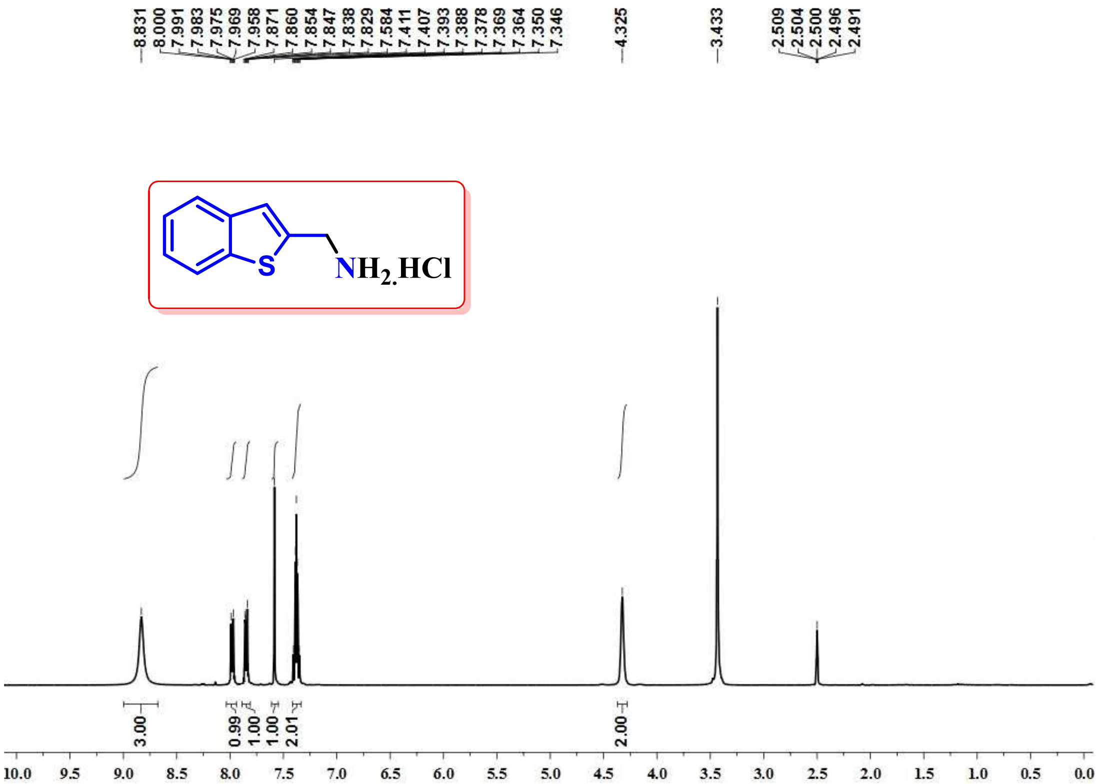

Figure S70. ^{1}H NMR spectrum of benzothiophene-2-ylmethanamine hydrochloride (**3ab)** in DMSO-d_6.

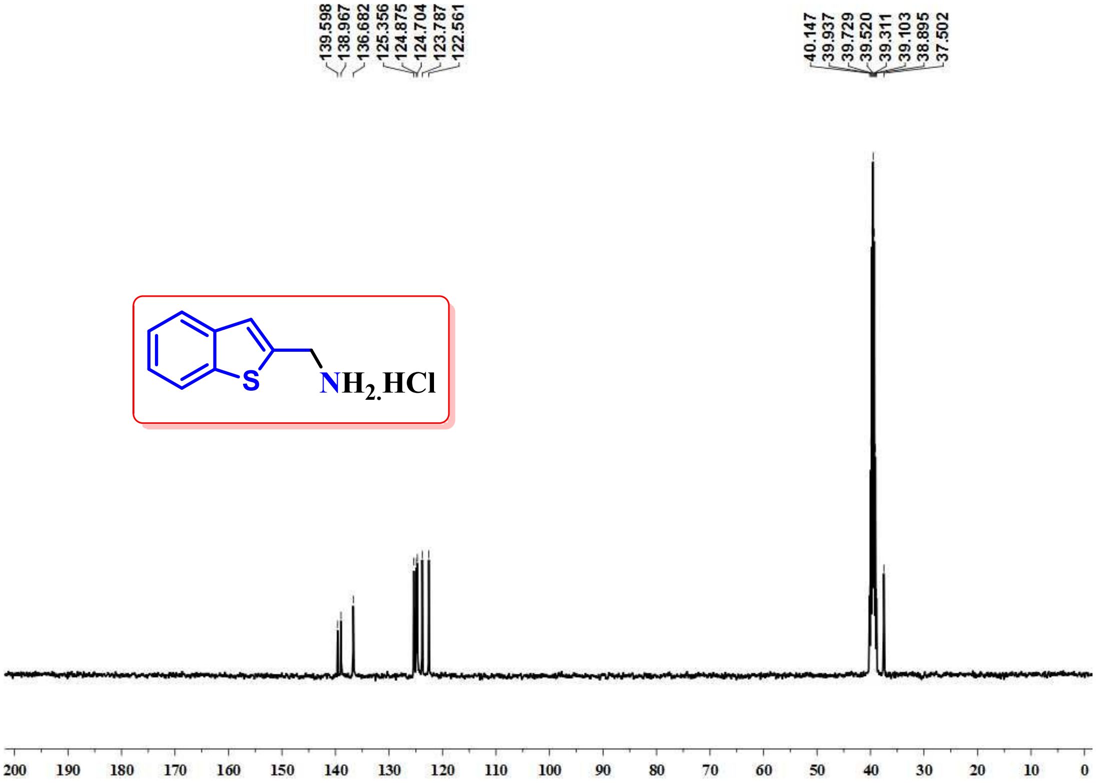

Figure S71. $^{13}C\{^{1}H\}$ NMR spectrum of benzothiophene-2-ylmethanamine hydrochloride (**3ab)** in DMSO-d_6.

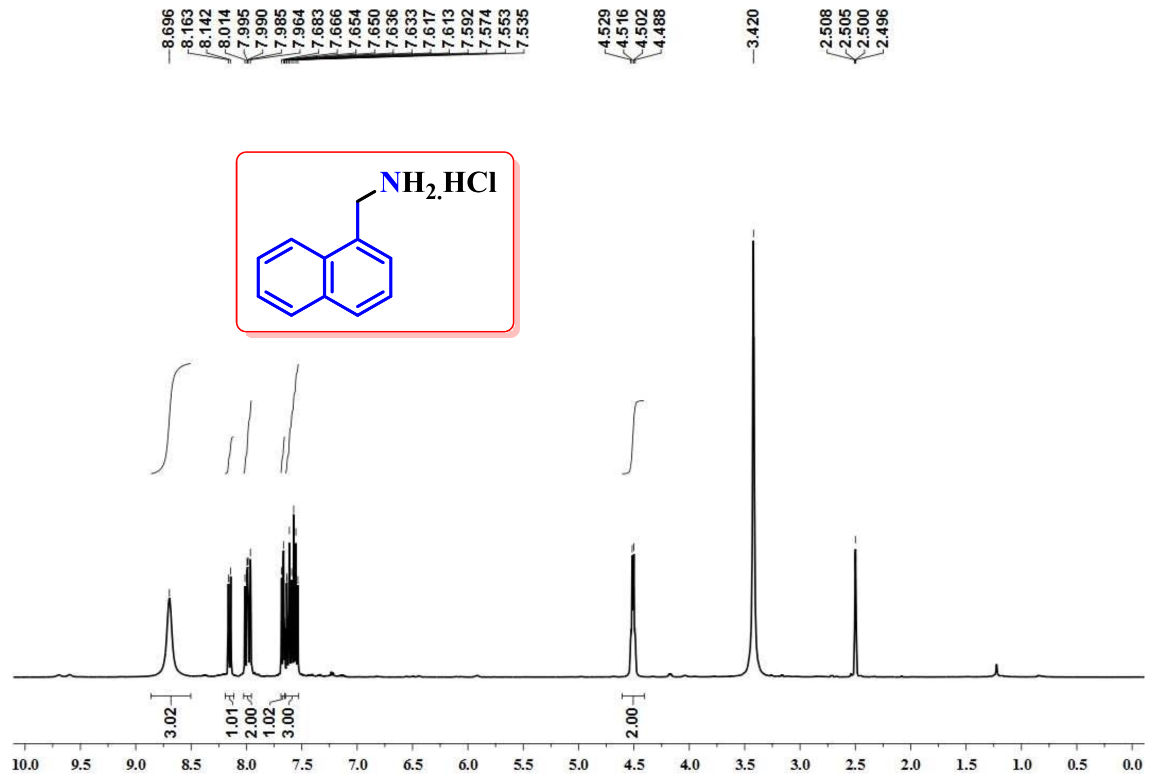

Figure S72. ^{1}H NMR spectrum of naphthalen-1-ylmethanamine hydrochloride (**3ad)** in DMSO-d_6.

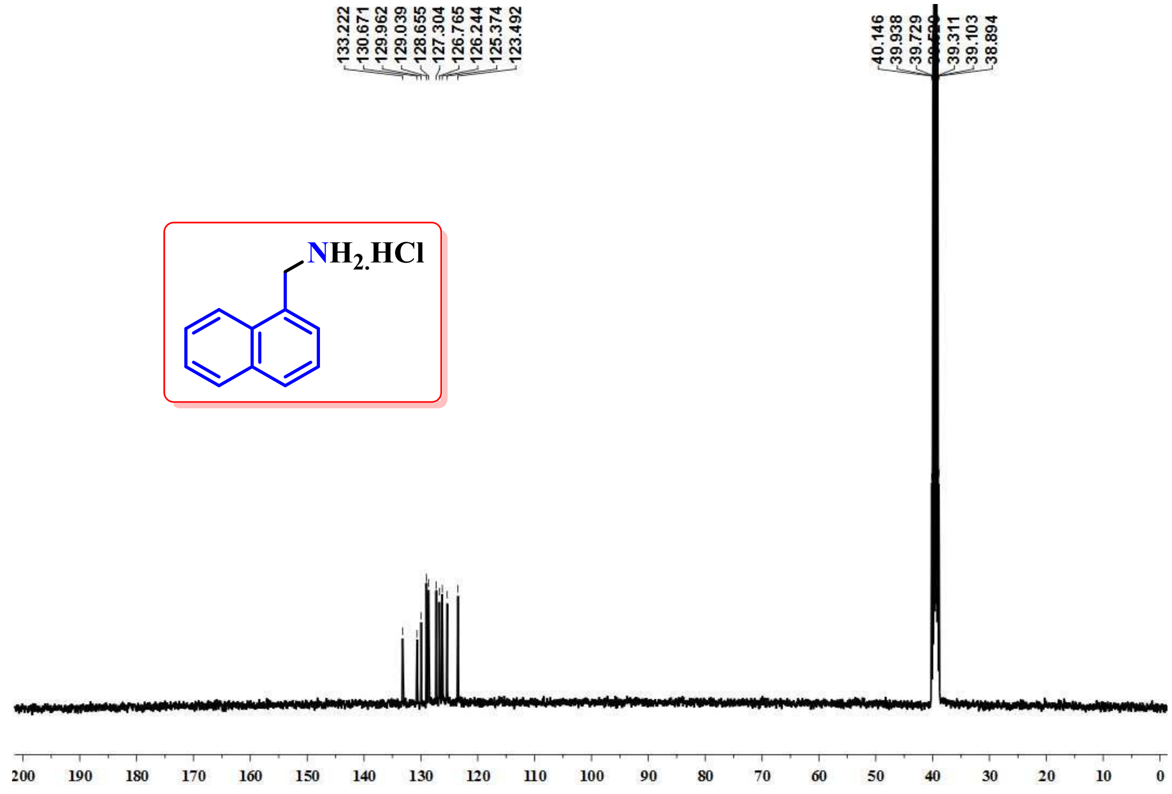

Figure S73. $^{13}C\{^{1}H\}$ NMR spectrum of naphthalen-1-ylmethanamine hydrochloride (**3ad)** in DMSO-d_6.

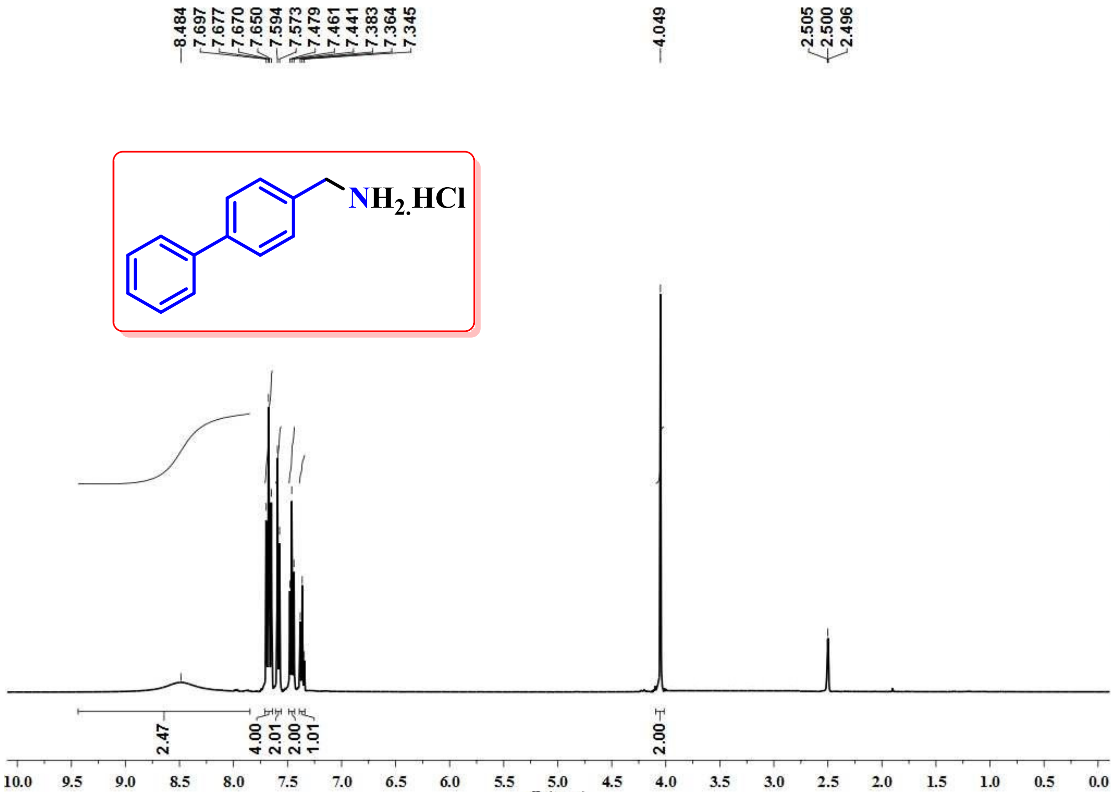

Figure S74. ^{1}H NMR spectrum of [1,1'-biphenyl]-4-ylmethanamine hydrochloride (**3ae)** in DMSO-d_6.

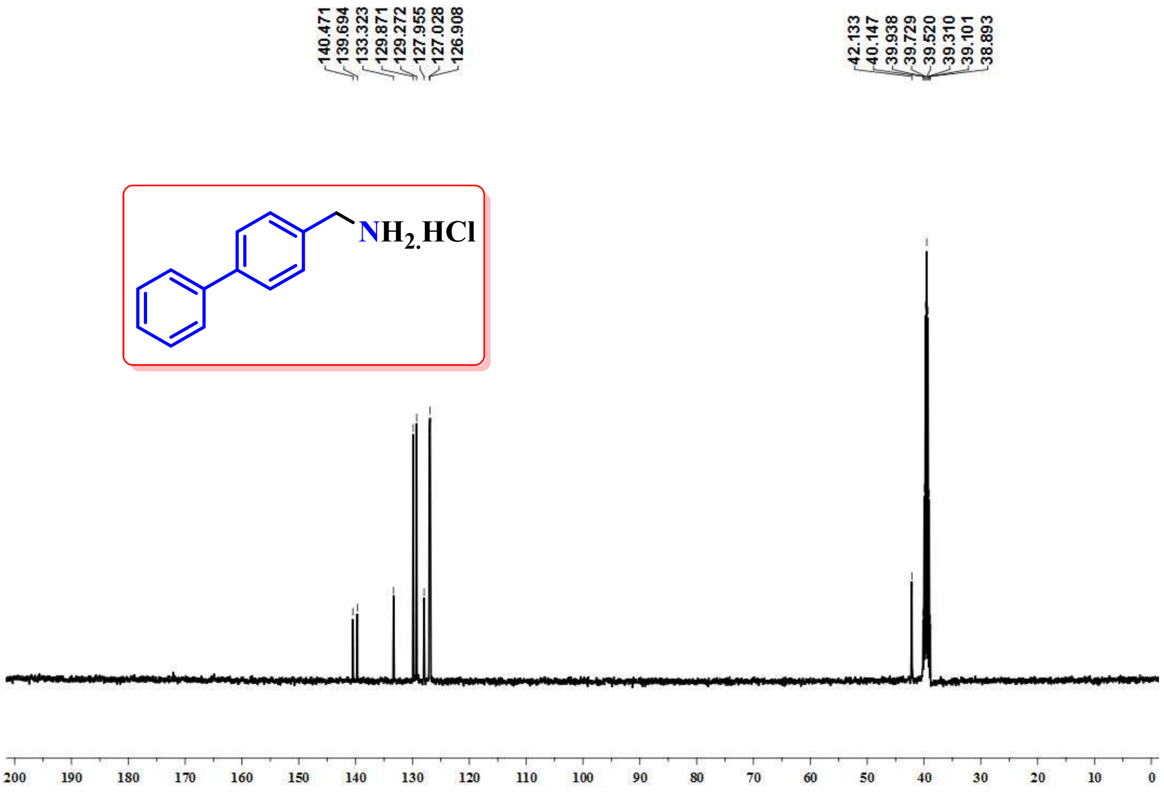

Figure S75. $^{13}C\{^1H\}$ NMR spectrum of [1,1'-biphenyl]-4-ylmethanamine hydrochloride (**3ae)** in DMSO-d_6.

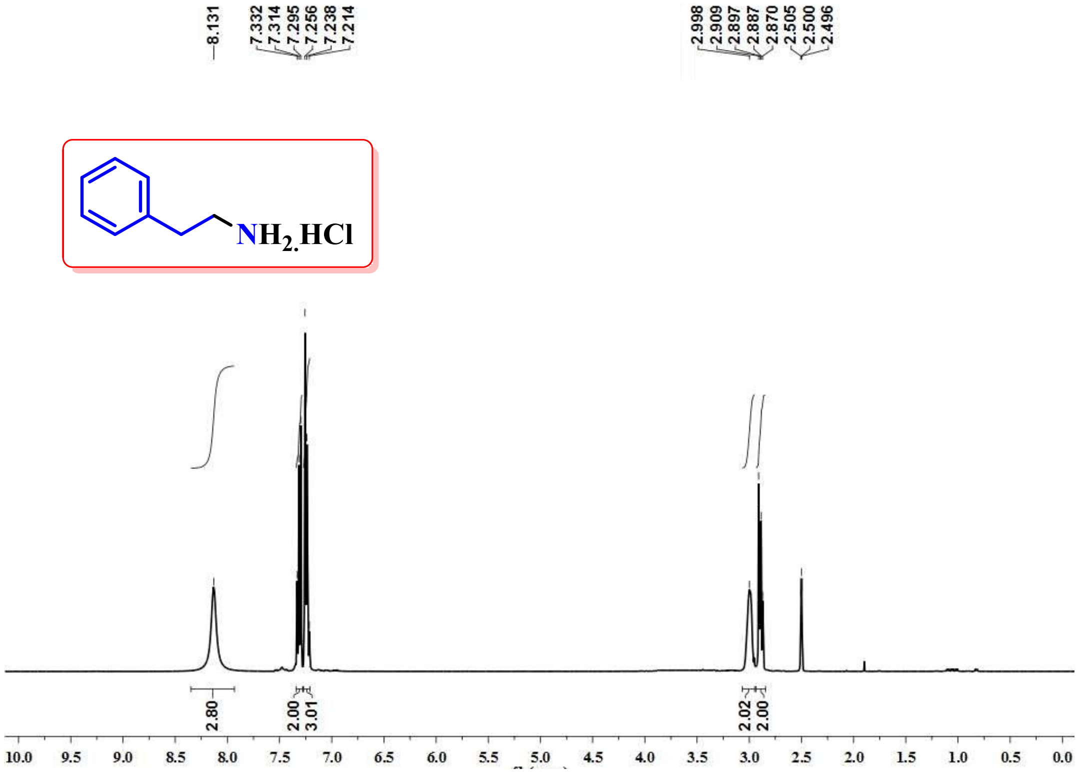

Figure S76. ^{1}H NMR spectrum of 2-phenylethanamine hydrochloride (**5a),** recorded in DMSO-d_6.

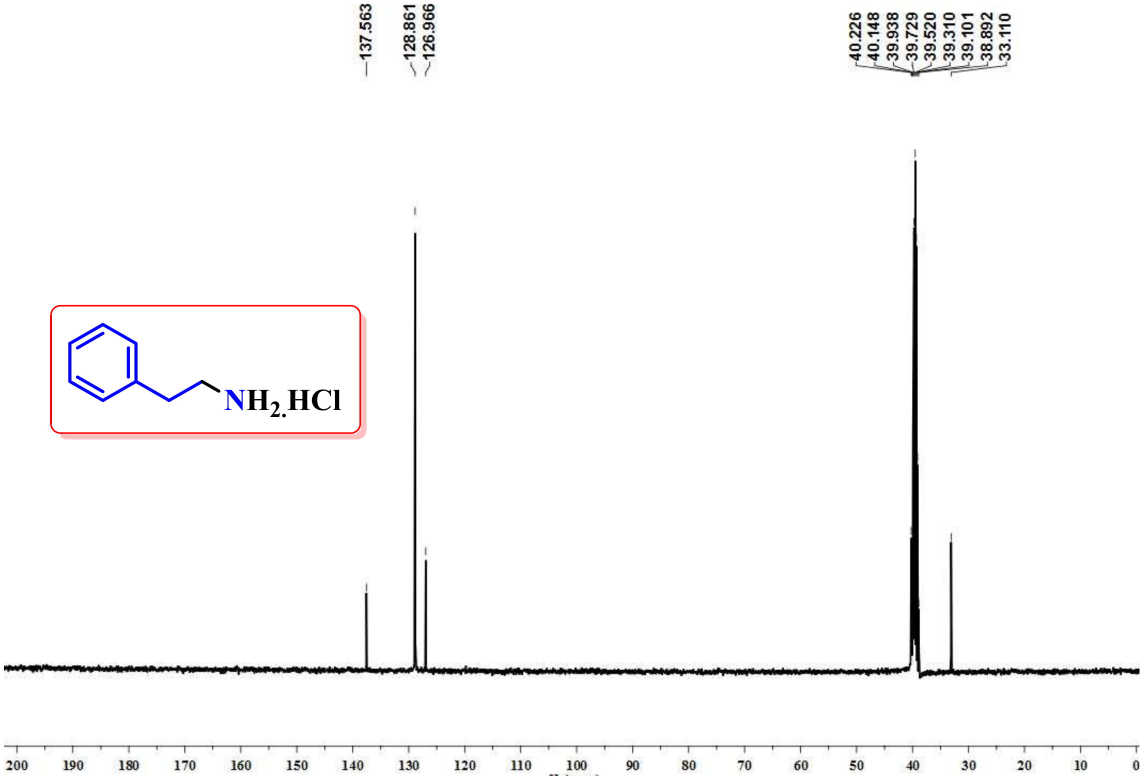

Figure S77. $^{13}C\{^{1}H\}$ NMR spectrum of 2-phenylethanamine hydrochloride (**5a),** recorded in DMSO-d_6.

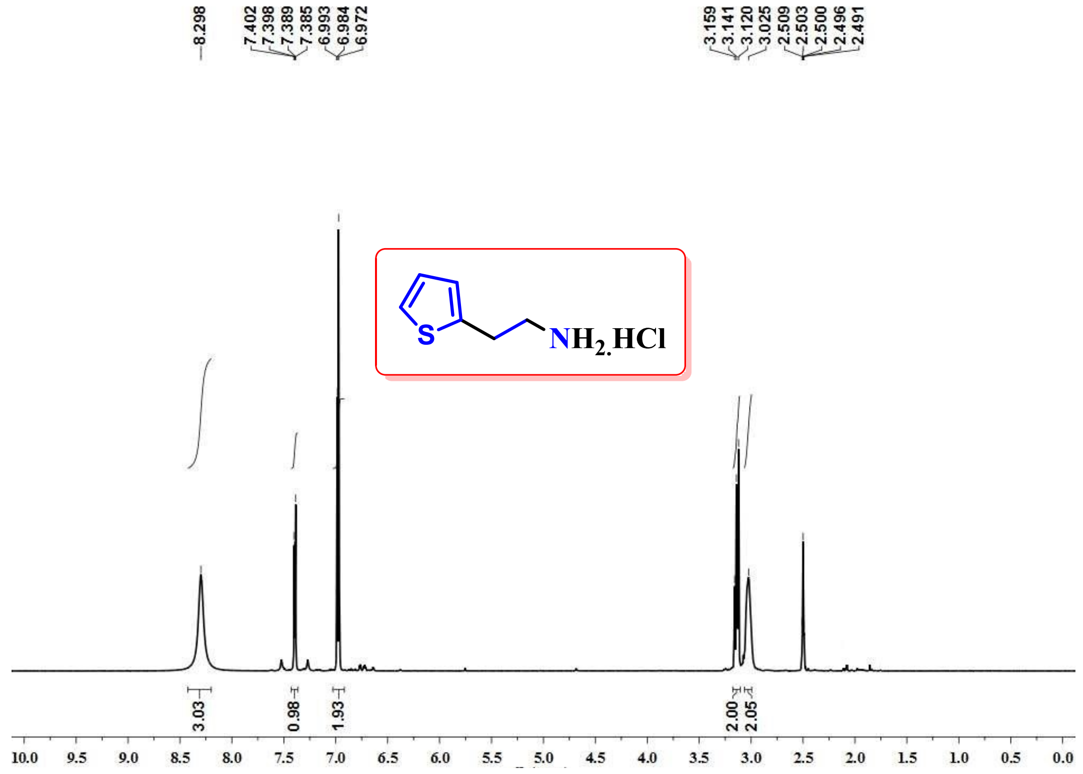

Figure S78. ^{1}H NMR spectrum of 2-(thiophen-2-yl)ethanamine hydrochloride (**5b)** in DMSO-d_6.

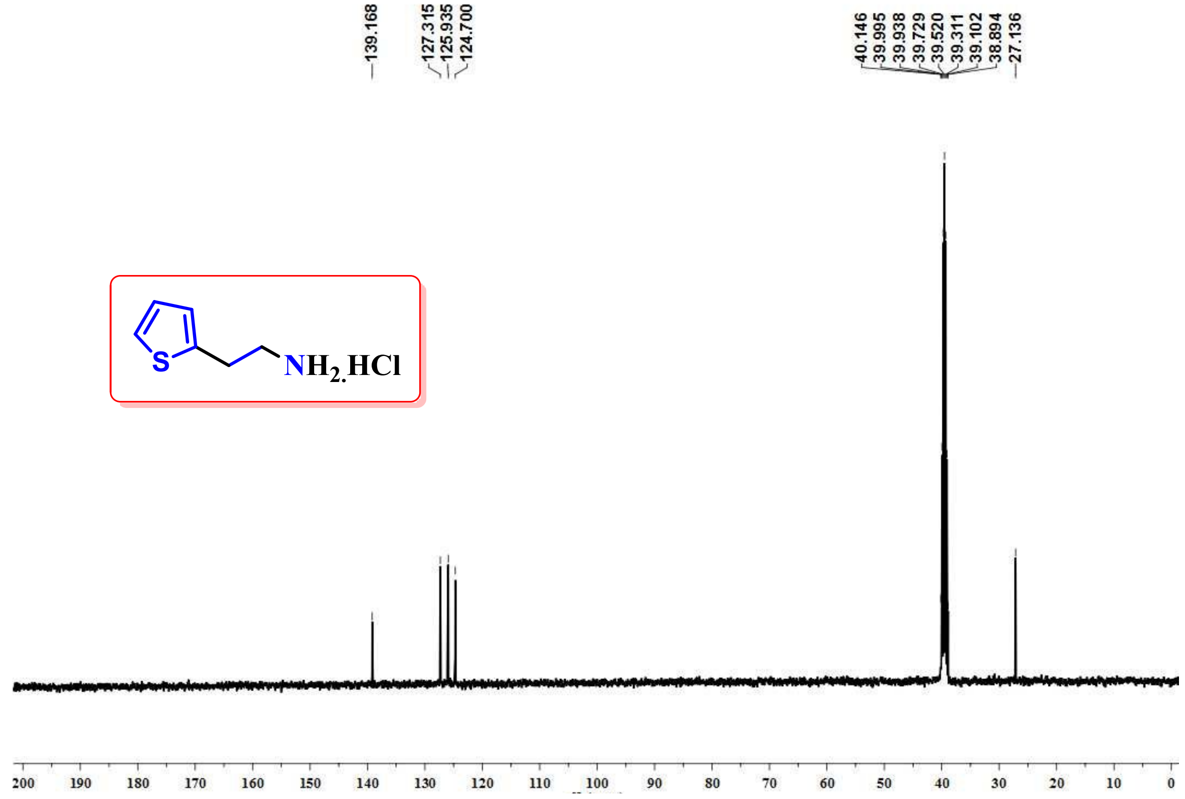

Figure S79. $^{13}C\{^{1}H\}$ NMR spectrum of 2-(thiophen-2-yl)ethanamine hydrochloride (**5b)** in DMSO-d_6.

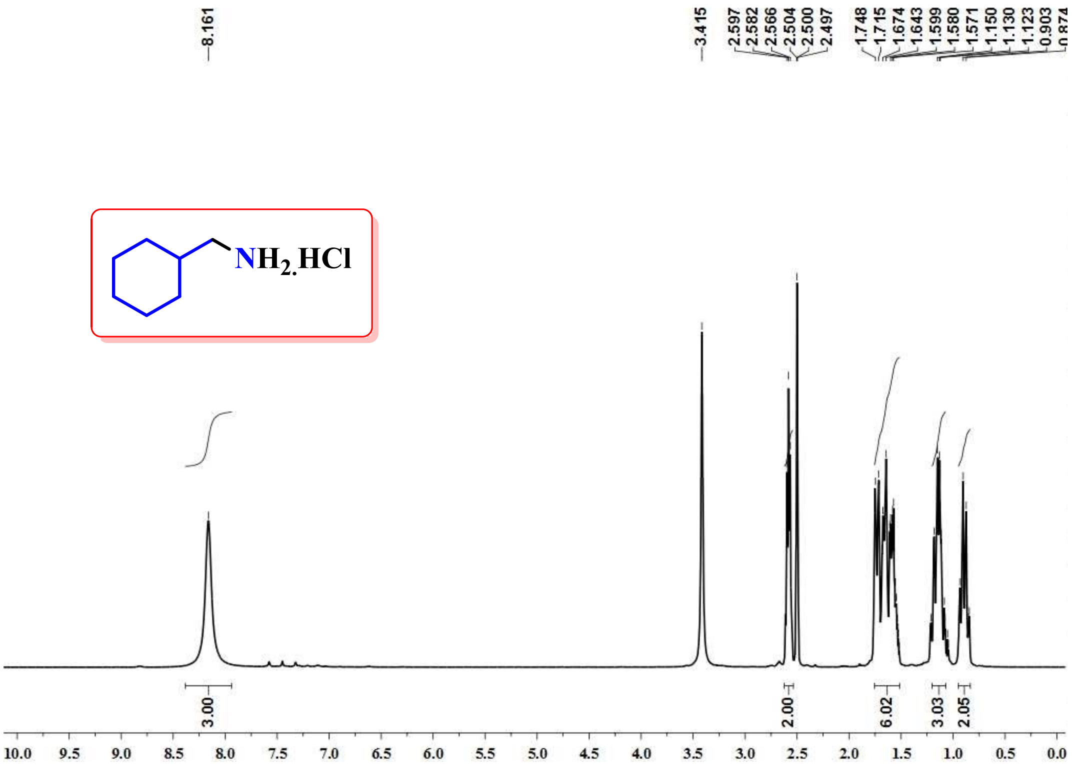

Figure S80. ^{1}H NMR spectrum of cyclohexylmethanamine hydrochloride (**5c)** in DMSO-d_6.

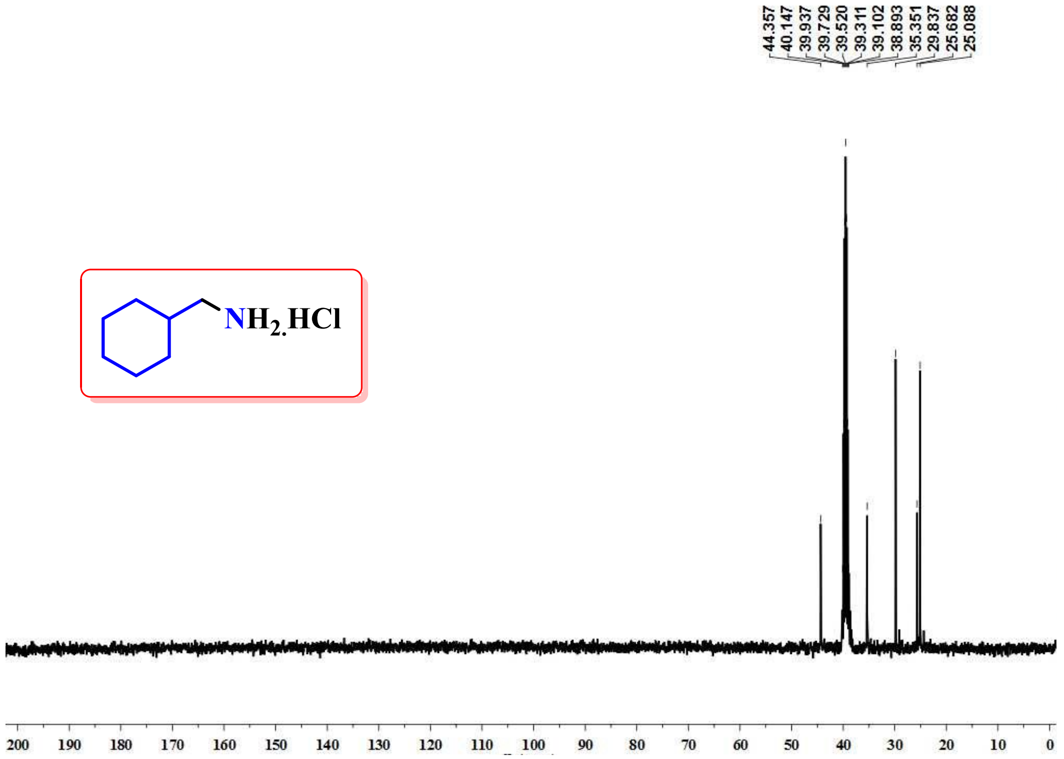

Figure S81. $^{13}C\{^{1}H\}$ NMR spectrum of cyclohexylmethanamine hydrochloride (**5c)** in DMSO-d_6.

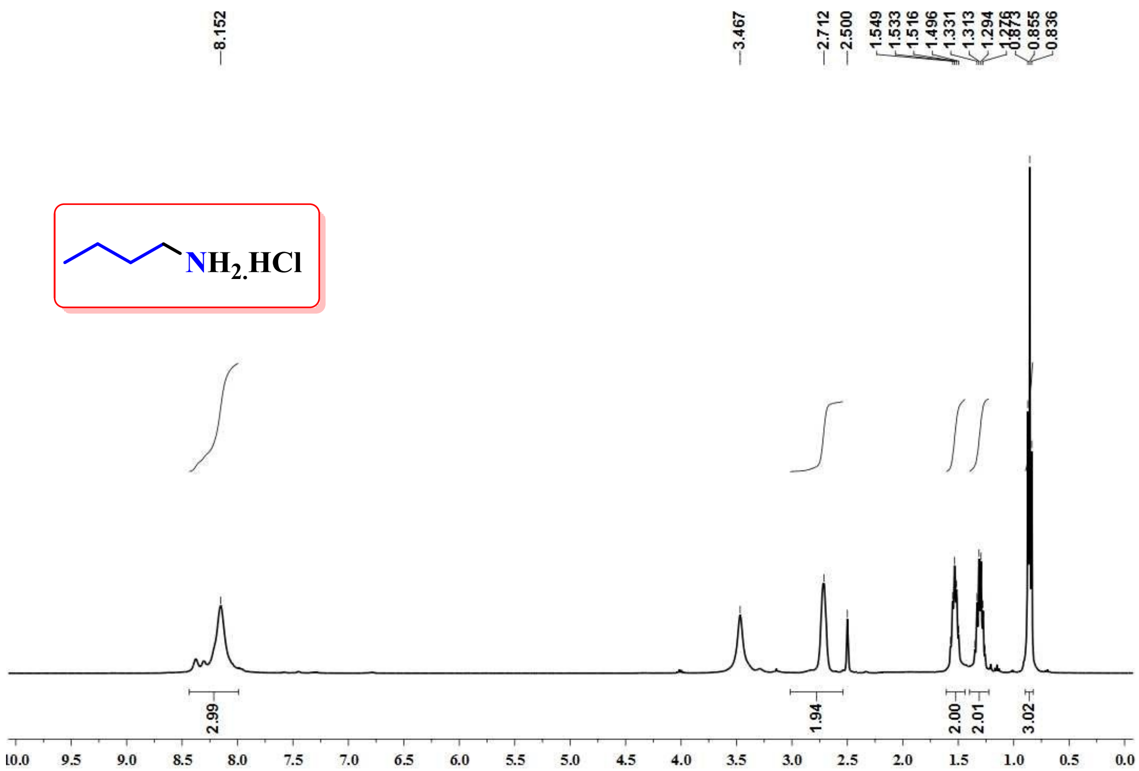

Figure S82. ^{1}H NMR spectrum of butan-1-amine hydrochloride (**5g**) in DMSO-d_6.

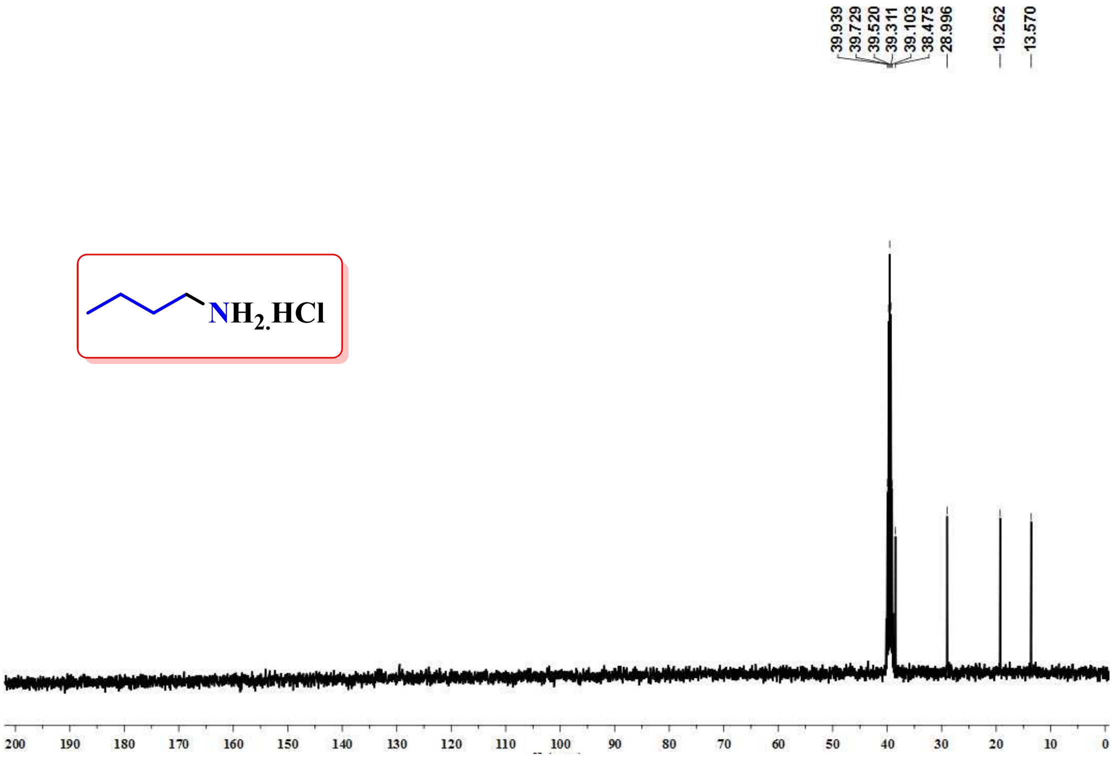

Figure S83. ^{13}C{^{1}H} NMR spectrum of butan-1-amine hydrochloride (**5g**) in DMSO-d_6.

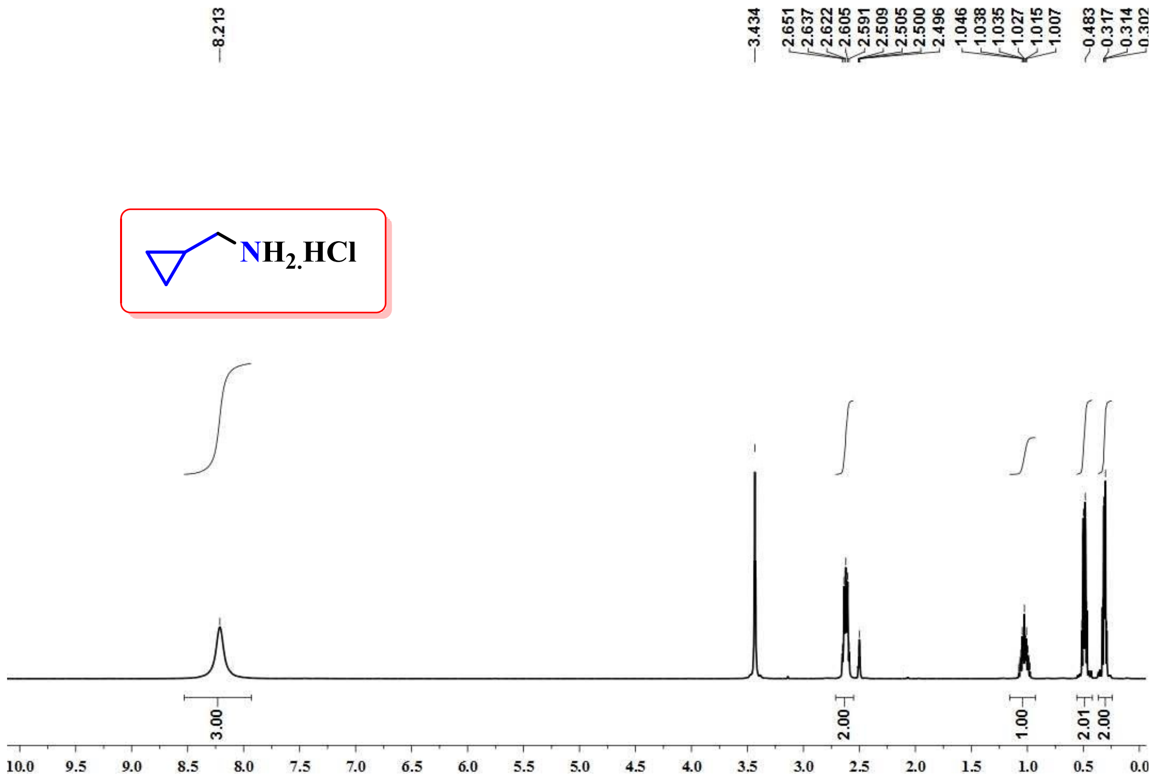

Figure S84. ^{1}H NMR spectrum of cyclopropylmethanamine hydrochloride (**5h)** in DMSO-d_6.

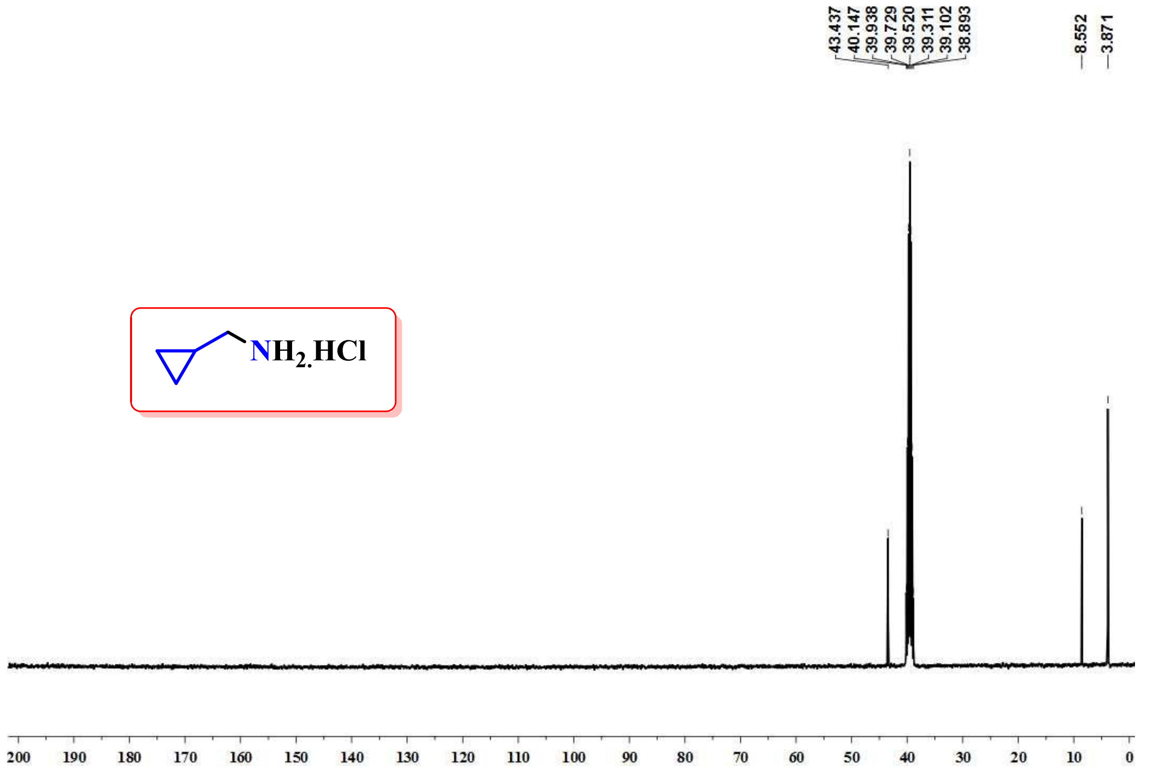

Figure S85. $^{13}C\{^{1}H\}$ NMR spectrum of cyclopropylmethanamine hydrochloride (**5h)** in DMSO-d_6.

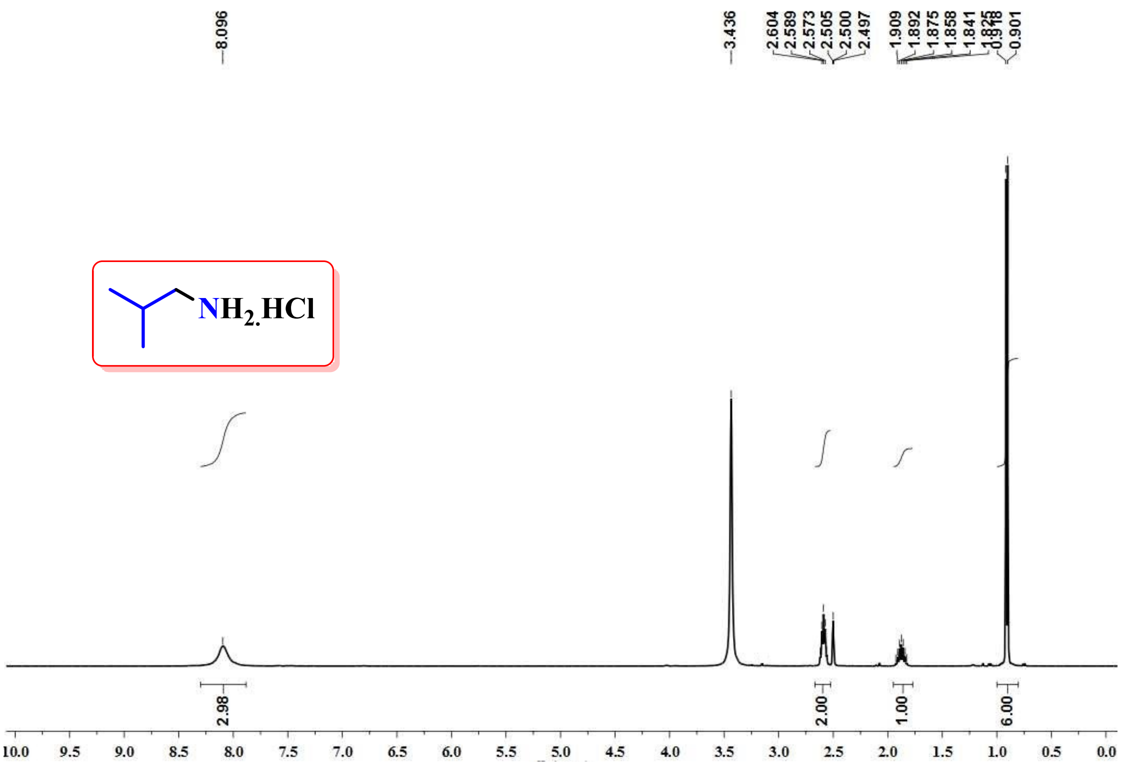

Figure S86. ^{1}H NMR spectrum of 2-methylpropan-1-amine hydrochloride (**5i)** in DMSO-d_6.

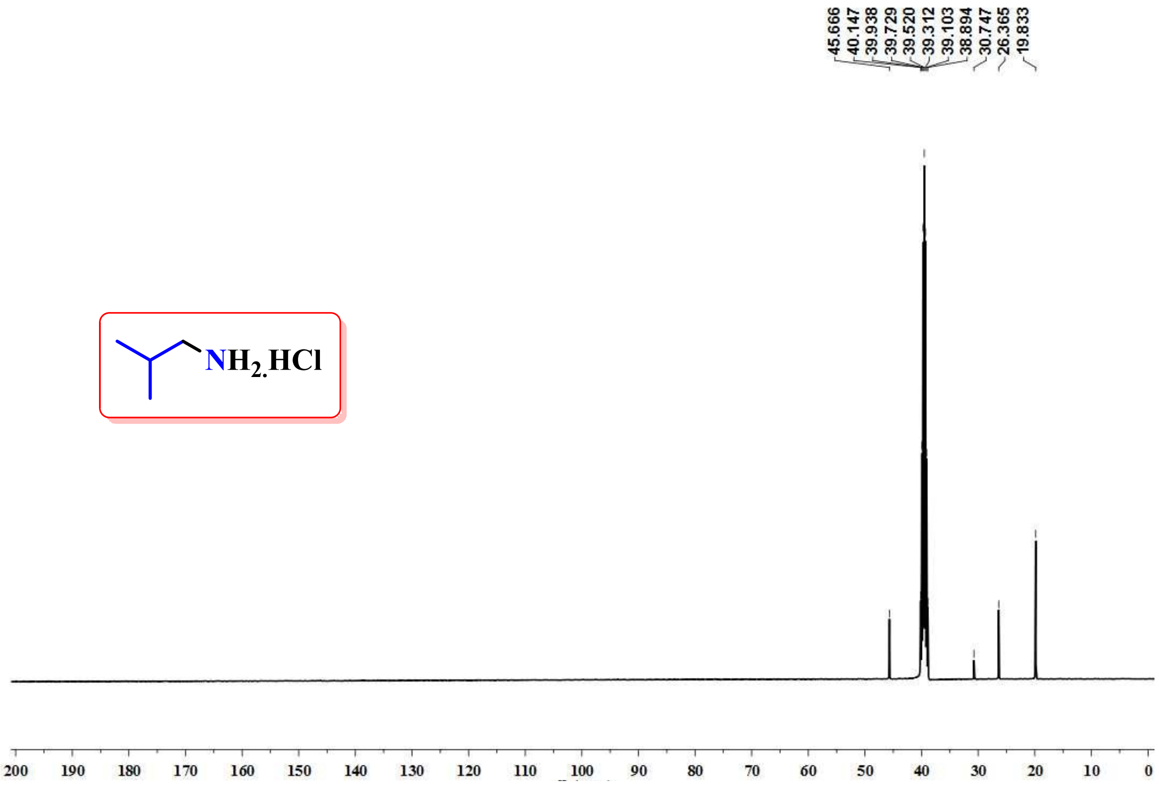

Figure S87. $^{13}C\{^{1}H\}$ NMR spectrum of 2-methylpropan-1-amine hydrochloride (**5i)** in DMSO-d_6.

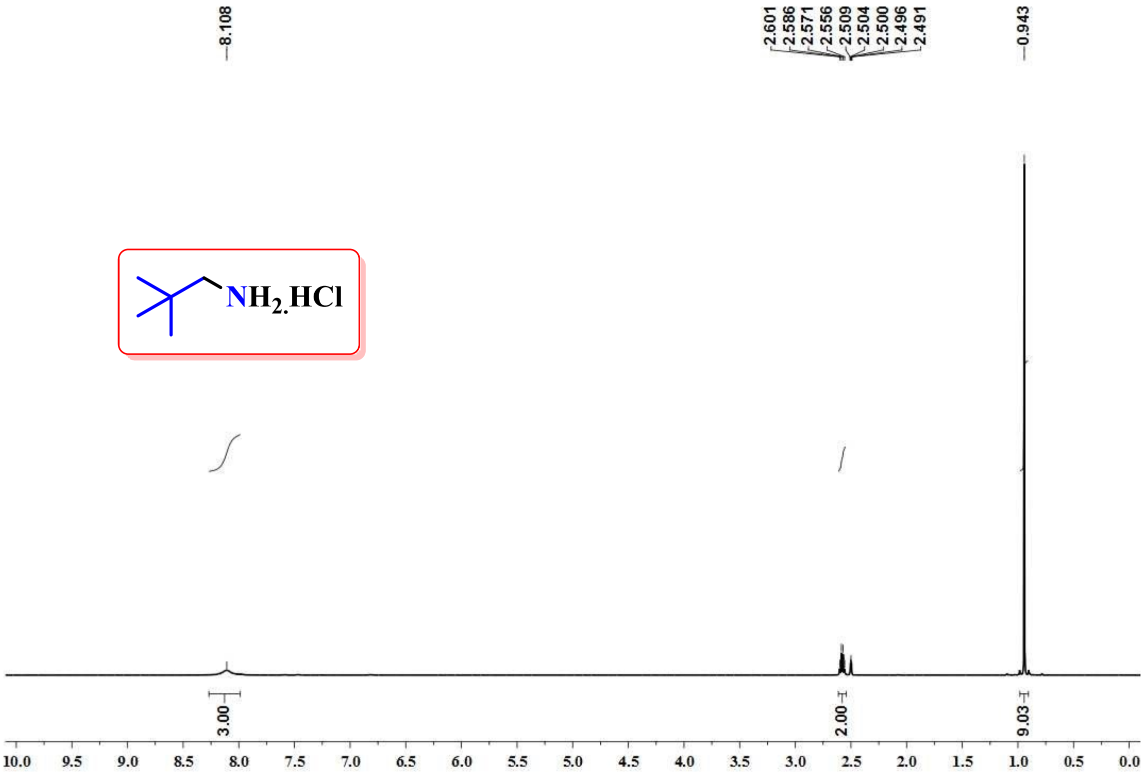

Figure S88. ^{1}H NMR spectrum of 2,2-dimethylpropan-1-amine hydrochloride (**5j)** in DMSO-d_6.

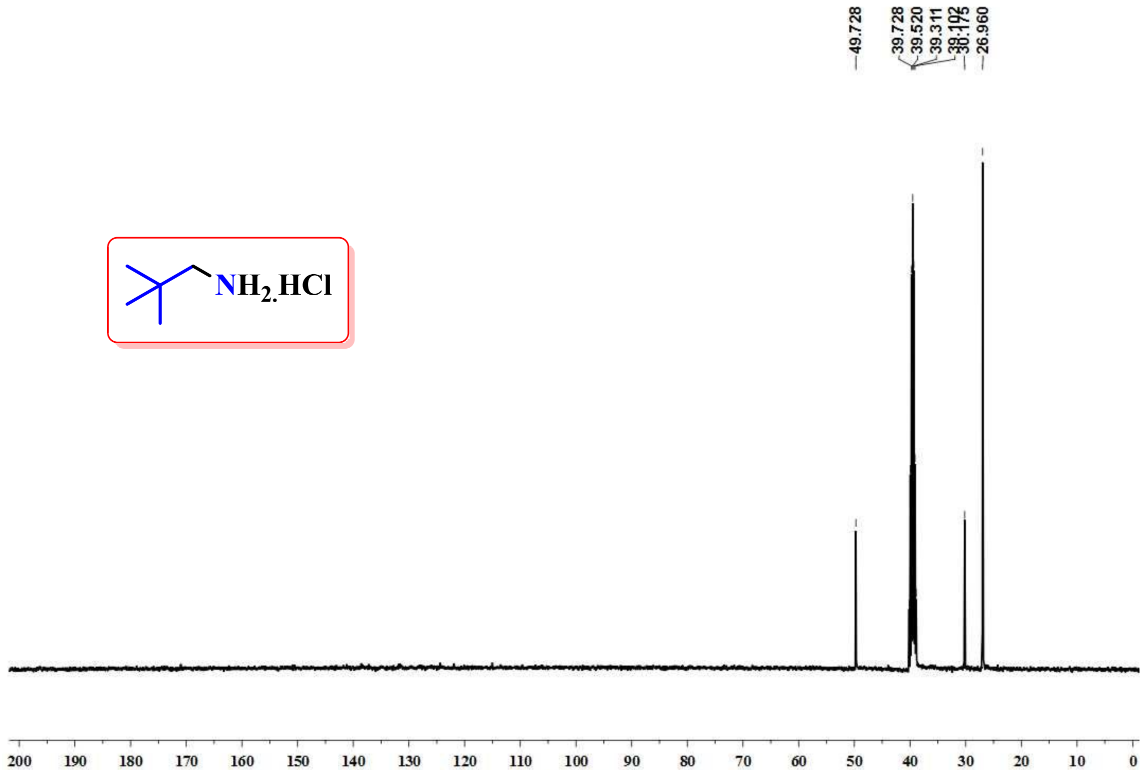

Figure S89. ^{13}C{^{1}H} NMR spectrum of 2,2-dimethylpropan-1-amine hydrochloride (**5j)** in DMSO-d_6.

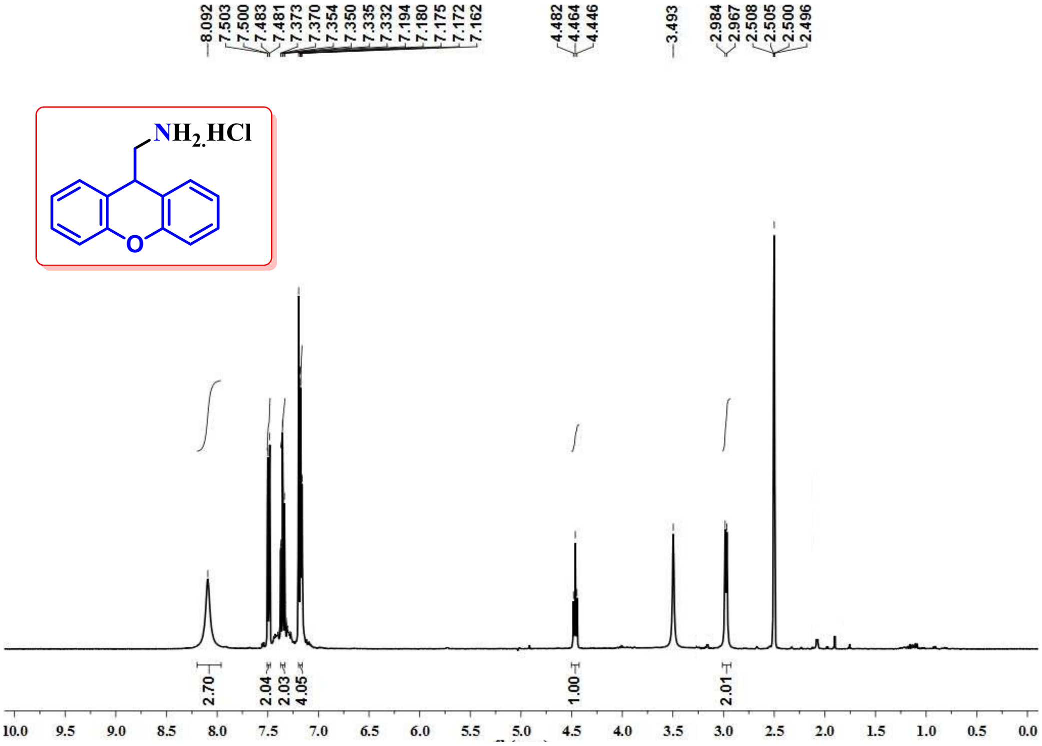

Figure S90. ^{1}H NMR spectrum of (9H-xanthen-9-yl)methanamine hydrochloride (**5l)** in DMSO-d_6.

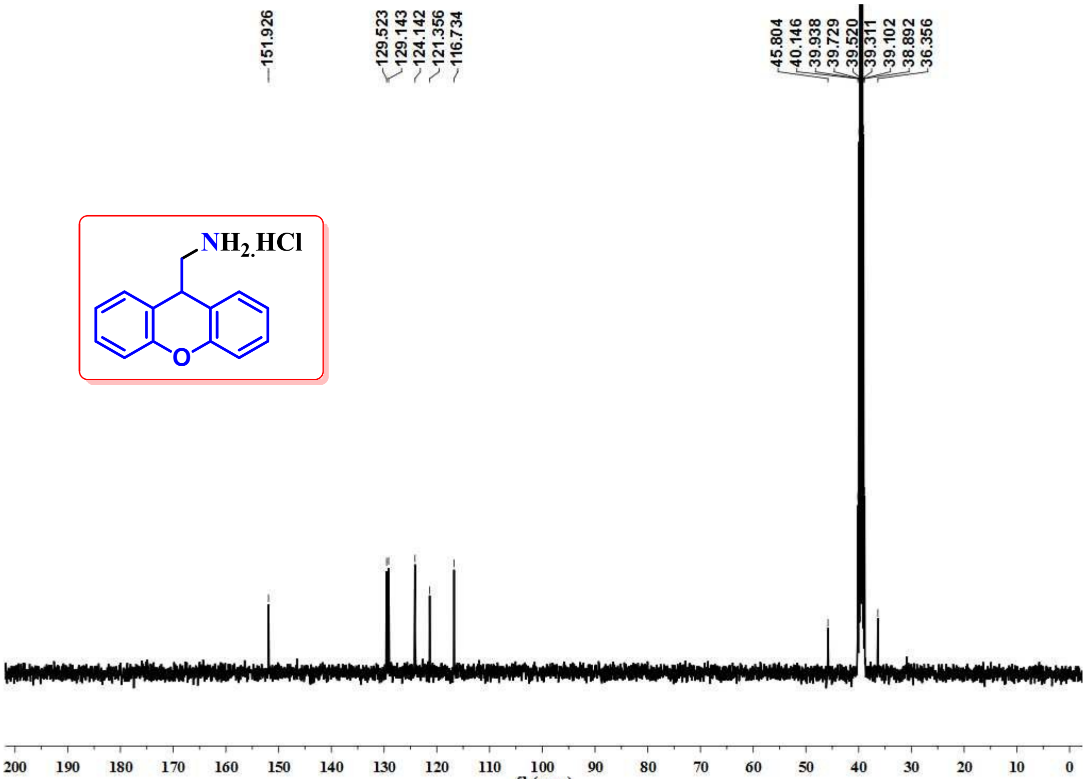

Figure S91. $^{13}C\{^{1}H\}$ NMR spectrum of (9H-xanthen-9-yl)methanamine hydrochloride (**5l)** in DMSO-d_6.

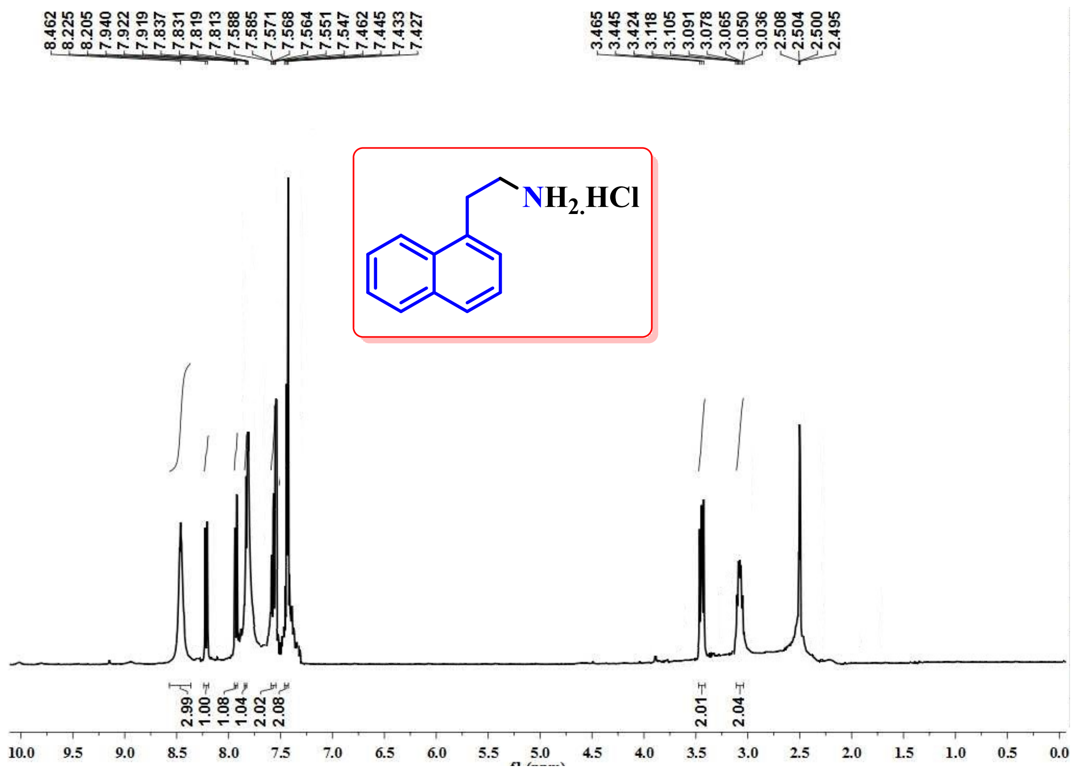

Figure S92. ^{1}H NMR spectrum of 2-(naphthalen-1-yl)ethanamine hydrochloride (**5m)** in DMSO-d$_6$.

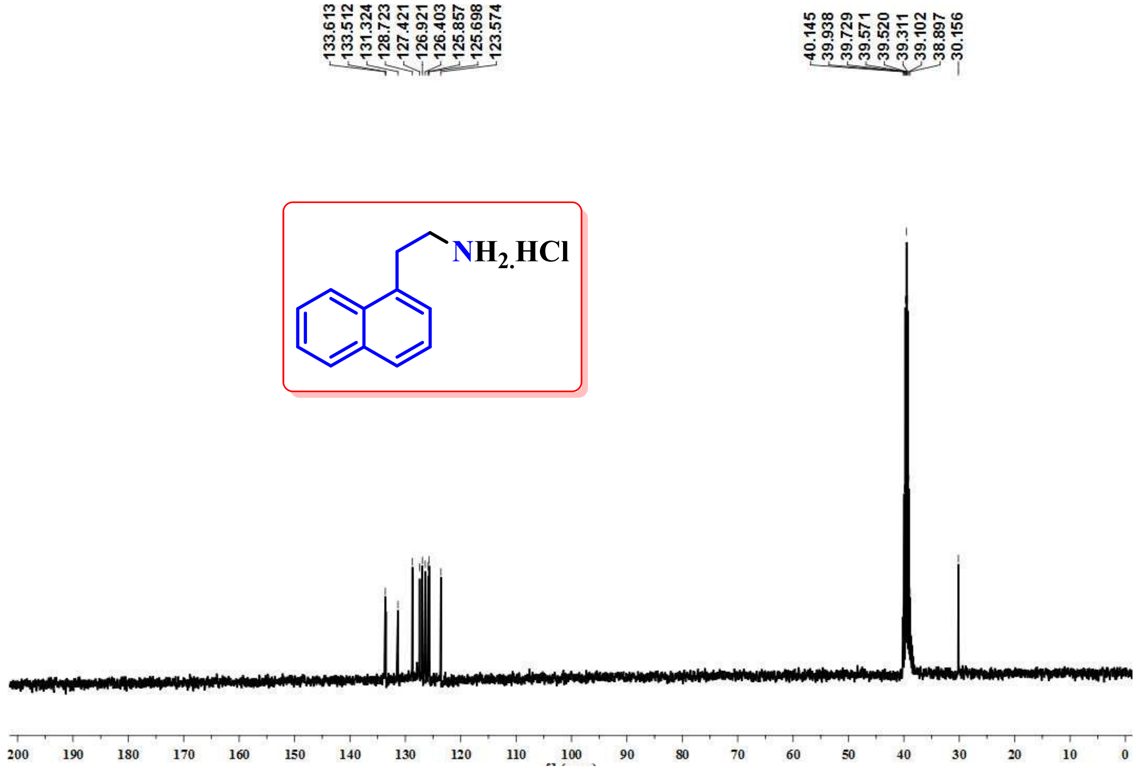

Figure S93. ^{13}C{^{1}H} NMR spectrum of 2-(naphthalen-1-yl)ethanamine hydrochloride (**5m)** in DMSO-d$_6$.

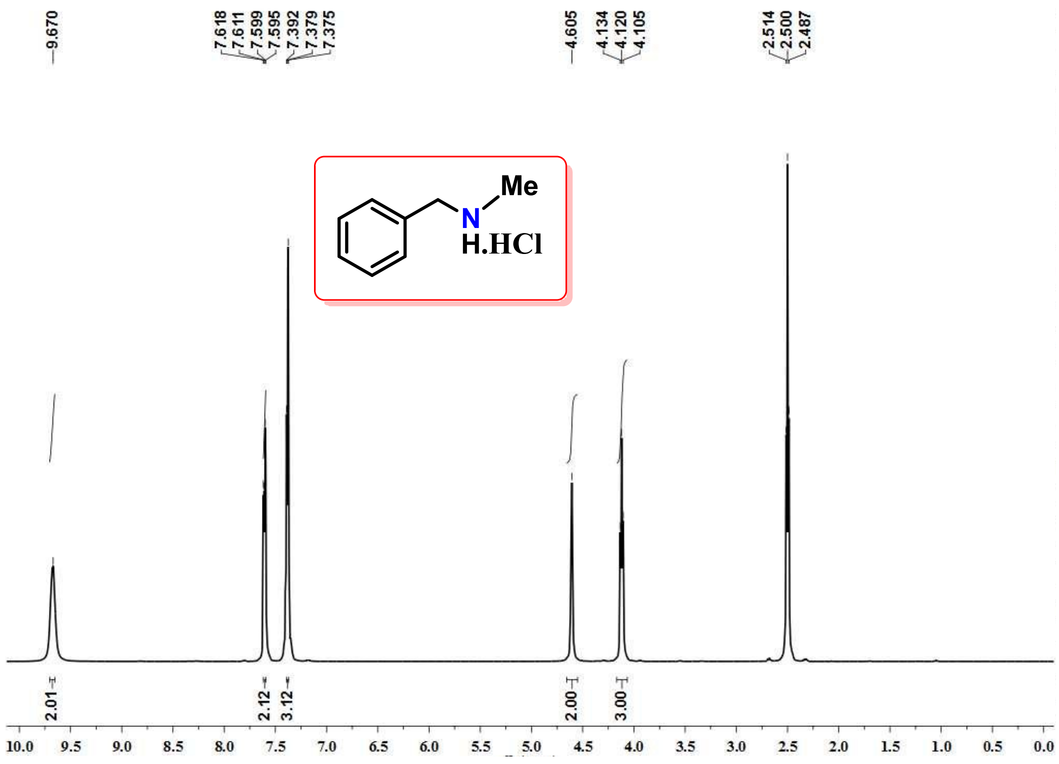

Figure S94. ^{1}H NMR spectrum of N-methyl-1-phenylmethanamine hydrochloride (**5n**) in DMSO-d_6.

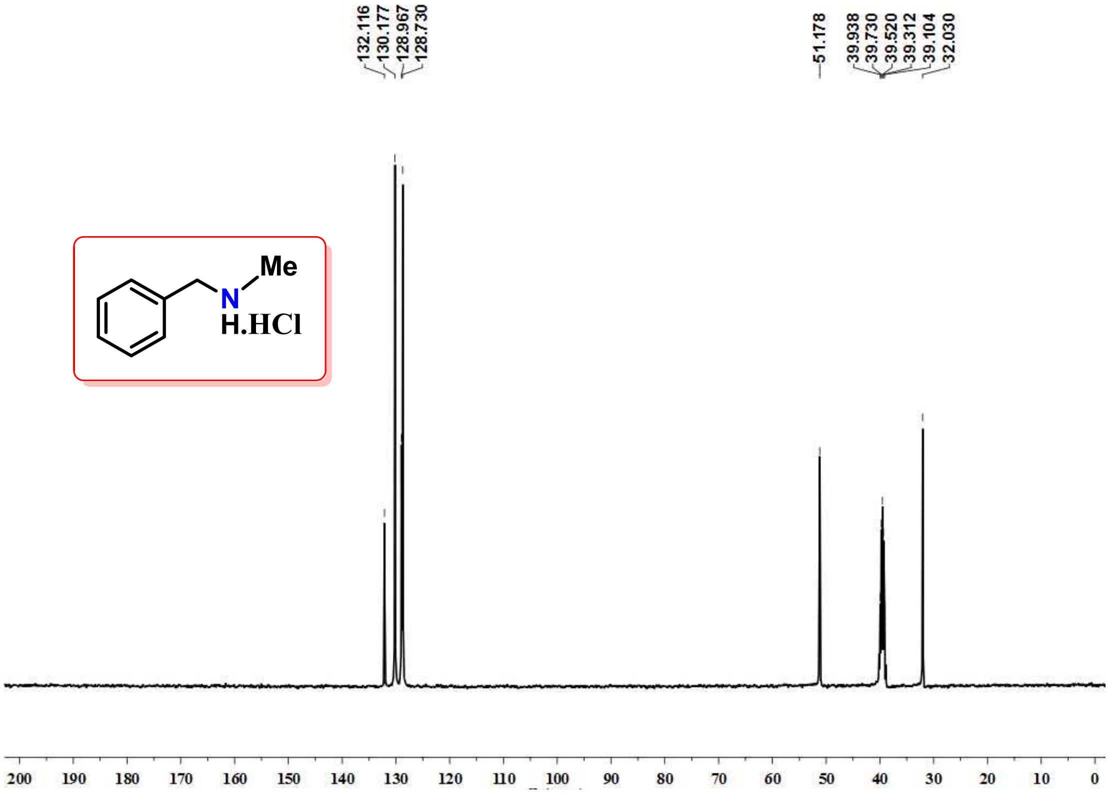

Figure S95. $^{13}C\{^{1}H\}$ NMR spectrum of N-methyl-1-phenylmethanamine hydrochloride (**5n)** in DMSO-d_6.

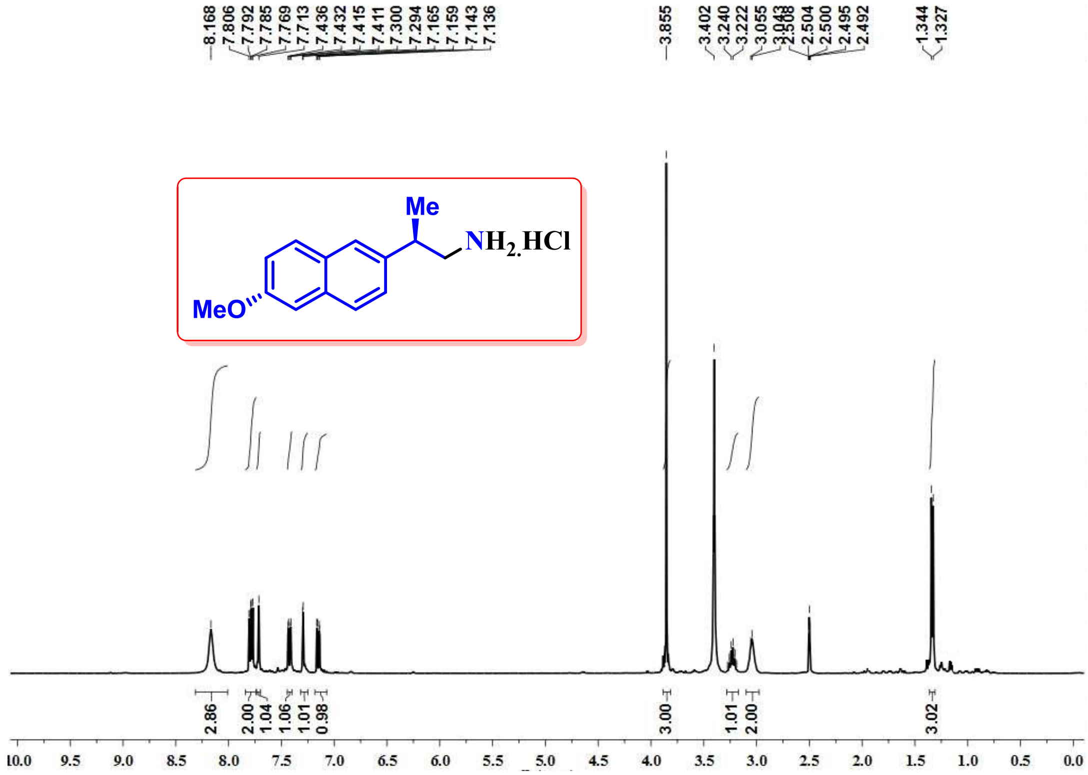

Figure S96. ^{1}H NMR spectrum of (*R*)-2-(6-methoxynaphthalen-2-yl)propan-1-amine hydrochloride (**5o**) in DMSO-d$_6$.

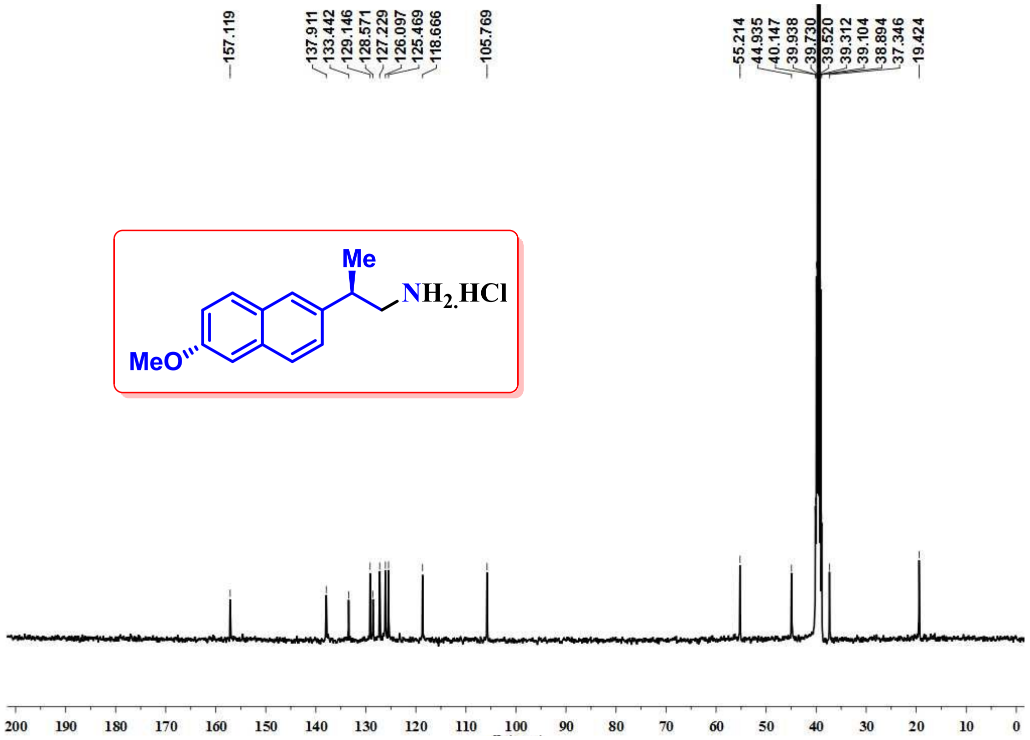

Figure S97. ^{13}C{^{1}H} NMR spectrum of (*R*)-2-(6-methoxynaphthalen-2-yl)propan-1-amine hydrochloride (**5o**) in DMSO-d$_6$.

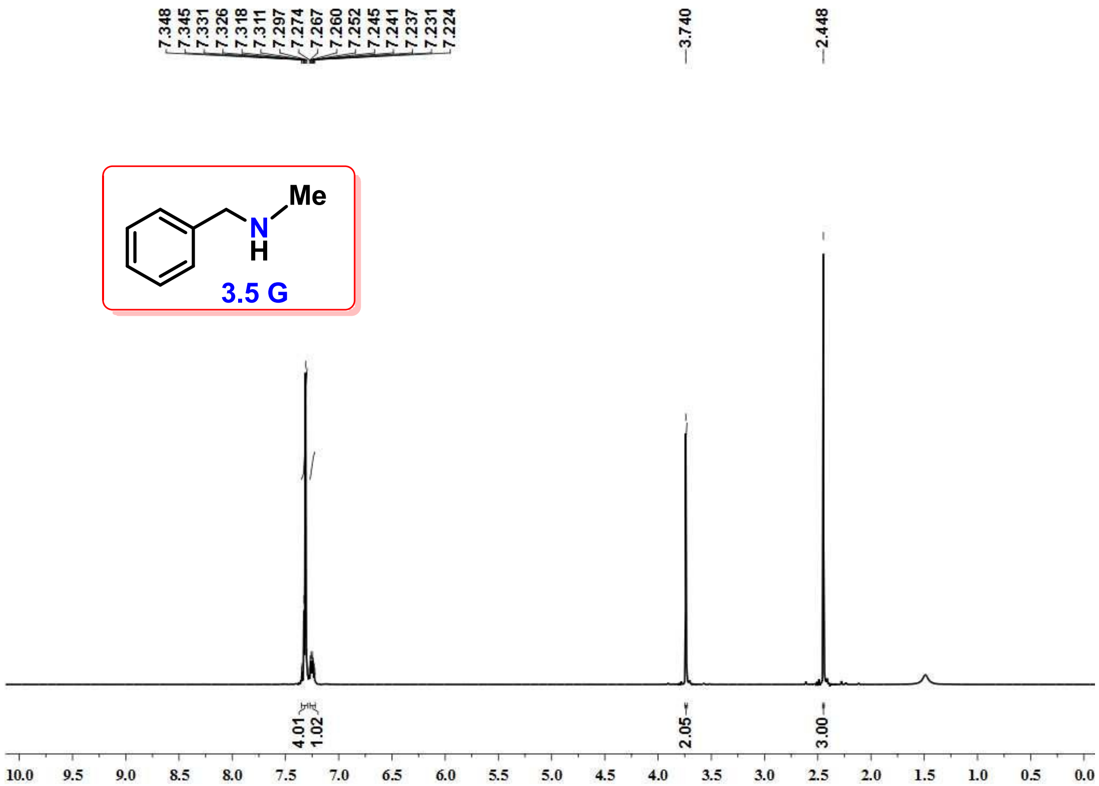

Figure S98. ^{1}H NMR spectrum of N-methyl-1-phenylmethanamine (**4′o**) in $CDCl_3$.

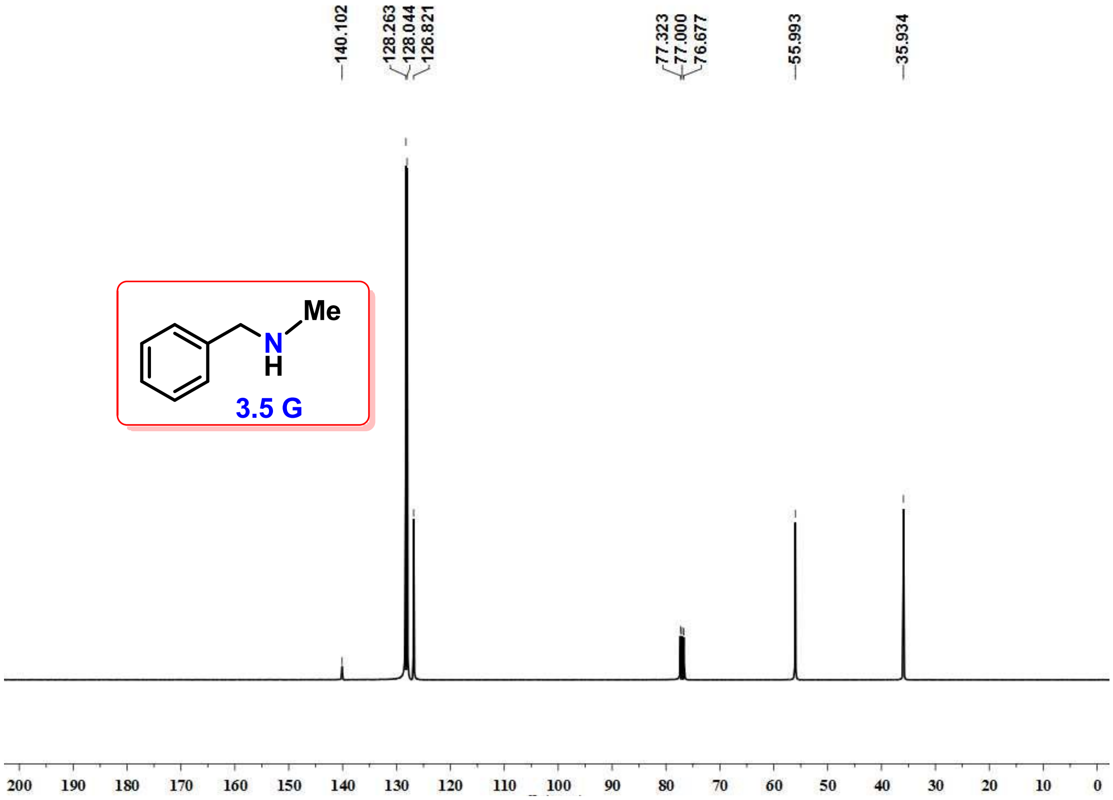

Figure S99. ^{13}C{^{1}H} NMR spectrum of N-methyl-1-phenylmethanamine (**4′o**) in $CDCl_3$.

7. Computational details and Coordinates.

All theoretical calculations for geometry optimization and Natural Bonding Orbital (NBO) analysis of all the complexes were carried out with the help of Gaussian16[S20] at B3LYP level of theory by using 6-31+g(2d,p) bases set[S21].

<u>Na-*a*NHC</u>

0 1

Si	4.88429200	-1.74208000	1.32104300
Si	5.75904700	0.44002600	-0.73761100
N	-1.99299800	-0.59987100	-0.06871400
N	-0.63800100	1.09494700	-0.21701100
C	-0.76443700	-1.05413400	-0.60641400
C	-3.26991100	2.79040000	-0.22421100
H	-2.69148000	2.99089600	-1.11624700
C	-0.07442900	2.42772800	-0.10941900
C	-3.29347400	-2.00922400	1.47961700
C	-1.90337900	0.73955000	0.13232500
C	0.12842600	0.00973700	-0.66353100
C	0.07565500	3.19265300	-1.28865800
C	-3.97403300	-0.94793600	-2.20122900
H	-3.05999600	-0.34732600	-2.20625000
C	-4.10365200	-1.60108900	-0.82797800
C	0.37205400	2.88375200	1.14807200
C	-3.77249200	1.40559400	1.68990600
H	-3.57211600	0.54371200	2.30959300
C	-3.16678900	-1.40474300	0.20538000
C	-0.35629400	2.69029300	-2.66652500
H	-1.00043100	1.81713700	-2.52173300

C	-5.06284400	3.41387500	1.27823000
H	-5.85987100	4.09473400	1.56131600
C	-2.99220300	1.64634100	0.54631800
C	0.67524300	4.45254200	-1.17047600
H	0.81177900	5.06346300	-2.05696100
C	0.95639000	4.15726900	1.20537800
H	1.31150400	4.53612500	2.15865100
C	-4.39768400	-2.84653600	1.68142800
H	-4.52860000	-3.33407700	2.64177400
C	0.45120800	-2.48735500	-2.19898900
H	0.91366100	-1.57401900	-2.55676100
C	-0.51297600	-2.39979900	-1.16967500
C	-1.14576000	-3.59488500	-0.77726700
H	-1.90034700	-3.59283000	-0.00439700
C	1.10766400	4.93600500	0.06267900
H	1.57260500	5.91528800	0.12953400
C	-4.29260100	3.66540900	0.14016400
H	-4.48916800	4.54127200	-0.47076600
C	0.28033700	2.05197100	2.42497700
H	-0.18771200	1.09506300	2.17625100
C	-0.81206700	-4.81558200	-1.36987700
H	-1.31742100	-5.71834700	-1.03863400
C	-4.79854100	2.27980900	2.04940600
H	-5.38630500	2.07520600	2.93923300
C	-1.16345000	3.73642400	-3.46202200
H	-2.01547700	4.12487000	-2.89429600

H	-1.54834200	3.28648500	-4.38347400
H	-0.54472700	4.59150700	-3.75316600
C	-5.33617400	-3.06635900	0.67356200
H	-6.18269100	-3.72202800	0.85627100
C	-0.59914600	2.73665200	3.49129600
H	-0.15822600	3.68336000	3.82177200
H	-0.69385700	2.09277600	4.37291600
H	-1.60314900	2.94785600	3.11172000
C	-2.28584300	-1.79595600	2.61489300
H	-1.71200300	-0.89125000	2.38839900
C	-5.19288600	-2.44281400	-0.56185700
H	-5.93361100	-2.61593800	-1.33635300
C	0.16228700	-4.88453000	-2.36598900
H	0.42252500	-5.83678700	-2.81810000
C	-5.15356000	0.00492300	-2.48458800
H	-6.10339800	-0.53876600	-2.53427700
H	-5.00904400	0.50645000	-3.44789800
H	-5.24473000	0.77259400	-1.71029500
C	0.79256700	-3.70706800	-2.77864700
H	1.54307400	-3.73425000	-3.56338400
C	-2.97117500	-1.57945800	3.98086700
H	-3.75978800	-0.82125300	3.94258800
H	-2.22863900	-1.25939900	4.71877500
H	-3.42102200	-2.50413700	4.35707700
C	1.67917000	1.73749600	2.99459800
H	2.31932600	1.23849100	2.26097900

H	1.59275300	1.08138700	3.86747000
H	2.19426500	2.64940500	3.31504100
C	-3.83238300	-1.99872300	-3.32127300
H	-2.98265200	-2.66287300	-3.14081500
H	-3.67884100	-1.50103100	-4.28499900
H	-4.73364500	-2.61584200	-3.40700400
C	0.86031900	2.22278200	-3.49174400
H	1.55859800	3.04850200	-3.66595500
H	0.53533400	1.84177600	-4.46661500
H	1.40057900	1.42603300	-2.97560700
C	-1.27006800	-2.95325000	2.73465600
H	-1.78378400	-3.90715500	2.90056400
H	-0.60526100	-2.77662100	3.58657900
H	-0.64511600	-3.05103600	1.84553700
N	4.69569900	-0.55541000	0.12644600
C	4.75655200	1.41483900	-2.05488500
H	4.26470600	0.73860400	-2.76918800
H	5.40102800	2.08008200	-2.64347200
H	3.98556200	2.05017600	-1.59550600
C	7.12791400	-0.47123500	-1.70988900
H	7.80698100	-1.00218000	-1.03128100
H	7.73375200	0.22180400	-2.30860200
H	6.69697000	-1.21771300	-2.38841800
C	6.65131900	1.76832500	0.30693800
H	5.92811900	2.39868800	0.83929800
H	7.28024900	2.42249900	-0.31148900

H	7.29667900	1.30467300	1.06315000
C	5.74955000	-1.15149900	2.92001900
H	6.78355700	-0.84543400	2.71761000
H	5.78175400	-1.94075000	3.68290300
H	5.23254300	-0.28493400	3.35045700
C	5.82980500	-3.30582700	0.76252400
C	3.15342500	-2.36302900	1.87013900
H	5.35157600	-3.75945200	-0.11453000
H	5.87030000	-4.06439800	1.55559500
H	2.59629500	-2.80863400	1.03393100
H	3.23216800	-3.13532800	2.64608400
H	6.86202800	-3.06367000	0.48135500
H	2.54625200	-1.54771500	2.28733300
Na	2.58413500	-0.25042500	-0.52597900

K-*a*NHC

0 1

Si	-5.51651700	1.96004500	0.86741600
Si	-6.22727700	-0.68615800	-0.64636200
N	2.37531900	0.50494700	-0.11914700
N	0.75851600	-0.94761500	-0.11660800
C	1.19909200	1.13017200	-0.59982400
C	2.95351500	-3.12976500	0.26731500
H	2.26559300	-3.40689000	-0.51965800
C	-0.02959400	-2.14670700	0.08374600
C	3.90105300	2.03879900	1.06164500

C	2.07745300	-0.78684200	0.18172400
C	0.14785300	0.22419800	-0.59491500
C	-0.30290100	-2.96742400	-1.03409000
C	4.48023100	-0.18327500	-2.08831800
H	3.46206600	-0.57718700	-2.02723400
C	4.68902600	0.78976900	-0.92719800
C	-0.57252400	-2.40119800	1.35948200
C	3.91300500	-1.48095100	1.74926200
H	3.96349900	-0.47168800	2.13384700
C	3.68458900	1.11586900	0.01265700
C	0.27954800	-2.69064700	-2.42127500
H	1.11547600	-1.99403600	-2.30026600
C	4.71215500	-3.76700100	1.80631100
H	5.37274000	-4.52308000	2.21971900
C	2.99185600	-1.80199000	0.73422800
C	-1.15080700	-4.06491900	-0.83911900
H	-1.39141400	-4.71090100	-1.67722300
C	-1.40438200	-3.52205600	1.49751900
H	-1.84122400	-3.74418600	2.46616700
C	5.17136400	2.62273300	1.15526000
H	5.36987500	3.33482300	1.95016200
C	-0.04262100	3.26945600	-0.67524500
H	-0.79645900	2.79671400	-0.05505700
C	1.11433800	2.54297700	-1.02017300
C	2.09543500	3.19506100	-1.78834500
H	2.99609100	2.66935200	-2.07854400

C	-1.69729800	-4.34301000	0.41288300
H	-2.35739800	-5.19599000	0.53940900
C	3.80201200	-4.09961300	0.80004500
H	3.75296100	-5.11615900	0.42154400
C	-0.30659500	-1.51154100	2.57117700
H	0.33133500	-0.68310900	2.25087100
C	1.92430300	4.52014500	-2.19571800
H	2.69731400	4.99921900	-2.79000400
C	4.76381900	-2.45225700	2.27585200
H	5.46328400	-2.17878600	3.06011600
C	0.83287600	-3.95932900	-3.10063000
H	1.54096600	-4.49472300	-2.45937200
H	1.35319300	-3.68850600	-4.02553200
H	0.03534200	-4.65908000	-3.37078100
C	6.18254600	2.31201100	0.25018800
H	7.15904200	2.77831800	0.34414600
C	0.44622400	-2.27666100	3.67918400
H	-0.15842200	-3.09992400	4.07516100
H	0.67583700	-1.60431800	4.51333300
H	1.38660800	-2.69687100	3.31011200
C	2.83216400	2.42895300	2.08440400
H	1.90834100	1.90046800	1.83441800
C	5.93841500	1.40627800	-0.77857900
H	6.73165700	1.17499000	-1.48275000
C	0.76727000	5.22368900	-1.85296600
H	0.63592700	6.25316400	-2.17275000

C	5.44908300	-1.38147000	-2.00679100
H	6.48810300	-1.06136100	-2.14096900
H	5.22187800	-2.10310700	-2.79913600
H	5.37603500	-1.89649500	-1.04568600
C	-0.21769400	4.58934800	-1.08969500
H	-1.11841200	5.12405800	-0.80247400
C	3.22430400	2.02200200	3.52101100
H	3.38370800	0.94400700	3.61973300
H	2.42775800	2.30399000	4.21786500
H	4.14106500	2.52700600	3.84424900
C	-1.60765100	-0.89325300	3.12133000
H	-2.14239900	-0.32335500	2.35578300
H	-1.38125500	-0.21349300	3.94994000
H	-2.29145200	-1.66036300	3.49944600
C	4.61296100	0.51498500	-3.45826900
H	3.89075500	1.32796100	-3.57498300
H	4.43625100	-0.20604700	-4.26385800
H	5.61607600	0.93154800	-3.60008700
C	-0.74952500	-2.00007800	-3.34002900
H	-1.65548600	-2.60673400	-3.44645900
H	-0.32611200	-1.84365300	-4.33851700
H	-1.02316100	-1.02082400	-2.93813900
C	2.52729300	3.94120400	2.03387600
H	3.39229300	4.53082800	2.35750100
H	1.69565300	4.17695500	2.70631400
H	2.25213900	4.26428400	1.02767400

N	-5.28056000	0.59061700	-0.08566400
C	-5.16996700	-1.80659200	-1.80493800
H	-4.80020100	-1.25217900	-2.68046800
H	-5.76038600	-2.64582400	-2.19409000
H	-4.30867800	-2.24648100	-1.28102200
C	-7.74593200	-0.19189300	-1.69762500
H	-8.46005000	0.39235800	-1.10409100
H	-8.27864900	-1.06884400	-2.08922000
H	-7.44699700	0.43090100	-2.54997400
C	-6.89160900	-1.86855200	0.70269100
H	-6.07085200	-2.27420800	1.30752700
H	-7.44731200	-2.71358200	0.27444200
H	-7.56686200	-1.33888300	1.38620600
C	-5.98441100	1.62243400	2.69139800
H	-6.95429500	1.11370900	2.75770900
H	-6.05436900	2.54834400	3.27798100
H	-5.24377100	0.97159300	3.17295000
C	-6.81484500	3.20567100	0.22041900
C	-3.87455900	2.96438200	0.95555500
H	-6.57899200	3.52109100	-0.80364300
H	-6.87097000	4.10542600	0.84772400
H	-3.55479900	3.29830800	-0.04249600
H	-3.98671700	3.86663400	1.57010300
H	-7.81422600	2.75375700	0.19818800
H	-3.05931600	2.37719500	1.40348300
K	-2.80332800	0.38500500	-0.97772000

8. References.

S1. M. Bhunia, G. Vijaykumar, D. Adhikari and S. K. Mandal, *Inorg. Chem.*, 2017, **56**, 14459-14466.

S2. H. S. Das, S. Das, K. Dey, B. Singh, R. K. Haridasan, A. Das, J. Ahmed and S. K. Mandal, *Chem. Commun.*, 2019, **55**, 11868-11871.

S3. N. Gandhamsetty, J. Jeong, J. Park, S. Park and S. Chang, *J. Org. Chem.*, 2015, **80**, 7281-7287.

S4. F. Chen, C. Topf, J. Radnik, C. Kreyenschulte, H. Lund, M. Schneider, A.-E. Surkus, L. He, K. Junge and M. Beller, *J. Am. Chem. Soc.*, 2016, **138**, 8781-8788.

S5. S. Wübbolt and M. Oestreich, *Synlett.*, 2017, **28**, 2411-2414.

S6. J. B. Geri and N. K. Szymczak, *J. Am. Chem. Soc.* 2015*,* **137**, 12808-12814.

S7. K.Tokmic, B. J. Jackson, A. Salazar, T. J. Woods and A. R. Fout, *J. Am. Chem. Soc.,* 2017*,* **139***,* 13554-13561.

S8. H. C. Brown, Y. M. Choi and S. Narasimhan, *J. Org. Chem.*, 1982, **47**, 3153-3163.

S9. Q. Guan, M. Jiang, J. Wu, Y. Zhai, Y. Wu, K. Bao and W. Zhang, *Green Chem.*, 2016, **18**, 5794-5799.

S10. S. Laval, W. Dayoub, L. Pehlivan, E. Métay, A. Favre-Réguillon, D. Delbrayelle, G. Mignani and M. Lemaire, *Tetrahedron Letters*, 2011, **52**, 4072-4075.

S11. T. Senthamarai, K. Murugesan, J. Schneidewind, N. V. Kalevaru,W. Baumann,H. Neumann, P. C. J. Kamer, M. Beller and R. V. Jagadeesh, *Nat. Commun.*, 2018, **9**, 4123.

S12. W. Yao, H. Fang, Q. He, D. Peng, G. Liu and Z. Huang, *J. Org. Chem.*, 2019, **84**, 6084-6093.

S13. N. Ostermann, S. Ruedisser, C. Ehrhardt, W. Breitenstein, A. Marzinzik, E. Jacoby, E. Vangrevelinghe, J. Ottl, M. Klumpp, J. C. D. Hartwieg, F. Cumin, U. Hassiepen, J. Trappe, R. Sedrani, S. Geisse, B. Gerhartz, P. Richert, E. Francotte, T. Wagner, M. Krömer, T. Kosaka, R. L. Webb, D. F. Rigel, J. Maibaum and D. K. Baeschlin, *J. Med. Chem.,* 2013, **56**, 2196-2206.

S14. N. L. Lampland, M. Hovey, D. Mukherjee and A. D. Sadow, *ACS Catal.*, 2015, **5**, 4219-4226.

S15. O. V. Dolomanov, L. J. Bourhis, R. J. Gildea, J. A. K. Howard and H. Puschmann, OLEX2: a complete structure solution, refinement and analysis program. *J. Appl. Cryst.*, 2009, **42**, 339-341.

S16. L. Palatinus and G. Chapuis, SUPERFLIP, *J. Appl. Cryst.,* 2007, **40**, 786-790.

S17. SHELXL, G. M. Sheldrick, *Acta Cryst.*, 2008, **A64**, 112-122.

S18. L. J. Barbour, X-Seed, Graphical Interface to SHELX97 and POV-Ray; University of Missouri-Columbia: Columbia, MO, 1999.

S19. C. Weetman, M. D. Anker, M. Arrowsmith, M. S. Hill, G. Kociok-Köhn, D. J. Liptrot and M. F. Mahon, *Chem. Sci.*, 2016, **7**, 628-641.

S20. Frisch, M. J. et al., Gaussian 09, Rev. D.01; Gaussian, Inc.: Wallingford, CT, 2010.

S21. Y. Zhao, D. G. Truhlar, The M06 suite of density functionals for main group thermochemistry, thermochemical kinetics, noncovalent interactions, excited states, and transition elements: two new functionals and systematic testing of four M06-class functionals and 12 other functionals. *Theor. Chem. Acc.*, 2008, **120**, 215-241.
